# Evaluation of RTS,S/AS02A and RTS,S/AS01B in Adults in a High Malaria Transmission Area

**DOI:** 10.1371/journal.pone.0006465

**Published:** 2009-07-31

**Authors:** Mark E. Polhemus, Shon A. Remich, Bernhards R. Ogutu, John N. Waitumbi, Lucas Otieno, Stella Apollo, James F. Cummings, Kent E. Kester, Christian F. Ockenhouse, Ann Stewart, Opokua Ofori-Anyinam, Isabelle Ramboer, Conor P. Cahill, Marc Lievens, Marie-Claude Dubois, Marie-Ange Demoitie, Amanda Leach, Joe Cohen, W. Ripley Ballou, D. Gray Heppner,

**Affiliations:** 1 US Army Medical Research Unit-Kenya, Nairobi, Kenya; 2 Kenya Medical Research Institute, Nairobi, Kenya; 3 Walter Reed Army Institute of Research, Silver Spring, Maryland, United States of America; 4 GlaxoSmithKline Biologicals s.a., Rixensart, Belgium; Walter and Eliza Hall Institute of Medical Research, Australia

## Abstract

**Background:**

This study advances the clinical development of the RTS,S/AS01B candidate malaria vaccine to malaria endemic populations. As a primary objective it compares the safety and reactogenicity of RTS,S/AS01B to the more extensively evaluated RTS,S/AS02A vaccine.

**Methodology:**

A Phase IIb, single centre, double-blind, controlled trial of 6 months duration with a subsequent 6 month single-blind follow-up conducted in Kisumu West District, Kenya between August 2005 and August 2006. 255 healthy adults aged 18 to 35 years were randomized (1∶1∶1) to receive 3 doses of RTS,S/AS02A, RTS,S/AS01B or rabies vaccine (Rabipur®; Chiron Behring GmbH) at months 0, 1, 2. The primary objective was the occurrence of severe (grade 3) solicited or unsolicited general (i.e. systemic) adverse events (AEs) during 7 days follow up after each vaccination.

**Principal Findings:**

Both candidate vaccines had a good safety profile and were well tolerated. One grade 3 systemic AE occurred within 7 days of vaccination (RTS,S/AS01B group). No unsolicited AEs or SAEs were related to vaccine. A marked increase in anti-CS antibody GMTs was observed post Dose 2 of both RTS,S/AS01B (31.6 EU/mL [95% CI: 23.9 to 41.6]) and RTS,S/AS02A (16.7 EU/mL [95% CI: 12.9 to 21.7]). A further increase was observed post Dose 3 in both the RTS,S/AS01B (41.4 EU/mL [95% CI: 31.7 to 54.2]) and RTS,S/AS02A (21.4 EU/mL [95% CI: 16.0 to 28.7]) groups. Anti-CS antibody GMTs were significantly greater with RTS,S/AS01B compared to RTS,S/AS02A at all time points post Dose 2 and Dose 3. Both candidate vaccines produced strong anti-HBs responses. Vaccine efficacy in the RTS,S/AS01B group was 29.5% (95% CI: −15.4 to 56.9, p = 0.164) and in the RTS,S/AS02A group 31.7% (95% CI: −11.6 to 58.2, p = 0.128).

**Conclusions:**

Both candidate malaria vaccines were well tolerated over a 12 month surveillance period. A more favorable immunogenicity profile was observed with RTS,S/AS01B than with RTS,S/AS02A.

**Trial Registration:**

Clinicaltrials.gov NCT00197054

## Introduction

*Plasmodium falciparum* is one of the most frequent causes of morbidity and mortality in areas where it is endemic [1], [2]. In Sub-Saharan Africa, *P. falciparum* causes the deaths of between 0.5 and 2.0 million children every year and is the most common reason for their admission to hospital [3]. Economic models have indicated that malaria may considerably retard economic development in African countries [4], [5]. Despite successful activities over the past century to decrease the land area suitable for malaria transmission, advances in understanding malaria ecology, and the development of interventions, the number of people at risk of malaria continues to increase [6]. As an adjunct to other interventions, the development of a safe, effective and affordable malaria vaccine is a critical global public health priority [7].

The RTS,S antigen adjuvanted with AS02A was developed by GlaxoSmithKline (GSK) Biologicals and tested in collaboration with the Walter Reed Army Institute of Research (WRAIR) since 1992 [8]. It is today the world's leading malaria candidate vaccine. The AS02 Adjuvant System contains an oil-in-water emulsion, the immunostimulant monophosphoryl lipid A (MPL) and QS21 (a natural saponin) molecule purified from the bark of the South American tree *Quillaja saponaria* [9], [10].

The RTS,S/AS02A vaccine has been shown to have an acceptable safety profile, to be immunogenic and to provide complete or partial protection against infection in malaria-naïve adults [11]–[16] undergoing experimental challenge. Similarly, this vaccine has shown an acceptable safety profile, robust immunogenicity and has conferred partial protection against infection and/or clinical malaria in adults [17]–[20] children [21]–[25], and infants [26] living in malaria-endemic areas.

The RTS,S/AS01B formulation has been developed in parallel with the aim of improving the immune response and vaccine efficacy. The AS01 Adjuvant System is based on liposomes and contains the same amounts of MPL and QS21 as AS02. Preclinical studies suggested that the liposomal formulation AS01 is more immunogenic than the oil-in-water emulsion formulation AS02 [27]–[29]. In healthy malaria-naïve adults both vaccines were equally well tolerated, however, RTS,S/AS01B was significantly more immunogenic than RTS,S/AS02A and showed a strong trend for greater efficacy [30].

The aims of this study were to evaluate RTS,S/AS01B and RTS,S/AS02A in adults in a malaria-endemic region. The primary objective was to compare the safety profile of RTS,S/AS01B to that of RTS,S/AS02A in adults to determine if RTS,S/AS01B should proceed to evaluation in children. Secondary objectives included evaluations of immunogenicity and efficacy.

## Methods

The protocol for this trial and supporting CONSORT checklist are available as supporting information; see Checklist S1 and Protocol S1.

### Ethics statement

The protocol was approved by the KEMRI and Kenya National Ethical Review Committee, Nairobi, and the US Army Medical Research and Materiel Command's Human Subjects Research Review Board, Fort Detrick, Maryland. The trial was undertaken according to the International Conference on Harmonization, Good Clinical Practice guidelines and was monitored by GSK Biologicals. A Local Safety Monitor and a Safety Monitoring Group closely reviewed the conduct and results of the trial.

**Figure 1:**
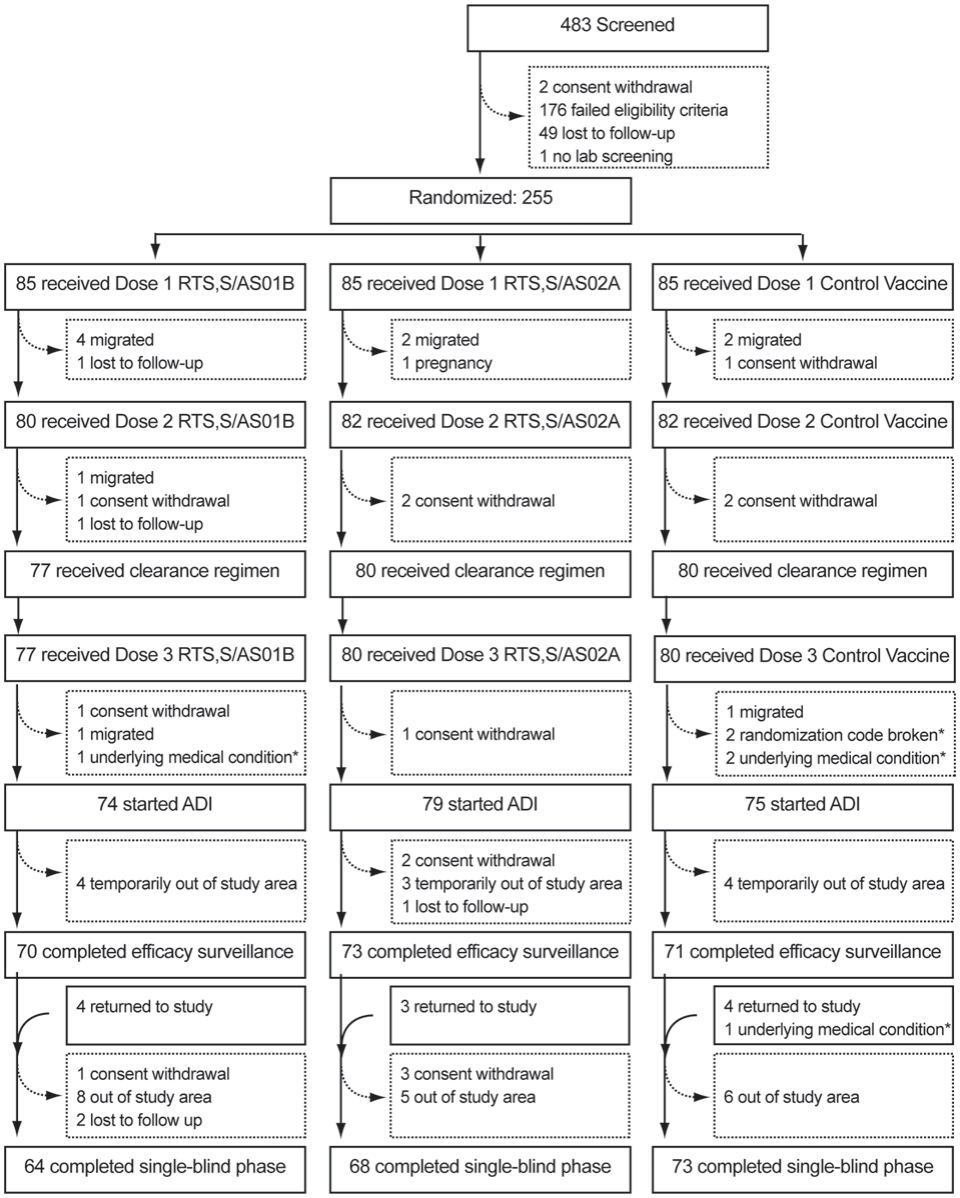
CONSORT diagram for study participants. 176 failed eligibility criteria: 13 outside age range [18–35]; 9 not available for the whole study duration (12 months); 44 not free of obvious health problem (med history/clin exam); 14 female of childbearing potential not using adequate contraceptives; 8 confirmed or suspected HIV; 10 acute disease at time of enrolment; 27 acute or chronic clinically significant pulmonary, cardiovascular hepatic or renal functional abnormality; 6 ALT outside range; 15 hemoglobin outside range; 9 history of chronic alcohol/drug use; 21 other (including pregnant, administration of IG/blood products, sickle cell disease, HBsAg positive, other safety labs outside range, history of seizures, or allergic reactions, planned administration of non-study vaccine). Note: Underlying medical conditions were not detected at screening. ADI: active detection of infection. * These subjects did not complete ADI assessments, but were followed up for safety assessments and appear in the total of completed single-blind phase.

Following an information campaign, local consultation, exhaustive informed consent process and screening, 255 adult volunteers between 18 and 35 years were enrolled to the trial (refer to Figure 1 for an overview of study recruitment). Written informed consent or, in case of illiteracy, a thumb print in the presence of a literate witness was obtained before study procedures began.

### Participants

The trial was conducted at the KEMRI-Walter Reed Project's Kombewa Clinical Research Center in healthy adults almost exclusively of the Luo tribe, predominantly Seme sub-tribe, aged 18 to 35 years recruited from Kombewa Division, Kisumu West District, Nyanza Province of Western Kenya.

The climate is tropical. There are two intense malaria transmission periods from April to August - the ‘long rains’ - and from October to December - the ‘short rains’. Malaria disease is primarily a result of infection with *P. falciparum*.

All volunteers had their medical histories taken and a full medical examination was conducted. Volunteers were excluded if they had any confirmed or suspected immunodeficient condition, history of allergic reactions to immunizations, history of neurologic disorders or seizures, clinically significant acute disease at time of enrolment, were pregnant (or lactating) or planning to become pregnant, were positive for HBsAg, a history of drug or alcohol abuse, were positive for homozygous sickle cell disease, or had significantly abnormal tests of renal function, or of hepatic or hematologic parameters. Female volunteers of childbearing potential were only enrolled if they had used adequate contraceptive precautions for 30 days prior to vaccination and agreed to continue such precautions for two months after completion of the vaccination series.

### Procedures and interventions

Any volunteers who were found to have a medical condition that excluded them from participation in the trial were informed at a private appointment with a member of the clinical staff. Time was taken to fully counsel the volunteer on the causes and severity of their condition, any implications the condition might have on their lifestyle, and evaluation and treatment options. Where appropriate, volunteers were then referred to speciality or sub-speciality physicians in the local area capable of dealing with the volunteers' condition in an appropriate manner.

**Figure 2:**
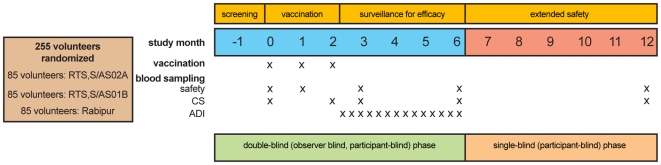
Study design overview. ADI = active detection of infection. CS = circumsporozoite protein.

Recipients of candidate vaccine were administered 50 µg of lyophilized RTS,S reconstituted with 500 µL of either AS02A or AS01B Adjuvant Systems. Both candidate and control vaccines were administered intramuscularly to the deltoid muscle of the non-dominant arm on a 0, 1, 2-month schedule (Figure 2). Vaccinees were observed for 30 minutes following each vaccination.

Volunteers were followed daily for the solicited adverse events (AEs) of pain, swelling, fever, fatigue, gastrointestinal problems, headache, joint pain and muscle ache for a total of 7 days following each vaccination and for unsolicited AEs for 30 days following each dose; serious AEs (SAEs) were recorded throughout the study period. Blood draws for safety evaluation and humoral responses were taken at scheduled time points during the study (Figure 2).

The study population was drawn from one-mile radius catchment areas around each of 13 field stations. The field stations were staffed 24 hours/day throughout the study period to facilitate referral medical care and were 0.5 to 10 miles distant from the Kombewa Clinical Research Center.

One week prior to vaccine Dose 3 all volunteers were presumptively treated with three daily doses of Malarone® (atovaquone and proguanil hydrochloride, GSK, Uxbridge, UK) administered under direct observation by the study staff. All subjects were re-checked for asexual *P. falciparum* parasitemia one week post Dose 3. Any subject who tested positive would have been treated with a second line drug, and the absence of parasitemia again confirmed just prior to inclusion in the efficacy evaluation which started 2 weeks after Dose 3.

Efficacy evaluation included both active detection of infection (ADI) with weekly blood draws and passive case detection in all volunteers presenting with symptoms consistent with malaria.

### Objectives

The study was a Phase II, controlled, randomized, double-blind study of 12 months duration of two candidate malaria vaccine formulations, RTS,S/AS02A and RTS,S/AS01B. The study was prospectively designed to analyse safety, immunogenicity and efficacy endpoints over a 6 month surveillance period in a double-blinded fashion. A subsequent 6 month single-blind follow-up was defined for the assessment of safety and immunogenicity . The primary objective was to compare the safety and reactogenicity of RTS,S/AS01B to RTS,S/AS02A in adults in Kenya. Secondary objectives were to describe the safety of the vaccine candidates, describe antibody responses to the circumsporozoite (anti-CS) antigen and hepatitis B surface antigen (anti-HBs) and to assess efficacy against infection with *P. falciparum* malaria (defined as *P. falciparum* asexual parasitemia>0/µL by microscopy) over a period of 14 weeks starting two weeks post Dose 3.

### Outcomes: safety

The analysis for safety was conducted on the total vaccinated cohort (defined as all subjects receiving at least one dose of study vaccine).

The primary safety outcome was the occurrence of grade 3 solicited or unsolicited general reactions (i.e. systemic reactions) after each vaccination during a 7 day follow-up period. Grade 3 general reactions (solicited or unsolicited) were defined as those that prevented normal daily activity, or in the case of fever an oral temperature >39.0°C. Grade 3 solicited pain at the injection site was defined as preventing normal daily activities, or in the case of swelling at the injection site, swelling that exceeded 50 mm in diameter. Secondary safety outcomes included the occurrence of SAEs until 10 months post Dose 3, unsolicited AEs after each vaccination over a 30 day follow-up period (day of vaccination and 29 subsequent days), solicited general and local reactions over a 7 day follow-up period (day of vaccination and 6 subsequent days) after each vaccination, abnormal hematological, renal, and hepatic parameters.

### Outcomes: immunogenicity

The primary analysis for immunogenicity was conducted on the ATP cohort for immunogenicity, defined as all evaluable subjects (i.e. those meeting all eligibility criteria, complying with the procedures defined in the protocol, with no elimination criteria during the study) for whom data concerning immunogenicity endpoint measures were available.

Anti-CS and anti-HBs humoral responses were evaluated as secondary outcomes: anti-CS at baseline, 1 month post Dose 2, 1 month post Dose 3, 4 months post Dose 3 and 10 months post Dose 3, and anti-HBs at baseline, 1 month post Dose 3 and 10 months post Dose 3. Antibody levels against CS were measured in Elisa units/milliliter (EU/mL) by standard ELISA methodology using plate-adsorbed R32LR antigen [NVDP(NANP)_15_]_2_LR [24]. Anti-HBsAg was measured using a commercially available ELISA immunoassay (AUSAB EIA test kit from Abbott).

### Outcomes: efficacy

The primary analysis for efficacy was conducted on the ATP cohort for efficacy defined as all evaluable subjects (i.e. those meeting all eligibility criteria, complying with the procedures defined in the protocol, with no elimination criteria during the study) for whom data concerning efficacy endpoint measures were available.

First episodes of infection with *P. falciparum* (first recording of infection with asexual stage parasites detected by ADI or passive case detection)were assessed by weekly sampling over a period starting 14 days after Dose 3 and extending for a 14 week duration. A cross-sectional evaluation of asexual *P. falciparum* parasitemia (prevalence and density) at 16 weeks post Dose 3 was conducted. Parasitemia was determined by microscopy. Each slide was read independently by two microscopists, each of whom examined 100 oil immersion fields before declaring a slide to be negative. Parasite density was assessed by counting the number of asexual parasites per 200 leukocytes. Parasite densities were then calculated using a concomitant leukocyte count.

The percentage change in hemoglobin value between baseline and 16 weeks post Dose 3 was an exploratory outcome.

### Sample Size

For the primary objective, the study had a 90% power to detect a difference between the proportion of subjects experiencing a grade 3 solicited or unsolicited general reaction following vaccination, should the difference in the proportion afflicted exceed approximately 20% to 30%, depending on the rates in the control group (rates of between 5% and 50% were assumed).

For the secondary vaccine efficacy endpoint, the study had 90% power to detect significant (p<0.05) vaccine efficacy of either candidate malaria vaccine versus control assuming an infection rate of 72% over the 14 week period for surveillance of infection and a true vaccine efficacy of 45%.

### Randomization Procedures: sequence generation, allocation concealment, implementation

Volunteers were randomized 1∶1∶1 to receive RTS,S/AS02A or RTS,S/AS01B or Rabipur®. A randomization list (block randomization) was provided and subjects were allocated to treatment on the day of first dose. Subjects were allocated sequentially to treatment numbers in the order that they presented for vaccination. Treatment numbers were assigned to vaccines with a randomization list generated using a standard SAS® program (Statistical Analysis System).

### Blinding

Due to the differences in visual appearance of each of the study vaccines, blinding was maintained by preparation of the vaccines by the pharmacy staff in an area separate from where vaccination occurred. Post-vaccination evaluations of AEs were conducted by a separate team.

### Statistical methods

#### Safety

Analysis was carried out according to a report and analysis plan established before unblinding of trial data.

The proportion of subjects with a grade 3 solicited or unsolicited general reaction, following each vaccination, was tabulated with exact 95% confidence intervals (CI). Comparisons between groups were conducted using Fisher's Exact Test. The proportion of subjects experiencing SAEs or AEs as classified at the MedDRA (Medical Dictionary for Regulatory Activities) preferred term level was tabulated by group. The percentage of subjects experiencing AEs and the percentage of doses followed by AEs were tabulated by group. Fever, temperature in 0.5°C increments, grade 3 events, and the relationship of events to vaccination as judged by the investigator were investigated.

At each blood sampling timepoint biochemical parameters (ALT and creatinine) above normal range and hematological parameters (hemoglobin, total white blood cell count, platelets and absolute lymphocyte count) below normal range were described.

#### Immunogenicity

Geometric mean titers (GMTs) of anti-CS and anti-HBs antibodies, seropositivity rates of anti-CS and anti-HBs antibodies and seroprotection rates of anti-HBs antibodies were summarized with 95% CI. Analyses by vaccine group and by infection status (subjects were regarded as ‘non-infected’ if no malaria parasites were detected during the active detection of cases) were performed.

#### Efficacy

Vaccine efficacy (VE) against infection was assessed with Cox regression models, defined as 1 minus the hazard ratio. Time at risk was corrected for absences from the study area and for antimalarial treatment. The proportional hazards assumption was investigated graphically, using a test based on the Schoenfeld residuals. An adjusted analysis for VE was performed for covariates of age, sickle cell trait, village of residence, and distance of residence from the Kombewa Clinic. Bednets were not distributed as part of the trial and data were not collected on the use of bednets during the trial.

Prevalence and density of asexual *P. falciparum* parasitemia was also assessed at 16 weeks post Dose 3 (end of the double-blind phase). Assessment of parasite prevalence was assessed using Fisher's exact test. The percentage change in hemoglobin between baseline and the end of the double-blind phase of the study was assessed.

Analyses were done using SAS version 8 (SAS, Cary, NC, USA).

## Results

### Participant flow

483 volunteers were screened and 255 were enrolled to the study and were evaluated for safety; 85 to each vaccine group (see Figure 1). 237 subjects received all 3 vaccine doses as scheduled and 228 were evaluable for surveillance for infection.

### Recruitment

Recruitment commenced in July 2005. Vaccination took place between August and October 2005; all three doses were completed just prior to a period of increased malaria transmission (the short rainy season). Surveillance for infection ran from October 2005 to January 2006. The study completed in August 2006.

### Baseline Data

There was no significant difference in baseline characteristics between the three study groups (Table 1). The According to Protocol (ATP) and Total vaccinated cohorts were comparable.

**Table 1:** Baseline characteristics and distribution of covariates of study participants (Total vaccinated cohort).

		RTS,S/AS01B N = 85		RTS,S/AS02A N = 85		Rabies N = 85		Total N = 255	
		n	%	n	%	n	%	n	%
Age (years)	Mean	24.9	–	25.2	–	26.1	–	25.4	–
	Range	17 to 35		18 to 35		18 to 35		17 to 35	
Gender	Female	21	24.7	19	22.4	12	14.1	52	20.4
	Male	64	75.3	66	77.6	73	85.9	203	79.6
Village of residence	ABOL	6	7.1	6	7.1	4	4.7	16	6.3
	BARKORWA	3	3.5	3	3.5	6	7.1	12	4.7
	GOT AGULU	7	8.2	7	8.2	5	5.9	19	7.5
	KITARE	12	14.1	8	9.4	9	10.6	29	11.4
	KUOYO KOWE	2	2.4	1	1.2	3	3.5	6	2.4
	MANYWANDA	3	3.5	4	4.7	5	5.9	12	4.7
	MIRIERI	7	8.2	7	8.2	6	7.1	20	7.8
	NDURU KADERO	5	5.9	6	7.1	2	2.4	13	5.1
	ORUGA	6	7.1	7	8.2	11	12.9	24	9.4
	OSEWRE	6	7.1	4	4.7	4	4.7	14	5.5
	RANEN	5	5.9	2	2.4	0	0.0	7	2.7
	RERU	2	2.4	5	5.9	1	1.2	8	3.1
	WRP	21	24.7	25	29.4	29	34.1	75	29.4
Distance from Kombewa Clinic	0–5 km	21	24.7	25	29.4	29	34.1	75	29.4
	5–10 km	16	18.8	15	17.6	13	15.3	44	17.3
	10–20 km	48	56.5	45	52.9	43	50.6	136	53.3
Sickle Cell Trait positive		19	22.4	19	22.4	23	27.1	61	23.9

### Numbers analyzed

All 255 planned subjects received at least Dose 1 of vaccine and were included in the Total vaccinated cohort analysis. 222 subjects were included in the ATP cohort for immunogenicity (75 recipients of RTS,S/AS01B, 74 recipients of RTS,S/AS02A, and 73 recipients of rabies vaccine). 228 subjects were included in the ATP cohort for efficacy (74 RTS,S/AS01B, 79 RTS,S/AS02A, and 75 rabies vaccine).

### Outcomes and estimation

#### Safety outcomes

There was only one grade 3 general symptom occurring within 7 days of vaccination (primary endpoint). One recipient of RTS,S/AS01B had a fever of 39.7°C (grade 3) one day after receiving Dose 2 in association with a confirmed episode of malaria (*P. falciparum* density 4735 parasites/µL).

There were similar rates of solicited general symptoms, irrespective of intensity, in all three groups (96.5% RTS,S/AS01B, 94.1% RTS,S/AS02A and 96.5% control, per dose). Most reactions were mild and transient. The frequency of solicited local and general symptoms, overall and by dose, is provided in Table 2.

**Table 2:** Incidence of solicited local and general adverse events within 7 days per dose and overall per dose (Total vaccinated cohort).

		RTS,S/AS01B						RTS,S/AS02A						Rabies vaccine					
		All			Grade 3			All			Grade 3			All			Grade 3		
		N	n	%	N	n	%	N	n	%	N	n	%	N	n	%	N	n	%
Pain	Dose 1	85	58	68.2	85	0	0.0	85	72	84.7	85	1	1.2	85	18	21.2	85	0	0.0
	Dose 2	80	42	52.5	80	0	0.0	82	54	65.9	82	0	0.0	82	26	31.7	82	0	0.0
	Dose 3	77	32	41.6	77	0	0.0	80	40	50.0	80	0	0.0	80	24	30.0	80	0	0.0
	Overall	242	132	54.5	85	0	0.0	247	166	67.2	247	1	0.4	247	68	27.5	85	0	0.0
Swelling	Dose 1	85	7	8.2	85	0	0.0	85	19	22.4	85	4	4.7	85	2	2.4	85	0	0.0
	Dose 2	80	3	3.8	80	0	0.0	82	1	1.2	82	0	0.0	82	0	0.0	82	0	0.0
	Dose 3	77	2	2.6	77	0	0.0	80	2	2.5	80	0	0.0	80	0	0.0	80	0	0.0
	Overall	242	12	5.0	85	0	0.0	247	22	8.9	247	4	1.6	247	2	0.8	85	0	0.0
Fatigue	Dose 1	85	19	22.4	85	0	0.0	85	17	20.0	85	0	0.0	85	19	22.4	85	0	0.0
	Dose 2	80	15	18.8	80	0	0.0	82	10	12.2	85	0	0.0	82	10	12.2	82	0	0.0
	Dose 3	77	9	11.7	77	0	0.0	80	6	7.5	80	0	0.0	80	6	7.5	80	0	0.0
	Overall	242	43	17.8	85	0	0.0	247	33	13.4	85	0	0.0	247	35	14.2	85	0	0.0
Fever	Dose 1	85	7	8.2	85	0	0.0	85	6	7.1	85	0	0.0	85	7	8.2	85	0	0.0
	Dose 2	80	7	8.8	80	1	1.3	82	3	3.7	82	0	0.0	82	3	3.7	82	0	0.0
	Dose 3	77	7	9.1	77	0	0.0	80	5	6.3	80	0	0.0	80	2	2.5	80	0	0.0
	Overall	242	21	8.7	242	1	0.4	247	14	5.7	85	0	0.0	247	12	4.9	85	0	0.0
Gastrointestinal	Dose 1	85	15	17.6	85	0	0.0	85	12	14.1	85	0	0.0	85	21	24.7	85	0	0.0
	Dose 2	80	15	18.8	85	0	0.0	82	12	14.6	82	0	0.0	82	12	14.6	82	0	0.0
	Dose 3	77	18	23.4	77	0	0.0	80	18	22.5	80	0	0.0	80	11	13.8	80	0	0.0
	Overall	242	48	19.8	85	0	0.0	247	42	17.0	85	0	0.0	247	44	17.8	85	0	0.0
Headache	Dose 1	85	34	40.0	85	0	0.0	85	44	51.8	85	0	0.0	85	33	38.8	85	0	0.0
	Dose 2	80	24	30.0	80	0	0.0	82	26	31.7	82	0	0.0	82	23	28.0	82	0	0.0
	Dose 3	77	26	33.8	77	0	0.0	80	25	31.3	80	0	0.0	80	11	13.8	80	0	0.0
	Overall	242	84	34.7	85	0	0.0	247	95	38.5	85	0	0.0	247	67	27.1	85	0	0.0
Joint pain at other location	Dose 1	85	8	9.4	85	0	0.0	85	8	9.4	85	0	0.0	85	12	14.1	85	0	0.0
	Dose 2	80	11	13.8	80	0	0.0	82	8	9.8	82	0	0.0	82	4	4.9	82	0	0.0
	Dose 3	77	8	10.4	77	0	0.0	80	6	7.5	80	0	0.0	80	6	7.5	80	0	0.0
	Overall	242	27	11.2	85	0	0.0	247	22	8.9	85	0	0.0	247	22	8.9	85	0	0.0
Muscle aches	Dose 1	85	14	16.5	85	0	0.0	85	9	10.6	85	0	0.0	85	15	17.6	85	0	0.0
	Dose 2	80	5	6.3	80	0	0.0	82	7	8.5	82	0	0.0	82	6	7.3	82	0	0.0
	Dose 3	77	8	10.4	77	0	0.0	80	7	8.8	80	0	0.0	80	8	10.0	80	0	0.0
	Overall	242	27	11.2	85	0	0.0	247	23	9.3	85	0	0.0	247	29	11.7	85	0	0.0

N = number of administered doses.

n/% = number/percentage of doses followed by at least one type of symptom.

Injection site pain was the most frequently reported local symptom in all vaccine groups. The incidence of pain was significantly lower in the RTS,S/AS01B group (reported following 54.5% of doses) compared to the RTS,S/AS02A group (reported following 67.2% of doses) (p = 0.005); pain was reported following 27.5% of doses of rabies vaccine. The incidence of swelling was also slightly lower in the RTS,S/AS01B group (following 5.0% of doses) compared to the RTS,S/AS02A group (following 8.9% of doses); swelling was reported following 0.8% of doses of rabies vaccine. Five grade 3 solicited local AEs were reported, all occurring in the RTS,S/AS02A group (1 pain, 4 swelling). All severe events occurred after Dose 1 of RTS,S/AS02A and all resolved within the 7 day follow-up period.

96.1% of subjects reported an unsolicited AE within 30 days of vaccination; four were grade 3 (3 RTS,S/AS01B, 1 rabies). No unsolicited AE was judged to be related to vaccination by the investigator.

Fourteen subjects had hemoglobin (Hb) levels below the normal range (males<11 g/dL, females<9.5 g/dL) (8 RTS,S/AS01B, 4 RTS,S/AS02A, 2 rabies) the majority occurring 6 days post Dose 1 (RTS,S/AS01B n = 4; RTS,S/AS02A n = 1; rabies n = 1) and 6 days post Dose 3 (RTS,S/AS01B n = 1; RTS,S/AS02A n = 1; rabies n = 1). The lowest recorded Hb values in the RTS,S/AS01B group ranged from 8.5–10.5 g/dL, in the RTS,S/AS02A group from 9.1–10.6 g/dL and in the rabies vaccine group from 8.4–9.9 g/dL. Hemoglobin levels had not normalised by the last laboratory assessment in 7 subjects (5 RTS,S/AS01B, 2 RTS,S/AS02A). The low hemoglobin was attributed, in the 5 subjects from the RTS,S/AS01B group, to chronic anemia, microcytic anemia, infectious bloody diarrhea, immunosuppression and pregnancy, and in the 2 subjects from the RTS,S/AS02A group, to microcytic anemia in both cases.

Few subjects had WBC levels below the normal range (males<3×10^3^/µL, females<2.5 ×10^3^/µL) (4 RTS,S/AS01B, 2 rabies group) or lymphocyte counts below the normal range (<1×10^3^/µL) (2 RTS,S/AS01B, 1 rabies group). No subjects had abnormal platelet levels (above 77 000 per mm^3^) during the study.

Eight subjects had ALT levels above the normal range (males≥60 IU/mL, females≥40 IU/mL) (5 RTS,S/AS02A, 3 rabies), first recorded 6 days post Dose 1 in all 5 subjects in the RTS,S/AS02A group and 1 month post Dose 3 in all 3 subjects in the rabies group. All out of range values were mild in intensity (<2.5×Upper Limit of Normal [ULN]), judged not to be clinically significant and not investigated further.

Eleven subjects had bilirubin values above the normal range (>1.48 mg/dL) which onset during the study (3 RTS,S/AS01B, 3 RTS,S/AS02A, 5 rabies group), first recorded 6 days post Dose 1 in 4 subjects (2 RTS,S/AS01B; 1 RTS,S/AS02A; 1 rabies) and 1 month post Dose 3 in 7 subjects (1 RTS,S/AS01B; 2 RTS,S/AS02A; 4 rabies). The highest recorded bilirubin values in recipients of RTS,S/AS01B were 1.50–2.65 mg/dL, in recipients of RTS,S/AS02A 1.51–2.42 mg/dL and in recipients of rabies vaccine 1.52–2.34 mg/dL. All bilirubin elevations were judged not to be clinically significant and not investigated further. None were associated with any other liver test abnormality. No subjects had abnormal creatinine levels (outside range 0.45 to 1.0 mg/dL) during the study.

Nine subjects became pregnant during the course of the study: 4 recipients of RTS,S/AS01B, 3 of RTS,S/AS02A, and 2 of rabies vaccine. One recipient of RTS,S/AS01B had a stillbirth at 27 weeks of pregnancy for which no cause was identified. This occurred 42 weeks after the administration of the third dose and the blinded investigator considered the stillbirth unlikely to be related to vaccination. In the other eight cases, the mothers gave birth to healthy infants.

During the entire 12 month study duration, 5 subjects from the RTS,S/AS01B group (5.9%), 1 subject (1.2%) from the RTS,S/AS02A group and 5 subjects (5.9%) from the rabies group reported at least one SAE. None were considered to be related to study vaccine and none were fatal. No volunteer withdrew due to an AE.

SAEs resulting in hospitalisation were reported by two subjects during the trial, both recipients of RTS,S/AS01B. One subject, a 26 year old woman was admitted to hospital 29 days post Dose 3 suffering from bleeding peptic ulcer disease. She was discharged from hospital after two days. The second subject, a 36 year old man, was diagnosed with pneumonia, pulmonary tuberculosis, meningitis and HIV infection five months post Dose 3. Following resolution of the meningitis, the subject was discharged with medication for the tuberculosis and HIV infections. The volunteer received counselling and was enrolled in a Ministry of Health approved HIV care and treatment program.

#### Immunogenicity outcomes

**Figure 3:**
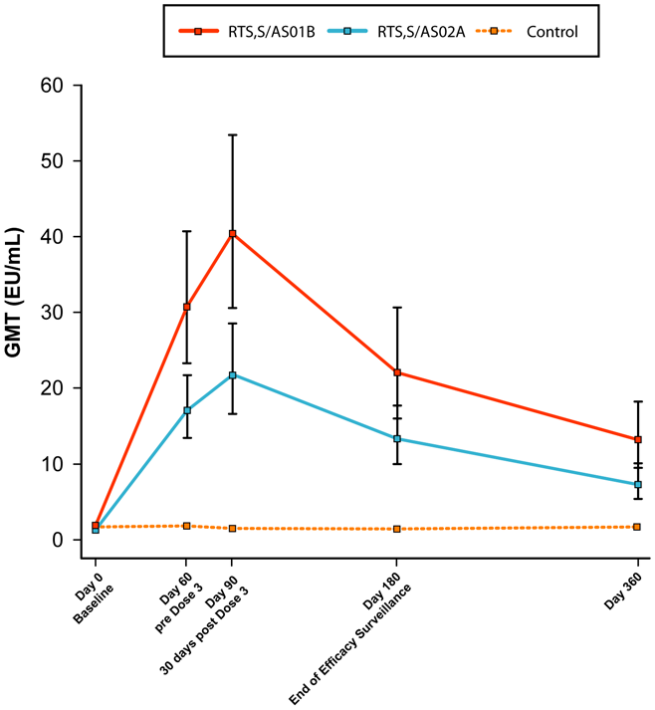
Anti-CS GMTs over time (ATP cohort for immunogenicity). Note: bars represent 95% confidence intervals.

Both malaria vaccines were immunogenic for anti-CS antibodies, with RTS,S/AS01B producing a significantly more robust response than RTS,S/AS02A (Figure 3). Pre-vaccination anti-CS GMTs were low and equivalent in the study groups. A marked increase in anti-CS antibody GMTs was observed post Dose 2 of both RTS,S/AS01B (31.6 EU/mL [95% CI: 23.9 to 41.6]) and RTS,S/AS02A (16.7 EU/mL [95% CI: 12.9 to 21.7]). A further increase was observed post Dose 3 in both the RTS,S/AS01B (41.4 EU/mL [95% CI: 31.7 to 54.2]) and RTS,S/AS02A (21.4 EU/mL [95% CI: 16.0 to 28.7]) groups. Anti-CS antibody GMTs were significantly greater with RTS,S/AS01B compared to RTS,S/AS02A 1 month post Dose 2 (Day 60, p≤0.001), 1 month post Dose 3 (Day 90, p≤0.001), 4½ months post Dose 3 (Month 6½; p = 0.003) and 10 months post Dose 3 (Month 12; p = 0.002). Although anti-CS antibody GMTs decreased at 6 and 10 months post Dose 3, they remained higher in the RTS,S/AS01B compared to the RTS,S/AS02A group, and significantly greater in the candidate vaccine groups versus the rabies control group.

Both candidates produced strong anti-HBs responses (Table 3). A marked increase in anti-HBs GMTs was observed in recipients of candidate malaria vaccines 1 month post Dose 3 (Day 90); GMTs were similar in the RTS,S/AS01B (1435 mIU/mL [95% CI: 743 to 2771]) and RTS,S/AS02A (1714 mIU/mL [95% CI: 836 to 3515]) groups. Although anti-HBs antibody GMTs had decreased at 6 and 10 months post Dose 3 in both the RTS,S/AS01B and RTS,S/AS02A groups, GMTs were greater than the rabies control group. At baseline, 40% of subjects in the RTS,S/AS01B group and 49% of subjects in the RTS,S/AS02A group were seroprotected for anti-HBs, and at 10 months post Dose 3 (Month 12) seroprotection had increased to 90% and 92% respectively.

**Table 3:** Seropositivity rates, seroprotection rates and GMTs for anti-HBs antibodies by vaccine group (ATP cohort for immunogenicity).

Group	Timing		Seropositive				Seroprotected				GMTs (mIU/mL)		
		N	n	%	95% CI		n	%	95% CI		value	95% CI	
RTS,S/AS01B	PRE	73	31	43	31	55	29	40	29	52	11	6	20
	PIII(D90)	72	68	94	86	99	65	90	81	96	1435	743	2771
	PIII(M12)	61	57	93	84	98	55	90	80	96	594	317	1112
RTS,S/AS02A	PRE	70	38	54	42	66	34	49	36	61	18	9	35
	PIII(D90)	72	68	94	86	99	67	93	85	98	1714	836	3515
	PIII(M12)	62	59	95	87	99	57	92	82	97	636	342	1183
Rabies vaccine	PRE	68	35	52	39	64	33	49	36	61	24	11	49
	PIII(D90)	65	37	57	44	69	33	51	38	63	32	15	69
	PIII(M12)	66	40	61	48	72	33	50	37	63	29	14	59

Seropositive≥3.3 mIU/mL; Seroprotected≥10 mIU/mL.

GMT = geometric mean antibody titer calculated on all subjects.

PRE = prevaccination; PIII(D90) = post Dose 3 (Day 90); PIII(M12) = post Dose 3 (Month 12).

#### Efficacy outcomes

**Figure 4:**
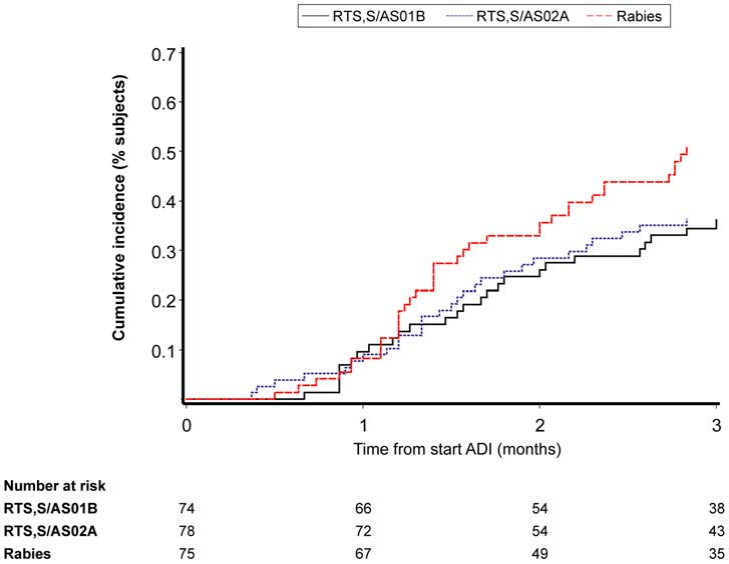
Vaccine Efficacy: reverse cumulative curve showing the time to infection with malaria by vaccine group (ATP cohort for efficacy). Gr 1 = RTS,S/AS01B; Gr 2 = RTS,S/AS02A; Gr 3 = Rabies; Day 0 = 14 days post Dose 3; ADI = Active Detection of Infection.

Table 4 and Figure 4 show the results for VE against infection. VE in the RTS,S/AS01B group was 29.5% (95% CI: −15.4 to 56.9, p = 0.164 vs control) and in the RTS,S/AS02A group 31.7% (95% CI: −11.6 to 58.2, p = 0.128 vs control); pooled data yielded an unadjusted VE of 30.9% (95% CI: –4.7 to 54.4, p = 0.081 vs control).

**Table 4:** Vaccine efficacy against *P. falciparum* infection (ATP cohort for efficacy).

Group	N	n	PYAR	Rate	Point estimate of VE unadjusted for covariates				Point estimate of VE adjusted for covariates*			
					%	95% CI		P value	%	95% CI		P value
RTS,S/AS01B	74	28	14.1	1.99	29.5	−15.4	56.9	0.164	11.0	−49.7	47.1	0.659
RTS,S/AS02A	79	28	14.6	1.92	31.7	−11.6	58.2	0.128	35.1	−9.9	61.6	0.108
RTS,S/AS01B & RTS,S/AS02A	153	56	28.7	1.95	30.9	−4.70	54.4	0.081	23.4	−18.3	50.4	0.229
Rabies control	75	37	13.2	2.80	–	–	–	–	–	–	–	

N = number of subjects.

n = number of subjects with an episode of parasitemia.

PYAR = person years at risk.

VE = vaccine efficacy.

* adjusted for age, sickle cell trait, village of residence and distance of residence from Kombewe clinic.

VE adjusted for age, sickle cell trait, village of residence and distance of residence from the Kombewa Clinical Research Center was 11.0% (95% CI −49.7 to 47.1: p = 0.659) in recipients of RTS,S/AS01B and 35.0% (95% CI −9.9 to 61.6: p = 0.108) in recipients of RTS,S/AS02A. Adjustments not taking into account village of residence were similar to unadjusted analyses. Further analyses were carried out to explain this finding, including an examination of the role of baseline anti-CS antibody, but did not reveal any obvious factors.

The proportion of subjects positive for parasitemia at 16 weeks post dose 3 was similar in all the groups: RTS,S/AS01B 8.6% (95% CI 3.2 to 17.7), RTS,S/AS02A 6.8% (95% CI 2.3 to 15.3) and rabies control 4.2% (95% CI 0.9 to 11.9). The geometric mean parasite densities of the positives were: RTS,S/AS01B 275 parasites per µL (95% CI 33 to 2295), RTS,S/AS02A 206 parasite per µL (95% CI 51 to 843) and rabies control 521 parasites per µL (95% CI 42 to 6470).

There was no difference in mean hemoglobin levels between baseline and 16 weeks post Dose 3 for any group (data not shown).

#### Association of immunogenicity and efficacy

**Figure 5:**
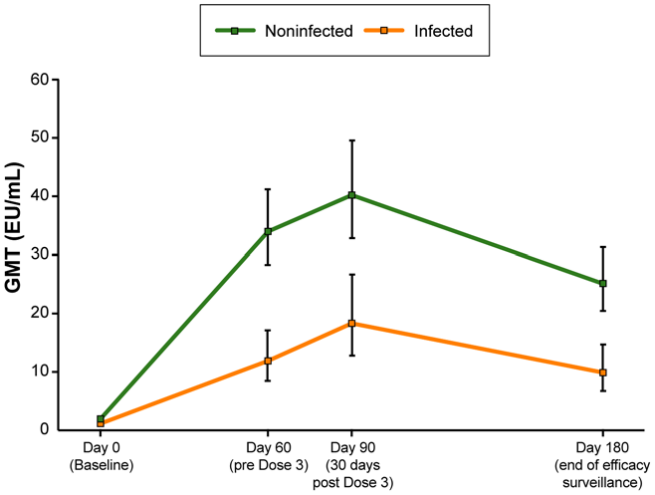
Anti-CS GMTs during efficacy surveillance by infection status (ATP cohort for efficacy).

Ancillary analyses were performed to evaluate the association of anti-CS antibodies with VE, irrespective of vaccine group. The analysis was restricted to recipients of candidate malaria vaccines and compared anti-CS GMTs between subjects that did, and those that did not, become infected with malaria during the 14 week follow-up period, from 14 days to 4 months post Dose 3 (infection was defined as *P. falciparum* asexual parasitemia >0/µL detected on a scheduled ADI visit or by passive case detection). During this assessment period, anti-CS antibody GMTs were higher in non-infected compared to infected subjects, which was statistically significant at 1 month post Dose 2 (Day 60; p<0.0001), 1 month post Dose 3 (Day 90; p = 0.0007) and 4½ months post Dose 3 (Month 6 ½; p<0.0001) (Figure 5).

## Discussion

### Interpretation

This paper presents the first safety, immunogenicity, and efficacy data for the candidate RTS,S malaria vaccine combined with the novel Adjuvant System AS01B when administered to adult subjects in a malaria endemic region.

Overall, both the RTS,S/AS02A and RTS,S/AS01B formulations were shown to have a good safety profile and were well tolerated. With respect to the primary endpoint of the study, the occurrence of severe (grade 3) systemic reactions occurring for up to 7 days after each vaccination, one severe event was observed (fever) in the RTS,S/AS01B group and none in either the RTS,S/AS02A candidate vaccine group or the rabies control group. The investigator considered the fever to be unrelated to the study vaccine and due to concurrent malaria. Additional safety and reactogenicity data showed the RTS,S/AS01B candidate vaccine to compare favorably to the RTS,S/AS02A candidate vaccine in terms of solicited local events (pain and swelling). Both candidate vaccines were well tolerated; no subject withdrew due to an AE and no unsolicited AEs or serious AEs were considered to be related to study vaccine.

The safety and tolerability of the RTS,S/AS02A candidate vaccine observed in this study conducted in Kenyan adults is consistent with studies conducted in young African children from The Gambia, Mozambique and Tanzania [21]–[26], [31]. RTS,S/AS01B had never before been evaluated under field conditions; evaluation of the safety and reactogenicity of RTS,S/AS01B in adults is a crucial requirement before evaluation in children. The favorable safety profile of RTS,S/AS01B supported progression of this candidate vaccine to further studies in children [32].

Both candidate vaccines were immunogenic for anti-CS and anti-HBs antibodies. The level of anti-CS response to the RTS,S/AS02A vaccine was consistent with previous studies conducted in African adults [17], [18], [20], and is generally lower than the anti-CS responses to RTS,S/AS02A in malaria-naïve adults [11], [14], [15] and in children [21], [23], [24]. In this study, the RTS,S/AS01B vaccine demonstrated significantly higher anti-CS antibody responses compared to RTS,S/AS02A confirming observations from a previous study conducted in malaria-naïve adults at the WRAIR [30]. Although GMTs for anti-CS antibodies had decreased at 6 and 10 months after dose 3 of both candidate vaccines, they remained significantly higher than the control group.

The study population had substantial baseline seroprotective anti-HBs levels in the RTS,S/AS01B and RTS,S/AS02A groups (40% and 49% respectively). Seroprotective levels of anti-HBs antibodies were readily attained with both candidate vaccines. RTS,S/AS01B and RTS,S/AS02A resulted in equivalent anti-HBs responses over a 12 month surveillance period. Although a waning of GMTs against HBs was observed by 10 months post Dose 3, group seroprotection rates were at least 90% for both candidate malaria vaccines.

This study was underpowered to measure the vaccine efficacy of the two candidate vaccine formulations compared to control based on the observed attack rate of 50% (predicted rate was 72%). In this study, vaccine efficacy in the RTS,S/AS01B group was 30% (95% CI: −15 to 57, p = 0.164), in the RTS,S/AS02A group 32% (95% CI: −12 to 58, p = 0.128) and pooling the two vaccines was 31% (95% CI: −4.7 to 54, p = 0.081). The finding that the point estimate of vaccine efficacy fell when adjusted for village of residence from 30% to 11% is difficult to interpret. It was unexplained by the known characteristics of the villages or observed malaria transmission patterns. However confidence intervals (−50 to 47) were wide and do not support the hypothesis that vaccine efficacy adjusted for village was different from the unadjusted.

The efficacy results for the RTS,S/AS02A vaccine are consistent with those obtained in naturally infected adults from The Gambia in which vaccine efficacy against malaria infection over a 15 week surveillance period was shown to be 34% (95% CI: 8.0 to 53, p = 0.014) [18]. Also, in a Phase II experimental challenge model in which RTS,S/AS01B was compared to RTS,S/AS02A in malaria-naïve adults, efficacy against infection was higher in the RTS,S/AS01B group (50% [95% CI: 32.9 to 67.1]) than the RTS,S/AS02A group (32% [95% CI: 17.6 to 47.6]) [30].

In this trial in adults, as in the experimental challenge studies [11], [14], [15], [33] and field studies [18] in adults with the candidate RTS,S/AS02A vaccine, the degree of vaccine induced protection was associated with the antibody response to circumsporozoite protein. Subjects who were not infected with malaria during the course of the study exhibited significantly higher anti-CS antibody responses than subjects who became infected. Similarly, in field studies a significant association of antibody response and vaccine induced protection against infection was found in infants [26], but not against disease in young children [21]. The efficacy results of RTS,S/AS01B in this current trial and in the challenge model in malaria-naïve adults [30] together with the significantly improved immunogenicity of RTS,S/AS01B compared to RTS,S/AS02A in both malaria-naïve adults and in semi-immune adults in this trial strongly support the continued evaluation of RTS,S/AS01B in children.

### Generalisability

The safety and tolerability of RTS,S/AS02A shown in this study are consistent with studies in young African children. This is however the first study of RTS,S/AS01B in Africa; it demonstrated favorable safety and reactogenicity and improved humoral immunogenicity when compared to the RTS,S/AS02A vaccine. Estimates of efficacy associated with RTS,S/AS01B were similar to those of RTS,S/AS02A. It is therefore planned to conduct trials of a pediatric formulation of the RTS,S/AS01 candidate vaccine (RTS,S/AS01E) in children in Africa.

### Overall evidence

This was a successful evaluation of the safety profiles of RTS,S/AS01B and RTS,S/AS02A and supports the decision to progress to the evaluation of a pediatric formulation of RTS,S/AS01 (RTS,S/AS01E) in children.

## Supporting Information

Protocol S1 Trial Protocol
(1.11 MB PDF)

Checklist S1 Consort Checklist
(0.06 MB DOC)
