# Supplementary material for: Evaluation of RTS,S/AS02A and RTS,S/AS01B in Adults in a High Malaria Transmission Area
Source: PLoS One. 2009 Jul 31;4(7):e6465. doi: 10.1371/journal.pone.0006465 (PMC2714466; doi:10.1371/journal.pone.0006465)
Supplement: Protocol S1 — Trial Protocol (1.11 MB PDF) [file pone.0006465.s001.pdf]

GlaxoSmithKline Biologicals
Rue de l'Institut 89, 1330 Rixensart,
Belgium.

**Study vaccines**	GlaxoSmithKline Biologicals' candidate *Plasmodium falciparum* malaria vaccine RTS,S/AS02A,<br>GlaxoSmithKline Biologicals' candidate *Plasmodium falciparum* malaria vaccine RTS,S/AS01B and<br>Chiron's Rabies vaccine, Rabipur®
**eTrack study number**	104743
**eTrack abbreviated title**	Malaria-044
**Investigational New Drug (IND) number**	BB-IND 11220
**Date of approval**	Final, 30 March 2005
**Amendment 1**	Final, 24 June 2005
**Amendment 2**	Final, 26 July 2005
Amendment 3	***Final, 13 June 2006***
**Title**	A Phase IIb randomized, double-blind, controlled study of the safety, immunogenicity and proof-of-concept of RTS,S/AS02A, and RTS,S/AS01B, two candidate malaria vaccines in malaria-experienced adults living in Western Kenya.

GlaxoSmithKline Biologicals

Co-ordinating author Conor Cahill, Scientific Writer.

Contributing authors

- W Ripley Ballou, Vice President – New Product Development, Early Development
- Ozzie Berger, US Regulatory Affairs
- Joe Cohen, Vice President – Research & Development – Vaccines For Emerging Diseases Program
- Sabine Corachan, Central Study Coordinator
- Marie Claude Dubois, Project Manager – Disease Area Programme Vaccines for Emerging Diseases
- Marguerite Koutsoukos, Scientist Clinical Immunology Manager, Disease Area Programme Vaccines for Emerging Diseases
- Amanda Leach, Clinical Development Manager
- Marc Lievens, Statistician
- Phillipe Moris, Scientist – TP Human Cellular Immunology
- Isabelle Ramboer, Central Study Coordinator
- Joelle Thonnard, Manager, Post Marketing Surveillance
- Marie Chantal Uwamwezi, Scientist – Regulatory Development Unit III

**eTrack study number**	104743
**eTrack abbreviated title**	Malaria-044
**IND number**	BB-IND 11220
**Date of approval**	Final, 30 March 2005
**Amendment 1**	Final, 24 June 2005
**Amendment 2**	Final, 26 July 2005
Amendment 3	***Final, 13 June 2006***
**Title**	A Phase IIb randomized, double-blind, controlled study of the safety, immunogenicity and proof-of-concept of RTS,S/AS02A, and RTS,S/AS01B, two candidate malaria vaccines in malaria-experienced adults living in Western Kenya.

Walter Reed Army Institute of Research (WRAIR)

- James F. Cummings, Department of Immunology, WRAIR
- D Gray Heppner, Chief, Department of Immunology, WRAIR
- Kent Kester, Director, Division of Regulated Activities, WRAIR
- Urszula Krzych, Department of Immunology, WRAIR
- Christian Ockenhouse, Department of Immunology, WRAIR
- Ann Stewart, Department of Immunology, WRAIR

US Army Medical Research Unit – Kenya (USAMRU-K)

- Steve Ntoburi, Clinical Investigator, USAMRU-K
- Bernards Ogutu, Clinical Investigator, USAMRU-K
- Lucas Otieno, Clinical Investigator, USAMRU-K
- Mark Polhemus, Chief of Clinical Trials, USAMRU-K
- Shon Remich, Clinical Investigator, USAMRU-K
- John Waitumbi, Laboratory Director, USAMRU-K
- Mark Withers, Kisumu Director, USAMRU-K

**eTrack study number**	104743
**eTrack abbreviated title**	Malaria-044
**IND number**	BB-IND 11220
**Date of approval**	Final, 30 March 2005
**Amendment 1**	Final, 24 June 2005
**Amendment 2**	Final, 26 July 2005
Amendment 3	***Final, 13 June 2006***
**Title**	A Phase IIb randomized, double-blind, controlled study of the safety, immunogenicity and proof-of-concept of RTS,S/AS02A, and RTS,S/AS01B, two candidate malaria vaccines in malaria-experienced adults living in Western Kenya.

London School of Hygiene and Tropical Medicine

- ***Brian Greenwood***
- ***Colin Sutherland***

Kenya Medical Research Institute

- ***Sammy Anyona***

Amended 13 June 2006

This product development plan is conducted under CRADA between WRAIR and GSK Biologicals.

U.S. Army Medical Materiel Development Activity (USAMMDA) will act as sponsor for this trial

**eTrack study number**	104743
**eTrack abbreviated title**	Malaria-044
**IND number**	BB-IND 11220
**Date of approval**	Final, 30 March 2005
**Amendment 1**	Final, 24 June 2005
**Amendment 2**	Final, 26 July 2005
Amendment 3	***Final, 13 June 2006***
**Title**	A Phase IIb randomized, double-blind, controlled study of the safety, immunogenicity and proof-of-concept of RTS,S/AS02A, and RTS,S/AS01B, two candidate malaria vaccines in malaria-experienced adults living in Western Kenya.
**Funding provided by**	• U.S. Army Medical Materiel Development Activity (USAMMDA)<br>• GlaxoSmithKline (GSK) Biologicals<br>• Walter Reed Army Institute of Research (WRAIR)

Sponsor Information

**eTrack study number**	104743
**eTrack abbreviated title**	Malaria-044
**IND number**	BB-IND 11220

1. Principal Investigator

Mark E. Polhemus
Tel: 254 733 616 550
email: mpolhemus@wrp-ksm.org

2. Medical Monitor

Amanda Leach,
Clinical Development Manager,
GlaxoSmithKline Biologicals,
Rue de l'Institut 89, 1330 Rixensart,
Belgium.
Tel: +32.2.656.77.88
Fax: +32.2.656.80.44
email: amanda.leach@gskbio.com

3. Central Study Coordinator

Isabelle Ramboer,
Central Study Coordinator,
GlaxoSmithKline Biologicals,
Rue de l'Institut 89, 1330 Rixensart,
Belgium.
Tel: +32.2.656.68.20
Fax: +32.2.656.80.44
email: isabelle.ramboer@gskbio.com

**eTrack study number**	104743
**eTrack abbreviated title**	Malaria-044
**IND number**	BB-IND 11220

4. Study Contacts for Reporting of a Serious Adverse Event

Central Study Coordinator

Isabelle Ramboer,
Central Study Coordinator,
GlaxoSmithKline Biologicals,
Rue de l'Institut 89, 1330 Rixensart,
Belgium.
Tel: +32.2.656.68.20
Fax: +32.2.656.80.44
email: isabelle.ramboer@gskbio.com

Human Subjects Research Review Board (HSRRB)

US Army Medical Research and Materiel
Command's Human Subjects Research
Review Board,
USA.
Tel: +001.310.619.21.65
Fax: +001.319.619.78.03
email: hsrrb@amedd.army.mil

USAMRMC Deputy for Reporting Serious Adverse Events

US Army Medical Research and Materiel
Command,
ATTN: MCMR-ZB P,
504 Scott Street,
Fort Detrick, MD 2170 2-5012,
USA.
Tel: +001.301.619.2165/6
Fax: +001.301.619.7803
email: hsrrb@amedd.army.mil

WRAIR Office of Research Management

Walter Reed Army Institute of Research
Office of Research Management
503 Robert Grant Avenue
Silver Spring, MD 20910-7500
USA

Tel: +001.301.319.9940
Fax: +001.301.319.9961
email: jody.ference@na.amedd.army.mil

Kenya National Ethical Review Committee

The Chairman,
Kenya National Ethical Review Committee,
c/o Kenya Medical Research Institute,
PO Box 54840, Nairobi, Kenya.
Tel: +254.20.272.25.41
email: mwasunna@nairobi.mimcom.net

Back-up Study Contact at GSK Biologicals for Reporting Serious Adverse Events

Manager Clinical Safety Vaccines

GSK Biologicals Clinical Safety Physician,
GlaxoSmithKline Biologicals,
Rue de l'Institut 89, 1330 Rixensart,
Belgium.
Tel: +32.2.656.87.98
Fax: +32.2.656.80.09
email: rix.ct-safety-vac-@gskbio.com
Mobile phone for 7/7 day availability: +32.477.40.47.13

5. Study Contact for Emergency Code Break

GSK Biologicals Clinical Safety Physician,
GlaxoSmithKline Biologicals,
Rue de l'Institut 89, 1330 Rixensart,
Belgium.
Tel: +32.2.656.87.98
Fax: +32.2.656.80.09
Mobile phone for 7/7 day availability: +32.477.40.47.13

6. Study Center

Kombewa Clinic, USAMRU-K, Kisumu Kenya

Investigator Agreement

**eTrack study number**	104743
**eTrack abbreviated title**	Malaria-044
**IND number**	BB-IND 11220
**Title**	A Phase IIb randomized, double-blind, controlled study of the safety, immunogenicity and proof-of-concept of RTS,S/AS02A, and RTS,S/AS01B, two candidate malaria vaccines in malaria-experienced adults living in Western Kenya.

I agree:

- To assume responsibility for the proper conduct of the study at this site.
- To conduct the study in compliance with this protocol, any mutually agreed future protocol amendments, and with any other study conduct procedures provided by GlaxoSmithKline Biologicals (GSK Biologicals).
- Not to implement any changes to the protocol without agreement GSK Biologicals and prior review and written approval from the Institutional Review Board (IRB) or Independent Ethics Committee (IEC), except where necessary to eliminate an immediate hazard to the subjects, or for administrative aspects of the study (where permitted by all applicable regulatory requirements).
- That I am thoroughly familiar with the appropriate use of the vaccines, as described in this protocol, and any other information provided by the sponsor (USAMMDA) and GSK Biologicals, including, but not limited to, the following: the current Investigator’s Brochure (IB) or equivalent document, IB supplement (if applicable), prescribing information (in the case of a marketed vaccine) and/or Master Data Sheet (if the Master Data Sheet exists and serves as reference document for the vaccine in the case of a marketed vaccine).
- That I am aware of, and will comply with, “Good Clinical Practices” (GCP) and all applicable regulatory requirements.
- That I have been informed that certain regulatory authorities require the sponsor (USAMMDA) to obtain and supply, as necessary, details about the investigator’s ownership interest in the sponsor or the investigational product, and more generally about his/her financial ties with the sponsor. GSK Biologicals will use and disclose the information solely for the purpose of complying with regulatory requirements.

Hence I:

- Agree to supply GSK Biologicals with any necessary information regarding ownership interest and financial ties (including those of my spouse and dependent children).
- Agree to promptly update this information if any relevant changes occur during the course of the study and for 1 year following completion of the study.

- Agree that GSK Biologicals may disclose any information it has about such ownership interests and financial ties to regulatory authorities.
- Agree to provide GSK Biologicals with an updated Curriculum Vitae and other FDA required documents.

Synopsis

Title

A Phase IIb randomized, double-blind, controlled study of the safety, immunogenicity and proof-of-concept of RTS,S/AS02A, and RTS,S/AS01B, two candidate malaria vaccines in malaria-experienced adults living in Western Kenya.

Study Population

Healthy male and female adults aged 18 to 35 years at enrollment. Those determined to be eligible, based on the inclusion and exclusion criteria will be enrolled in the study.

Rationale

GSK Biologicals and the Walter Reed Army Institute of Research (WRAIR) are developing a candidate antigen against malaria caused by *Plasmodium falciparum*, RTS,S. The antigen consists of sequences of the circumsporozoite (CS) protein and hepatitis B surface antigen (HBsAg).

When adjuvanted with AS02, a proprietary adjuvant made up of oil-in-water emulsion, MPL® and QS21, RTS,S has been found to be highly immunogenic. In human challenge studies conducted at WRAIR, the RTS,S/AS02 candidate vaccine prevented or delayed infection.

Vaccines containing the RTS,S antigen are being developed by GSK Biologicals primarily for the prevention of disease in children in malaria-endemic countries, and therefore most of the data referring to the safety, immunogenicity and reactogenicity has, to date, been collected in the pediatric population in Africa. In a recent study in which 3 doses of RTS,S/AS02A (0.25 mL dose) were administered to approximately 1000 1 to 4 year old children in Mozambique, 30% Vaccine Efficacy (VE) was demonstrated against clinical malaria disease.

As a potential improvement to RTS,S/AS02A, another candidate vaccine, RTS,S/AS01B, is being developed in parallel. The AS01B adjuvant contains the same quantities of MPL® and QS21 as in AS02A, but is formulated with liposomes rather than oil-in-water emulsion. Recent preliminary results of a human challenge study conducted at WRAIR (Malaria-027) have been encouraging, indicating that RTS,S/AS01B may be more efficacious than RTS,S/AS02A. As a next step, this study proposes to evaluate RTS,S/AS01B and RTS,S/AS02A in adults in a malaria-endemic region under conditions of natural challenge.

The proposed study will also use molecular genetic typing methods to determine two important parasitological parameters: a) if there is detectable strain-specificity in the effect of the vaccine, and b) if vaccination alters the multiplicity of subsequent infections. **Amended 13 June 2006**

A Local Safety Monitor and Safety Monitoring Group will provide safety oversight and guidance for this trial.

This product development plan is conducted under a CRADA between WRAIR and GSK Biologicals.

Objectives **Primary: Safety & Reactogenicity**

- To compare the safety and reactogenicity of RTS,S/AS01B vs RTS,S/AS02A, when administered as 3 doses intramuscularly on a 0, 1 and 2 months schedule to adult volunteers living in a malaria endemic region

Secondary: Safety

- To describe the safety and reactogenicity of RTS,S/AS02A and RTS,S/AS01B, when administered as 3 doses intramuscularly on a 0, 1 and 2 months schedule to adult volunteers living in a malaria endemic region.

Secondary: Immunogenicity

- To describe antibody responses to the circumsporozoite (anti-CS) antigen and hepatitis B surface antigen (anti-HBs) of RTS,S/AS02A and RTS,S/AS01B when administered as 3 doses intramuscularly on a 0, 1 and 2 months schedule to adult volunteers living in a malaria endemic region.

Secondary: Proof-of-concept

- To assess the efficacy of RTS,S/AS02A and RTS,S/AS01B against infection with *P. falciparum* malaria (defined as *P. falciparum* asexual parasitemia > 0) over a period of 14 weeks post Dose 3, in adults under conditions of natural transmission.

Tertiary: Safety

- To describe the long-term safety of RTS,S/AS02A and RTS,S/AS01B until 12 months post Dose 1.

Exploratory: Parasite Genotyping

- ***To determine the prevalence of vaccine-like alleles of CSP in the study population.***
- ***To investigate whether vaccination with RTS,S/AS0A2 and RTS,S/AS01B modifies the distribution of amino acid substitutions in the Th2R and Th3R sequence regions of the CSP gene in subsequent P. falciparum infections.***

- ***To investigate whether vaccination with RTS,S/AS0A2 and RTS,S/AS01B modifies the number of P. falciparum genotypes (multiplicity of infection) as determined by analysis of msp-1 and msp-2 genes.***

Amended 13 June 2006

Study design

- Phase IIb, single-center, double blind (observer blind, participant blind), randomized controlled trial with three groups in one study site (randomization 1:1:1, RTS,S/AS01B: RTS,S/AS02A:rabies vaccine).

Vaccines	Schedule	Number of subjects to be enrolled	Estimated % evaluable
RTS,S/AS02A	0,1,2 months	85	90%
RTS,S/AS01B	0,1,2 months	85	90%
Rabies vaccine	0,1,2 months	85	90%

- 255 adults will be enrolled. It is assumed that 90% will complete study procedures and be evaluable.
- Prior to study start, a community information program will inform the local population of the study. Throughout the period of enrollment, study information will be presented at community centers.
- Healthy male and female adults aged 18 to 35 years will be screened. Those determined to be eligible, based on the inclusion and exclusion criteria, will be enrolled in the study.
- Route of administration: all vaccines will be administered by the intramuscular route to the deltoid of the non-dominant arm
- Each subject will be observed for at least 30 minutes after vaccination to evaluate and treat any acute adverse events
- After vaccination, the subjects will visit field workers at field stations for the detection of adverse events (AEs) occurring after vaccination for one week (days 1, 2, 3, 4, 5 and day 6 after each vaccination). Diary cards and thermometers will be provided for the field workers to record oral temperature and any local (at the injection site) or general adverse events.
- One week prior to Dose 3, subjects will be treated with Malarone® for presumptive clearance of malaria parasitemia (in the event that subjects are unable to tolerate Malarone®, Coartem® (artemether/lumefantrine, Novartis AG, Zurich, Switzerland) will be used as the alternative clearance treatment.
- Surveillance for Active Detection of Infection (ADI) will begin 2 weeks after Dose 3. Subjects will visit field workers at field-stations near their homes weekly during the ADI period. The total period of surveillance for ADI is 14 weeks. Two Clinic Visits and 14 field-

worker visits will contribute to the ADI.

- All subjects will have a blood slide prepared and read to check for asexual *P. falciparum* parasitemia one week post Dose 3. Any subject who tests positive will be treated with Coartem®
- At each contact for ADI, history of fever will be recorded and oral temperature taken; a smear will be taken for detection of malaria parasites.
- Infection with malaria parasites is defined as *P. falciparum* asexual parasitemia > 0
- Subjects who are symptomatic at the time of ADI contact (i.e. history of fever within previous 24 hours or oral temperature >37.5°C) will have a blood slide read and treated within the same day if the blood slide is positive.
- Subjects will be followed passively for the occurrence of clinical malaria from Day 0 until 16 weeks post Dose 3. Cases will be detected at Kombewa Clinic. If a subject feels ill they can present to a field station near their home and be transported to Kombewa Clinic for evaluation.
- ***DNA will be extracted from EDTA blood of all subjects with patent asexual parasitemia.*** **Amended 13 June 2006**
- There will be a 30 day follow-up period after each dose of vaccine for reporting unsolicited symptoms
- Recording of serious adverse events will be throughout the study period. They will be captured through the morbidity surveillance system at Kombewa Clinic.
- Anti-CS antibody titers will be determined at baseline, one month post Dose 2, 1 month post Dose 2, 1 month post Dose 3, 4 months post Dose 3 and 10 months post Dose 3.
- Anti-HBs antibody titers will be determined at baseline, one month post Dose 3 and 10 months post Dose 3.
- CS-specific CMI will be assessed at ~~***three***~~ ***four*** time points during the study; prevaccination, 1 month post Dose 3, ~~***and***~~ 4 months post Dose 3 ***and 9 months post Dose 3***. **Amended 13 June 2006**
- Data collection will be by conventional CRF.
- Duration of the study will be approximately 12 months per subject.
- There will be a planned interim analysis on all safety humoral and immunogenicity data collected up to 30 days after Dose 3.
- The final analysis of the study for all primary and secondary endpoints will take place on all data collected up to the end of the

surveillance for ADI (16 weeks post Dose 3).

- All subjects will be followed for safety for a period that extends to 10 months post Dose 3. Any data collected after the final analysis will be reported in an annex report.

Sample size

- **Primary Objective:** The primary objective will be assessed by comparing the proportion of subjects experiencing a Grade 3 solicited or unsolicited general reaction after each vaccination during a seven day follow-up period (day of vaccination and six subsequent days). In a previous study with RTS,S/AS02A in Africa (Malaria-008) the proportion affected was 10%. The proportion affected was higher in a recent study conducted in non-immune adults at WRAIR (Malaria-027); 39%. Therefore the table below has been generated for a wide range of hypothetical proportions of Grade 3 general symptoms. The table shows the potential of the study to detect a difference in the proportion of subjects affected by Grade 3 general symptoms assuming 90% power and a sample size of 255 at a significance level of $p < 0.05$.

Frequency of events for RTS,S/AS02A	Frequency of events for RTS,S/AS01B	Power to detect difference (75 per group) ($p < 0.05$)
5%	24%	90%
10%	33%	90%
20%	46%	90%
30%	57%	90%
40%	67%	90%
50%	77%	90%

- **Secondary Objective; Safety:** The data set will be examined, comparing the rates of SAEs at the Medical Dictionary for Regulatory Activities (MedDRA) preferred term level. SAEs are expected to occur infrequently and the study has therefore the power to detect only large differences in the frequencies of SAEs compared to control. See table below:

Frequency of events for Rabies vaccine	Frequency of events for RTS,S/AS02A or RTS,S/AS01B	Power to detect difference (75 per group) ($p < 0.05$)
1%	13%	80%
3%	17%	80%
5%	21%	80%
10%	29%	80%

- **Secondary Objective; Proof-of-Concept:** The minimum vaccine efficacy that a study of this size has the power to detect (i.e. vaccine vs. control) is shown in the table below. This assumes an attack rate of 72%.

VE in malaria vaccine groups	Power ($p < 0.05$)
41%	80%
45%	90%

The table below shows the increase in Vaccine Efficacy of RTS,S/AS01B over RTS,S/AS02A that can be detected with 80% power. Historically, RTS,S/AS02A has demonstrated efficacy against infection in African adults in The Gambia of 34% (Malaria-005) and in children in Mozambique of 45% (Malaria-026).

VE in RTS,S/AS02A vs control	VE in RTS,S/AS01B vs control	Power ($p < 0.05$)
30%	65%	80%
40%	71%	80%
50%	78%	80%

Endpoints

Primary endpoint: Safety & Reactogenicity

- Occurrence of Grade 3 solicited or unsolicited general reactions after each vaccination during a seven day follow-up period (day of vaccination and 6 subsequent days)

Secondary endpoints: Safety

- Occurrence of SAEs until 4 months post Dose 3.
- Occurrence of unsolicited AEs after Dose 1, 2 and 3 of vaccine over a 30 day follow-up period (day of vaccination and 29 subsequent days)
- Occurrence of solicited general and local reactions over a 7 day follow-up period (day of vaccination and 6 subsequent days) after Dose 1, 2 and 3 of vaccine.
- Occurrence of parameters of hematological monitoring below normal range.
- Occurrence of parameters of biochemical monitoring above normal range

Secondary endpoints: Immunogenicity

Endpoints assessed prior to vaccination, and 1 month post Dose 3:

- Anti HBs antibody titers

Endpoints assessed prior to vaccination,, 1 month post Dose 2, 1 month post Dose 3 and 4 months post Dose 3:

- Anti-CS antibody titers

Secondary endpoints: Efficacy

- The time to first malaria infection (first recording of infection of asexual stage falciparum parasites detected by the active case detection or passive case detection) over a period starting 14 days after Dose 3 and extending for 14 weeks.
- The asexual *P. falciparum* parasitemia (prevalence and density) at 16 weeks post Dose 3

Tertiary endpoints: Safety

- The occurrence of SAEs from 4 months post Dose 3 until study conclusion

Tertiary endpoints: Immunogenicity

Endpoints assessed 10 months post Dose 3:

- Anti HBs antibody titers
- Anti-CS antibody titers

Exploratory endpoint: Cell Mediated Immunity

Endpoint assessed prior to vaccination, 1 month post Dose 3, ~~***and***~~ 4 months post Dose 3 ***and 9 months post Dose 3***. **Amended 13 June 2006**

- For each subject, the frequency of CS-specific CD4+ and CD8+ T-cells, as measured by intracellular cytokine staining.

Exploratory endpoint: Efficacy

- The percentage change in hemoglobin value between baseline and 16 weeks post Dose 3.

Exploratory endpoints: Parasite Genotyping

- ***Sequencing of the Th2R and Th3R epitope regions in parasite-positive individuals identified by CSP PCR amplification of Day 0 DNA samples.***
- ***DNA samples prepared at first microscopy confirmed infections identified after the third vaccination, used to determine the distribution of amino acid substitutions in the Th2R and Th3R sequence regions of the CSP gene.***
- ***DNA samples prepared at first microscopy confirmed infection identified after the third vaccination, used to determine the number of P. falciparum genotypes (multiplicity of infection) as determined by analysis of msp-1 and msp-2 genes.***

Amended 13 June 2006

TABLE OF CONTENTS

PAGE

SYNOPSIS **10**

GLOSSARY OF TERMS **27**

1. INTRODUCTION **30**
1.1. Malaria 30
1.2. Hepatitis B 31
1.3. Rationale for vaccine development 31
1.3.1. AS02A Adjuvant 32
1.3.2. AS01B Adjuvant 33
1.3.3. The RTS,S/AS02A candidate malaria vaccine; data collected to date 34
1.3.3.1. Trials of RTS,S/AS02A in adults 34
1.3.3.1.1. Safety experience of RTS,S/AS02A in malaria-experienced adults in Africa 34
1.3.3.1.2. Efficacy of RTS,S/AS02A in adults 35
1.3.3.1.3. Safety and immunogenicity of alternative vaccination schedules in adults 36
1.3.3.1.4. Studies of RTS,S/AS02A in adults; conclusions 36
1.3.3.2. Trials of RTS,S/AS02A in children 40
1.3.3.2.1. Phase I pediatric trials of RTS,S/AS02A (Malaria-015, Malaria-020 and Malaria-025) 40
1.3.3.2.2. Phase IIb pediatric trial of RTS,S/AS02A (Malaria-026) 46
1.3.3.2.3. Phase II pediatric trial of RTS,S/AS02D; Bridging Study (Malaria-034) 55
1.3.3.2.4. Studies of RTS,S/AS02A in children; conclusions 58
1.3.4. The RTS,S/AS01B candidate malaria and hepatitis B vaccine; data collected to date 58
1.3.4.1. Trials of RTS,S/AS01B in adults 58
1.3.5. Concurrent protection against hepatitis B 62
1.4. Rationale for the study design 62
1.4.1. Safety monitoring plan 63
1.4.2. Assessment of proof-of-concept 63
1.4.3. Rationale for the use of Rabies vaccine as a control 63
1.5. Rationale for testing cell-mediated immunity 64
1.6. Rationale for Parasite Genotyping evaluation 64
1.7. HIV testing at screening 65
1.8. Risks and benefits for study volunteers 65
1.8.1. Risks of receipt of RTS,S/AS02A, RTS,S/AS01B or Rabies Vaccine and risk-management 65

1.8.2. Risk of receipt of Malarone and risk management........................ 66
1.8.3. Risk of receipt of Coartem and risk management......................... 66
1.8.4. Risk of disclosure of volunteer's confidential/personal information to persons not involved in the study.......................... 66
1.8.5. Benefits to study volunteers of taking part in the study................ 66

2. OBJECTIVES 67
2.1. Primary Objective.. 67
2.1.1. Safety & Reactogenicity.. 67
2.2. Secondary Objectives 67
2.2.1. Safety... 67
2.2.2. Immunogenicity.. 67
2.2.3. Proof-of-concept 67
2.3. Tertiary Objective 68
2.3.1. Safety... 68
2.4. Exploratory Objectives 68
2.4.1. Parasite Genotyping 68

3. STUDY DESIGN OVERVIEW... 68

4. STUDY COHORT & STUDY SITE.. 70
4.1. Study Site... 70
4.1.1. Kenya Medical Research Institute and Walter Reed Project Kombewa Clinic, Western Kenya 70
4.1.2. Study site; geography 71
4.1.3. Study site; malaria transmission 71
4.1.4. Study site; population... 72
4.2. Number of subjects 72
4.3. Inclusion criteria 73
4.4. Exclusion criteria for enrollment 73
4.5. Elimination criteria during the study 74
4.6. Contraindications to subsequent vaccination.............................. 75
4.6.1. Absolute contraindications to further vaccination......................... 75
4.6.2. Indications for deferral of vaccination 75

5. CONDUCT OF STUDY.. 76
5.1. Ethics and regulatory considerations 76
5.1.1. Institutional Review Board/Independent Ethics Committee (IRB/IEC).. 76
5.1.2. Informed consent 77
5.1.3. Safety monitoring plan 79
5.1.3.1. Local Safety Monitor (LSM).. 80
5.1.3.2. Safety Monitoring Group (SMG)................................. 80
5.1.3.3. Safety Reports.. 81
5.1.3.4. Process for the suspension of vaccination.................. 81
5.1.3.4.1. Indications for immediate suspension of all vaccination 81
5.1.3.4.2. Indications for suspension of progression to next sequential dose 82
5.1.3.4.3. Process if the trial is suspended 82
5.1.4. Exposure to rabies.. 82

5.1.5. Cross over immunization with rabies vaccine 82
5.2. Recruitment/Screening 82
5.2.1. Community information 82
5.2.2. Recruitment 83
5.2.3. Screening of volunteers 83
5.3. Vaccination process 84
5.4. Home follow-up visits for assessment of reactogenicity (7-day follow-up period) 85
5.5. Monitoring of hematological and biochemical laboratory parameters 86
5.6. Surveillance for SAEs (all subjects) 86
5.7. Study site; health care provision in the study area 87
5.7.1. Outpatient Care at Kombewa Clinic 87
5.7.2. New Nyanza Provincial General Hospital 87
5.7.3. Referring of Excluded Volunteers to Medical Care 87
5.8. Management and treatment of malaria in all subjects 88
5.9. Passive case detection of clinical cases of malaria 88
5.10. Active detection of infection 89
5.11. Subject identification 90
5.12. Outline of study procedures 91
5.13. Detailed description of study visits 92
5.14. Sample handling and analysis 101
5.14.1. Treatment and storage of biological samples 101
5.14.2. Laboratory assays 102

6. INVESTIGATIONAL PRODUCTS AND ADMINISTRATION 103
6.1. Investigational products 103
6.1.1. RTS,S/AS02A candidate malaria and hepatitis B vaccine 104
6.1.2. RTS,S/AS01B candidate malaria and hepatitis B vaccine 104
6.1.3. Rabipur Rabies Vaccine 104
6.2. Dosage and administration 105
6.2.1. RTS,S/AS02A (0.5 mL dose) 105
6.2.2. RTS,S/AS01B (0.5 mL dose) 105
6.2.3. Rabipur rabies vaccine (1.0 mL dose) 105
6.3. Storage 105
6.4. Treatment allocation and randomization 106
6.4.1. Randomization of supplies 106
6.4.2. Randomization of subjects 106
6.5. Method of blinding and breaking the study blind 106
6.6. Replacement of unusable vaccine doses 107
6.7. Packaging 107
6.8. Vaccine accountability 107
6.9. Concomitant medication/treatment 107

7. HEALTH ECONOMICS 108

8. ADVERSE EVENTS AND SERIOUS ADVERSE EVENTS 108
8.1. Definition of an adverse event 108
8.2. Definition of a serious adverse event 109
8.3. Definition of an Unanticipated Problem 110
8.4. Clinical laboratory parameters and other abnormal assessments qualifying as adverse events and serious adverse events 111

8.5. Time period, frequency, and method of detecting adverse events and serious adverse events ... 111
8.5.1. Solicited adverse events ... 112
8.6. Evaluating adverse events and serious adverse events ... 113
8.6.1. Assessment of intensity ... 113
8.6.2. Assessment of causality ... 115
8.7. Follow-up of adverse events and serious adverse events and assessment of outcome ... 116
8.8. Prompt reporting of serious adverse events and unanticipated problems to the study contacts for reporting of a serious adverse event ... 117
8.8.1. Time frames for submitting serious adverse event reports to GSK Biologicals and USAMMDA ... 117
8.8.2. Completion and transmission of serious adverse event reports to the study contacts for reporting of a serious adverse event ... 117
8.8.3. Reporting of unanticipated problems ... 119
8.9. Regulatory reporting requirements for serious adverse events ... 119
8.10. Post study adverse events and serious adverse events ... 120
8.11. Pregnancy ... 120
8.12. Treatment of adverse events ... 121

9. SUBJECT COMPLETION AND WITHDRAWAL ... 121
9.1. Subject completion ... 121
9.2. Subject withdrawal ... 121
9.2.1. Subject withdrawal from the study ... 121
9.2.2. Subject withdrawal from investigational product ... 122

10. DATA EVALUATION: CRITERIA FOR EVALUATION OF OBJECTIVES ... 122
10.1. Primary Endpoint ... 122
10.1.1. Safety & Reactogenicity ... 122
10.2. Secondary endpoints ... 123
10.2.1. Safety ... 123
10.2.2. Immunogenicity ... 123
10.2.3. Efficacy ... 123
10.3. Tertiary endpoints ... 123
10.3.1. Safety ... 123
10.3.2. Immunogenicity ... 123
10.4. Exploratory endpoints: ... 124
10.4.1. Cell Mediated Immunity ... 124
10.4.2. Efficacy ... 124
10.4.3. Parasite Genotyping ... 124
10.5. Estimated sample size ... 124
10.5.1. Sample size for the Primary Objective ... 124
10.5.2. Sample Size for the Secondary Objective for Safety ... 125
10.5.3. Sample Size for the Secondary Objective for Proof-of-Concept ... 125
10.6. Study cohorts to be evaluated ... 126
10.6.1. Total Vaccinated cohort ... 126
10.6.2. According-To-Protocol (ATP) cohort for analysis of safety ... 126
10.6.3. According To Protocol (ATP) cohort for analysis of immunogenicity ... 126

10.6.4. According To Protocol (ATP) cohort for analysis of proof-of-concept 127
10.7. Derived and transformed data 127
10.8. Final analyses 127
10.8.1. Analysis of demographics/baseline characteristics 127
10.8.2. Analyses of safety 127
10.8.2.1. Primary objective 128
10.8.2.2. Secondary objectives 128
10.8.3. Analyses of immunogenicity 129
10.8.3.1. Anti-CS antibodies 129
10.8.3.2. Anti-HBs antibodies 129
10.8.4. Analyses of efficacy 129
10.8.4.1. Case Definitions 129
10.8.4.2. Calculation of the time at risk 129
10.8.4.3. Vaccine efficacy 130
10.8.4.3.1. Vaccine efficacy compared to Rabies control vaccine 130
10.8.4.3.2. Vaccine efficacy of RTS,S/AS02A against RTS,S/AS01B 131
10.8.4.4. Adjustment for covariates 131
10.8.4.5. Hemoglobin values 131
10.8.5. Analysis of parasite genotyping 131
10.9. Planned Interim Analysis 132
10.9.1. Analysis of demographics/baseline characteristics 132
10.9.2. Analyses of safety 132
10.9.3. Analyses of immunogenicity 133
10.9.3.1. Anti-CS antibodies 133
10.9.3.2. Anti-HBs antibodies 133

11. ADMINISTRATIVE MATTERS 133

12. REFERENCES 133

APPENDICES

Appendix A World Medical Association Declaration of Helsinki 137

Appendix B Administrative Matters 141

Appendix C Overview of the Recruitment Plan 147

Appendix D Handling of Biological Samples Collected by the Investigator 149

Appendix E Shipment of Biological Samples 153

Appendix F Laboratory Assays 154

Appendix G Vaccine supplies, packaging and accountability 158

Appendix H Description of drugs to be used in the trial for clearance of malaria parasites 161

Appendix I Roles and responsibilities of study personnel 162

Appendix J Amendments to the Protocol 163

Appendix K Amendments to the Protocol 180

Appendix L Amendments to the Protocol 182

LIST OF ABBREVIATIONS

ADI	Active Detection of Infection
AE	Adverse event
ALT	Alanine aminotransferase
anti-CS	antibody to the *P. falciparum* circumsporozoite protein (CS) repeat region
anti-HBs	antibody to the hepatitis B surface antigen
AS01B	one of GlaxoSmithKline Biologicals' proprietary adjuvant systems; contains QS21, MPL® and liposomes
AS02(A)	one of GlaxoSmithKline Biologicals' proprietary adjuvant systems; contains QS21, MPL® and proprietary oil-in-water emulsion
ATP	According to protocol
CBC	Complete blood count
CI	Confidence interval
CMI	Cell-mediated immunity
CRF	Case report form
CS	Circumsporozoite protein
CSC	Central study coordinator
CTL	Cytotoxic T-lymphocyte
D	Diphtheria
dL	Deciliter
EIA	Enzyme immunosorbent assay
EIR	Entomological inoculation rate
EISR	Expedited investigator safety report
ELISA	Enzyme linked immunosorbent assay
EPI	Expanded program on immunization
FDA	Food and Drug Administration, United States
G6PD	Glucose-6-Phosphate Dehydrogenase
GCP	Good clinical practice
GMT	Geometric mean titer
GSK	GlaxoSmithKline
HBsAg	Hepatitis B surface antigen
HBV	Hepatitis B virus

HIV	Human immunodeficiency virus
HSRRB	US Army Medical Research and Materiel Command's Human Subjects Research Review Board
IB	Investigator's brochure
ICF	Informed consent form
ICS	Intracellular staining
IEC	Independent ethics committee
IFN-γ	Interferon-gamma
IM	Intramuscular
IND	Investigational new drug
IRB	Institutional review board
IU	International unit
KEMRI	Kenya Medical Research Institute
KC	Kombewa Clinic
kg	Kilogram
LSHTM	***London School of Hygiene and Tropical Medicine***
LSM	Local Safety Monitor
MBF	Malaria blood film
MedDRA	Medical Dictionary for Regulatory Activities
mg	Milligram
mL	Milliliter
MOI	***Multiplicity of Infection***
MPL®	3-deacylated monophosphoryl lipid A
NNPGH	New Nyanza Provincial General Hospital
OPD	Out patient department
P. falciparum	*Plasmodium falciparum*
PBS	Phosphate buffered saline
PCR	Polymerase chain reaction
PFS	Pre-filled syringe
PI	Principal Investigator
PRP	The polyribosyl ribitol phosphate capsule of the *Hemophilus influenzae* bacterium
QS 21	'*Quillaja saponaria* 21': a triterpene glycoside purified from the bark of *Quillaja saponaria*

RAP	Report and Analysis Plan
RTS	Hybrid protein comprising S (hepatitis B surface antigen) and CSP portions
RTS,S	Particulate antigen, containing both RTS and S (hepatitis B surface antigen) proteins
SAE	Serious adverse event
SMG	Safety Monitoring Group
SOP	Standard operating procedure
SP	sulfadoxine-pyrimethamine
USAMRMC	United States Army Medical Research and Materiel Command
USAMRU-K	United States Army Medical Research Unit – Kenya
VE	Vaccine efficacy
WRAIR	Walter Reed Army Institute of Research
WRP	Walter Reed Project, Kisumu, Kenya

Amended 13 June 2006

Glossary of Terms

Adverse event:	Any untoward medical occurrence in a patient or clinical investigation subject, temporally associated with the use of a medicinal product, whether or not considered related to the medicinal product.<br><br>An AE can therefore be any unfavorable and unintended sign (including an abnormal laboratory finding), symptom, or disease (new or exacerbated) temporally associated with the use of a medicinal product. For marketed medicinal products, this also includes failure to produce expected benefits (i.e. lack of efficacy), abuse or misuse.
Active detection of Infection (ADI):	The detection of malaria parasitemia on a blood sample taken at a scheduled or unscheduled visit during a defined ADI phase of the study, i.e. regardless of whether a subject has signs or symptoms of infection.
Blinding:	A procedure in which one or more parties to the trial are kept unaware of the treatment assignment in order to reduce the risk of biased study outcomes. In a single-blind trial, the investigator and/or his staff are aware of the treatment assignment but the subject is not. In an observer-blind study, the subject and the study personnel involved in the clinical evaluation of the subjects are blinded while other study personnel may be aware of the treatment allocation. When the investigator and sponsor staff who are involved in the treatment or clinical evaluation of the subjects and review/analysis of data are also unaware of the treatment assignments, the study is double blind. Partially blind is to be used for study designs with different blinding levels between different groups, e.g. double blinded consistency lots which are open with respect to the control group. The level of blinding is maintained throughout the conduct of the trial, and only when the data are cleaned to an acceptable level of quality will appropriate personnel be unblinded or when required in case of a serious adverse event.
Central Study Coordinator:	An individual assigned by and centrally located at GSK Biologicals at Rixensart who is responsible for assuring proper conduct of a clinical study.
Eligible:	Qualified for enrollment into the study based upon strict adherence to inclusion/exclusion criteria.
eTrack:	GSK’s clinical trials tracking tool

Evaluable:	Meeting all eligibility criteria, complying with the procedures defined in the protocol, and, therefore, included in the according-to-protocol (ATP) analysis (see Sections 4.3, 4.4 and 10.6 for details on criteria for evaluability).
Investigational product:	A pharmaceutical form of an active ingredient or placebo being tested or used as a reference in a clinical trial, including a product with a marketing authorization when used in a way different from the approved form, or when used for an unapproved indication, or when used to gain further information about an approved use.
Local Safety Monitor (LSM):	The overall role of the Local Safety Monitor, an experienced physician based in-country, will be to support the study investigators and to act as a link between the investigators and the Safety Monitoring Group (SMG) (see Section 5.1.3.2 for further details).
Medical Monitor:	An individual medically qualified to assume the responsibilities of the GSK Biologicals especially in regards to the ethics, clinical safety of a study and the assessment of adverse events.
Protocol amendment:	ICH defines a protocol amendment as: “A written description of a change(s) to or formal clarification of a protocol”. GSK Biologicals further details this to include a change to an approved protocol that affects the safety of subjects, scope of the investigation, study design, or scientific integrity of the study.
Protocol administrative change:	A protocol administrative change addresses changes to only logistical or administrative aspects of the study.<br><br>N.B. Any change that falls under the definition of a protocol amendment (e.g. a change that affects the safety of subjects, scope of the investigation, study design, or scientific integrity of the study) MUST be prepared as an amendment to the protocol.
Randomization:	Process of random attribution of treatment to subjects in order to reduce bias of selection
SMG:	The SMG is a group of medical experts representing the institutions participating in the study, appointed to oversee safety aspects of the conduct of the study. See Section 5.1.3.2 for a full overview of the role and structure of the SMG.
Solicited adverse event:	Adverse events (AEs) to be recorded as endpoints in the clinical study. The presence/occurrence/intensity of these events is actively solicited from the subject or an observer during a specified post-vaccination follow-up period.

Study Monitor:	An individual assigned by the sponsor, USAMMDA or GSK Biologicals who is responsible for assuring proper conduct of a clinical study.
Subject:	Term used throughout the protocol to denote an individual that has been contacted in order to participate in the clinical study, either as a recipient of the investigational product(s) or as a control.
Treatment:	Term used throughout the clinical study to denote a set of investigational product(s) or marketed product(s) or placebo intended to be administered to a subject, identified by a unique number, according to the study randomization or treatment allocation.
Treatment number:	A unique number identifying a treatment to a subject, according to the study randomization or treatment allocation.
Unsolicited adverse event:	Any adverse event (AE) reported in addition to those solicited during the clinical study. Also any “solicited” symptom with onset outside the specified period of follow-up for solicited symptoms will be reported as an unsolicited adverse event.

1. INTRODUCTION

GSK Biologicals and the Walter Reed Army Institute of Research (WRAIR) are developing a candidate antigen against malaria caused by *Plasmodium falciparum*, RTS,S. The antigen consists of sequences of the circumsporozoite (CS) protein and hepatitis B surface antigen (HBsAg).

When adjuvanted with AS02, a proprietary adjuvant made up of oil-in-water emulsion, MPL® and QS21, RTS,S has been found to be highly immunogenic. In human challenge studies conducted at WRAIR, the RTS,S/AS02 candidate vaccine prevented or delayed infection.

Vaccines containing the RTS,S antigen are being developed by GSK Biologicals primarily for the prevention of disease in children in malaria-endemic countries, and therefore most of the data referring to the safety, immunogenicity and reactogenicity has, to date, been collected in the pediatric population in Africa. In a recent study in which 3 doses of RTS,S/AS02A (0.25 mL dose) were administered to approximately 1000 1 to 4 year old children in Mozambique, 30% Vaccine Efficacy (VE) was demonstrated against clinical malaria disease.

As a potential improvement to RTS,S/AS02A, another candidate vaccine, RTS,S/AS01B, is being developed in parallel. The AS01B adjuvant contains the same quantities of MPL® and QS21 as in AS02A, but is formulated with liposomes rather than oil-in-water emulsion. Recent preliminary results of a human challenge study conducted at WRAIR (Malaria-027) have been encouraging, indicating that RTS,S/AS01B may be more efficacious than RTS,S/AS02A. As a next step, this study proposes to evaluate RTS,S/AS01B and RTS,S/AS02A in adults in a malaria-endemic region under conditions of natural challenge.

1.1. Malaria

Four species of the *Plasmodium* protozoan parasite are the etiologic agents of malaria in humans (*P. falciparum*, *P. vivax*, *P. ovale* and *P. malariae*). Of these four parasites, *P. falciparum* is the major cause of severe morbidity and mortality.

There can be no doubt of the importance of *P. falciparum* malaria as a major cause of human suffering and economic drain across sub-Saharan Africa [Breman 2001a, Gallup 2001]. In this region, it causes the deaths of between 0.5 and 2.0 million children every year and is the most common reason for admission to hospital, leading each year to about 300 million clinical episodes in children under five years [Breman 2001a].

The incidence of malaria in much of Africa is increasing for a variety of reasons: changes in agricultural practices, armed conflicts, migration of refugees, increasing drug resistance to conventional anti-malarial drugs, and insecticide resistance of the anopheline mosquito vectors. It is estimated that the number of cases of clinical malaria will more than double over the next 20 years without effective control. The burden of malaria at the country level correlates closely with the rate of economic development

even after adjustment for confounding factors, indicating that malaria is an important constraint on economic progress [Breman 2001b].

1.2. Hepatitis B

The hepatitis B surface antigen (HBsAg) contained in the RTS,S candidate malaria vaccine is encoded by the hepatitis B virus S protein gene that is identical to the gene used to express HBsAg in GSK Biologicals' Engerix-B® vaccine against hepatitis B. The RTS,S containing vaccines are therefore being developed to provide protection against hepatitis B.

Hepatitis B is an infection of the liver due to hepatitis B virus (HBV); it is an important public health problem across the developing world. World-wide approximately 350 million people carry HBV and about 1 million chronic carriers die annually [Vryheid 2001]. In sub-Saharan Africa infection primarily occurs by one of three routes: perinatally from mother to infant, during childhood through close personal contact and from adolescence onwards through sexual contact. The likelihood of the infection becoming chronic is dependent upon the age at infection: 90% if infected in infancy, 30% to 50% if infected between the ages of 1 to 4 years, and low in adulthood. For those that become chronically infected during childhood the risk of death from HBV-related liver cancer or cirrhosis in adult life is approximately 25% [World Health Organization 2003].

1.3. Rationale for vaccine development

The RTS,S malaria vaccines are hypothesized to reduce frequency and severity of clinical disease episodes through their impact on the initial sporozoite and liver stage parasite burden, leading to a significant reduction of the infectious inoculum of liver stage merozoites released into the blood stream. This hypothesis is fully consistent with the data observed in clinical trials of RTS,S/AS02A to date. In the homologous sporozoite challenge model the vaccine prevents approximately 40% of individuals from becoming infected and a delay in the prepatent period of non-protected individuals is observed. [Kester, 2001]. In the field efficacy study in children (Malaria-026; refer to Section 1.3.3.2.2) the point estimate of VE against clinical disease was 30% (95% CI 11% to 45%), and higher against severe malaria; 58% (95% CI 16% to 81%). A vaccine that induces partial immunity against pre-erythrocytic stages of disease may provide protection to vulnerable young children from the severe forms of the disease, whilst continuing exposure allows them to build up natural bloodstage immunity. Acquisition of natural immunity may be important to prevent a shift of severe disease burden from younger to older age groups upon waning of vaccine-induced immunity.

In parallel to the continued development of RTS,S/AS02A and RTS,S/AS02D in children in endemic countries, GSK Biologicals and WRAIR have continued to pursue alternate strategies to improve the VE and duration of efficacy. One such strategy is the combination of the RTS,S antigen with the AS01B adjuvant. Both adjuvant formulations have a number of similar key components (refer to Section 1.3.1, Section 1.3.2 and Table 1). Also refer to Table 1 for an overview of the status of the vaccine formulations within the clinical development plan.

Table 1 Formulations of RTS,S

Formulation	RTS,S (µg)	MPL® (µg)	QS21 (µg)	Volume (mL)	Comments	
RTS,S/AS02A (0.5 mL dose)	50	50	50	0.5	oil/water emulsion	Efficacy against infection demonstrated in adults in an endemic country (Malaria-005 [a])
RTS,S/AS02A (0.25 mL dose)	25	25	25	0.25	oil/water emulsion	Efficacy against clinical disease demonstrated in children in an endemic area (Malaria-026 [b])
RTS,S/AS02D [c] (0.5 mL dose)	25	25	25	0.5	oil/water emulsion	Bridged to RTS,S/AS02A (0.25 mL dose) (Malaria-034) [d]
RTS,S/AS01B	50	50	50	0.5	liposomes	Efficacy against infection demonstrated in malaria-naïve adults (Malaria-027[e])

a GSK data on file, Malaria-005 Clinical Study Protocol, 1997
b GSK data on file, Malaria-026 Clinical Study Protocol, 2003
c AS02D is composed of the same active constituents in the same quantities as in a 0.25 mL dose of RTS,S/AS02A, but is formulated to supply a 0.5 mL dose
d GSK data on file, Malaria-034 Clinical Study Protocol, 2003
e Study not yet reported (January 2005)

1.3.1. AS02A Adjuvant

In RTS,S/AS02A, the vaccine antigen, RTS,S is formulated in the GSK proprietary adjuvant system 2 (AS02); containing a proprietary oil-in-water emulsion and the immunostimulants QS21 (a triterpene glycoside purified from the bark of *Quillaja saponaria*) and MPL®. The choice of AS02 for evaluation as a malaria pre-erythrocytic vaccine was based on extensive pre-clinical experimentation conducted by GSK Biologicals and collaborators. Pre-clinical immunogenicity studies in mice and in rhesus monkeys demonstrated conclusively that this formulation is a powerful inducer of both humoral and CMI responses, including IFN-γ-secreting T-helper 1 lymphocytes [GSK data on file, Malaria Investigator's Brochure 2003].

A confirmation of the critical importance of adequate formulation and of the value of AS02 as an adjuvant system for pre-erythrocytic malaria vaccine was obtained in a clinical trial conducted in collaboration with the Walter Reed Army Institute of Research (WRAIR), WRMAL-003 [Stoute 1997]. In this Phase I/IIa trial, a vaccine consisting of the RTS,S antigen formulated in AS02 protected six out of seven volunteers (86% vaccine efficacy; (95% CI 0.02 to 0.88) from infection following a sporozoite challenge delivered by the bites of laboratory-reared infectious *Anopheles* mosquitoes. In this trial, two other vaccine formulations (adjuvanted with AS04 and AS03) of the same RTS,S antigen were also evaluated but afforded only marginal protection against challenge.

The AS02 formulation to be used in malaria vaccine trials no longer contains any thiomersal as preservative and is supplied as monodose pre-filled syringes.

1.3.2. AS01B Adjuvant

The pre-clinical evaluation of new adjuvant formulations has led to the selection of AS01B as a potential candidate possibly capable of prolonging persistence of the immune response. AS01B is a liposome-based adjuvant containing MPL® and QS21 (see Table 1).

Preclinical studies in mice and rhesus monkeys show that the AS01B adjuvant induces significantly superior antigen-specific cytotoxic T lymphocyte (CTL) and interferon gamma (IFN-gamma) responses than does AS02A, while humoral responses generated by both formulations remain equivalent. In rhesus monkey studies, AS01B also induces a cell-mediated response characterized by a more Th1-biased profile: a higher proportion of IFN-gamma-secreting lymphocytes than observed with AS02A. In toxicology studies in rabbits and primates the vaccine has been shown to be safe.

Most of the previous clinical experience with AS01B comes from a study of AS01B-adjuvanted hepatitis B vaccine. In this double blind Phase I/II immunogenicity and safety study in adults, AS01B was formulated with hepatitis B surface antigen (HBsAg). The HBsAg used in this study is encoded by the same gene as that used to express HBsAg in the RTS,S antigen, and the commercially available hepatitis B vaccine (Engerix-B). A total of 146 doses of HBsAg/AS01B have been administered to date to 49 healthy adults according to a 0, 1, 10 month vaccination schedule. No SAEs deemed causally related by the investigator to vaccination have been reported. No clinically significant abnormal hematological or biochemical laboratory test results have been reported. Most solicited symptoms reported after vaccination with HBsAg/AS01B were not severe in intensity and resolved in the course of the 7-day follow-up period after vaccination. All unsolicited symptoms that were causally related to HBsAg/AS01B vaccination and of intensity Grade 3 (symptoms that prevented normal everyday activity) were local (injection site) adverse events and resolved within 2 days. In conclusion, the AS01B adjuvant formulated with HBsAg is safe and induces an acceptable amount of reactogenicity.

In the evaluation of immunogenicity, HBsAg/AS01B induced a high and persistent humoral immune response as shown by the presence of seroprotective levels of HBs antibodies 40 weeks after the second dose of vaccine and dramatic boosting of geometric mean titers of anti-HBs antibodies to levels above 900 000 mIU/mL after Dose 3. The cell-mediated immune (CMI) responses to vaccination were characterized by a HBs-specific cytotoxic response, marked lymphoproliferation and IFN-gamma secretion but low interleukin-5 secretion. These CMI results are indicative of a Th1-like immune response and confirm the immunogenicity profile established for the AS01B adjuvant in preclinical studies with RTS,S/AS01B.

The AS01B formulation to be used in malaria vaccine trials is supplied in single-dose vials.

1.3.3. The RTS,S/AS02A candidate malaria vaccine; data collected to date

1.3.3.1. Trials of RTS,S/AS02A in adults

The RTS,S/AS02A vaccine has progressed through sequential stages of evaluation in trials in adults. Evaluation commenced with a series of trials to assess safety and proof-of-concept using the malaria sporozoite challenge model in malaria-naïve adults at the laboratories of WRAIR. Assessments of safety and immunogenicity have also been carried out on malaria-naïve adults in Belgium. Most relevant to this trial, the vaccine was subsequently assessed in malaria-experienced adults in malaria-endemic countries in Africa. An overview of the study design of each of the trials of RTS,S/AS02A in adults can be found in Table 4.

To date 14 trials in adults have been completed. During the course of these trials 607 volunteers have received 1608 doses of RTS,S/AS02A.

1.3.3.1.1. Safety experience of RTS,S/AS02A in malaria-experienced adults in Africa

Three studies of RTS,S/AS02A have been carried out in adults in a malaria-endemic region of The Gambia (Malaria-003, Malaria-004 and Malaria-005) and another in adults in a malaria-endemic region of Kenya (Malaria-008). The largest trial in malaria-experienced adults to date has been Malaria-005. In this double-blind, randomized, controlled Phase IIb study in a malaria-endemic region of The Gambia, 250 men received 3 doses of the vaccine on a 0, 1 and 5 month schedule and were followed for up to 15 weeks. Subsequently, 158 of the men received a fourth dose during the peak malaria season of the following year and were followed for up to 9 weeks. The Malaria-008 study in Kenya was an open Phase I trial to investigate safety and immunogenicity of RTS,S/AS02A in 20 adults on a 0, 1 and 6 month schedule.

In both of these studies, the vaccine was safe and well tolerated (refer to Table 2 and Table 3). Other trials of RTS,S/AS02A in malaria-experienced adults yielded comparable results.

Table 2 The frequency of solicited symptoms per dose during the 4-day follow-up period for each vaccine group (Total Cohort, Malaria-005)

		RTS,S/AS02A		Rabies Vaccine	
		n	%	n	%
Limited arm motion	Any	113	26	26	6
Pain	Any	378	87	228	53
Swelling	Any	0	0	1	0.2
Arthralgia	Any	55	13	41	10
Fever	Any	13	3	6	1
Headache	Any	158	36	111	26
Malaise	Any	118	27	70	16
Myalgia	Any	35	8	31	7
Nausea	Any	36	8	25	6

Table 3 The frequency of solicited symptoms per dose during the 4-day follow-up period for each vaccine group (Total Cohort, Malaria-008)

		RTS,S/AS02	
		n	%
Arthralgia	Any	27	45.0
	Grade 3	0	0.0
	Grade 3/Rel	0	0.0
Gastrointestinal	Any	24	40.0
	Grade 3	0	0.0
	Grade 3/Rel	0	0.0
Headache	Any	47	78.3
	Grade 3	1	1.7
	Grade 3/Rel	1	1.7
Malaise	Any	28	46.7
	Grade 3	1	1.7
	Grade 3/Rel	1	1.7
Myalgia	Any	13	21.7
	Grade 3	0	0.0
	Grade 3/Rel	0	0.0
Fever	Any	18	30.0
	Grade 3	0	0.0
	Grade 3/Rel	0	0.0

Pain at the injection site (almost all mild to moderate in intensity) has been the most frequently reported local solicited symptom in adults in all trials. In malaria-naïve populations, the most frequently reported general solicited symptoms causally related to vaccination have been myalgia and fatigue (a small number of transient severe cases have been observed). In studies in Africa, headache and malaise were the most frequently reported.

Only one serious adverse event has been suspected of being causally related to vaccination with RTS,S/AS02A in adults to date. A chronic carrier of HBsAg had a raised ALT during the vaccination course of Malaria-005. ALT levels returned to normal within 2 weeks. In the comparator group of the same study, four recipients of rabies vaccine had similarly deranged ALT levels. ALT levels for these subjects returned to normal ranges within 4 weeks.

1.3.3.1.2. *Efficacy of RTS,S/AS02A in adults*

Evidence of efficacy comes from both the malaria sporozoite challenge model in malaria-naïve adults in the laboratory and from field studies in malaria-experienced adults. The RTS,S/AS02A vaccine has been shown to protect between 42% and 86% of healthy non-immune adult volunteers against infection in homologous sporozoite challenge studies, when given according to a 2 or 3 dose vaccination schedule. In Malaria-005, VE was determined as the time to first detection of infection due to *P. falciparum*. Overall efficacy against infection, adjusted for covariates, was 34% (95% confidence interval [CI]: 8% to 53%). The estimated efficacy during the first 9 weeks of follow-up was 71% (95% CI: 46% to 85%), decreasing to 0% in the last 6 weeks. Vaccine efficacy against infection was 47% (95% CI: 3.8 to 71 %) and a strong boosting of immune responses was induced.

1.3.3.1.3. Safety and immunogenicity of alternative vaccination schedules in adults

Various vaccination schedules have been assessed in adults. These include 0, 1, 5 and 19 months (Malaria-005); 0, 1, 6 and13 months (Malaria-002) and 0, 1, and 9 months (WRMAL-004). All these schedules showed the vaccine to be immunogenic with acceptable reactogenicity in adults. There has been no significant increase in reactogenicity with a fourth vaccine dose. Data from a study conducted in the USA to evaluate a 0, 1 and 3-month and a 0, 7 and 28-day vaccination schedule in malaria-naïve adults (Malaria-012) indicates that the immune response and efficacy following sporozoite challenge are comparable to that after a 0, 1 and 6-month vaccination schedule (Malaria-009). No changes in the safety or reactogenicity profile of the vaccine were identified with either schedule.

1.3.3.1.4. Studies of RTS,S/AS02A in adults; conclusions

Evaluation of RTS,S/AS02A has gradually progressed from laboratory evaluation of safety and proof-of-concept in malaria-naïve adults to evaluation in the field in Africa in malaria-experienced adults. The vaccine has been shown to be safe, with acceptable reactogenicity. Efficacy against malaria in both the laboratory and the field have been demonstrated.

Table 4 Overview of clinical trials with the RTS,S/AS02A malaria vaccine in adults to date

Protocol number	Study design; Phase; Country	Vaccine formulation	Vaccine dose delivered	Schedule (months)	N	Doses given	Refer to Section	Age (Years)
WRMAL 003 (WRAIR 544)	Open randomized, challenge; Phase I/IIa; USA	RTS,S/AS04<br>RTS,S/AS03<br>RTS,S/AS02A	50µg RTS,S in 1 mL<br>50 µg RTS,S in 0.5 mL[1]<br>50 µg RTS,S in 0.5 mL[1]	0,1,7[2]<br>0,1,7[2]<br>0,1,7[2]	14<br>15<br>17	35<br>40<br>34		18-45
WRMAL 004 (A-7492)	Challenge study, some randomized groups, some non-randomized; Phase I/IIa; USA	RTS,S/AS02A<br>RTS,S/AS02A<br>RTS,S/AS02A<br>RTS,S/AS02A<br>RTS,S/AS02A<br>RTS,S/AS02A<br>AS02A	50 µg RTS,S in 0.5 mL (1 dose)<br>50 µg RTS,S in 0.5 mL (2 doses)<br>50 µg RTS,S in 0.5 mL (2 doses)<br>50 µg RTS,S in 0.5 mL (3 doses)<br>50 µg RTS,S in 0.25 mL (3 doses)<br>50 µg RTS,S in 0.1 mL (3 doses)<br>0.5 mL	0<br>0,1<br>0,1<br>0,1<br>0,1,9<br>0,1,9<br>0	11<br>16<br>20<br>13<br>12<br>9<br>6	11<br>32<br>40<br>39<br>36<br>27<br>6		18-45
WRMAL 005 (A-8420)	Open randomized, challenge; Phase I/IIa; USA	RTS,S/AS02A lyo<br>RTS,S/AS02A liq	50 µg RTS,S in 0.5 mL<br>50 µg RTS,S in 0.5 mL	0,1<br>0,1	25<br>9	47<br>18		18-45
Malaria-002 (257049-002)	Open, safety, immunogenicity; Phase I/II; Belgium	RTS,S/AS02A	50 µg RTS,S in 0.5 mL	0,1,6,13	10	40		18-50
Malaria-003[3] (257049-003)	Randomized, double-blind, controlled; Phase IIb; The Gambia	RTS,S/AS02A<br>Rabies vaccine[4]	50 µg RTS,S in 0.5 mL	0,1,5	73<br>67	145<br>134		15-45
Malaria-004 (257049-004)	Open, safety, immunogenicity; Phase I; The Gambia	RTS,S/AS02A	50 µg RTS,S in 0.5 mL	0,1,6	20	58		18-45
Malaria-005 (257049-005)	Randomized, double-blind, controlled; single-blind, controlled booster amendment; efficacy; Phase IIb; The Gambia	RTS,S/AS02A<br>Rabies vaccine[4]	50 µg RTS,S in 0.5 mL	0,1,5 (liq), Bo 19 (lyo)	153<br>153	436 + Bo 84<br>431 + Bo 87		18-45
Malaria-006 (257049-006)	Open, randomized, dose escalation; Phase I; Belgium	RTS,S/AS02A (lyo & liq)	10 µg RTS,S in 0.1 mL<br>25 µg RTS,S in 0.25 mL<br>50 µg RTS,S in 0.5 mL<br>50 µg RTS,S in 0.5 mL	0,1<br>0,1<br>0,1<br>0,1	3 (lyo)<br>3 (lyo)<br>10 liq<br>10 lyo	6<br>6<br>20<br>20		18-50
Malaria-007 (257049-007)	Open, randomized, safety, immunogenicity, Phase I; Belgium	TRAP/AS02A<br>RTS,S/AS02A<br>RTS,S & TRAP/AS02A	20 µg TRAP in 0.5 mL<br>50 µg RTS,S in 0.5 mL<br>50 µg RTS,S & 50 µg TRAP in 0.5 mL	0,1,6<br>0,1,6<br>0,1,6	10<br>10<br>20	30<br>30<br>60		18-50
Malaria-008 (257049-008)	Open, safety, immunogenicity; Phase I; Kenya	RTS,S/AS02A lyo	50 µg RTS,S in 0.5 mL	0,1,6	20	60		18-45

Protocol number	Study design; Phase; Country	Vaccine formulation	Vaccine dose delivered	Schedule (months)	N	Doses given	Refer to Section	Age (Years)
Malaria-009 (257049-009)	Double-blind, randomized, challenge; Phase I/IIa; USA	RTS,S & TRAP in AS02A	50 µg RTS,S & 20 µg TRAP in 0.5 mL	0,1	25	50		18-45
		TRAP/AS02A	20 µg TRAP in 0.5 mL	0,1	10	20		
		RTS,S & TRAP in AS02A[5]	50 µg RTS,S & 20 µg TRAP in 0.5 mL	0,1,6	--	--		
		TRAP/AS02A[5]	20 µg TRAP in 0.5 mL	0,1,6	--	--		
Malaria-012[6] (257049-012)	Open, randomized, challenge; Phase I/IIa; USA	RTS,S/AS02A	50 µg RTS,S in 0.5 mL	0,7,28 days	20	60		18-45
		RTS,S/AS02A	50 µg RTS,S in 0.5 mL	0,1,3	20	59		
Malaria-013 (257049-013)	Open, safety, immunogenicity; Phase I; USA	DNA prime & RTS,S/AS02A boost	50 µg RTS,S in 0.5 mL	0,2	10	20		18-45
		RTS,S/AS02A (comparator)	50 µg RTS,S in 0.5 mL	0,2	14	27		
Malaria-019[7] (257049-019)	Double-blind, randomized, challenge: Phase I/IIa; USA	MSP1/AS02A + RTS,S/AS02A[8]	25 µg RTS,S in 0.25 mL & 25 µg MSP1 in 0.25 mL	0,1,3	15	42		18-45
		MSP1 & RTS,S in AS02A + saline[9]	25 µg MSP1 & 25 µg MSP1 in 0.25 mL	0,1,3	15	42		
		MSP1/AS02A + AS02A[10]	25 µg MSP1 in 0.25 mL	0,1,3	15	42		
		RTS,S/AS02A + AS02A[11]	25 µg RTS,S in 0.25 mL & 0.25 mL AS02A	0,1,3	15	44		

AS03 = proprietary oil-in-water emulsion; AS04 = MPL® & Alum salt
Lyo = Lyophilized; Liq = Liquid; Bo = Booster; TRAP = Thrombospondin-related anonymous protein; N = Number of subjects enrolled
[1] Dose 3 reduced to 0.1mL
[2] RTS,S/AS04 and RTS,S/AS03 group vaccination schedule: 0, 4, 28 weeks (0, 1, 7 months) except for one subject (RTS,S/AS04 group) who received the 3rd dose during week 29; RTS,S/AS02A group vaccination schedule: 0, 4, 25 weeks (0, 1, 6-7 months)
[3] Due to antigen degradation in the specific study lot the 3rd dose was not administered and the efficacy component of the study was abandoned–a safety follow-up of the volunteers was completed uneventfully
[4] Human diploid cell rabies vaccine (Merieux HDCV) by Aventis Pasteur (Lyon, France)
[5] Three-dose cohort study was cancelled
[6] Clinical trials Malaria-010 and Malaria-011 planned with the RTS,S/TRAP/AS02A candidate vaccine were cancelled due to lack of efficacy observed in Malaria-009
[7] Malaria-019 compared the efficacy of concurrent administration of MSP1/AS02A and RTS,S/AS02A as well as an extemporaneous syringe mix of the two formulations with the corresponding monovalent vaccines (1/2 doses of all formulations). The study has completed, but is unreported to date.
[8] Each subject received concomitantly ½ dose of MSP1 (lyo)/AS02A and ½ dose of RTS,S (50 µg)/AS02A at separate injection sites
[9] Each subject received an *ad hoc* bedside mixture of ½ dose of MSP1 (lyo)/AS02A and ½ dose of RTS,S (50 µg)/AS02A at one injection site and normal saline at the other injection site
[10] Each subject received concomitantly ½ dose of MSP1 (lyo)/AS02A and ½ dose of AS02A at separate injection sites
[11] Each subject received concomitantly ½ dose of RTS,S (50 µg)/AS02A and ½ dose of AS02A at separate injection sites

Table 5 Overview of clinical trials with the RTS,S/AS02A malaria vaccine in children to date

Protocol number	Study design; Phase; Country	Vaccine formulation	Vaccine dose delivered	Schedule (months)	N	Doses given	Refer to Section	Age (Years)
Malaria-015 (257049-015)	Double-blind, dose escalation; Phase I; The Gambia	RTS,S/AS02A	50 µg RTS,S in 0.5 mL	0,1,3	20	60	1.3.3.2.1	6-11
		RTS,S/AS02A	25 µg RTS,S in 0.25 mL	0,1,3	20	59		
		RTS,S/AS02A	10 µg RTS,S in 0.1 mL	0,1,3	20	60		
		Rabies vaccine[1]		0,1,3	30	90		
Malaria-020 (257049-020)	Double-blind, dose escalation; Phase I; The Gambia	RTS,S/AS02A	50 µg RTS,S in 0.5 mL	0,1,3	30	89	1.3.3.2.1	1-5
		RTS,S/AS02A	25 µg RTS,S in 0.25 mL	0,1,3	30	89		
		RTS,S/AS02A	10 µg RTS,S in 0.1 mL	0,1,3	30	89		
		Rabies vaccine[3]		0,1,3	45	125		
Malaria-025 (257049-025)	Double-blind, randomized, safety, reactogenicity, immunogenicity; Phase I; Mozambique	RTS,S/AS02A	25 µg RTS,S in 0.25 mL	0,1,2	30	86	1.3.3.2.1	1-4
		Engerix-B			30	84		
Malaria-026 (257049-026)	Double-blind, randomized, efficacy, safety, reactogenicity, immunogenicity; Phase IIb; Mozambique	RTS,S/AS02A	25 µg RTS,S in 0.25 mL	0,1,2	1012	2926	1.3.3.2.2	1-4
		7 valent Pneumococcal conjugate (Dose 1& 3) *and* Hiberix (Dose 2): age < 24 months *OR* Engerix-B (Doses 1, 2 and 3): age ≥ 24 months	0.5 mL	0,1,2	1010	2912		
Malaria-034 (257049-034)	Double-blind (observer blind), randomized, safety, immunogenicity; Phase I/II; Mozambique	RTS,S/AS02D	25 µg RTS,S in 0.5 mL	0, 1, 2	100	293	1.3.3.2.3	3-5
		RTS,S/AS02A	25 µg RTS,S in 0.25 mL	0, 1, 2	100	288		

AS03 = proprietary oil-in-water emulsion; AS04 = MPL® & Alum salt
Lyo = Lyophilized; Liq = Liquid; Bo = Booster; TRAP = Thrombospondin-related anonymous protein; N = Number of subjects enrolled
[1] Human diploid cell rabies vaccine (Merieux HDCV) by Aventis Pasteur (Lyon, France)

1.3.3.2. Trials of RTS,S/AS02A in children

Following demonstration of the safety, immunogenicity and efficacy of the RTS,S/AS02A vaccine in adults, the candidate vaccine progressed to clinical evaluation in children.

To date, five trials of RTS,S/AS02A in children have been completed. During the course of these trials 1292 children have received 3751 doses of RTS,S/AS02A. An overview of the design of each of the studies of RTS,S/AS02A in children conducted to date can be found in Table 5.

1.3.3.2.1. Phase I pediatric trials of RTS,S/AS02A (Malaria-015, Malaria-020 and Malaria-025)

Two Phase I safety and immunogenicity studies evaluating 0.1 mL, 0.25 mL and 0.5 mL doses of RTS,S/AS02A have been conducted in Gambian children aged 1 to 11 years (Malaria-015 [GSK data on file] and Malaria-020 [GSK data on file])—a 0.5 mL dose of RTS,S/AS02A contains 50 μg RTS,S, 50 μg MPL®, 50 μg QS21 and oil-in-water emulsion. Each study included a control arm vaccinated with human diploid cell rabies vaccine (Aventis Pasteur, Lyon).

These studies were to aid in the selection of the dose level to be used in the future pediatric development of the RTS,S/AS02 candidate vaccine.

The solicited symptoms are displayed in Table 6 and Table 7 (corresponding to Malaria-015 and Malaria-020 respectively). Pain at the injection site was a frequent symptom across all study groups. The incidence of swelling increased with dose level. The majority of general solicited symptoms were mild to moderate in intensity and short lasting. In children aged 6 to 11 years headache was the most frequently occurring general solicited symptom in subjects receiving RTS,S/AS02A (any dose level). In the younger children (aged 1 to 5 years), the most frequently occurring general symptom in subjects receiving 0.1 mL dose RTS,S/AS02A was fever, reported after 11% of doses compared to 26% of doses in the rabies control group. In subjects receiving 0.25 mL dose RTS,S/AS02A, loss of appetite was the most frequent symptom, being reported after 12% of doses, which compared to 20% of doses in the control group. In subjects receiving 0.5 mL dose RTS,S/AS02A, irritability/fussiness was most frequently reported, occurring after 27% of doses compared to 12% of doses in the control group. Grade 3 general solicited symptoms were infrequent in both studies and resolved or decreased in intensity within 24 hours.

Unsolicited symptoms were recorded with similar (6 to 11 year-olds) or lower frequency (1 to 5 year-olds) in the study vaccine groups compared to the control vaccine groups. The majority of unsolicited symptoms were mild to moderate in intensity and unrelated to vaccination.

Hematocrit values were generally low but comparable between study groups. Six children in the RTS,S/AS02A groups (one in 0.1 mL dose group, five in 0.25 mL dose group) experienced moderate anemia, defined as a hematocrit of 15% to 24% during the period

to 30 days post Dose 3. There were no cases of anemia in the 0.5 mL or control groups. All these children had documented malaria episodes before or at the time that anemia was recorded.

All children were monitored for liver function. Among the 6 to 11 year-olds, two children in the RTS,S/AS02A groups experienced a transient rise in ALT levels judged not to be related to vaccination and not clinically relevant. In the younger children, increases in ALT levels from pre-vaccination were observed in 2 subjects in RTS,S/AS02A groups and 2 subjects in the rabies groups. Of these, only one case in the rabies control group was judged to be clinically relevant and vaccination was discontinued after Dose 1. No other clinically relevant abnormalities of hematological or biochemical laboratory parameters were observed.

In the interval to 30 days post Dose 3, 6 serious adverse events (SAEs) in total occurred. One SAE occurred in the 6 to 11 year old study; a case of bronchopneumonia was reported in a subject in the rabies-control group. The event was not considered causally related to vaccination and the subject made a full recovery. Five SAEs were reported in the 1 to 5 year old study. Among the RTS,S/AS02A recipients one subject suffered acute malaria with acute upper respiratory tract infection (0.1 mL dose) and one subject suffered cerebral malaria (0.25 mL dose). In the rabies control group three SAEs were reported: acute severe malaria with urinary tract infection and salmonella septicemia, bronchopneumonia with bronchial asthma and accidental death due to drowning. All SAEs were considered not to be related to study vaccines. Apart from the fatal SAE, all subjects made a full recovery and were not withdrawn from the study.

A total of 20 subjects out of 135 subjects enrolled in the Malaria-015 study were under 2 years of age. The safety and reactogenicity profiles of these subjects receiving the RTS,S/AS02A candidate vaccine were comparable to that seen in the older children.

Table 8 details the antibody responses to anti-CS and Table 9 the antibody responses to anti-HBs for these two studies. All dose levels of RTS,S/AS02A (0.1 mL, 0.25 mL and 0.5 mL doses) were highly immunogenic for anti-CS and anti-HBs antibodies, irrespective of pre-vaccination HBsAg serostatus.

For both Malaria-015 and Malaria-020, all subjects receiving RTS,S/AS02A were seropositive for anti-CS antibodies post Dose 2. Post Dose 3 the lowest anti-CS antibody geometric mean titers (GMTs) occurred in the 0.1 mL RTS,S/AS02A dose group (Table 8 below). In the 0.25 mL and 0.5 mL RTS,S/AS02A dose groups, similar GMT values were recorded. All subjects in the RTS,S/AS02A groups were seroprotected for anti-HBs antibodies post Dose 2 in Malaria-015 and post Dose 3 (first post-vaccination assessment) in Malaria-020; the highest GMTs were observed in the 0.25 mL RTS,S/AS02A dose group in Malaria-015 and in the 0.5 mL RTS,S/AS02A dose group in Malaria-020 (Table 9 below). Overall, the GMT values observed in this population of children aged 1 to 11 years were within the ranges seen in previous studies with the RTS,S/AS02A vaccine in malaria-naïve adult subjects [Kester 2001].

Table 6 Frequency of solicited symptoms per dose during the 4-day follow-up period for each vaccine group (Total Cohort, Malaria-015)

		**RTS,S/AS02A (0.1 mL)** N=60		**Rabies Vaccine** N=30		**RTS,S/AS02A (0.25 mL)** N=59		**Rabies Vaccine** N=30		**RTS,S/AS02A (0.5 mL)** N=60		**Rabies Vaccine** N=30	
Symptom		n	%	n	%	n	%	n	%	n	%	n	%
Injection site	Any	53	88.3	21	70.0	53	89.8	27	90.0	59	98.3	26	86.7
Pain	Grade 3	0	0.0	0	0.0	4	6.8	0	0.0	8	13.3	0	0.0
Injection site	Any	5	8.3	1	3.3	10	16.9	0	0.0	12	20.0	0	0.0
Swelling	Grade 3	1	1.7	0	0.0	1	1.7	0	0.0	4	6.7	0	0.0
Limited arm	Any	5	8.3	1	3.3	12	20.3	2	6.7	11	18.3	0	0.0
Motion	Grade 3	0	0.0	0	0.0	2	3.4	0	0.0	2	3.3	0	0.0
Fever*	Any*	6	10.0	0	0.0	5	8.5	3	10.0	6	10.0	2	6.7
	Grade 3	1	1.7	0	0.0	0	0.0	0	0.0	0	0.0	0	0.0
	Grade 3/Rel	0	0.0	0	0.0	0	0.0	0	0.0	0	0.0	0	0.0
Malaise	Any	5	8.3	0	0.0	8	13.6	2	6.7	11	18.3	2	6.7
	Grade 3	0	0.0	0	0.0	0	0.0	0	0.0	1	1.7	0	0.0
	Grade 3/Rel	0	0.0	0	0.0	0	0.0	0	0.0	1	1.7	0	0.0
Nausea	Any	4	6.7	1	3.3	2	3.4	1	3.3	6	10.0	0	0.0
	Grade 3	0	0.0	0	0.0	0	0.0	0	0.0	0	0.0	0	0.0
	Grade 3/Rel	0	0.0	0	0.0	0	0.0	0	0.0	0	0.0	0	0.0
Headache	Any	11	18.3	4	13.3	14	23.7	4	13.3	12	20.0	3	10.0
	Grade 3	0	0.0	0	0.0	0	0.0	0	0.0	2	3.3	0	0.0
	Grade 3/Rel	0	0.0	0	0.0	0	0.0	0	0.0	1	1.7	0	0.0

N = number of documented doses.
n/% = number/percentage of doses followed by a local/general symptom.
*oral body temperature ≥ 37.5°C.
Grade 3 limited arm motion; abduction at the shoulder < 30°.
Grade 3 injection site pain; spontaneously painful.
Grade 3 swelling; > 50 mm and persisting for more than 24 hours.
Grade 3 fever; oral body temperature ≥ 39.0°C.
For other symptoms; adverse event that prevents normal activity.
Rel = related to study vaccine.
Note: all local injection site symptoms were considered related to study vaccine.

Table 7 Frequency of solicited symptoms per dose during the 4-day follow-up period for each vaccine group (Total Cohort, Malaria-020)

		RTS,S/AS02A (0.1 mL) N=89		Rabies Vaccine N=39		RTS,S/AS02A (0.25 mL) N=89		Rabies Vaccine N=45		RTS,S/AS02A (0.5 mL) N=89		Rabies Vaccine N=41	
Symptom		n	%	n	%	n	%	n	%	n	%	n	%
Injection site Pain	Any	70	78.7	34	87.2	75	84.3	38	84.4	82	92.1	32	78.0
	Grade 3	1	1.1	0	0.0	5	5.6	1	2.2	13	14.6	0	0.0
Injection site Swelling	Any	5	5.6	2	5.1	23	25.8	2	4.4	26	29.2	3	7.3
	Grade 3	5	5.6	1	2.6	16	18.0	2	4.4	24	27.0	1	2.4
Fever*	Any*	10	11.2	10	25.6	8	9.0	9	20.0	15	16.9	5	12.2
	Grade 3	1	1.1	1	2.6	1	1.1	1	2.2	0	0.0	1	2.4
	Grade 3/Rel	0	0.0	1	2.6	1	1.1	1	2.2	0	0.0	1	2.4
Drowsiness	Any	8	9.0	2	5.1	7	7.9	4	8.9	5	5.6	2	4.9
	Grade 3	1	1.1	0	0.0	0	0.0	1	2.2	0	0.0	0	0.0
	Grade 3/Rel	0	0.0	0	0.0	0	0.0	0	0.0	0	0.0	0	0.0
Loss of Appetite	Any	7	7.9	1	2.6	11	12.4	9	20.0	13	14.6	4	9.8
	Grade 3	1	1.1	0	0.0	2	2.2	2	4.4	2	2.2	0	0.0
	Grade 3/Rel	0	0.0	0	0.0	2	2.2	1	2.2	2	2.2	0	0.0
Irritability/ Fussiness	Any	4	4.5	0	0.0	9	10.1	6	13.3	24	27.0	5	12.2
	Grade 3	0	0.0	0	0.0	0	0.0	1	2.2	3	3.4	0	0.0
	Grade 3/Rel	0	0.0	0	0.0	0	0.0	1	2.2	3	3.4	0	0.0

N = number of documented doses.
n/% = number/percentage of doses followed by a local/general symptom.
*oral body temperature ≥ 37.5°C.
Grade 3 injection site pain; cries when limb is moved/spontaneously painful.
Grade 3 swelling; > 20 mm.
Grade 3 fever; oral body temperature ≥ 39.0°C.
Grade 3 drowsiness; drowsiness that prevents normal activity.
Grade 3 loss of appetite; not eating at all.
Grade 3 irritability/fussiness; crying that cannot be comforted/prevents normal activity.
Rel = related to study vaccine.
Note: all local injection site symptoms were considered related to study vaccine.

Table 8 Geometric Mean Titers (GMT) for anti-CS antibody titers (ATP Cohort for Immunogenicity for each study) post Dose 3

	RTS,S/AS02 dose volume			RTS,S/AS02D (95% CI)	Rabies Vaccine (95% CI)	Engerix-B (95% CI)	Prevnar & Hiberix (95% CI)
Study	0.1 mL (95% CI)	0.25 mL (95% CI)	0.5 mL (95% CI)				
Malaria-015 [a]	30.5 (18.4—50.4)	73.7 (42.4 —128.0)	62.3 (38.7 —100.5)	--	1.0 (0.4—1.0)	--	--
Malaria-020 [a]	69.6 (49.8—97.2)	90.3 (60.7 —134.4)	84.1 (56.4 —125.4)	--	0.8 (0.7—1.0)	--	--
Malaria-025 [a]	--	270.4 (182.7—400.3)	--	--	--	0.6 (0.5—0.7)	--
Malaria-026 [b] (subj. < 24 m)	--	273.9 (228.7—328.1)	--	--	--	--	0.3 (0.3—0.3)
Malaria-026 [b] (subj. ≥ 24 m)	--	158.1 (141.9—176.2)	--	--		0.3 (0.3—0.4)	--
Malaria-034 [b]	--	179.6 (145.9—221.0)	--	190.9 (150.3—242.4)	--	--	--

[a]: GMTs for anti-CS antibodies were measured at WRAIR in μg/mL for these studies
[b]: GMTs for anti-CS antibodies were measured at GSK Biologicals in EL.U/mL for these studies

Table 9 Geometric Mean Titers (GMT) for anti-HBs antibody titers (ATP Cohort for Immunogenicity for each study) post Dose 3

Study	RTS,S/AS02 dose volume			RTS,S/AS02D (95% CI)	Rabies Vaccine (95% CI)	Engerix-B (95% CI)	Prevnar & Hiberix (95% CI)
	0.1 mL (95% CI)	0.25 mL (95% CI)	0.5 mL (95% CI)				
Malaria-015	16442 (9204—29372)	32374 (14418—72694)	16604 (8627—31955)	--	27 (9—74)	--	--
Malaria-020	34925 (18482—65999)	54711 (27784—107737)	68041 (39221—118036)	--	87 (31—239)	--	--
Malaria-025	--	10386 (4872—22142)	--	--	--	328 (108—99631)	--
Malaria-026 (subj. < 24 m)	--	51035 (27918—93291)	--	--	--	--	67 (33—135)
Malaria-026 (subj. ≥ 24 m)	--	11368 (8518—15171)	--	--		349 (236—517)	--
Malaria-034	--	23977 (17895—32126)	--	17410 (13322—22751)	--	--	--

The 0.25 mL dose RTS,S/AS02A was selected for future pediatric development, because it exhibited comparable immunogenicity to the 0.5 mL dose, with a tendency to a lower reactogenicity profile.

Subsequent to these studies, RTS,S/AS02A (0.25 mL dose) was evaluated in a double-blind, randomized, controlled Phase I study under a new schedule (0, 1, 2 month) in children aged 1 to 4 years in Mozambique (Malaria-025 [GSK data on file]). Subjects received RTS,S/AS02A (0.25 mL dose) or Engerix-B hepatitis B vaccine (GSK Biologicals, Rixensart, Belgium). The frequency of solicited symptoms per dose during the 4-day follow-up period for each vaccine group during the study is summarized in Table 10.

As previously observed in Malaria-020, local reactions at the site of injection were common. Unlike Malaria-020 where swelling at the injection site was the most frequently observed local reaction, pain was the most frequently observed reaction in Malaria-025. Grade 3 swelling was reported after 23% of doses of RTS,S/AS02A (0.25 mL dose), but did not occur following administration of Engerix-B. The incidence of swelling in the RTS,S/AS02A group decreased after Dose 2 of vaccine, but increased after Dose 3.

Few solicited general adverse events were reported. In the RTS,S/AS02A group the most frequently reported solicited adverse events related to vaccination were fever (9.5% after all doses) followed by loss of appetite (7.5% after all doses). In the Engerix-B comparator group, fever (1.2%) was the only solicited general adverse event related to vaccination. There was no trend in increase in solicited general adverse events related to vaccination with subsequent doses. Only one Grade 3 solicited general adverse event was reported in the study; one subject suffered fever in the RTS,S/AS02A group. All solicited general symptoms reported resolved within the 4 day follow-up period after vaccination. The frequency of solicited symptoms was similar to that previously observed in Malaria-020.

Unsolicited adverse events were recorded over a 30-day follow-up period after vaccination. Unsolicited symptoms were recorded with similar frequency in the study vaccine group compared to the control vaccine group. No unsolicited event was considered by the investigator to be related to vaccination. The unsolicited events

reflected the pattern of childhood morbidity expected in the population and unsolicited adverse events were balanced between treatment groups in terms of frequency and severity.

Table 10 Frequency of solicited symptoms per dose during the 4-day follow-up period for each vaccine group (Total Cohort, Malaria-025)

		RTS,S/AS02A (0.25 mL dose) N=84		Engerix-B N=83	
Symptom		n	%	n	%
Injection site pain	Any	25	29.8	3	3.6
	Grade 3	1	1.2	0	0.0
Injection site swelling	Any	63	75.0	37	44.6
	Grade 3	19	22.6	0	0.0
Fever	Any*	12	14.3	3	3.6
	Grade 3	0	0.0	1	1.2
	Grade 3/Rel	1	1.2	0	0.0
Drowsiness	Any	4	4.8	1	1.2
	Grade 3	0	0.0	0	0.0
	Grade 3/Rel	0	0.0	0	0.0
Loss of Appetite	Any	10	11.9	1	1.2
	Grade 3	0	0.0	0	0.0
	Grade 3/Rel	0	0.0	0	0.0
Irritability	Any	6	7.1	2	2.4
	Grade 3	0	0.0	0	0.0
	Grade 3/Rel	0	0.0	0	0.0

N = number of documented doses.
n/% = number/percentage of doses followed by a local/general symptom.
*oral body temperature ≥ 37.5°C.
Grade 3 injection site pain; cries when limb is moved/spontaneously painful.
Grade 3 swelling; > 20 mm.
Grade 3 fever; oral body temperature ≥ 39.0°C.
Grade 3 drowsiness; drowsiness that prevents normal activity.
Grade 3 loss of appetite; not eating at all.
Grade 3 irritability/fussiness; crying that cannot be comforted/prevents normal activity.
Rel = related to study vaccine.
Note: all local injection site symptoms were considered related to study vaccine.

Two children experienced moderate anemia (one child with a hematocrit of 15%, the other with 24%) during the period to one month post Dose 3. One child who received RTS,S/AS02A had anemia associated with an acute case of malaria. In the control group the cause was not identified but the child recovered when administered a course of antibiotics and iron supplementation.

In total 7 subjects in the control group and 3 subjects in the RTS,S/AS02A group had an elevation in ALT during the period to one months post Dose 3 (reference range < 60 IU/L). One subject in the control group was observed to have an ALT level of 61 at a single time point. The subject was clinically well and, because the value was just outside the reference range, he was not investigated further. In the other 9 subjects the raised ALT was associated with a viral hepatitis. Five of these subjects had acute hepatitis A. One subject in each group was a chronic carrier of hepatitis B and 1 subject in the RTS,S/AS02A group was suffering from acute hepatitis B. One other subject who

received Engerix-B was HBsAg positive and was a probable chronic carrier of hepatitis B; no confirmatory serology was performed.

No other clinically relevant abnormalities of hematological or biochemical laboratory parameters were observed.

A total of four serious adverse events were reported, two in each group, during the period to one month post Dose 3. One subject in the Engerix-B group suffered *P. falciparum* malaria; a second subject suffered glomerulonephritis secondary to skin lesions. In the RTS,S/AS02A (0.25 mL dose) group, one subject suffered a febrile convulsion 15 days after the second vaccination and another subject suffered from bronchopneumonia. All SAEs were considered not to be related to study vaccines. All subjects made a full recovery and were not withdrawn from the study.

All subjects receiving RTS,S/AS02A (0.25 mL dose) were seropositive (titers ≥ 1 µg/mL) for anti-CS antibodies 14 days after Dose 1 and titers increased with each subsequent injection. Post Dose 3 a GMC of 270.4 µg/mL (95% CI 183 to 400 µg/mL) was observed in the group receiving RTS,S/AS02A (0.25 mL dose), in comparison to the control group in which GMC did not change over time (Post Dose 3: GMC of 0.6 µg/mL, 95% CI 0.5 to 0.7 µg/mL) (Table 8 above). Antibody concentrations in this study were higher than those observed previously in The Gambia.

Antibody GMTs to HBsAg after three doses of RTS,S/AS02A (0.25 mL dose) were significantly higher than those observed after 3 doses of Engerix-B: GMT 10 387 (95% CI 4872 to 22 142) compared to GMT 329 (95% CI 108 to 996). One month post Dose 3, seroprotection rates (anti-HBs titers ≥ 10 mIU/mL) for RTS,S/AS02A (0.25 mL dose) and Engerix-B were 96.3% and 95.2% respectively. There was 1 non-responder in each group (ATP cohort): in the RTS,S/AS02A (0.25 mL dose) group the subject was a chronic carrier of hepatitis B virus, in the Engerix-B group the subject was HBsAg negative.

The experience of the use of RTS,S/AS02A in children to date is that it has an acceptable reactogenicity and safety profile. The 0.25 mL dose is highly immunogenic for anti-CS and anti-HBs antibodies.

1.3.3.2.2. Phase IIb pediatric trial of RTS,S/AS02A (Malaria-026)

The trial Malaria-026 evaluated the efficacy of the 0.25 mL Dose of RTS,S/AS02A in children aged 1 to 4 years in Mozambique. It was a double-blind randomized design; 2022 children received either 3 doses of the candidate malaria vaccine, containing 25µg RTS,S and 0.25mL AS02A adjuvant, or 3 doses of a control vaccine using a 0, 1 and 2 month vaccination schedule. The control vaccination regime was 3 doses of hepatitis B vaccine (Engerix-B, GlaxoSmithKline Biologicals), administered to children of 24 months and older, or 2 doses of a 7-valent pneumococcal conjugate vaccine (Prevnar®, Wyeth Lederle Vaccines) at Dose 1 and Dose 3 and 1 dose of *Hemophilus influenzae* type b vaccine (Hiberix™, GlaxoSmithKline Biologicals) at Dose 2 to children less than 24 months. Study subjects were recruited to two Cohorts for the study. Cohort 1 included children recruited from the town of Manhiça, Cohort 2 included children from the nearby

town of Ilha Josina. Cohort 2 was used to examine vaccine efficacy (VE) against infection.

Primary Efficacy Endpoint, Malaria-026

The Primary Efficacy Endpoint of the trial was the determination of the VE against clinical malaria disease and was evaluated in Cohort 1. The malaria Case Definition for the Primary Efficacy Endpoint was 'the presence of *P. falciparum* asexual parasitemia above 2500 per µL on Giemsa stained thick blood films and the presence of fever (oral temperature ≥ 37.5°C) at the time of presentation and occurring in a child who is unwell and brought for treatment to a healthcare facility'.

In Cohort 1 and Cohort 2 the RTS,S/AS02A and control groups were well-balanced for age, gender, bednet usage, distance of residence from health facility and geographical area of residence. Malaria transmission intensity, as indicated by Hct, prevalence of splenomegaly and IFAT, was higher in Ilha Josina where Cohort 2 was evaluated than Manhiça where Cohort 1 was evaluated. Malaria transmission was not constant over the six-month period. Malaria disease rates in the control group were lower in the second half of the observation period.

VE was determined over a six-month surveillance period commencing from 14 days post Dose 3. As specified per RAP, the time at risk was adjusted for malaria drug usage, residence in the study, withdrawal and death and the estimate of effect was adjusted for the covariates of age of the subject, geographical area of the residence of the subject, bednet use, and distance of the subject's residence from the nearest health center. The estimate of VE determined as the time to the first clinical episode in the ATP Cohort for Efficacy (i.e. all children who received full vaccination courses and contributed to the time at risk) was 26.9% (95% CI 7.4% to42.2%) and after adjustment for covariates was 29.9% (95% CI 11.0% to 44.8%).

No waning of efficacy over the 6-month observation period was noted for the primary endpoint when analyzed by different methods (test for proportionality of hazards with Schoenfeld residuals, p=0.139). Consistent with these data, at the cross-sectional survey 6.5 months after Dose 3, prevalence of parasitemia in recipients of RTS,S/AS02A was 37% lower than in the recipients of control vaccines; 11.9% in RTS,S/AS02A vs 18.9%in controls, p=0.0003). Parasite densities in these children were similar between RTS,S/AS02A recipients and controls (geometric mean density 2271 vs 2513; p=0.699).

Secondary Efficacy Endpoints of clinical malaria disease, Malaria-026

The study evaluated three other Case Definitions of clinical malaria disease specified per protocol. These endpoints explored different parasite density threshold values and history of fever as opposed to documented fever. The point estimates were consistent for all the Secondary Case Definitions of malaria disease.

For the first episode meeting Secondary Case Definition 1 for malaria episodes assessed over 6 months of Dose 3 defined as: the time to the first clinical episode of symptomatic *P. falciparum* malaria meeting Secondary Case Definition 1 for malaria episodes (the presence of *P. falciparum* asexual parasitemia [any level of parasitemia] on Giemsa

stained thick blood films *and* the presence of fever [oral temperature ≥ 37.5°C] at the time of presentation *and* occurring in a child who is unwell and brought for treatment detected by passive case detection) in children (1–4 years of age at first vaccination), determined over a six-month surveillance period post Dose 3; the VE was 28.6% (95% CI 10.4% to 43.1%; p=0.0036)

First episode meeting Secondary Case Definition 2 for malaria episodes assessed over 6 months of Dose 3: the time to the first clinical episode of symptomatic *P. falciparum* malaria meeting Secondary Case Definition 2 for malaria episodes (the presence of *P. falciparum* asexual parasitemia [any level of parasitemia] on Giemsa stained thick blood films *and* a history of fever within 24 hours or documented fever [oral temperature ≥ 37.5°C] at the time of presentation *and* occurring in a child who is unwell and brought for treatment) detected by passive case detection in children (1–4 years of age at first vaccination), determined over a six-month surveillance period post Dose 3; the VE was 33.8% (95% CI 19.7% to 45.3%; p=<0.001)

First episode meeting Secondary Case Definition 3 for malaria episodes assessed over 6 months of Dose 3: the time to the first clinical episode of symptomatic *P. falciparum* malaria meeting Secondary Case Definition 3 (the presence of *P. falciparum* asexual parasitemia above 15 000 per µL on Giemsa stained thick blood films *and* the presence of fever [oral temperature ≥ 37.5°C] at the time of presentation *and* occurring in a child who is unwell and brought for treatment to a healthcare facility) detected by passive case detection in children (1–4 years of age at first vaccination), determined over a six-month surveillance period post Dose 3; the VE was 31.7% (95% CI 11.5% to 47.2%; p=0.0039).

Incidence of moderate anemia, Malaria-026

All children presenting to a Health Center as outpatients who were evaluated for malaria also had a blood sample taken by fingerprick to determine their hematocrit (Hct). It should be noted that there was no requirement for concurrent malaria parasitemia in this case definition and this therefore measures all cause anemia, of which in the study area malaria is the most important cause. The adjusted estimate of VE against first incident case of moderate anemia (defined as Hct < 25%) was 28.2% (95% CI -19.6% to 56.9%; p=0.203) in the ATP Cohort for Efficacy.

All clinical episodes meeting Primary Case Definition for Malaria, Malaria-026

Relatively few children had second or third episodes of malaria that met the Primary Case Definition (see Table 11). No child experienced more than 3 episodes of clinical malaria during the course of the trial. The significance of the differences in the distribution of the number of episodes was p=0.0508 assessed using a Fisher exact test.

Table 11 Frequency of children according to the number of episodes by treatment group (ATP Cohort for Efficacy) (Cohort 1), Malaria-026

Number of episodes per child	R(1) N= 745		C(1) N= 745		Total N= 1490		p-value
	Value or n	%	Value or n	%	Value or n	%	
0	622	83.5	586	78.7	1208	81.1	0.0508
1	99	13.3	131	17.6	230	15.4	-
2	18	2.4	25	3.4	43	2.9	-
3	6	0.8	3	0.4	9	0.6	-

R(1): RTS,S/AS02A (Cohort 1, < 24 months)/ RTS,S/AS02A (Cohort 1, ≥ 24 months)
C(1): Prevnar and Hiberix (Cohort 1, < 24 months)/ Engerix-B (Cohort 1, ≥ 24 months)
N=number of subjects
n=number of subjects in a given category
Value=value of the considered parameter
%=n / Number of subjects with available results x 100
p-value: Fisher Exact test

To estimate the VE against all clinical episodes of malaria meeting the Primary Case Definition, the time at risk was adjusted using all of the criteria for the Primary Efficacy Endpoint with the addition of an exclusion period of 28 days after an episode. The adjusted VE against all clinical episodes in the ATP Cohort for Efficacy was 27.3% (95% CI 6.2%to 43.8; p=0.0143).

Exploratory Efficacy Endpoints of more severe disease, Malaria-026

The malaria transmission season over which the trial was conducted was particularly intense. The increased number of cases (compared to number expected), particularly those at the severe end of the spectrum, allowed the inclusion of Exploratory Efficacy Endpoints to evaluate severe disease to the RAP. It should be emphasized that all case determination was done systematically according to predefined criteria and prior to unblinding.

Malaria requiring hospitalization

Malaria requiring hospitalization was defined as an admission where malaria was either the sole cause of illness or a significant contributing factor. It was analyzed as the proportion of children experiencing one or more episodes during the six-month surveillance period post Dose 3 in the ATP cohort. There were 42 affected children in recipients of RTS,S/AS02A compared to 62 in the controls. The unadjusted estimate of VE was 32.3% (95% CI 1.3% to 53.9%; p=0.053).

Severe malaria disease

The proportion of children affected by one or more episodes of severe malaria meeting the Case Definition was compared. No adjustment was made for covariates. There were 11 cases of severe malaria disease in recipients of RTS,S/AS02A compared to 26 cases in recipients of control vaccine(s); the measured VE was 57.7% (95% CI 16.2% to 80.6%, p=0.019).

Total number of hospital admissions

The analysis of the total number of hospital admissions is restricted to those hospital admissions which occurred in the six-month surveillance period and in children belonging to the ATP Cohort for Efficacy, Cohort 1. In total there were 79 admissions in the RTS,S/AS02A group and 90 in the control Table 12. The Fisher exact test showed no statistical significance difference between the groups.

Table 12 Frequency distribution of all-cause hospital admissions (ATP Cohort for Efficacy), Malaria-026

Characteristics	Parameters or Categories	R(1) N= 745		C(1) N= 745		Total N= 1490		p-value
		Value or n	%	Value or n	%	Value or n	%	
Hospital admissions	0	678	91.0	665	89.3	1343	90.1	0.5281
	1	56	7.5	71	9.5	127	8.5	-
	2	10	1.3	8	1.1	18	1.2	-
	3	1	0.1	1	0.1	2	0.1	-

R(1): RTS,S/AS02A (Cohort 1, < 24 months)/ RTS,S/AS02A (Cohort 1, ≥ 24 months)
C(1): Prevnar and Hiberix (Cohort 1, < 24 months)/ Engerix-B (Cohort 1, ≥ 24 months)
N=number of subjects
n=number of subjects in a given category
Value=value of the considered parameter
%=n / Number of subjects with available results x 100
p-value: Fisher Exact test

An analysis was performed comparing the rate of all hospital admissions. The time at risk was adjusted in a similar way to the time at risk for the analysis except that there was no adjustment for antimalarial drug usage and absences from the study area. The estimate was not adjusted for covariates. The unadjusted estimate of VE was 14.4% (95% CI -19.7% to 38.8%; p=0.362).

Association of CS response with efficacy, Malaria-026

As specified in the RAP, two approaches to the analysis of the association of the CS response with hazard rate in the RTS,S/AS02A recipients were used. The association between CS and Hazard Rate by means of a comparison of hazard rates between the higher tertile (measured in the treated group) against the lower tertile of the anti-CS antibodies as well as the hazard rates per ten-fold increase in the value of anti-CS antibodies was calculated. Neither analysis detected an association reaching statistical significance (see Table 13)

Table 13 Time to the first episode of clinical malaria according to anti-CS value (ATP Cohort for Efficacy) (Cohort 1), Malaria-026

Label	Hazard Ratio	LL	UL	p-value
Per 10-fold increase	0.94	0.66	1.33	0.7079
Higher versus Lower Tertile	1.38	0.89	2.12	0.1498

LL=95% lower limit
UL=95% upper limit
p-value from Cox proportional hazards model

Efficacy analysis in the ITT Cohort from Day 0, Malaria-026

For safety reasons, the FDA requested the analyses from Day 0 be conducted on the Primary Case Definition in the ITT Cohort. This was repeated for all clinical attacks fitting the Primary Case Definition and the incidence of anemia. For the analysis of the ITT Cohort the time at risk was not adjusted for temporary travel from the study area, malaria drug usage or for previous clinical episodes, and no adjustment was made for covariates.

The VE for the Primary Case Definition was 30.2% (95% CI 14.4% to 43.0%; p < 0.001). The estimate of VE against all clinical episodes was 33.5% (95% CI 16.4% to 47.1%; p < 0.001). The estimate for VE against incidence of anemia was 24.8% (95% CI -12.8% to 49.9%; p=0.168). The estimate for VE for severe malaria disease was 48.5% (95% CI 7.3% to 73.1%; p=0.027); for hospitalized malaria the VE was estimated to be 31.2% (95% CI 3.1% to 51.5%; p=0.032).

The results in the ITT Cohort from Day 0 were consistent with the results of the ATP Cohort for Efficacy post Dose 3 for all the endpoints analyzed

Efficacy against infection, Malaria-026

The first infection could be detected actively on a scheduled home visit or at the time a child presented at a health facility with clinical malaria. All children received sulfadoxine-pyrimethamine and amodiaquine 14 days prior to Dose 3 and only children with a negative blood slide for malaria parasites were included in the ATP Cohort for Efficacy. As specified in the RAP, the time at risk was adjusted for malaria drug usage, residence in the study, withdrawal and death. The unadjusted estimate of VE determined as the time to the first infection in the ATP Cohort for Efficacy (i.e. all children who received full vaccination courses and contributed to the time at risk) was 44.7% (95% CI 31.0% to 55.6%; p ≤ 0.001). After adjustment for the covariates of age of subject, bednet usage and distance of residence of subject from a health center, the VE was 45.0% (95% CI 31.4% to 55.9%; p ≤ 0.001).

Immunogenicity, Malaria-026

Full details of the CS and HBs responses for Malaria-026 can be found in Table 8 and Table 9 respectively.

A formal evaluation of non-inferiority of RTS,S/AS02A over Engerix-B in terms of anti-HBs response was completed in children 2 to 4 years of age in Malaria-026. Table 14 shows the outcome of the assessment of non-inferiority of RTS,S/AS02A compared to Engerix-B with respect to seroprotection rates in subjects HBsAg negative pre-vaccination. The lower limit of the 95% CI for the difference in anti-HBs seroprotection rates (≥10 mIU/mL) was 4.3%, hence greater than the *a priori* defined limit of -10%, thus demonstrating non-inferiority of RTS,S/AS02A compared to Engerix-B.

Table 14 Difference between groups in percentage of HBsAg negative subjects with anti-HBs titer of at least 10 mIU/mL one month post Dose 3 (ATP cohort for immunogenicity)

						Inference			
						Diff	Value	95% CI	
Group	N	%	Group	N	%		%	LL	UL
C(2)>24	111	91.9	R(2)>24	126	100.0	R(2)>24 - C(2)>24	8.1	4.3	14.7

R(2)>24 : RTS,S/AS02A (Cohort 2, ≥24 months)
C(2)>24 : Engerix-B (Cohort 2, ≥24 months)
N = number of subjects with available results
% = percentage of subjects with anti-HBs titer ≥ 10 mIU/mL
Diff = Difference calculated
CI = Standardized asymptotic confidence interval
LL=95% lower limit
UL=95% upper limit

Table 15 shows the outcome of the assessment of non-inferiority of RTS,S/AS02A compared to Engerix-B with respect to the ratio of anti-HBs GMTs in HBsAg negative subjects at baseline. The upper limit of the 95% CI for the ratio of anti-HBs GMTs was 0.04, hence lower than the *a priori* defined limit of 2.0, thus demonstrating non-inferiority of RTS,S/AS02A compared to Engerix-B.

Table 15 Non-inferiority in terms of anti-HBs GMTs in HBsAg negative subjects one month post Dose 3; Point estimate and CI for the ratio of GMTs (ATP cohort for immunogenicity)

	N in C(2)	N in R(2)	Ratio	95% CI	
				LL	UL
C(2)>24 vs R(2)>24	111	126	0.02754	0.0176	0.0431

C(2)>24 : Engerix-B (Cohort 2, ≥24 months)
R(2)>24 : RTS,S/AS02A (Cohort 2, ≥24 months)
LL=95% lower limit
UL=95% upper limit

A seroprotection rate of > 97% was achieved. Again, RTS,S/AS02A was found to be highly immunogenic for anti-HBs antibodies. At 30 days post Dose 3 of RTS,S/AS02A, GMTs of 51 035 (95% CI 27 919 to 93 292) were observed in recipients of RTS,S/AS02A younger than 24 months of age; for children older than 24 months, GMTs of 11 369 were observed (8519 to 15 172) at the same timepoint. GMTs remained high at 180 days post Dose 3: 13 642 (95% CI 7342 to 25 347) in children younger than 24 months, 4556 (3500 to 5932 in children older than 24 months). The response to HBsAg was approximately 32 fold greater with RTS,S/AS02A compared to Engerix-B.

Safety and reactogenicity, Malaria-026

Solicited symptoms are shown in Table 16 and Table 17, corresponding to results from Cohort 1 and Cohort 2 respectively. As with previous studies with RTS,S/AS02A, local reactions at the site of injection were common. Pain was the most frequently observed local symptom. In the recipients of RTS,S/AS02A, the most commonly observed solicited general adverse event related to vaccination was fever (In Cohort 1, 11.1% after all doses in children < 24 months of age, 3.7% after all doses in children ≥ 24 months of age: in Cohort 2 related fever was observed after 4.9% of doses in children < 24 months, 1.3% of doses in children ≥ 24 months). In recipients of control vaccines in Cohort 1, fever was also the most commonly observed solicited adverse event related to vaccination (1.1% in children < 24 months of age [who received Prevnar and Hiberix] and 0.9% in children > 24 months of age [who received Engerix-B]). For recipients of RTS,S/AS02A < 24 months of age, a trend of increased local reactogenicity with sequential doses was observed. For recipients of RTS,S/AS02A ≥ 24 months of age, local reactogenicity was

most frequent following Dose 3. The incidence of general symptoms of drowsiness, irritability, loss of appetite and fever (≥ 37.5°C) was higher in recipients of RTS,S/AS02A than recipients of control vaccine; Grade 3 events were infrequent. In children < 24 months, the most commonly reported AEs were Upper Respiratory Tract infection, Malaria and Diarrhea and in children ≥ 24 months Upper Respiratory Tract Infection (URTI), Malaria and Ascariasis; frequencies were similar in the RTS,S/AS02A and control groups.

Table 16 Frequency of solicited symptoms per dose during the 4-day follow-up period for each vaccine group (Total Cohort, Cohort 1, Malaria-026)

**Dose**		**COHORT 1 All/Dose**															
Group		R(1)<24				C(1)<24				R(1)>24				C(1)>24			
N		558				551				1768				1768			
		n	%	95% CI		n	%	95% CI		n	%	95% CI		n	%	95% CI	
				LL	UL			LL	UL			LL	UL			LL	UL
Pain	Any	165	29.6	25.8	33.5	43	7.8	5.7	10.4	459	26.0	23.9	28.1	78	4.4	3.5	5.5
	Grade 3	2	0.4	0.0	1.3	0	0.0	0.0	0.7	3	0.2	0.0	0.5	1	0.1	0.0	0.3
Swelling	Any	133	23.8	20.4	27.6	54	9.8	7.4	12.6	314	17.8	16.0	19.6	54	3.1	2.3	4.0
	Grade 3	45	8.1	5.9	10.6	8	1.5	0.6	2.8	104	5.9	4.8	7.1	1	0.1	0.0	0.3
Drowsiness	Any	38	6.8	4.9	9.2	15	2.7	1.5	4.5	46	2.6	1.9	3.5	25	1.4	0.9	2.1
	Grade 3	5	0.9	0.3	2.1	1	0.2	0.0	1.0	5	0.3	0.1	0.7	2	0.1	0.0	0.4
	Rel* Grade 3	0	0.0	0.0	0.7	0	0.0	0.0	0.7	0	0.0	0.0	0.2	0	0.0	0.0	0.2
Irritability	Any	73	13.1	10.4	16.2	14	2.5	1.4	4.2	53	3.0	2.3	3.9	22	1.2	0.8	1.9
	Grade 3	4	0.7	0.2	1.8	1	0.2	0.0	1.0	3	0.2	0.0	0.5	3	0.2	0.0	0.5
	Rel* Grade 3	0	0.0	0.0	0.7	0	0.0	0.0	0.7	0	0.0	0.0	0.2	0	0.0	0.0	0.2
Loss of Appetite	Any	101	18.1	15.0	21.6	33	6.0	4.2	8.3	130	7.4	6.2	8.7	62	3.5	2.7	4.5
	Grade 3	6	1.1	0.4	2.3	2	0.4	0.0	1.3	4	0.2	0.1	0.6	3	0.2	0.0	0.5
	Rel* Grade 3	0	0.0	0.0	0.7	0	0.0	0.0	0.7	0	0.0	0.0	0.2	0	0.0	0.0	0.2
Temperature	Any	152	27.2	23.6	31.1	32	5.8	4.0	8.1	173	9.8	8.4	11.3	91	5.1	4.2	6.3
	Grade 3	10	1.8	0.9	3.3	1	0.2	0.0	1.0	7	0.4	0.2	0.8	6	0.3	0.1	0.7
	Rel* Grade 3	4	0.7	0.2	1.8	0	0.0	0.0	0.7	1	0.1	0.0	0.3	1	0.1	0.0	0.3

R(1)<24; recipients of RTS,S/AS02A less than 24 months of age (Cohort 1)
C(1)<24; recipients of control vaccines (Prevnar & Hiberix) less than 24 months of age (Cohort 1)
R(1)>24; recipients of RTS,S/AS02A greater than or equal to 24 months of age (Cohort 1)
C(1)>24; recipients of control vaccines (Engerix-B) greater than or equal to 24 months of age (Cohort 1)
N = number of documented doses.
n/% = number/percentage of doses followed by a local/general symptom.
LL=95% lower limit
UL=95% upper limit
*oral body temperature ≥ 37.5°C.
Grade 3 injection site pain; cries when limb is moved/spontaneously painful.
Grade 3 swelling; > 20 mm.
Grade 3 fever; oral body temperature ≥ 39.0°C.
Grade 3 drowsiness; drowsiness that prevents normal activity.
Grade 3 loss of appetite; not eating at all.
Grade 3 irritability/fussiness; crying that cannot be comforted/prevents normal activity.
Rel = related to study vaccine.
Note: all local injection site symptoms were considered related to study vaccine.

Table 17 Frequency of solicited symptoms per dose during the 4-day follow-up period for each vaccine group (Total Cohort, Cohort 2, Malaria-026)

Dose		COHORT 2 All/Dose															
Group		R(2)<24				C(2)<24				R(2)>24				C(2)>24			
N		143				144				457				449			
		n	%	95% CI		n	%	95% CI		n	%	95% CI		n	%	95% CI	
				LL	UL			LL	UL			LL	UL			LL	UL
Pain	Any	40	28.0	20.8	36.1	9	6.3	2.9	11.5	80	17.5	14.1	21.3	12	2.7	1.4	4.6
	Grade 3	0	0.0	0.0	2.5	0	0.0	0.0	2.5	2	0.4	0.1	1.6	0	0.0	0.0	0.8
Swelling	Any	48	33.6	25.9	41.9	12	8.3	4.4	14.1	113	24.7	20.8	28.9	18	4.0	2.4	6.3
	Grade 3	28	19.6	13.4	27.0	3	2.1	0.4	6.0	47	10.3	7.7	13.4	2	0.4	0.1	1.6
Drowsiness	Any	14	9.8	5.5	15.9	8	5.6	2.4	10.7	14	3.1	1.7	5.1	7	1.6	0.6	3.2
	Grade 3	1	0.7	0.0	3.8	0	0.0	0.0	2.5	3	0.7	0.1	1.9	0	0.0	0.0	0.8
	Rel* Grade 3	0	0.0	0.0	2.5	0	0.0	0.0	2.5	0	0.0	0.0	0.8	0	0.0	0.0	0.8
Irritability	Any	19	13.3	8.2	20.0	2	1.4	0.2	4.9	13	2.8	1.5	4.8	4	0.9	0.2	2.3
	Grade 3	1	0.7	0.0	3.8	0	0.0	0.0	2.5	0	0.0	0.0	0.8	0	0.0	0.0	0.8
	Rel* Grade 3	0	0.0	0.0	2.5	0	0.0	0.0	2.5	0	0.0	0.0	0.8	0	0.0	0.0	0.8
Loss of Appetite	Any	27	18.9	12.8	26.3	12	8.3	4.4	14.1	28	6.1	4.1	8.7	10	2.2	1.1	4.1
	Grade 3	1	0.7	0.0	3.8	0	0.0	0.0	2.5	2	0.4	0.1	1.6	0	0.0	0.0	0.8
	Rel* Grade 3	0	0.0	0.0	2.5	0	0.0	0.0	2.5	0	0.0	0.0	0.8	0	0.0	0.0	0.8
Temperature	Any	40	28.0	20.8	36.1	9	6.3	2.9	11.5	57	12.5	9.6	15.9	48	10.7	8.0	13.9
	Grade 3	2	1.4	0.2	5.0	1	0.7	0.0	3.8	1	0.2	0.0	1.2	3	0.7	0.1	1.9
	Rel* Grade 3	0	0.0	0.0	2.5	0	0.0	0.0	2.5	0	0.0	0.0	0.8	0	0.0	0.0	0.8

R(2)<24; recipients of RTS,S/AS02A less than 24 months of age (Cohort 2)
C(2)<24; recipients of control vaccines (Prevnar & Hiberix) less than 24 months of age (Cohort 2)
R(2)>24; recipients of RTS,S/AS02A greater than or equal to 24 months of age (Cohort 2)
C(2)>24; recipients of control vaccines (Engerix-B) greater than or equal to 24 months of age (Cohort 2)
N = number of documented doses.
n/% = number/percentage of doses followed by a local/general symptom.
LL=95% lower limit
UL=95% upper limit
*oral body temperature ≥ 37.5°C.
Grade 3 injection site pain; cries when limb is moved/spontaneously painful.
Grade 3 swelling; > 20 mm.
Grade 3 fever; oral body temperature ≥ 39.0°C.
Grade 3 drowsiness; drowsiness that prevents normal activity.
Grade 3 loss of appetite; not eating at all.
Grade 3 irritability/fussiness; crying that cannot be comforted/prevents normal activity.
Rel = related to study vaccine.
Note: all local injection site symptoms were considered related to study vaccine.

Fifteen subjects died during the double-blind phase of the Malaria-026 study; 5 had received RTS,S/AS02A and 10 had received control vaccines. Four deaths of recipients of control vaccines had malaria as a sole or contributing factor; no deaths of recipients of RTS,S/AS02A had malaria as a contributing factor. None of the deaths were, in the judgment of the investigator, related to vaccination. Extrapolating from the mortality rate in the study population, approximately 17 deaths would be expected (this calculation assumes an adjusted seasonal rate of 24 per 1000 child years at risk [personal communication, Pedro Alonso, October 2003]) and 1010 child-years of observation approximately 17 July 2003 through 29 March 2004). The observed rate is lower and can be attributed to the 'healthy cohort effect'; only fit children were enrolled in the trial and they remained under close medical surveillance.

There was a tendency for the proportion of children experiencing an SAE to be lower in the recipients of RTS,S/AS02A compared to recipients of control vaccines. In Cohort 1, for recipients of RTS,S/AS02A < 24 months of age, 25% of children suffered at least one

SAE; for recipients of RTS,S/AS02A ≥ 24 months of age, 14% of subjects suffered at least one SAE. This is compared to recipients of Prevnar and Hiberix (children < 24 months of age), 37% of whom suffered at least one SAE; for recipients of Engerix-B (those children ≥ 24 months of age) 20% of subjects suffered at least one SAE. It should be noted that apart from five deaths that occurred at home, the non-hospitalized SAEs were all children with episodes of acute malaria associated with a high parasite density (defined as 5+ parasitemia on quick read).

There were no SAEs judged related to vaccination.

In Cohort 1, SAE reports of malaria tended to be fewer in recipients of RTS,S/AS02A. In children <24 months who received RTS,S/AS02A 19% of children experienced a malaria episode compared to 33% in the control group. There was a similar trend in the children ≥ 24 months; in the recipients of RTS,S/AS02A 13% experienced a malaria episode compared to 16% in the recipients of control vaccines. In Cohort 2, where surveillance for malaria was intense, no difference was observed. For the other SAEs classified at the MedDRA preferred term level, there were no differences in the pattern of morbidity observed between recipients of RTS,S/AS02A and recipients of control vaccines. The pattern of morbidity and SAEs in the study participants was similar to that previously observed at the study site (personal communication, Dr. Pedro Alonso, October 2003) and that has been described in the region [Fidel, 2002; Iriso, 2000].

1.3.3.2.3. Phase II pediatric trial of RTS,S/AS02D; Bridging Study (Malaria-034)

Malaria-034 was a phase I/II randomized double-blind bridging study to evaluate the safety and immunogenicity of 3 doses of GlaxoSmithKline Biologicals' candidate vaccine RTS,S/AS02D (0.5 mL dose) in comparison to 3 doses of the existing formulation RTS,S/AS02A (0.25 mL dose) administered to children aged 3 to 5 years living in a malaria-endemic region of Mozambique. Both vaccines contain the same constituents but the final volume was adjusted to be compatible with existing EPI practices.

Two hundred children were enrolled into two groups of 100 subjects each, of which 189 (93 of whom received RTS,S/AS02D [D group] and 96 received RTS,S/AS02A [A group]) subjects were included in the ATP analysis of safety and reactogenicity and 143 (67 and 76 subjects respectively) subjects in the analysis of immunogenicity.

The safety profile of the two vaccine formulations was similar. Solicited symptoms were as shown in Table 18, with pain the more frequent local symptom and fever the most frequent general symptom reported. Symptoms were generally mild to moderate in intensity, and decreased in frequency with subsequent doses.

Unsolicited symptoms were reported following 69 (24.7%) doses in the RTS,S/AS02D group and following 56 (19.4%) in the RTS/SAS02A group. The most frequently reported symptoms were malaria (18% of the subjects in the D group and 25% in the A group) and upper respiratory tract infection (14% of the subjects in the D group and 12% in the A group). An unsolicited symptom, injection site erythema, deemed to be related to vaccination was reported for a single subject, following one dose in the D group. Three

symptoms in the D group and 8 in the A group were graded 3 in intensity, none of which were related to vaccination.

One serious adverse event occurred in a subject in the A group. This subject had severe malaria which lasted 4 days and required hospitalization. The subject recovered and the event was deemed by the investigator to be unrelated to vaccination.

Table 18: Incidence of any and Grade 3 local and general solicited symptoms in the 7-day follow-up after vaccination

Group		RTS,S/AS02D (N = 93)				RTS,S/AS02A (N = 96)			
		n	%	95% CI		n	%	95% CI	
				LL	UL			LL	UL
Pain	Any	31	33.3	23.9	43.9	43	44.8	34.6	55.3
	Grade 3	2	2.2	0.3	7.6	1	1.0	0.0	5.7
Swelling	Any	8	8.6	3.8	16.2	23	24.0	15.8	33.7
	Grade 3	1	1.1	0.0	5.8	4	4.2	1.1	10.3
Drowsiness	Any	12	12.9	6.8	21.5	8	8.3	3.7	15.8
	Grade 3	2	2.2	0.3	7.6	1	1.0	0.0	5.7
Fever	Any	26	28.0	19.1	38.2	25	26.0	17.6	36.0
	Grade 3	6	6.5	2.4	13.5	3	3.1	0.6	8.9
Irritability	Any	11	11.8	6.1	20.2	3	3.1	0.6	8.9
	Grade 3	3	3.2	0.7	9.1	1	1.0	0.0	5.7
Loss of Appetite	Any	15	16.1	9.3	25.2	14	14.6	8.2	23.3
	Grade 3	0	0.0	0.0	3.9	0	0.0	0.0	3.8

For each dose and overall/subject:
95%CI = Exact 95% confidence interval
N = number of subjects having received at least one dose
n/% = number/percentage of subjects reporting a specified symptom
LL=95% lower limit
UL=95% upper limit
Grade 3 injection site pain; cries when limb is moved/spontaneously painful.
Grade 3 swelling; > 20 mm.
Fever: oral body temperature ≥ 37.5°C.
Grade 3 fever; oral body temperature ≥ 39.0°C.
Grade 3 drowsiness; drowsiness that prevents normal activity.
Grade 3 loss of appetite; not eating at all.
Grade 3 irritability/fussiness; crying that cannot be comforted/prevents normal activity

Non-inferiority of the RTS,S/AS02D vaccine formulation compared to the RTS,S/AS02A formulation was proven with respect to the immune response elicited. Table 19 shows the anti-CS seropositivity rates and GMTs one month following the third vaccine dose and Table 20 gives the ratios of post vaccination GMTs. All subjects in both groups were seropositive for anti-CS antibodies one month following the third vaccine dose. The RTS,S/AS02A:RTS,S/AS02D anti-CS GMT ratio was 0.9, with as upper limit of 95% CI: 1.3, hence below the *a priori* defined clinical limit of non-inferiority.

Table 19 Seropositivity rates and geometric mean titers (GMT) for anti-CS antibody titers one month after Dose 3 (ATP cohort for immunogenicity)

Group	N	≥ 0.5 EU/mL				GMT			MIN	MAX
		n	%	95% CI		Value	95% CI			
				LL	UL		LL	UL		
RTS,S/AS02D	67	67	100.0	94.6	100.0	190.9	150.3	242.4	14.3	1314.3
RTS,S/AS02A	76	76	100.0	95.3	100.0	179.6	145.9	221.0	16.3	1276.9

N = number of subjects with available results
GMTs calculated on all subjects
EU/mL: ELISA units per mL
n/% = number/percentage of subjects with titer within the specified range
95% CI = 95% confidence interval; LL = Lower Limit; UL = Upper Limit
MIN/MAX = Minimum/Maximum
PRE = pre-vaccination
PIII (M3) = Post dose 3 Month 3

Table 20 Ratios of post-vaccination anti-CS GMT one month Post Dose 3 between recipients of RTS,S/AS02A and recipients of RTS,S/AS02D (ATP cohort for immunogenicity)

Antibody	RTS,S/AS02A		RTS,S/AS02D		Ratio (Group A/Group D)		
	N	GMT	N	GMT	GMT ratio	95% CI	
						LL	UL
ANTI-CS	76	179.6	67	190.9	0.9	0.7	1.3

N: Number of subjects with available results
95% CI LL, UL: 95% lower and upper confidence interval limits (ANOVA model)

All subjects were seroprotected with respect to anti-HBs after vaccination, as shown in Table 21, and GMTs exceeded 17 000 mIU/mL in both groups. The RTS,S/AS02A: RTS,S/AS02D ratio of post-vaccination anti-HBs GMTs (Table 22) was 0.7 (with, as upper limit of 95% CI:1.1, which is below the *a priori* defined clinical limit of non-inferiority, 3).

Table 21 Seroprotection rates and geometric mean titers (GMT) for anti-HBs antibody titers one month after dose 3 (ATP cohort for immunogenicity)

Group	N	≥ 10 mIU/ML				GMT			MIN	MAX
		n	%	95% CI		Value	95% CI			
				LL	UL		LL	UL		
RTS,S/AS02D	67	67	100.0	94.6	100.0	23977.6	17895.5	32126.9	984.9	446880.0
RTS,S/AS02A	76	76	100.0	95.3	100.0	17410.0	13322.3	22751.9	1264.5	823500.0

N = number of subjects with available results
GMTs calculated on all subjects
n/% = number/percentage of subjects with titer within the specified range
95% CI = 95% confidence interval; LL = Lower Limit; UL = Upper Limit
MIN/MAX = Minimum/Maximum
PRE = pre-vaccination
PIII (M3) = Post dose 3 Month 3

Table 22 Ratios of post-vaccination anti-HBs GMT one month post Dose 3 between recipients of RTS,S/AS02A and recipients of RTS,S/AS02D (ATP cohort for immunogenicity)

Antibody	RTS,S/AS02A		RTS,S/AS02D		Ratio (Group A/Group D)		
	N	GMT	N	GMT	GMT ratio	95% CI	
						LL	UL
Anti-HBs	76	17 410.0	67	23 977.6	0.7	0.5	1.1

N: Number of subjects with available results
95% CI LL, UL: 95% lower and upper confidence interval limits (ANOVA model)

1.3.3.2.4. Studies of RTS,S/AS02A in children; conclusions

Following demonstration of the safety and efficacy of RTS,S/AS02A in adults, assessment of the RTS,S/AS02A vaccine progressed into children. Studies commenced with dose selection studies in older children, before progressing to dose selection studies in younger children. The RTS,S/AS02A vaccine was shown to be safe and immunogenic in both age groups at all dose levels. Subsequent to the selection of a suitable dose (0.25 mL containing 0.25 µg of antigen) and schedule (0, 1 and 2 month) with these studies, assessment of safety and efficacy progressed further with a study in approximately 2000 1 to 4 year old volunteers. The vaccine was shown to be safe, immunogenic and efficacious.

Following this study, a bridging study to evaluate the safety and immunogenicity of a new formulation of the vaccine, RTS,S/AS02D (designed to be compatible with EPI vaccination) was carried out. The new formulation was found to have an acceptable safety and reactogenicity profile and to induce similar antibody titers for anti-CS and anti-HBs as RTS,S/AS02A.

1.3.4. The RTS,S/AS01B candidate malaria and hepatitis B vaccine; data collected to date

1.3.4.1. Trials of RTS,S/AS01B in adults

The first clinical study of RTS,S adjuvanted with AS01B was initiated in healthy, malaria-naive adults, aged 18 to 45 years at WRAIR, Silver Spring, MD, USA. This study (Malaria-027) was a double-blind, randomized, controlled challenge, Phase I/IIa study involving two sequential cohorts, Cohort 1 (n=50) and Cohort 2 (n=50). Volunteers received 3 doses of either RTS,S/AS02A or RTS,S/AS01B vaccine at months 0, 1, 2, followed by a challenge with *P. falciparum*-infected mosquitoes 14 to 28 days post Dose 3 and rechallenge in protected subjects at approximately 6 months post Dose 3. Infectivity controls were enrolled for the challenge phases of the study. As of March 2005, Cohort 1 has completed all study procedures and Cohort 2 is ongoing.

To date, results are available for an interim analysis of safety (solicited and unsolicited events only), immunogenicity and efficacy data of Cohort 1 only.

The overall incidence of solicited and unsolicited AEs was similar in the RTS,S/AS02A and RTS,S/AS01B groups (per dose: 91.9% and 88.2%; per subject: 96.0% and 92.3%,

respectively). Grade 3 events tended to be more frequent in subjects receiving RTS,S/AS02A than those receiving RTS,S/AS01B (per dose: 28.4% and 17.6%; per subject: 48.0% and 30.8%, respectively).

The incidence of solicited local events recorded over the 6-day follow-up following each dose were slightly higher in the RTS,S/AS02A group compared to the RTS,S/AS01B group (Table 23). Grade 3 events of pain were observed to be more frequent in the RTS,S/AS02A group (after 14.9% of doses) than in the RTS,S/AS01B group (after 2.9% of doses). Grade 3 events of redness and swelling occurred with a similar and low frequency in both group.

The incidence of solicited general symptoms in the RTS,S/AS01B group was similar to that observed in the RTS,S/AS02A group (Table 23). Grade 3 events were infrequent, occurring in no more than 3 subjects in either group.

Unsolicited events occurred more frequently in the RTS,S/AS02A group (after 66.2% of doses) compared to the RTS,S/AS01B group. Grade 3 unsolicited events were rare, occurring after 6.8% of doses of RTS,S/AS02A and 4.4% of doses of RTS,S/AS01B. Two events in each vaccine group were graded 3 in intensity and were considered to be causally related to vaccine (RTS,S/AS02A: dizziness and insomnia; RTS,S/AS01B: chills and hyperhidrosis).

Two SAEs were reported by two subjects; the vaccine groups for both subjects have not been unblinded. One subject, a 42-year-old female developed gait disorder, vertigo, ill-defined condition, progressive weakness and facial droop 99 days after Dose 3 for which she was subsequently hospitalized. A subcortical infarct within the left internal capsule with a non-embolic distribution was diagnosed. Following examination by a neurologist, the cerebral infarct was considered likely to be associated with the use of oral contraceptives. The subject recovered with sequelae. A second subject was hospitalized for a ruptured tendon which occurred 107 days after Dose 3. The second subject, Neither SAEs were considered to have a causal relationship to study drug.

Table 23 Frequency of solicited symptoms per dose during the 6-day follow-up period for each vaccine group (Total Cohort, Malaria-027)

		RTS,S/AS02A		RTS,S/AS01B	
		N=74		N=68	
		n	%	n	%
Injection site pain	Any	60	81.1	53	77.9
	Grade 3	11	14.9	2	2.9
Injection site swelling	Any	19	25.7	11	16.2
	Grade 3	0	0.0	2	2.9
Injection site redness	Any	23	31.1	20	29.4
	Grade 3	1	1.4	4	5.9
Arthralgia	Any	10	13.5	14	20.6
	Grade 3	0	0.0	1	1.5
	Grade 3/Rel	0	0.0	1	1.5
Fatigue	Any	37	50.0	25	36.8
	Grade 3	3	4.1	3	4.4
	Grade 3/Rel	2	2.7	3	4.4
Gastrointestinal symptoms	Any	12	16.2	14	20.6
	Grade 3	3	4.1	0	0.0
	Grade 3/Rel	1	1.4	0	0.0
Headache	Any	28	37.8	22	32.4
	Grade 3	1	1.4	2	2.9
	Grade 3/Rel	0	0.0	2	2.9
Malaise	Any	27	36.5	22	32.4
	Grade 3	3	4.1	1	1.5
	Grade 3/Rel	2	2.7	1	1.5
Myalgia	Any	19	25.7	15	22.1
	Grade 3	0	0.0	2	2.9
	Grade 3/Rel	0	0.0	2	2.9
Fever	Any	10	13.5	12	17.6
	Grade 3	0	0.0	0	0.0
	Grade 3/Rel	0	0.0	0	0.0

N = number of subjects with available results
n/%: number/(percentage) of subjects reporting specified symptom
Grade 3 symptoms are those that prevent normal daily activity
Rel: related to study vaccine

At 28 days post dose 1, at least 95% of subjects were seropositive for anti-CS antibody titers in both the RTS,S/AS02A and RTS,S/AS01B vaccine groups (Table 24); all subjects in both groups were seropositive 56 days post dose 2 and remained so until 5 months post dose 3. GMTs for anti-CS antibodies tended to be higher at all time points in the RTS,S/AS01B group compared to the RTS,S/AS02A group.

At 28 days post dose 1, 68% of subjects in the RTS,S/AS02A group compared to 91% of subjects in the RTS,S/AS01B group had seroprotective anti-HBs antibody titers (Table 25); all subjects in both groups were seroprotected 56 days post dose 2 and remained so until 5 months post dose 3. GMTs for anti-HBs antibodies tended to be higher at all time points in the RTS,S/AS01B group compared to the RTS,S/AS02A group

Table 24 Seropositivity rates and geometric mean titers (GMT) for anti-CS antibody titers (ATP cohort)

Group	Timing	N	≥ 1 µg/mL				GMT			MIN	MAX
			n	%	95% CI		EU/mL	95% CI			
					LL	UL		LL	UL		
**RTS,S/AS02A**	PRE	25	0	0	0	13.7	0.5	0.5	0.5	<1.0	<1.0
	PI(D28)	25	24	96.0	79.6	99.9	10.4	5.8	18.6	<1.0	77.0
	PII(D56)	24	24	100	85.8	100	29.1	21.8	38.9	5.0	78.0
	DOC	24	24	100	85.8	100	97	73.8	127.5	25.0	256.0
	PIII(M5)	22	22	100	84.6	100	45.4	30.3	68.2	7.0	410.0
**RTS,S/AS01B**	PRE	26	2	7.7	0.9	25.1	0.5	0.5	0.6	<1.0	1.0
	PI(D28)	22	21	95.5	77.2	99.9	8.3	4.3	15.8	<1.0	99.0
	PII(D56)	20	20	100	83.2	100	71.1	44.6	113.2	10.0	990.0
	DOC	17	17	100	80.5	100	177.7	109.3	288.9	36.0	990.0
	PIII(M5)	15	15	100	78.2	100	73.7	41.1	132.2	11.0	440.0

N = number of subjects with available results
GMTs calculated on all subjects
n/(%) = number/(percentage) of subjects with anti-CS antibody titer within the specified range
95% CI = 95% confidence interval; LL = Lower Limit; UL = Upper Limit
MIN/MAX = Minimum/Maximum; PRE = pre-vaccination; PI(D28) = Post Dose 1 Day 28; PII(D56) = Post Dose 2 Month 3; DOC = Day of Challenge; PIII(M5) = Post Dose 3 Month 5.

Table 25 Seroprotection rates and geometric mean titers (GMT) for anti-HBs antibody titers (ATP cohort)

Group	Timing	N	≥ 10 MIU/mL				GMT			MIN	MAX
			n	%	95% CI		EU/mL	95% CI			
					LL	UL		LL	UL		
**RTS,S/AS02A**	PRE	25	8	32.0	14.9	53.5	12.5	3.6	43.8	<3.3	48190
	PI(D28)	25	17	68.0	46.5	85.1	819.6	92.0	7299.0	<3.3	4977000
	PII(D56)	24	24	100.0	85.8	100	6888.0	1759.5	26964.8	177.3	1900000
	DOC	24	24	100.0	85.8	100	31327.3	12843.7	76410.8	1265.2	1464300
	PIII(M5)	23	23	100.0	85.2	100	20637.8	9672.5	44034.0	1357.5	554000
**RTS,S/AS01B**	PRE	26	12	46.2	26.6	66.6	13.4	4.7	37.5	<3.3	7221
	PI(D28)	22	20	90.9	70.8	98.9	4424.7	593.7	32979.2	<3.3	3333000
	PII(D56)	20	20	100.0	83.2	100	31741.1	8205.2	122787	197.0	2495000
	DOC	17	17	100.0	80.5	100	52121.4	17874.0	151988	996.7	2277000
	PIII(M5)	14	14	100.0	76.8	100	25293.1	7671.1	83396.3	1284.0	871900

N = number of subjects with available results
GMTs calculated on all subjects
n/(%) = number/(percentage) of subjects with anti-HBs antibody titer within the specified range
95% CI = 95% confidence interval; LL = Lower Limit; UL = Upper Limit
MIN/MAX = Minimum/Maximum; PRE = pre-vaccination; PI(D28) = Post Dose 1 Day 28; PII(D56) = Post Dose 2 Month 3; DOC = Day of Challenge; PIII(M5) = Post Dose 3 Month 5.

Following challenge with *P. falciparum* 14 to 28 days after vaccination, 15/24 subjects (62.5%) in the RTS,S/AS02A group and 7/17 subjects (41.2%) in the RTS,S/AS01B group were infected (Table 26). All infectivity control subjects became infected.

Vaccine Efficacy (VE) compared to infectivity control was significant in both vaccine groups (P<0.001). The observed VE was higher in the RTS,S/AS01B group than in the RTS,S/AS02A group (58.8% [95% CI: 32.1, 81.6] vs 37.5% [95% CI: 11.8, 59.4], respectively), although this was not statistically significant (p=0.2085). The ratio of VE for RTS,S/AS01B to RTS,S/AS02A was 1.57.

In the unprotected individuals, the mean time to infection was similar in both groups (13.9 days [range 11 to 19 days] RTS,S/AS02A vs 13.4 days [range 12 to 15 days] RTS,S/AS01B) and longer than in the infectivity control (10.6 days [range 9 to 12 days]).

Table 26 Malaria-027; interim efficacy results, March 2005

		RTS,S/AS02A			RTS,S/AS01B			Control
		n/%	95% CI		n/%	95% CI		n/%
			LL	UL		LL	UL	
Number Challenged		24			17			12
Number Infected		15			7			12
Percentage Infected		62.5			41.2			100.0
Time to Infection	Minimum	11			12			9
	Median	14			14			11
	Mean	13.9			13.4			10.6
	Maximum	19			15			12
VE		37.5	11.8	59.4	58.8	32.1	81.6	--

VE: Vaccine Efficacy LL=95% lower limit
CI: confidence interval UL=95% upper limit

1.3.5. Concurrent protection against hepatitis B

The hepatitis B surface antigen (HBsAg) contained in the RTS,S candidate malaria and hepatitis B vaccine is encoded by the hepatitis B virus S protein gene that is identical to the gene used to express HBsAg in GSK Biologicals' Engerix-B vaccine against hepatitis B.

Non-inferiority of the anti-HBs response of RTS,S/AS02A compared to the licensed vaccine Engerix-B has been formally demonstrated in the study Malaria-026 (refer to Section 1.3.3.2.2).

1.4. Rationale for the study design

Preclinical studies and an ongoing Phase IIa study of RTS,S (Malaria-027) suggest that AS01B efficacy may be superior to that of AS02A. Efficacy of RTS,S/AS01B needs to be further evaluated as well as assurance of safety of the vaccine.

This study advances the clinical development of the RTS,S/AS01B vaccine to malaria endemic populations. As a primary objective it compares the safety and reactogenicity of RTS,S/AS01B to the RTS,S/AS02A vaccine which has been more extensively evaluated. As there is limited previous experience of the RTS,S/AS01B vaccine, the sample size of the study is limited for safety reasons.

As a secondary objective the study will establish proof of concept of efficacy of RTS,S/AS01B under conditions of natural exposure in an endemic country. Point estimates of efficacy will be determined for both RTS,S/AS01B and RTS,S/AS02A by comparing the time to first infection in the recipients of the experimental vaccines to controls. Under the anticipated malaria transmission intensity, the study has at least 80% power to detect a difference from control if the true vaccine efficacy is greater than or equal to 41%.

1.4.1. Safety monitoring plan

The safety monitoring plan for this trial is facilitated by the Local Safety Monitor (LSM); the role of the LSM is fully explained in 5.1.3.1. The LSM acts as an advocate for the study volunteers, advisor to the PI on clinical safety issues and a link between the study site, medical monitor and the Safety Monitoring Group (fully explained in 5.1.3.2). Furthermore, the LSM is empowered to temporarily suspend vaccination on the trial for major safety concerns, pending discussion with the SMG.

The SMG is empowered to temporarily suspend the trial on the basis of any safety concern or upon meeting predefined criteria for temporary suspension (Section 5.1.3.4). The SMG will review summarized safety and reactogenicity data at protocol-defined timepoints, so that the trial can be halted in a timely fashion if a safety issue is detected (refer to Section 5.1.3.2).

The full safety monitoring plan process is detailed in Section 5.1.3.

1.4.2. Assessment of proof-of-concept

Proof-of-concept will be assessed as time-delay to first infection with asexual forms of *P. falciparum*.

The time to infection was assessed in the challenge model conducted at the WRAIR; estimates of vaccine efficacy with RTS,S/AS02A were consistently approximately 40% [Kester, 2001; Stoute, 1997]. When the vaccine was evaluated under conditions of natural exposure in The Gambia [Malaria-005], the vaccine efficacy for the time to first infection in adults was 34% (CI 8.0% to 53.0%). The recently conducted Phase 2b trial in Mozambican children (Malaria-026) was the first study in which both clinical and parasitological endpoints were assessed. Measured concurrently in two groups of children, vaccine efficacy against first clinical episode was 30% (95% CI: 11.0% to 44.8%) and against first infection was 45% (95% CI: 31.4% to 55.9%).

Time to first infection will be used to assess proof-of-concept, because it has proven to be consistent when measured by different methodologies in different populations and because, in the recent Malaria-026 study, the point estimate of efficacy against infection was consistent with the estimate of prevention of clinical episodes.

1.4.3. Rationale for the use of Rabies vaccine as a control

Rabies vaccine has been chosen as the comparator because: 1) the vaccine can be administered on a 0, 1, 2 month schedule; 2) rabies vaccine has been used in previous studies of RTS,S-based vaccines in Africa; and 3) volunteers will benefit from receiving rabies vaccine as rabid animals occur in the study area.

Chiron's Rabipur® vaccine will be used. When the Rabipur rabies vaccine is administered according to the recommended vaccination schedule (days 0, 7, 21), nearly 100% of subjects attain a protective titer. In two studies carried out in the US in 101 subjects, protective antibody titers >0.5 IU/mL were obtained by day 28 in all subjects. In studies

carried out in Thailand in 22 subjects, and in Croatia in 25 subjects, antibody titers of >0.5 IU/mL were obtained by day 14 (injections on days 0, 7, 21) in all subjects [Dreesen, 1989; Nicholson, 1987; Vodopija, 1986; Wasi, 1986].

High antibody titers have also been demonstrated with off-label immunization with rabies vaccines. Among participants in England, Germany, France and Belgium who received two vaccinations one month apart, nearly 100% of the participants developed specific antibody and the geometric mean titer for the group was 10 IU [Ajjan, 1978; Costy-Berger, 1978; Cox, 1976; Kuwert, 1978]. The proposed vaccination schedule of 0, 1, and 2 months is therefore expected to be highly successful in conferring protective immunity against rabies among the control participants. However all subjects that are in contact with a potential rabid animal will be advised to seek medical attention immediately.

1.5. Rationale for testing cell-mediated immunity

RTS,S/AS02A has been shown to be a powerful inducer of antigen-specific humoral and CMI response in preclinical and clinical studies [GSK data on file, Malaria Investigator's Brochure 2003]. The RTS,S/AS02A and RTS,S/AS01B vaccines are believed to elicit a strong humoral immune response directed against surface-exposed sporozoite proteins, and elicit CMI responses characterized by predominantly CD4+—Th1 cells, that are hypothesized to either destroy infected hepatocytes and/or limit intracellular parasite development through appropriate cytokines. As CMI response is believed to be a key component in protecting vaccinees against the *P. falciparum* parasite it will be measured in this trial and exploratory analyses conducted.

1.6. Rationale for Parasite Genotyping evaluation

It is proposed to carry out an ancillary study in collaboration with the London School of Hygiene and Tropical Medicine (LSHTM) to use molecular genetic typing methods to determine two important parasitological parameters a) if there is detectable strain-specificity in the effect of the vaccine b) if vaccination alters the multiplicity of subsequent infections. Both these parameters will be examined in both incident and prevalent infections.

The CSP is the predominant protein found in the surface of the sporozoite. Studies of the genetic diversity of the gene encoding the CSP of P. falciparum have demonstrated the existence of high levels of genetic polymorphisms within the region known to contain T-cell epitopes in isolates from different areas in Africa [Escalante, 2002], which is therefore potentially important for generation of immunity. This has raised the concern whether a vaccine containing a sequence of a selected strain of P. falciparum would confer protection against other variant parasites. This high number of alleles should be considered when testing the efficacy of CSP-based vaccines. In the Gambia, the RTS,S/AS02A vaccine was demonstrated to induce protection against first P. falciparum infections that was not strain-specific. The frequency of the vaccine allele type, which was derived from the sequence of the CSP allele in laboratory clone 3D7, was similar to that of all other CSP alleles studied in the vaccine vs control group [Alloueche, 2003]. The key polymorphic sites in the CSP gene which are encompassed by the RTS,S antigen are the T-cell epitopes at the carboxy-terminus of the protein,

designated Th2R and Th3R. Polymorphisms in these epitopes were the focus of the evaluation of strain-specificity of vaccine efficacy in the Gambian trial [Alloueche, 2003]. Evidence was also found in that study that RTS,S modified the average number of clones (multiplicity) carried during post-vaccination parasitemia. Modification of multiplicity of P. falciparum infections was also found after vaccination with the chimaeric peptide multimer vaccine SPf66 [Haywood, 1999]. These findings were confirmed by recent analysis of CSP sequences and clone multiplicity in 521 parasite isolates from Mozambiquan children who participated in a large Phase II study of the efficacy of RTS,S/AS02A [Alonso, 2004; Enosse, 2006].

To evaluate strain-specific vaccine effects in this trial of the RTS,S/AS02A and RTS,S/AS01B vaccines in Kenyan adults, DNA samples will be used for sequence analysis of the Th2R and Th3R sequences in the CSP gene of parasitemic individuals identified during follow-up by active detection of infections (ADI). Vaccine effects on multiplicity of infection (MOI) will also be investigated using polymorphic antigens loci, namely msp1 and msp2.

Amended 13 June 2006

1.7. HIV testing at screening

As part of this trial, no HIV testing is scheduled to be carried out at screening. It is a condition of the trial that all enrolled subjects are in good health (refer to Section 4.3), as confirmed by a range of clinical and blood tests at screening (refer to Section 4.4 for the exclusion criteria for this trial). Those volunteers who exhibit clinical features of immunosuppresion or immunodeficiency will not be enrolled (refer to Section 4.4). There is no reason to believe that any of the vaccines to be administered during this study may have any ill-effect on clinically asymptomatic volunteers infected with HIV.

In addition, HIV testing has been found to be culturally unacceptable at the site. The local community have hesitated to embrace HIV testing and local elders have voiced their concerns about mandatory HIV testing as part of any studies in this community.

1.8. Risks and benefits for study volunteers

1.8.1. Risks of receipt of RTS,S/AS02A, RTS,S/AS01B or Rabies Vaccine and risk-management

Recipients of any of the investigational products in this study (RTS,S/AS02A, RTS,S/AS01B or Rabies Vaccine) may experience pain and/or swelling at the injection site, fever, headache, fatigue, nausea, vomiting and/or abdominal pain, joint pain and/or muscle aches. As with any vaccination, there is the potential for an anaphylactic reaction. Additionally, there may other reactions that at this time are not known.

To facilitate management of these potential problems, medical staff experienced in the management of anaphylactic reaction will observe patients for at least 30 minutes following each vaccination to ensure that should any anaphylactic reaction occur it is

managed in a timely manner (refer to Section 5.3). Subjects will be followed up daily by study staff for 6 days following each dose of investigational product in order to evaluate and arrange treatment (where necessary) of any post-vaccination symptoms (refer to Section 5.4).

1.8.2. Risk of receipt of Malarone and risk management

Subjects who receive Malarone® may experience stomach pain, headache, nausea, vomiting, loss of appetite, cough, rash, tiredness, mouth ulcers, hair loss or diarrhea.

Subjects will be seen by field workers on the day following Dose 1 and Dose 2 of Malarone®, and by a study clinician one week post Dose 3 of Malarone®. Each dose of Malarone® will be given under the supervision of a fieldworker. Any subjects who are unwell at these visits will be referred to a study clinician for evaluation and treatment. Subjects who are unable to tolerate Malarone® will be given Coartem® (refer to Section 5.10).

1.8.3. Risk of receipt of Coartem and risk management

Subjects who receive Coartem® may experience abdominal pain, anorexia, nausea, vomiting, diarrhea, headache and/or dizziness.

For those subjects who receive Coartem® as an alternative clearance treatment, they will be seen by field workers on the day following the first two doses and the second two doses of Coartem, and by a study clinician one week post the final two doses of Coartem®. Dose 1, Dose 3 and Dose 5 of Coartem® will be given under the supervision of a fieldworker. Any subjects who are unwell at these visits will be referred to a study clinician for evaluation and treatment.

1.8.4. Risk of disclosure of volunteer's confidential/personal information to persons not involved in the study

In order to ensure that all information collected on study volunteers is kept confidential, the following safeguards will be applied:

- Access to study files and personal information will be limited to only WRP and GlaxoSmithKline personnel with a need to access the information.
- Study information will be kept in locked cabinets when not in use.
- All information or samples that leave WRP will be labeled in a way that it cannot be linked back to a specific study subject.

1.8.5. Benefits to study volunteers of taking part in the study

All volunteers for this study will receive the following benefits for their participation:

- All volunteers will undergo a medical examination at screening free of charge. All volunteers, whether accepted for enrollment into the trial or not will benefit from this

free health check-up. The results of all tests will be communicated to all volunteers. Where illnesses are newly-diagnosed, a referral to an appropriate specialist will be made for the volunteer.

- For the duration of their participation in the trial, all randomized subjects will receive free health care from study medical personnel.
- All enrolled subjects will be offered, free of charge, a full three dose course of vaccinations against rabies (except where the principal investigator deems that vaccination is unsuitable for safety concerns). Subjects who are randomized to receive the rabies control vaccine will receive these vaccinations during the course of the trial. Subjects who are randomized to receive RTS,S/AS01B or RTS,S/AS02A will be offered the vaccination course after they have completed all study procedures and returned for the final study visit.

2. OBJECTIVES

2.1. Primary Objective

2.1.1. Safety & Reactogenicity

- To compare the safety and reactogenicity of RTS,S/AS01B vs RTS,S/AS02A, when administered as 3 doses intramuscularly on a 0, 1 and 2 months schedule to adult volunteers living in a malaria endemic region

Refer to Section 10.1 for definition of the primary endpoint.

2.2. Secondary Objectives

2.2.1. Safety

- To describe the safety and reactogenicity of RTS,S/AS02A and RTS,S/AS01B, when administered as 3 doses intramuscularly on a 0, 1 and 2 months schedule to adult volunteers living in a malaria endemic region.

2.2.2. Immunogenicity

- To describe antibody responses to the circumsporozoite (anti-CS) antigen and hepatitis B surface antigen (anti-HBs) of RTS,S/AS02A and RTS,S/AS01B when administered as 3 doses intramuscularly on a 0, 1 and 2 months schedule to adult volunteers living in a malaria endemic region.

2.2.3. Proof-of-concept

- To assess the efficacy of RTS,S/AS02A and RTS,S/AS01B against infection with *P. falciparum* malaria (defined as *P. falciparum* asexual parasitemia > 0) over a period of 14 weeks post Dose 3, in adults under conditions of natural transmission

Refer to section 10.2 for definition of the secondary endpoints.

2.3. Tertiary Objective

2.3.1. Safety

- To describe the long-term safety of RTS,S/AS02A and RTS,S/AS01B until 12 months post Dose 1.

Refer to Section 10.3 for definition of the tertiary endpoints.

2.4. Exploratory Objectives

2.4.1. Parasite Genotyping

- ***To determine the prevalence of vaccine-like alleles of CSP in the study population.***
- ***To investigate whether vaccination with RTS,S/AS0A2 and RTS,S/AS01B modifies the distribution of amino acid substitutions in the Th2R and Th3R sequence regions of the CSP gene in subsequent P. falciparum infections.***
- ***To investigate whether vaccination with RTS,S/AS0A2 and RTS,S/AS01B modifies the number of P. falciparum genotypes (multiplicity of infection) as determined by analysis of msp-1 and msp-2 genes.***

Refer to Section 10.4 for definition of the exploratory endpoints.

Amended 13 June 2006

3. STUDY DESIGN OVERVIEW

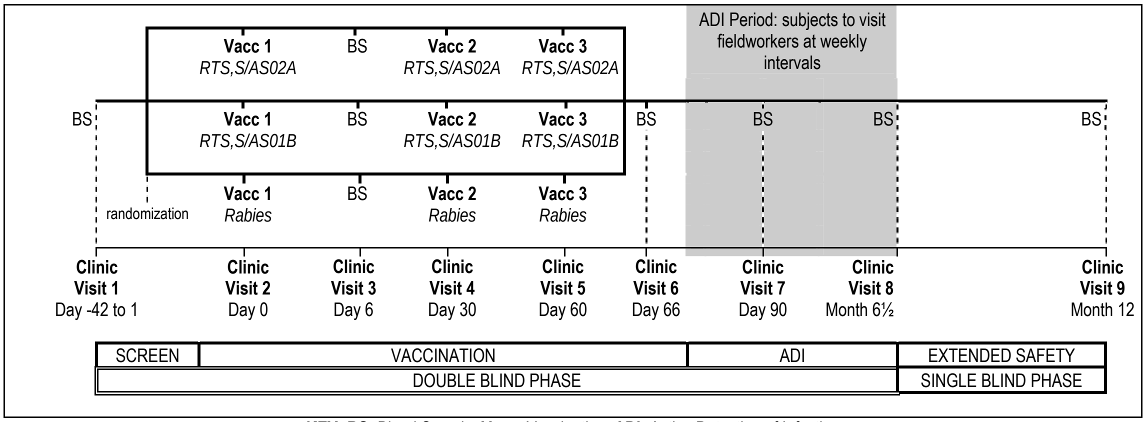

KEY: BS; Blood Sample. **Vacc;** Vaccination. **ADI;** Active Detection of Infection

- Phase IIb, single-center, double blind (observer blind, participant blind), randomized controlled trial with three groups in one study site (randomization 1:1:1, RTS,S/AS01B: RTS,S/AS02A:rabies)
- 255 adults will be enrolled. It is assumed that 90% will complete study procedures and be evaluable.

- Prior to study start, a community information program will inform the local population of the study. Throughout the period of enrollment, study information will be presented at community centers.
- Healthy male and female adults aged 18 to 35 years will be screened. Those determined to be eligible, based on the inclusion and exclusion criteria, will be enrolled in the study.
- Route of administration: all vaccines will be administered by the intramuscular route to the deltoid of the non-dominant arm
- Each subject will be observed for at least 30 minutes after vaccination to evaluate and treat any acute adverse events
- After vaccination, the subjects will visit field workers at field stations for the detection of adverse events (AEs) occurring after vaccination for one week (days 1, 2, 3, 4, 5 and day 6 after each vaccination). Diary cards and thermometers will be provided for the field workers to record oral temperature and any local (at the injection site) or general adverse events.
- One week prior to Dose 3, subjects will be treated with Malarone® for presumptive clearance of malaria parasitemia (in the event that subjects are unable to tolerate Malarone®, Coartem® (artemether/lumefantrine, Novartis AG, Zurich, Switzerland) will be used as the alternative clearance treatment.
- Surveillance for Active Detection of Infection (ADI) will begin 2 weeks after Dose 3. Subjects will visit field workers at field-stations near their homes weekly during the ADI period. The total period of surveillance for ADI is 14 weeks. Two Clinic Visits and 14 field-worker visits will contribute to the ADI.
- All subjects will have a blood slide prepared and read to check for asexual *P. falciparum* parasitemia one week post Dose 3. Any subject who tests positive will be treated with Coartem®
- At each contact for ADI, history of fever will be recorded and oral temperature taken; a smear will be taken for detection of malaria parasites.
- Infection with malaria parasites is defined as *P. falciparum* asexual parasitemia > 0
- Subjects who are symptomatic at the time of ADI contact (i.e. history of fever within previous 24 hours or oral temperature >37.5°C) will have a blood slide read and treated within the same day if the blood slide is positive.
- Subjects will be followed passively for the occurrence of clinical malaria from Day 0 until 16 weeks post Dose 3. Cases will be detected at Kombewa Clinic. If a subject feels ill they can present to a field station near their home and be transported to Kombewa Clinic for evaluation.
- ***DNA will be extracted from EDTA blood of all subjects with patent asexual parasitemia.*** **Amended 13 June 2006**
- There will be a 30 day follow-up period after each dose of vaccine for reporting unsolicited symptoms

- Recording of serious adverse events will be throughout the study period. They will be captured through the morbidity surveillance system at Kombewa Clinic.
- Anti-CS antibody titers will be determined at baseline, one month post Dose 2, 1 month post Dose 2, 1 month post Dose 3, 4 months post Dose 3 and 10 months post Dose 3.
- Anti-HBs antibody titers will be determined at baseline, one month post Dose 3 and 10 months post Dose 3.
- CS-specific CMI will be assessed at ~~***three***~~ ***four*** time points during the study; prevaccination, 1 month post Dose 3, ~~***and***~~ 4 months post Dose 3 ***and 9 months post Dose 3***. **Amended 13 June 2006.**
- Data collection will be by conventional CRF.
- Duration of the study will be approximately 12 months per subject.
- There will be a planned interim analysis on all safety humoral and immunogenicity data collected up to 30 days after Dose 3.
- The final analysis of the study for all primary and secondary endpoints will take place on all data collected up to the end of the surveillance for ADI (16 weeks post Dose 3).
- All subjects will be followed for safety for a period that extends to 10 months post Dose 3. Any data collected after the final analysis will be reported in an annex report.

4. STUDY COHORT & STUDY SITE

4.1. Study Site

4.1.1. Kenya Medical Research Institute and Walter Reed Project Kombewa Clinic, Western Kenya

The Walter Reed Project Kombewa Clinic (KC) is a clinical trials center developed in collaboration with the US Army Medical Research Unit, Kenya; Kenya Medical Research Institute, KEMRI, and other non-government partners for the study of malaria vaccines and drugs. The clinic consists of a malaria microscopy section, clinical laboratory, phlebotomy room, pharmacy, an outpatient evaluation center with six examination/consultation rooms, an urgent-care treatment room, and a four-bed observation suite. It also has a dedicated data-entry section, an active-records room, a records archive, offices for clinical officers and clinical research coordinators, on-call rooms and a general store. Additionally, recent construction of a two-story annex block now offers new office spaces, more commodious general storage areas, a secure records archive room, meeting rooms, and a library.

To facilitate accessibility to the study participants, field stations have been designated within Kombewa Division; all within one-mile radii of the homes of study subjects. As these field stations are intended to be points of first contact for sick study participants

they too are staffed 24 hours a day and equipped with communication systems contact KC to summon transportation. Twelve of these field stations will be utilized in this study. The field sites will be open until the end of the ADI period. After that, subjects will access study staff at KC.

4.1.2. Study site; geography

Kombewa Clinic is located about 40 kilometers west of Kisumu Town in the village of Kombam (near Kombewa Town), in Kombewa Division, Kisumu District, Nyanza Province, Kenya. Kombewa Division (the area from which study volunteers are recruited) runs from the northern edge of Winam Gulf on Lake Victoria to Meseno Division in the north.

There are two distinct rainy seasons. The "long rainy season" of late March through May produces intense transmission from April through August and the "short rainy season" of October through December produces another intense transmission season from November through January.

4.1.3. Study site; malaria transmission

Malaria transmission occurs all year but is maximal during the two rainy seasons. The principal vector is *Anopheles gambiae*. *P. falciparum* parasitemia is present in over 90% of malaria cases. *P. ovale* and *P. malariae*, which together constitute less than 10% of cases, are usually present in mixed infections with *P. falciparum.*

The Malaria Infection Rate in adults has been estimated to be been roughly 95% during the long rains (April to August) and 72% during the short rains (November to January) (unpublished data, WRAIR). Approximately 25% of infected adults become symptomatic.

A longitudinal epidemiological study conducted in 2003/2004 in Kombewa indicated that monthly attack rates range from approximately 20% to 55% in children aged 1 to 3 years in the proposed study area (unpublished data WRAIR) (Figure 1).

Figure 1 Malaria Attack Rates in Kombewa Division 2003-2004 (EPI study of 270 Children age 1-3 years)

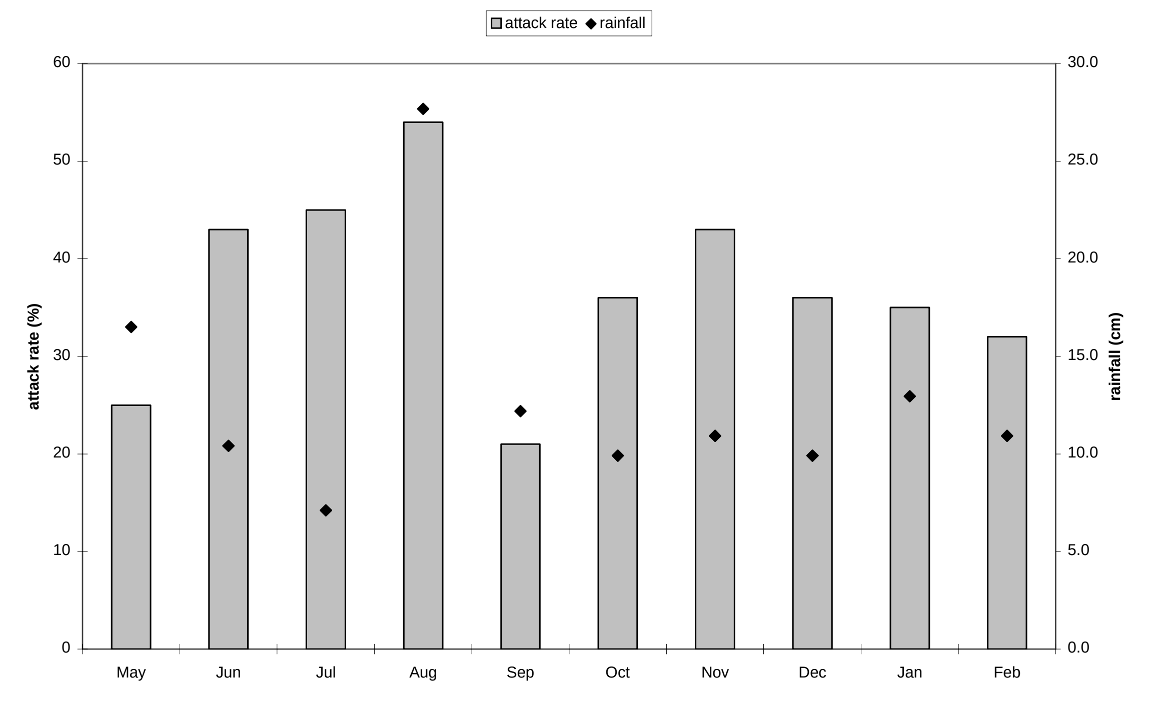

4.1.4. Study site; population

The population of Kombewa Division is primarily Luo. The majority of the population speaks both Dholuo and Kiswahili. Subsistence farming and related industries are the main activities in the area.

Villages in this area are typically a loose conglomeration of family compounds near a family garden plot and grazing land. Houses are typically made of mud with thatched or corrugated roofs. In Kombewa Town, homes are often attached to family shops/businesses. Most homes do not have glass windows or screens. Water source is mainly from community wells and local streams though some households have their own wells. Most water sources are not chlorinated or covered.

4.2. Number of subjects

Subjects will be recruited by non-coercive methods according to existing policies. Eligible subjects will be male and female adults between the ages of 18 and 35 years.

The necessary number of volunteers will be screened in order to enroll approximately 255 eligible subjects, 85 to each of the RTS,S/AS02A, RTS,S/AS01B groups and Rabies Vaccine groups.

4.3. Inclusion criteria

All subjects must satisfy the following criteria at study entry:

- A male or female of between 18 and 35 years of age at the time of first vaccination
- Written informed consent obtained from the subject
- Available to participate for the study duration (approximately 12 months)
- Free of obvious health problems as established by medical history and clinical examination before entering into the study.
- If the volunteer is female, she must be of non-childbearing potential, i.e. either surgically sterilized or one year post-menopausal; or, if of childbearing potential, she must be abstinent or have used adequate contraceptive precautions (i.e. natural family planning, intrauterine contraceptive device; oral contraceptives; diaphragm or condom in combination with contraceptive jelly, cream or foam; Norplant® or DepoProvera®) for 30 days prior to vaccination, have a negative pregnancy test and must agree to continue such precautions for two months after completion of the vaccination series.
- Negative pregnancy test

4.4. Exclusion criteria for enrollment

The following criteria should be checked at the time of study entry. If any apply, the subject must not be included in the study:

- Planned administration/administration of a vaccine not foreseen by the study protocol within 30 days of the first dose of vaccine(s) with the exception of tetanus toxoid.
- Any confirmed or suspected immunosuppressive or immunodeficient condition, including human immunodeficiency virus (HIV) infection.
- A family history of congenital or hereditary immunodeficiency.
- History of allergic reactions significant IgE-mediated events or anaphylaxis to previous immunizations.
- History of any neurologic disorders or seizures.
- Acute disease at the time of enrolment (acute disease is defined as the presence of a moderate or severe illness with or without fever). All vaccines can be administered to persons with a minor illness, such as diarrhoea or mild upper respiratory infection without fever, i.e. Oral temperature < 37.5°C.
- Acute or chronic, clinically significant pulmonary, cardiovascular, hepatic or renal functional abnormality, as determined by physical examination or laboratory screening tests.
- ALT above normal range as defined in Table 32
- Creatinine above normal range as defined in Table 32

- Hemoglobin below normal range as defined in Table 33
- Platelet count below normal range as defined in Table 33
- Absolute lymphocyte count below normal range as defined in Table 33
- Total white cell count below normal range as defined in Table 33
- HBsAg positive
- Homozygous sickle cell disease (Hgb SS)
- History of splenectomy.
- Administration of immunoglobulins, blood transfusions or other blood products within the three months preceding the first dose of study vaccine or planned administration during the study period.
- Pregnant or lactating female (i.e. a positive pregnancy test).
- Female planning to become pregnant or planning to discontinue contraceptive precautions.
- History of chronic alcohol consumption and/or drug abuse.
- Use of any investigational or non-registered drug or vaccine within 30 days preceding the first dose of study vaccine, or planned use during the study period.
- Chronic administration (defined as more than 14 days) of immunosuppressants or other immune-modifying drugs within six months prior to the first vaccine dose (for corticosteroids, this will mean prednisone, or equivalent, ≥ 0.5 mg/kg/day. Inhaled and topical steroids are allowed).
- Any chronic drug therapy to be continued during the study period.
- Major congenital defects or serious chronic illness.
- Previous participation in any other malaria vaccine trial.
- Simultaneous participation in any other clinical trial.
- Any other findings that the investigator feels would increase the risk of having an adverse outcome from participation in the trial.

As part of the screening procedure for this trial, no HIV testing will be performed.

4.5. Elimination criteria during the study

The following criteria should be checked at each visit subsequent to the first visit. If any become applicable during the study, it will not require withdrawal of the subject from the study but may determine a subject's evaluability in the according-to-protocol (ATP) analysis. See Section 10.6 for definition of study cohorts to be evaluated.

- Use of any investigational or non-registered product (drug or vaccine) other than the study vaccine(s) during the study period.

- Chronic administration (defined as more than 14 days) of immunosuppressants or other immune-modifying drugs during the study period (for corticosteroids, this will mean prednisone, or equivalent, ≥ 0.5 mg/kg/day. Inhaled and topical steroids are allowed).
- Administration of a vaccine (except tetanus toxoid) not foreseen by the study protocol during the period starting from 90 days before Dose 1 and ending 30 days after Dose 3.
- Administration of immunoglobulins and/or any blood products during the study period.
- Drug or alcohol abuse.

4.6. Contraindications to subsequent vaccination

4.6.1. Absolute contraindications to further vaccination

The following adverse events (AEs) constitute absolute contraindications to further administration of RTS,S/AS02A, RTS,S/AS01B or Rabipur; if any of these AEs occur during the study, the subject must not receive additional doses of vaccine but may continue other study procedures at the discretion of the investigator (see Section 9). The subject must be followed until resolution of the event, as with any AE (see Section 8.7):

- Acute allergic reaction, significant IgE-mediated event or anaphylactic shock following the administration of vaccine investigational product
- Any confirmed or suspected immunosuppressive or immunodeficient condition, including human immunodeficiency virus (HIV) infection.
- Pregnancy, i.e. a positive urine test (see Section 8.11).

4.6.2. Indications for deferral of vaccination

The following AEs constitute contraindications to administration of RTS,S/AS02A, RTS,S/AS01B or Rabipur at that point in time. If any one of these AEs occurs at the time scheduled for administration of investigational product, the investigational product may be administered to the subject at a later date but within the time window specified in the protocol (see Section 5.12), or withdrawn at the discretion of the investigator (see Section 9). The subject must be followed until resolution of the event, as with any AE (see Section 8.7).

- Acute disease at the time of administration of investigational product (acute disease is defined as the presence of a moderate or severe illness with or without fever). All vaccines can be administered to persons with a minor illness such as diarrhoea or mild upper respiratory infection without fever, i.e. Oral temperature < 37.5°C
- Oral temperature of ≥ 37.5°C

5. CONDUCT OF STUDY

5.1. Ethics and regulatory considerations

The study will be conducted according to Good Clinical Practice (GCP), the appended Declaration of Helsinki (Protocol Appendix A), and local rules and regulations of the country.

The following two sections provide guidance to the study investigator of the minimal ethical and regulatory requirements required by GSK but the final responsibility for the interactions between the Institutional Review Boards (IRBs) and Independent Ethics Committees (IECs) and for informed consent remain with the Principal Investigator and may be subject to local rules and regulations.

5.1.1. Institutional Review Board/Independent Ethics Committee (IRB/IEC)

The IRB/IEC must be constituted according to the local laws/customs of each participating country. The ICH Harmonized Tripartite Guideline for Good Clinical Practice recommends that the IRB/IEC should include:

(a) At least five members.

(b) At least one member whose primary area of interest is in a non-scientific area.

(c) At least one member who is independent of the institution/study site.

Only those IRB/IEC members who are independent of the investigator and the study sponsor (USAMMDA), GSK Biologicals or WRAIR should vote/provide opinion on a study-related matter.

This protocol and any other documents that the IRB/IEC may need to fulfill its responsibilities, including subject recruitment procedures and information about payments and compensation available to subjects, will be submitted to the IRB/IEC by the principal investigator. Written unconditional approval of the IRB/IEC must be in the possession of the investigator and GSK Biologicals and USAMMDA before commencement of the study. This approval must refer to the study by exact protocol title and number, and should identify the documents reviewed and state the date of review. Relevant GSK Biologicals' data will be supplied by the principal investigator to the independent IRB/IEC for review and approval of the protocol. Verification of IRB/IEC unconditional approval of the protocol and the written informed consent statement will be transmitted by the principal investigator to GSK Biologicals' Study Monitor, using the standard notification form, prior to shipment of vaccine supplies and CRFs to the site.

No deviations from, or changes to, the protocol should be initiated without prior written consent of the sponsor, USAMMDA, or GSK Biologicals and IRB/IEC favorable opinion of an appropriate amendment or administrative change except when necessary to

eliminate immediate hazards to the subjects Approvals/verifications must be transmitted in writing to GSK Biologicals' Study Monitor by the principal investigator.

The IRB/IEC must be informed by GSK Biologicals' Study Monitor, of:

- all subsequent protocol amendments, informed consent changes or revisions of other documents originally submitted for review;
- serious and/or unexpected adverse events occurring during the study, where required,
- all subsequent protocol modifications (for information);
- new information that may affect adversely the safety of the subjects or the conduct of the study;
- an annual update and/or request for re-approval, where required;
- when the study has been completed, where required.

If a trial is prematurely terminated or suspended for reasons including, but not limited to, safety or ethical issues or severe non-compliance, the sponsor, USAMMDA will promptly inform the regulatory authorities of the termination or suspension and the reason(s) for the termination or suspension. If required by applicable regulations, the investigator must inform the IEC/IRB promptly and provide the reason for the suspension or termination (see Section 5.1.3 and Appendix B for further details).

5.1.2. Informed consent

The details of the informed consent process are provided in Appendix C. The following principles will also apply.

In obtaining and documenting informed consent, the investigator should comply with the applicable regulatory requirement(s), and should adhere to GCP and to the ethical principles that have their origin in the appended Declaration of Helsinki. Prior to the beginning of the trial, the investigator should have the IRB/IEC's written approval/favorable opinion of the written informed consent form and any other written information to be provided to the subjects.

Information should be given in both oral and written form whenever possible and as deemed appropriate by the IRB/IEC.

An investigator or designate will describe the protocol to potential subjects face to face. The Subject Information and Consent Form may be read to the subjects, but, in any event, the investigator or designate shall give the subjects ample opportunity to inquire about details of the study and ask any questions before dating and signing the Consent Form.

Subject information and consent forms must be in a language fully comprehensible to the prospective subjects. Informed consent shall be documented by the use of a written consent form approved by the IRB/IEC and signed and dated by the subjects and by the person who conducted the informed consent discussion. The signature confirms the consent is based on information that has been understood. All illiterate individuals will

have the study, the Subject Information and Consent Form explained to them point by point by the interviewer in the presence of an impartial witness. The subjects will thumbprint or sign the consent form. The witness will also sign and date the consent form. Oral witnessed consent will replace written consent only in countries where the local custom is contrary or if the subjects incapacity precludes this and provided that the local legal obligations are fulfilled.

Each subject's signed informed consent form must be kept on file by the investigator for possible inspection by Regulatory Authorities and/or GSK Biologicals' professional and Regulatory Compliance persons. Only the signed Informed Consent Agreement pages of each volunteer will be kept on file at the investigator's site. In addition one entire representative copy of the informed consent documentation will be kept on file by the investigator for reference. The subjects should receive a copy of the signed and dated written informed consent form and any other written information provided to the subjects and should receive copies of any signed and dated consent form updates. Any amendments to the written information will be provided to subjects.

Both the informed consent discussion and the written informed consent form and any other written information to be provided to the subjects should include explanations of the following:

(a) That the trial involves research.

(b) The purpose of the trial.

(c) The trial treatment(s) and the probability for random assignment to each treatment.

(d) The trial procedures to be followed, including all invasive procedures.

(e) The subject's responsibilities.

(f) Those aspects of the trial that are experimental.

(g) The reasonably foreseeable risks or inconveniences to the subjects and, when applicable, to an embryo, fetus or nursing infant.

(h) The reasonable expected benefits. When there is no intended clinical benefit to subjects, the subjects should be made aware of this.

(i) The alternative procedure(s) or course(s) of treatment/methods of prevention that may be available to subjects, and their important potential benefits and risks.

(j) The compensation and/or treatment available to subjects in the event of trial-related injury.

(k) The anticipated prorated payment, if any, to subjects for participating in the trial.

(l) The anticipated expenses, if any, to subjects for participating in the trial.

(m) That the subjects' participation in the trial is voluntary and subjects may refuse to participate or withdraw from the trial, at any time, without penalty or loss of benefits to which subjects are otherwise entitled.

(n) That the monitor(s), the auditor(s), the IRB/IEC, and the regulatory authority(ies) will be granted direct access to the subject's original medical records for verification of clinical trial procedures and/or data, without violating the confidentiality of subjects, to the extent permitted by the applicable laws and regulations and that, by signing a written informed consent, the subject's is authorizing such access.

(o) That records identifying subjects will be kept confidential and, to the extent permitted by the applicable laws and/or regulations, will not be made publicly available. If the results of the trial are published, subjects' identity will remain confidential.

(p) That the subjects will be informed in a timely manner if information becomes available that may be relevant to the subjects willingness for continued participation in the trial.

(q) The person(s) to contact for further information regarding the trial and the rights of trial subjects, and who to contact in the event of trial-related injury.

(r) The foreseeable circumstances and/or reasons under which a subject's participation in the trial may be terminated.

(s) The expected duration of a subject's participation in the trial.

(t) The approximate number of subjects involved in the trial.

GSK Biologicals will prepare a model Informed Consent Form which will embody all the elements described above. While it is strongly recommended that this model document be followed as closely as possible, the informed consent requirements given in this document are not intended to pre-empt any local regulations which require additional information to be disclosed for informed consent to be legally effective. Clinical judgment, local regulations and requirements should guide the final structure and content of the document.

The investigator has the final responsibility for the final presentation of Informed Consent Form, respecting the mandatory requirements of local regulations. The consent form generated by the investigator with the assistance of the sponsor's representative, must be approved (along with the protocol, and any other necessary documentation) by the IRB/IEC and be acceptable to GSK Biologicals.

5.1.3. Safety monitoring plan

A local safety monitor (LSM) will be in place for the trial.

A Safety Monitoring Group (SMG) comprising medical representation from KEMRI, the sponsor's representative (USAMMDA), GSK Biologicals and WRAIR will review data. (5.1.3.2)

The PI, LSM and SMG are empowered to suspend the trial for any safety concern. To supplement this, the protocol defines criteria for both the immediate suspension of all vaccination and also for the progression to the next sequential dose. Blinded safety reports will be produced by the Principal Investigator for the SMG on the first 60 subjects to receive Dose 1 and Dose 2 (5.1.3.3).

The decision making process for resuming vaccination or permanently stopping the trial is described in Section 5.1.3.4.

5.1.3.1. Local Safety Monitor (LSM)

The overall role of the LSM, who is an experienced clinician based in-country, will be to support the PI and to act as a link between the PI and the SMG (refer to Section 5.1.3.2).

The LSM's role will include:

- Acting as the study volunteer's advocate;
- Promptly communicating relevant safety information to the medical monitor and the SMG;
- Providing advice to the PI on whether a set of clinical circumstances in a study warrants formal notification to the medical monitor and the SMG;
- Unblinding a subject if deemed necessary to allow for adequate treatment;
- Suspension of vaccination for a major safety concern pending discussion with the SMG (see Section 5.1.3.4.1 for full details);

A second, qualifed, experienced physician based in-country will serves as the alternate LSM, in the event the LSM is out of the country or unavailable to fulfill time-sensitive duties.

The LSM is required to review all unanticipated problems (refer to Section 8.3) involving risk to subjects or others, serious adverse events and all subject deaths associated with the protocol and provide an unbiased written report of the event. At a minimum, the LSM should comment on the outcomes of the event or problem, and in the case of a serious adverse event or death, comment on the relationship to participation in the study. The LSM should also indicate whether he concurs with the details of the report provided by the study investigator. Reports for events determined by either the investigator or LSM to be possibly or definitely related to participation and reports of events resulting in death should be promptly forwarded to the HSRRB and GSK Biologicals

5.1.3.2. Safety Monitoring Group (SMG)

The Safety Monitoring Group will include; Dr Al Magill, WRAIR; a representative of Clinical Safety, GSK Biologicals; Dr Art Brown, USAMMDA and Dr Norbert Peshu, KEMRI.

The SMG is not an independent Data Safety Monitoring Board. It is a group of experienced clinicians, from the organizations involved in the study, that is charged

thorough evaluation of the safety reports after Dose 1 and Dose 2. The members do not form a part of the investigator groups; i.e. the investigator site, the GSK Biologicals malaria vaccine clinical project team or the investigator group at WRAIR. They will be one of the parties responsible for deciding whether to progress to the next dose and whether to resume immunizations if the trial has been suspended. The SMG may, if deemed necessary, convene a meeting with, or request further information from the PI or LSM.

A charter will be agreed upon prior to initiation of this study that will further define the role of the SMG.

5.1.3.3. Safety Reports

Safety reports will be written on the first 60 subjects to receive Dose 1 and Dose 2. These reports will be blinded to vaccine allocation and will contain:

- All SAEs and any relationship to vaccines to date;
- For each dose, all Grade 3 solicited and unsolicited symptoms by relationship to vaccine;
  - Should the PI judge a Grade 3 case to be unrelated to vaccination, an alternative explanation for the cause of the symptom will be provided;
- All indicators of hepatic and renal function (as per Table 32) above the normal range (i.e. Gradings 1 to 3);
  - For all subjects above normal range, clinical details will be provided;
- Parameters of complete blood count below normal range (as per Table 33) post Dose 1;
  - For all subjects below normal range, clinical details will be provided;
- All withdrawals of study subjects by the PI due to adverse events;
  - For all subjects withdrawn clinical details will be provided.

5.1.3.4. Process for the suspension of vaccination

5.1.3.4.1. Indications for immediate suspension of all vaccination

The PI will suspend all vaccination for any of the following SAEs;

- Death or life-threatening SAE which is judged to be related to the study vaccine;
- Anaphylactic shock reaction or significant IgE-mediated event in an enrolled subject following vaccination.

The LSM, sponsor (USAMMDA) or GSK Biologicals may suspend all vaccination for any one SAE or pattern of SAEs.

5.1.3.4.2. Indications for suspension of progression to next sequential dose

If the blinded safety report shows ≥ 35% of subjects have a Grade 3 systemic reaction (solicited or unsolicited)

If the blinded safety report shows ≥ 5% of subjects have a Grade 3 laboratory abnormality of any one indicator of hepatic or renal function as defined in Table 32.

If the blinded safety report shows ≥ 5% of subjects are withdrawn by the PI or withdraw themselves due to local or systemic reactogenicity.

5.1.3.4.3. Process if the trial is suspended

The trial may be **temporarily** suspended by the LSM, PI or the SMG. The SMG will review all available safety data which may include a full description of reactogenicity. If the SMG judge it to be necessary, the safety data will be unblinded. However the final responsibility to recommend whether or not the trial should be stopped **permanently** rests with the sponsor (USAMMDA) in consultation with GSK Biologicals, after having considered all the safety information available.

In the event that the trial is temporarily suspended, the FDA will be informed by the sponsor (USAMMDA). If the SMG's recommendation is to continue, then a report will be submitted to the FDA detailing the rationale used in reaching this decision. The agreement of the FDA will be obtained prior to restarting the trial.

5.1.4. Exposure to rabies

All subjects will be reminded at each Clinic Visit that should they come into contact with an animal who they suspect to have rabies, they must immediately consult a physician for treatment.

5.1.5. Cross over immunization with rabies vaccine

After completion of the study (12 months after Dose 1), rabies vaccination will be offered to all subjects that did not receive it during the study (i.e. those subjects that received RTS,S/AS02A or RTS,S/AS01B)

5.2. Recruitment/Screening

5.2.1. Community information

The community in which the study will take place will be informed about the nature and design of the study. Community leaders (assistant chiefs and local village elders) will be formally briefed in their own language on the nature and purpose of the study. They will have the opportunity to ask questions of the PI or his designees.

5.2.2. Recruitment

Adults age 18 to 35 years will be recruited from the villages in Kombewa District around the Kombewa Clinic. Non-coercive means of recruitment will be used according to existing U.S. Army regulations (viz., AR 70-25 and AR 40-38). Prospective subjects will view a videotaped briefing on the nature and purpose of the study and will then receive both oral and written explanations of the study. Afterwards, written informed consent will be obtained from each person who wants to participate in the study. No question relating to the potential study subject's health will be asked prior to consenting. So that language and illiteracy will not be impediments to informed consent, all briefings and explanations will be in Dholuo, the local language. All screening tests and procedures (refer to 5.2.3 and Appendix C) will be performed during a separate visit. It is expected that at least 500 adult volunteers will have to be recruited to achieve the final enrolment number. Refer to Appendix C for an overview of the recruitment plan of the study.

5.2.3. Screening of volunteers

Screening will be done on a separate visit from recruiting. Only adults aged 18 to 35 years at the scheduled time of first vaccination with a written Informed Consent, signed/thumb printed and dated will be eligible to be screened for the trial.

Volunteers will provide a medical history through a one-on-one interview with a clinical officer. Volunteers will also undergo physical examination and standard laboratory screening tests, which include complete blood count (CBC), creatinine, and ALT. Blood to screen for hemoglobinopathies (sickle cell disease/trait, G6PD deficiency, and alpha-thalassemia) will also be drawn. Volunteers may be screened one additional time if re-screening is determined to be necessary (e.g. after treatment for malaria). Volunteers will be excluded from participation if they meet any of the exclusion criteria. Volunteers excluded from this study because of significant abnormalities will be referred to a named specialist at the New Nyanza Hospital for evaluation and treatment as described in Section 5.7.3). All screening tests will be completed within 42 days prior to entry into the study. Information gathered during screening (medical history, physical examination, and laboratory analysis) will be recorded in the Source Documents and the GSK Biologicals' CRFs.

A photograph will be taken of each volunteer who is screened and stored securely in a computer at KC. When screening and review of inclusion/exclusion criteria are complete, a picture ID card of each eligible volunteer will be created using the stored photo. In addition to the subject's photo, name, subject number and the study number and name, the card will contain contact information for KC. This will ensure that the subject can contact an investigator, and the clinic can be contacted if medical care is received outside the study health facilities. Copies of these pictures will be kept in the subjects' records to aid the study staff in confirmation of the volunteers identification for future visits. The photos of subjects who are determined ineligible to participate will be deleted from the computer.

At the screening visit each subject will be given a Subject Number and each will have a clinic record prepared. Subject Numbers will be issued consecutively in the order in

which the subjects arrive at the health center. This number will be used throughout the study to identify every document and blood sample associated with the volunteer. The clinic records will contain the Subject Number, the subject's date of birth, medical history, findings of the physical examination, the date of screening visit, whether the subject was enrolled, and (where applicable) reasons for exclusion from the study. The name of the study will be written on both the ID card and clinic records of enrolled volunteers.

All screening laboratory studies will be performed at WRP laboratories. After reviewing the medical history, physical examination and laboratory results, any reasons for non-eligibility will be documented on the screening list and in the GSK Biologicals Screening CRF.

Field workers will notify each volunteer of their eligibility to enroll in the study. It is expected that at least 500 volunteers will have to be screened to meet the required number for enrolment. Any volunteers who are found to be ineligible for the trial due to illness will be referred for specialist medical care as detailed in Section 5.7.3.

5.3. Vaccination process

After screening, all eligible volunteers will be asked to return for vaccination. On the day of Dose 1, randomization will occur on a first come, first served basis until the target number for the study has been reached. The RTS,S/AS02A, RTS,S/AS01B and Rabies vaccines will be packaged in identical boxes and will be identified by a Treatment Number. The unique Treatment Number will identify each dose of the vaccine administered to each subject. After randomization the Treatment Number will assign the subject to one group or another in a blinded way. The Treatment Number will be issued consecutively at Clinic Visit 2. Each subject will retain the same Treatment Number for their subsequent vaccine doses. The Treatment Number will be recorded on the subject's Clinic Record and in the GSK Biologicals CRF after the vaccine has been administered. The Clinic Record and GSK Biologicals CRF link the Subject Number and the Treatment Number.

Vaccinations will take place at KC. On each vaccination day, criteria for continued eligibility will be reviewed and verified. A history-directed physical examination will be done and oral temperature, blood pressure and baseline general symptom history will be recorded. Volunteers will then have venous blood collected for laboratory analysis as detailed in Section 5.3. The volunteer will then be placed in one of the individual vaccination rooms adjacent to the pharmacy.

After the identity of the subject is again checked (by comparing the Subject Number and photograph in the subject's record with the subject's photo ID), the photo ID and the Clinic Record will be given to the pharmacy through a hatch connecting the vaccination room with the pharmacy. The pharmacy will then prepare the appropriate vaccine following a consecutive treatment number assignment and return it to the vaccination room (with the Clinic Record) through the same hatch, for administration.

Since the vaccines used in this study are of distinct appearance, the Pharmacist and the Vaccinator are not blinded and perform no other function in the study (refer to Section 6.4). The Pharmacist will select the sealed box labeled with the subject's Treatment Number, containing the vials numbered with the Treatment Number, remove the vaccine vials and fill a syringe according to this study protocol (refer to Section 6.2). The Pharmacist will then place a numbered opaque label with the subject's Treatment Number on the syringe. The purpose of masking the syringe is to blind the subject. After administering the vaccination to the subject, the Vaccinator will enter the Treatment Number administered to the subject on the clinic form. Subjects will be vaccinated by deltoid intramuscular injection into the non-dominant arm by a qualified nurse. The volunteer will not know what injection was given. A staff member experienced in resuscitation will be available at all vaccination sessions. Facilities and equipment will be available to give emergency treatment in the case of an anaphylactic reaction following administration of vaccines.

Immediately after vaccination, each subject will proceed to the next station for assessment of local and systemic reactions. Each subject will be observed for at least 30 minutes post-vaccination. Appropriate medical treatment will be readily available in the unlikely case of an anaphylactic reaction following the administration of the vaccine.

Subjects who cannot be vaccinated on the originally scheduled date (due to acute illness, local lesion at the injection site or scheduling conflicts) may be vaccinated if eligible within the next 5 days. In the particular case of any subject found to be febrile (oral temperature ≥ 37.5°C), a malaria blood film (MBF) will be taken to investigate for malaria. Subjects will be treated as required for their condition and will be followed up until resolution of any symptoms and be vaccinated if their clinical symptoms resolve within 5 days.

Those who cannot be re-vaccinated within 5 days of their scheduled date will continue all study procedures apart from receiving further study vaccinations.

5.4. Home follow-up visits for assessment of reactogenicity (7-day follow-up period)

Subjects will be evaluated daily for seven days after each vaccination (day of vaccination plus six more days). Evaluation will be conducted at designated field stations by trained field workers under the supervision of the PI. Subjects will be evaluated by soliciting post-vaccination signs and symptoms from the volunteer and by examination of the injection site. Results of each visit will be recorded by the field worker on the diary card for that subject.

In the event that a field worker finds any Grade 3 solicited or unsolicited sign/symptom, the subject will be brought to KC for examination by a study clinician. The evaluation and any further clinical data, including treatment provided, will be written on the diary card as well as clinic forms and transcribed onto the CRF. If the clinician determines that the subject has experienced a serious adverse event (SAE), appropriate measures will be taken to notify the Local Safety Monitor (LSM), the SMG, the IRBs, the sponsor, USAMMDA, GSK Biologicals and WRAIR.

Every effort will be made to ensure that each subject completes every visit. If a subject does not appear for a scheduled clinic visit, field workers will be dispatched to their home that same day to attempt to contact them. Regular attempts to contact the subject will continue until the study ends or a reason is found for missing the visit (e.g. the subject moved away from the study area). When the subject is contacted the field worker will measure vital signs and collect information on any solicited or unsolicited symptoms since the last scheduled visit.

After each follow-up period, diary cards will be checked and verified by the PI or designate prior to transcription onto CRFs. The PI has a primary responsibility for the data transcribed onto the CRFs. Unresolved AEs will be followed-up by field workers until resolution under the supervision of the Principal Investigator and data will be entered onto the CRF. The procedures and frequency of visits will be outlined in a SOP at the investigator's site.

5.5. Monitoring of hematological and biochemical laboratory parameters

Results of hematological and biochemical laboratory tests will be reviewed as soon as possible after they are generated. Any value outside the normal range will be managed as appropriate by a medically qualified individual under the supervision of the Principal Investigator. The hematological and biochemical parameters will be documented on the CRF. For all values outside the normal range, the reason and/or clinical condition will be documented. Guidance on when to report abnormalities as SAEs is given in Section 8.3.

5.6. Surveillance for SAEs (all subjects)

All study subjects will have access to free health care at Kombewa Clinic 24 hours/day through one of the field stations located within one mile of their residence. Study subjects will be encouraged to present to the clinic or field station for any medical issue that may arise during the study. When a subject presents to a field station, transportation will be provided to KC where medical staff is available 24 hours/day. This will be the primary means of SAE surveillance.

Subjects are also given contact numbers for the clinical staff at KC on their study ID card. Should a study subject have to present to some other medical facility they are instructed to present the card to the health care provider who can then contact KC and arrange for transportation, treatment or payment of treatment for the subject. If a subject is treated at a medical facility other than KC, an attempt will be made to obtain a copy of the treatment record for review by the PI.

Additionally, volunteers will be asked if they have been seen or treated at an outside medical facility since their last visit to KC. If so, the PI will request a copy of the record from the relevant medical facility

The record of every acute clinic visit (at KC or otherwise) will be reviewed by the PI or designate for SAEs.

5.7. Study site; health care provision in the study area

5.7.1. Outpatient Care at Kombewa Clinic

The capabilities of the clinic and continuous staffing have been developed to ensure any acute illness or outpatient medical need of anyone involved in a study can be addressed 24 hours a day. The KC is staffed at all times (24 hours a day) during a study with at least a clinical officer, community nurse and a driver with an evacuation vehicle. During working hours, Monday though Friday, the basic staffing also includes a medical officer (physician), pharmacist, lab technician, and microscopist.

All outpatient services for study participants will be provided by the staff at KC. Transportation to the clinic and medical staff will be available 24 hours a day.

5.7.2. New Nyanza Provincial General Hospital

New Nyanza Provincial General Hospital (NNPGH) in Kisumu is the primary referral site for study participants requiring inpatient care or blood transfusion. If either is required, KC personnel will transport the subject to NNPGH. This hospital includes two adult wards with 30 beds each, oxygen supplementation capabilities, 24-hour nursing and physician coverage, radiology, laboratory, pathology, surgical suites, and screened blood supply. The subject will be admitted to the care of Kenyan physicians on staff. Subjects who are admitted will be followed daily by clinical personnel from KC as well. Specialty care will be coordinated for by KC personnel as required. The medical record from NNPGH will be the SAE source document.

5.7.3. Referring of Excluded Volunteers to Medical Care

If a volunteer is found to have a medical condition that excludes them from the trial they will be informed at a private appointment with a member of the clinical staff. The clinical staff member will take as much time as required to explain the medical condition including its severity, potential causes, long-term complications (if there are any), impact on current lifestyle and evaluation and treatment options. Once it is clear the volunteer understands the medical condition, the clinical staff member will develop an evaluation and treatment plan with the volunteer and ensure that the volunteer understands their options.

WRP has identified specialty and sub-specialty physicians in the local area. If a volunteer is excluded from the study for a medical condition or laboratory abnormality not previously known to the volunteer they will be referred for care to one of these physicians according to their illness. WRP personnel will coordinate with the relevant physician and the volunteer to make an appointment and provide transportation to the appointment if necessary. The history, physical exam, and laboratory findings that lead to exclusion and referral for evaluation will be provided to the subject and the referral physician. WRP will not pay for the referral but will ensure that the appointment is coordinated and transportation is provided.

5.8. Management and treatment of malaria in all subjects

Infection with malaria parasites is defined as *P. falciparum* asexual parasitemia > 0. If a subject is found to have a positive malaria smear (with or without symptoms) the subject will be treated with a regimen of artemether/lumefantrine (Coartem®, Novartis AG, Zurich, Switzerland). This regimen was introduced by the Kenyan Ministry of Health in April 2004 as the new treatment of choice of non-severe malaria in all age groups. Six doses over 60 hours is the regimen recommended by the World Health Organization for use in malaria endemic areas such as Kenya. See table below for specific dose timings:

Dose Number	Number of tablets per dose (20/120mg)	Dose Timing (hours after first dose)
**1**	4	0
**2**	4	8
**3**	4	24
**4**	4	36
**5**	4	48
**6**	4	60

In the event of treatment failure or inability to tolerate artemether/lumefantrine, Malarone® (atovaquone and proguanil hydrochloride, GlaxoSmithKline, Uxbridge, UK) or quinine will be used as the alternative outpatient treatment. The dosing regimen of quinine for adults with acute malaria is 600 mg orally every 8 hours for seven days.

Subjects with vomiting or manifestations of severe malaria (including mental status changes, convulsions, renal failure) will be admitted to NNPGH for treatment with parenteral antimalarials. Quinine will be used for severe malaria. A member of the KC clinical team will review the subject admitted to NNPGH daily until discharge from hospital.

All study subjects treated for malaria will be followed (at the clinic or by field worker home visit) until resolution of symptoms. All treatment courses will be administered under the direction of the attending clinician and recorded in the subject's record. Subjects found to have clinical malaria will be treated before any further vaccination. Subjects will be given the next dose of vaccine if their clinical symptoms resolve within the vaccination window. If the clinical symptoms do not resolve within that period, the subject will not be vaccinated; however, the subject will be followed for collection of all study data points.

5.9. Passive case detection of clinical cases of malaria

In the event that a subject presents to one of the field stations with fever (≥ 37.5ºC) or history of fever during the past 24 hours they will be transported to KC and evaluated by a clinician. An MBF will be taken and read immediately and complete blood count (CBC) evaluated. All subjects with asexual parasitemia will be treated. The procedures for MBFs are outlined in site SOPs.

Passive case detection of malaria will begin with screening and continue until 16 weeks post Dose 3 (end of the double-blind period).

5.10. Active detection of infection

The ADI period for malaria infection will begin at Day 74 (Field Worker Visit 40), 14 days after Dose 3. ADI visits will occur weekly for 14 weeks. Blood samples will be taken for examination of malaria parasitemia at each ADI visit. ADI visits will continue through to Month 6½ (Clinic Visit 8).

Three weeks prior to the start of surveillance for malaria infection (i.e. 1 week prior to Dose 3), all subjects will be presumptively treated for aymptomatic parasitemia with Malarone® (atovaquone and proguanil hydrochloride, GlaxoSmithKline, Uxbridge, UK). (in the event that subjects are unable to tolerate Malarone®, Coartem® (artemether/lumefantrine, Novartis AG, Zurich, Switzerland) will be used as the alternative clearance treatment. The subjects will visit field workers for three consecutive days. At each of these visits, the field worker will give the subject four tablets of Malarone® (containing 250 mg atovaquone and 100 mg proguanil hydrochloride), and observe the subject taking the tablets. The first dose of medication will be administered at the field stations on Study Day 53 (Field Worker Visit 32). The second dose will be given under observation the next morning by the field worker team (Study Day 54; Field Worker Visit 33). The third dose (the final one) will be given by the field worker team the following morning (Study Day 55; Field Worker Visit 34).

The absence of parasitemia prior to the start of the ADI period will be checked by MBF one week post Dose 3 (Study Day 66; Clinic Visit 6). Any subject with any level of parasitemia at this point will be treated with the second-line treatment for malaria (Coartem®).

For the ADI period, a field worker visit will consist of the subject presenting to a field station or the KC and completing a brief surveillance for infection morbidity questionnaire, which will include the reporting of malaria symptoms and a record of oral temperature. If the subject is well and afebrile, a blood-sample will be taken and examined for malaria parasitemia and evaluated for CBC. Blood slides from the sample will be Giemsa-stained and read at KC to determine the presence of parasites. Treatment for asymptomatic subjects with positive MBF will be sent by a field worker within 72 hours of the when the MBF was taken.

In the event that a subject reports a history of fever within the preceding 24 hours or has a documented oral temperature of ≥ 37.5°C, no blood slides will be taken by the field worker but transport by a project vehicle for the subject to KC will be arranged. The surveillance for infection morbidity questionnaire will record that the subject was transferred to KC for further evaluation. At KC the subject will be evaluated by a clinician and a venous blood sample will be taken for CBC, determination of parasitemia. Subjects with a positive MBF will be managed according to the procedures detailed above (see Section 5.8). Subjects who are diagnosed in such a manner will be classified as diagnosed by ADI.

At first detection of malaria infection with asexual forms of *P. falciparum*, either by ADI surveillance or on clinical presentation with malaria disease, the subject will be considered to be infected and no further visits for surveillance of infection will be

undertaken. The subject will still be under morbidity surveillance and encouraged to seek care at the KC.

5.11. Subject identification

Subject Numbers will be issued consecutively to all volunteers on the day of screening. To identify the volunteer at subsequent contacts, they will be issued a photo ID that includes the volunteer's subject number.

5.12. Outline of study procedures

Table 27 List of study procedures

	Recruit/ Screen	DOUBLE-BLIND PHASE													SINGLE-BLIND PHASE	
		VACCINATION									ADI					
		1			2		X	3								
**Study Day**	**-42 to 0**	**0**	**1-5**	**6**	**30**	**31-36**	**53-55**	**60**	**61-65**	**66**	**2 weekly visits**	**90**	**10 weekly visits**			
**Study Month**		**0**			**1**			**2**				**3**		**6½**	**7 to 11**	**12**
**Clinic Visit**	**1**	**2**		**3**	**4**			**5**		**6[f]**		**7[a]**		**8[a]**		**9**
**Field Worker Visit Code #**			**21-25**			**26-31**	**32-34**		**35-39**		**40, 41**		**42-51**		**52-56**	
**STUDY PROCEDURES**																
Informed consent	•															
Medical history	•															
Vital Signs	•	○		○	○			○		○		○		○		○
Complete Physical examination	•															
Assign PID	•															
Focused/Symptom directed Physical exam		•		•	•			•		•		•		•		•
Check inclusion/exclusion criteria	•	○														
Check of elimination criteria		•		•	•			•		•						
Pregnancy test	•	•			•			•								
Check contraindications to vaccination		•			•			•								
Randomization		•														
Administer RTS,S/AS02A, RTS,S/AS01B or Rabies vaccine		•			•			•								
Recording of medication		• [g]	• [g]	• [g]	• [g]	• [g]		• [g]	• [g]	• [g]	• [g]	• [g]	• [h]	• [h]		• [l]
Administer drug for clearance of parasitemia							•			• [d]						
**SAFETY DATA COLLECTION**																
Recording of Solicited Symptoms (Investigator)		•		•	•			•		•						
Solicited symptoms; field workers			•			•			•							
Recording of unsolicited AEs up to 1 month post-vaccination by investigator		•		•	•			•		•		•				
Recording of unsolicited AEs by Field Workers			•			•			•		•					
Morbidity surveillance/recording of SAEs	• [j]	•	•	•	•	•	•	•	•	•	•	•	•	•	•	•
History of fever (for ADI)										~~•~~	•	•	•	•		
Temperature (for ADI)										~~•~~	•	•	•	•		
**SAFETY LABS**																
Complete blood count [b]	•			•						•	• [e]	•	• [e]	•		•
Creatinine, ALT, Bilirubin	•			•								•				
Hemoglobinopathy screen	•															
Malaria Blood Film										•	• [e]	•	• [e]	•		
**INVESTIGATIONAL ASSAYS**																
Antibodies to HBs		•										•				•
HBsAg	•															
Antibodies to-CS		•						•				•		•		•
Cell-mediated immunity		•										•		•		•
Parasite Genotyping		•									•	•	•	•		
**INTERIM ANALYSIS SAFETY & IMMUNOGENICITY**												•				
**FINAL ANALYSIS**														•		
**STUDY CONCLUSION**																•

for table notes, please refer overleaf…

ADI; Active Detection of Infection
X; Drug for the presumptive clearance of *Plasmodium* parasitemia will be given during these visits
[a] all subjects will be required to attend these cross-sectional visits, regardless of whether or not they have previously reported malaria during the ADI period
[b] Includes analysis of hemoglobin, total white cell count absolute lymphocyte count and platelets. Done with safety labs and with MBF to determine parasite count
[c] If a subject presenting to a health center or field site reports fever within the preceding 24 hours or has a documented fever (oral temperature ≥ 37.5°C) then blood will be taken for parasitemia determination and hemoglobin
[d] any subjects who test positive for malaria parasitemia on this visit will be administered Coartem® to reclear them
[e] these blood samples are carried out on subjects for as long as they take part in the ADI Phase; if a subject has a positive MBF they no longer partake in this sampling
[f] this visit serves as both the final visit for soliciting symptoms at Day 6 post Dose 3 and the first visit for ADI
[g] all medications to be recorded at these visits
[h] record administration of immunosuppresants or other immune-modifying drugs during this period (for corticosteroids this means prednisone or equivalent, ≥ 0.5 mg/kg/day. Inhaled or topical steroids are allowed and should not be recorded. ALSO all immunoglobulins and blood products should be recorded during this period. ALSO any antimalarials.
[I] record administration of immunosuppresants or other immune-modifying drugs during this period (for corticosteroids this means prednisone or equivalent, ≥ 0.5 mg/kg/day. Inhaled or topical steroids are allowed and should not be recorded. ALSO all immunoglobulins and blood products should be recorded during this period.
[j] SAEs related to study procedures will be collected
● is used to indicate a study procedure that requires documentation in the indivudual CRF and ○ is used to indicate a study procedure that does not require documentation in the individual CRF. *SAEs that are related to study participation or are related to a concurrent medication will be collected and recorded from the time the subject consents to participate in the study until they are discharged

Amended 13 June 2006

5.13. Detailed description of study visits

When materials are provided by GSK Biologicals, it is **MANDATORY** that all clinical samples (including serum samples) will be collected and stored using exclusively those materials in the appropriate manner. The use of other materials could result in the exclusion of the subject from the ATP analysis (see Section 10.6 for definition of study cohorts to be evaluated). The investigator must ensure that his/her personnel and the laboratory(ies) under his/her supervision comply with this requirement. However, when GSK Biologicals does not provide material for collecting and storing clinical samples, then appropriate materials from the investigator's site are to be used. Refer to Appendix D and Appendix E.

The subjects will be instructed to contact the investigator immediately should the subject manifest any signs or symptoms they perceive as serious.

The following schedule reflects the visits for a single subject.

Table 28 Intervals between study visits

Interval	Length of interval
Clinic Visit 1 → Clinic Visit 2	0 to 42 days
Clinic Visit 2 → Clinic Visit 4	21 to 35 days
Clinic Visit 4 → Clinic Visit 5	21 to 35 days
Clinic Visit 5 → Clinic Visit 7	21 to 35 days
Clinic Visit 5 → Clinic Visit 8	4 months ± 1 month
Clinic Visit 8 → Clinic Visit 9	6 months ± 1 month
Clinic Visit 5 → Clinic Visit 9	9 months ± 2 months

RECRUITING AND SCREENING

Clinic Visit 1: Screening
Day –42 to Day 0

- Obtain written Informed Consent
- Assign Subject Number
- Record oral temperature, blood pressure,
- Provide medical history by subject
- Health assessment and physical examination (includes physical exam of all major body systems: ENT, pulmonary, cardiovascular, musculoskeletal, central nervous, renal, gastrointestinal and skin). Abnormal findings will be recorded in the subject's record.
- Collect 15 mL venous blood sample for measurement of:
  - Complete blood count (CBC), creatinine, ALT, Bilirubin
  - HBsAg
  - Hemoglobnopathies (Sickle cell, G6PD deficiency, and alpha-thalassemia)
- Check all inclusion and exclusion criteria
- For all female subjects, carry out pregnancy test. Vaccine may only be given if women have negative pregnancy test
- Provide each volunteer with study ID card containing their Study Number and photograph.
- Record any SAEs that may have occurred as a result of study procedures

VACCINATION

Clinic Visit 2: Vaccination 1
Day 0

Before vaccination:

- Check volunteer's study ID card to confirm identity
- Record oral temperature, blood pressure
- Review screening laboratory test results
- Review inclusion/exclusion criteria and check for contraindications/ precautions
- Record any subject complaints, interim history, history-directed physical examination and examination of the vaccination site for any abnormalities
- Review concomitant medications

- Record baseline data for solicited general symptoms
- For all female subjects, carry out pregnancy test. Vaccine may only be given if women have negative pregnancy test
- Check elimination criteria
- Collect 15 mL of whole venous blood for the measurement of:
  - Serology (antibodies to CS and antibodies to HBs) (requires 3 mL)
  - antibodies to CS in Plasma (requires 1 mL)
  - MBF (only in symptomatic subjects)
  - Cell-Mediated Immunity (requires 9 mL)
  - Malaria Parasite Genotyping (sample to be analyzed from all subjects)

Vaccination:

- Randomize subject to a vaccination group
- Confirm that the subject's randomization number and Subject Number agree with label on syringe
- Administer study vaccine Dose 1; record site, date of injection.

After vaccination:

- Observe each vaccinee for at least 30 minutes.
- Record blood pressure, oral temperature at the end of post-vaccination observation period
- Examine site of injection
- Record solicited and unsolicited AEs
- Record any post-vaccination SAEs
- Instruct subject to return to KC immediately should subject manifest any sign or symptom which they perceive as serious.

Field Worker Visit Code Numbers 21 to 25: Daily Post-vaccination follow up visits Day 1 to Day 5

- Examine site of injection
- Record solicited and unsolicited AEs
- Record SAEs experienced by the vaccinee since the last visit
- Check concomitant medications
- Refer subjects with fever, history of fever in previous 24 hours or Grade 3 AEs to KC

Clinic Visit 3: Collection of blood sample
Day 6

- Check volunteer's study ID card to confirm identity
- Record oral temperature, blood pressure
- Examine site of injection
- Record solicited and unsolicited AEs
- Record SAEs experienced by the vaccinee since the last visit
- Record any subject complaints, interim history, history-directed physical examination and examination of the vaccination site for any abnormalities
- Review concomitant medications
- Check elimination criteria
- Collect 2 mL whole venous blood for the measurement of
  - CBC, creatinine, ALT, Bilirubin

Clinic Visit 4: Vaccination 2
Day 30

Before vaccination:

- Check subject's study ID card to confirm identity
- Record oral temperature, blood pressure
- Review elimination criteria and check for contraindications/ precautions
- Record any subject complaints, interim history, history-directed physical examination and examination of the vaccination site for any abnormalities
- Review concomitant medications
- For all female subjects, carry out pregnancy test. Vaccine may only be given if women have negative pregnancy test
- Check elimination criteria

Vaccination:

- Confirm that the subject's randomization number and Subject Number agree with label on syringe
- Administer study vaccine Dose 2; record site, date of injection.

After vaccination:

- Observe vaccinee for at least 30 minutes.
- Record blood pressure, oral temperature at the end of post vaccination observation period

- Examine site of injection
- Record solicited and unsolicited AEs
- Record any post-vaccination SAEs
- Instruct subject to return to KC immediately should subject manifest any sign or symptom which they perceive as serious.

Field Worker Visit Code Numbers 26 to 31: Daily Post-vaccination follow up visits Day 31 to Day 36

- Record oral temperature
- Examine site of injection
- Record solicited and unsolicited AEs
- Record SAEs experienced by the vaccinee since the last visit
- Check concomitant medications
- Refer subjects with fever, history of fever in previous 24 hours or Grade 3 AEs to KC

Field Worker Visit Code Numbers 32 to 34: Malarone® administration for presumptive clearance of parasites Day 53 to 55

- Begin three-day Clearance Regimen
  - First Day: Field worker will administer first dose (four tablets) of Malarone®.
  - Second Day: Field worker will administer second dose (four tablets) of Malarone®.
  - Third Day: Field worker will administer third dose (four tablets) of Malarone®.
- Record SAEs experienced by the vaccinee since the last visit

Clinic Visit 5: Vaccination 3 Day 60

Before vaccination:

- Check subject's study ID card to confirm identity
- Record oral temperature, blood pressure
- Review elimination criteria and check for contraindications/ precautions
- Record any subject complaints, interim history, history-directed physical examination and examination of the vaccination site for any abnormalities
- Review concomitant medications

- For all female subjects, carry out pregnancy test. Vaccine may only be given if women have negative pregnancy test
- Check elimination criteria
- Collect 2½ mL of whole venous blood for the measurement of:
  - Serology (antibodies to CS)

Vaccination:

- Confirm that the subject's randomization number and Study Number agree with label on syringe
- Administer study vaccine Dose 3; record site, date of injection.

After vaccination:

- Observe vaccinee for at least 30 minutes.
- Record blood pressure, oral temperature at the end of post-vaccination observation period
- Examine site of injection
- Record solicited and unsolicited AEs
- Record any post-vaccination SAEs
- Instruct subject to return to KC immediately should subject manifest any sign or symptom which they perceive as serious.

Field Worker Visit Code Numbers 35 to 39: Daily Post-vaccination follow up visits Day 61 to Day 65

- Record oral temperature
- Examine site of injection
- Record solicited and unsolicited AEs
- Record SAEs experienced by the vaccinee since the last visit
- Check concomitant medications
- Refer subjects with fever, history of fever in previous 24 hours, or Grade 3 AEs to KC

Clinic Visit 6: Check for clearance of parasitemia Day 66

- Check volunteer's study ID card to confirm identity
- Record oral temperature, blood pressure~~, pulse~~
- Record oral temperature, solicit history of fever in the last 24 hours

- Examine site of injection
- Record solicited and unsolicited AEs
- Record SAEs experienced by the vaccinee since the last visit
- Record any subject complaints, interim history, history-directed physical examination and examination of the vaccination site for any abnormalities
- Review concomitant medications
- Check elimination criteria
- Collect 2 mL whole venous blood for the measurement of
  - MBF and CBC
  - Malaria Parasite Genotyping (sample to be analyzed only if MBF result is positive)

All subjects who are positive for malaria parasites should be treated with the secondary clearance drug, Coartem® as described in Section 5.8. These subjects are to be included in ADI surveillance

ACTIVE DETECTION OF INFECTION

Field Worker Visit Code Number 40 and 41: ADI visits #1 and 2 Weekly Visits

- Record oral temperature, solicit history of fever in the last 24 hours
- Transport any subject with a fever or history of fever in the past 24 hours to KC for evaluation, including MBF and CBC and malaria parasite genotyping
- Draw 2 mL of blood from asymptomatic patients for:
  - MBF and CBC
  - Malaria Parasite Genotyping (sample to be analyzed only if MBF result is positive)
- Document any hospitalizations, clinic visits, deaths, out-migrations, absences from Kombewa Study Area since last visit
- Record SAEs experienced by the vaccinee since the last visit
- Check concomitant medications

Clinic Visit 7: ADI visit #3 (Clinic Visit)
Day 90

All subjects who received Dose 3 should be invited to this visit regardless of whether they have had a positive MBF or reported having had malaria during the preceding ADI visits

- Record blood pressure
- Record oral temperatures, solicit history of fever in the last 24 hours
- Record any subject complaints, interim history, history-directed physical examination and examination of the vaccination site for any abnormalities
- Transport any subject with a fever or history of fever in the past 24 hours to KC for evaluation, including MBF and CBC and malaria parasite genotyping
- Record SAEs experienced by the vaccinee since the last visit
- Draw 15 mL of blood from asymptomatic patients for:
  - Creatinine, ALT, bilirubin
  - MBF and CBC (this sample will not be carried out on subjects who have already had a positive MBF or reported having had malaria during the preceding ADI visits)
  - Malaria Parasite Genotyping (sample to be analyzed only if MBF result is positive)
  - Serology (antibodies to CS and antibodies to HBs) (requires 3 mL)
  - Cell-mediated immunity (requires 9 mL)
- Document any hospitalizations, clinic visits, deaths, out-migrations, absences from Kombewa since last visit
- Check concomitant medications

Field Worker Visit Code Numbers 42, 43, 44, 45, 46, 47, 48, 49, 50, 51:
ADI visits # 4, 5, 6, 7, 8, 9, 10, 11, 12, 13
Weekly visits

- Record oral temperature, solicit history of fever in the last 24 hours
- Transport any subject with a fever or history of fever in the past 24 hours to KC for evaluation, including MBF and CBC and malaria parasite genotyping
- Draw 2 mL of blood from asymptomatic patients for:
  - MBF and CBC
  - Malaria Parasite Genotyping (sample to be analyzed only if MBF result is positive)

- Document any hospitalizations, clinic visits, deaths, out-migrations, absences from Kombewa Study Area since last visit
- Record SAEs experienced by the vaccinee since the last visit
- Check concomitant medications

Clinic Visit 8: Final Visit for ADI
Month 6½

All subjects who received Dose 3 should be invited to this visit regardless of whether they have had a positive MBF or reported having had malaria during the preceding ADI visits

- Record blood pressure
- Record oral temperatures, solicit history of fever in the last 24 hours
- Record any subject complaints, interim history, history-directed physical examination and examination of the vaccination site for any abnormalities
- Record SAEs experienced by the vaccinee since the last visit
- Transport any subject with a fever or history of fever in the past 24 hours to KC for evaluation, including MBF and CBC and malaria parasite genotyping
- Draw 15 mL of blood from asymptomatic patients for
  - MBF
  - CBC
  - Malaria Parasite Genotyping (sample to be analyzed only if MBF result is positive)
  - Serology (antibodies to CS) (requires 1 mL)
  - Cell-mediated immunity (requires 9 mL)
- Document any hospitalizations, clinic visits, deaths, out-migrations, absences from Kombewa since last visit
- Check concomitant medications

SAFETY FOLLOW-UP

Field Worker Visit Code Numbers 52, 53, 54, 55, 56
Month 7, 8, 9, 10, 11

- Record SAEs experienced by the vaccinee since the last visit

FINAL STUDY VISIT

Clinic Visit 9
Month 12

- Record oral temperature, blood pressure
- Draw ***4 13*** mL of blood from asymptomatic patients for
  - CBC
  - Serology (antibodies to CS and HBs) (requires 3 mL)
  - ***Cell-mediated immunity (requires 9 mL)***
- Document any hospitalizations, clinic visits, deaths, out-migrations, absences from Kombewa since last visit
- Record SAEs experienced by the vaccinee since the last visit
- Check concomitant medications
- Inform subjects of study end

Amended 13 June 2006

END OF STUDY

5.14. Sample handling and analysis

5.14.1. Treatment and storage of biological samples

See Appendix D of the protocol for details of treatment and storage of biological samples

See Appendix E for instructions for shipment of biological samples

5.14.2. Laboratory assays

Table 29 Summary of laboratory immunology tests to be performed

Assay	Marker	Assay method	Test Kit/ Manufacturer	Assay unit	Assay cut-off	Laboratory
Anti-CS antibodies	R32LR	ELISA	In-house ELISA	EU/mL	0.5	Leroux-Roels Laboratory, Ghent, Belgium
Anti-HBs antibodies		EIA	AUSAB EIA ABBOTT†	mIU/mL	3.3 (10**)	GSK Bio*, Rixensart
CS-specific CMI		ICS	In-house ICS	% cytokine positive cells		KEMRI and GSK Bio*

* or designated validated laboratory
** seroprotective level
† or equivalent
CMI: cell-mediated immunity
ELISA: Enzyme-linked Immunoabsorbent Assay
EIA: Enzyme Immunoassay
ICS: intra-cellular cytokine staining

Separation of serum from the blood samples will be performed at the investigator's center.

Hematology and biochemistry determination will be performed at the KEMRI laboratories. The acceptable reference ranges will be specified in an SOP at the investigator site and in the GSK central study file.

CS-specific CMI will be determined at KEMRI and at GSK Biologicals by intra-cellular cytokine staining (CD4, CD8, CD3, IFN-gamma and activation markers) and measured as frequency (percent) of cytokine positive cells.

DNA extraction, PCR and determination of P. falciparum genotypes (MOI) will be performed at the Walter Reed laboratory, Kisumu, Kenya. The determination of CSP polymorphic sequences will be done at HPA Malaria Reference Laboratory, LSHTM, UK. **Amended 13 June 2006**

Serological responses will be primarily measured by evaluating antibody responses to CS (anti-CS also referred to as anti-R32LR antibodies). Antibody levels will be measured by standard Enzyme Linked Immunosorbent Assay (ELISA) methodologies using R32LR antigen as coating antigen and a standard reference in order to assess the relative titer of each test sample. Results will be reported in EU/mL. Anti-HBs antibody levels will be measured by ELISA with a commercial AUSAB EIA kit from Abbott or equivalent.

Serum for antibody determination will be collected by venous blood sample (as specified at each visit in Sections 5.4 and 5.5). Samples for safety will be analyzed at the time they are collected.

Serum samples from each subject will be shipped to GSK Biologicals, Rixensart, Belgium.

Any serum not immediately used in antibody assays will be stored at -20°C or less and would only be used to assess the immune response to vaccination or to assess any potential toxicity of the vaccine (Assay details are provided in Appendix F).

Table 30 Summary of blood sampling timepoints/immunological assays

**Blood sampling timepoint**			**Test**	**No. subjects**	**Laboratory**	**Priority Rank**
Timing	Timepoint	Clinic Visit No.				
Dose 1	Day 0	2	Anti-CS antibodies	255	GSK Bio*	1
			Anti-HBs antibodies	255	GSK Bio*	3
			CMI	255	KEMRI/ GSK Bio/ WRAIR	2
Dose 3	Day 60	5	Anti-CS antibodies	255	GSK Bio*	1
Post Dose 3	Day 90	7	Anti-CS antibodies	255	GSK Bio*	1
			Anti-HBs antibodies	255	GSK Bio*	3
			CMI	255	KEMRI/ GSK Bio/ WRAIR	2
Post Dose 3	Month 6½	8	Anti-CS antibodies	255	GSK Bio*	1
			CMI	255	GSK Bio*	2
Post Dose 3	Month 12	9	Anti-HBs antibodies	255	GSK Bio*	2
			Anti-CS antibodies	255	GSK Bio*	1
			CMI	*255*	*KEMRI/ GSK Bio/ WRAIR*	*2*

*or designated laboratory
CMI: cell-mediated immunity

Amended 13 June 2006

DNA will be extracted from 200 μL of the EDTA blood using QIAmp DNA blood mini kit as described by the manufacturer (QIAGEN Inc., CA). DNA samples will be stored at -80 °C until use. Details for PCR analysis, determination of CSP polymorphic sequences, genotyping of msp-1 and msp-2 and detection of multiplicity of infection are provided in Appendix F. **Amended 13 June 2006**

6. INVESTIGATIONAL PRODUCTS AND ADMINISTRATION

6.1. Investigational products

The RTS,S/AS02A, RTS,S/AS01B vaccines to be used have been developed and manufactured by GSK Biologicals.

Rabipur is manufactured by Chiron Behring GmbH, Marburg, Germany.

The Quality Control Standards and Requirements for the candidate vaccines are described in separate release protocols and the required approvals have been obtained.

Commercial vaccines are assumed to comply with the specifications given in the manufacturer's Summary of Product Characteristics.

Refer to Appendix G for details of vaccine supplies.

6.1.1. RTS,S/AS02A candidate malaria and hepatitis B vaccine

RTS,S antigen presentation:

- The lyophilized antigen pellet contains 62.5 µg of RTS,S with 25.2 mg of sucrose as cryoprotectant per 3 mL monodose vial. The pellet is reconstituted with adjuvant in liquid form and 0.5 mL of reconstituted vaccine contains 50 µg RTS,S.

AS02A adjuvant:

- AS02A contains 50 µg of MPL®, 50 µg QS21 (QS21 is a triterpene glycoside purified from the bark of *Quillaja saponaria*) and 250 µL of a proprietary oil-in-water emulsion in phosphate buffered saline per 0.5 mL, presented in prefilled syringes.

A dose of 0.5 mL will be delivered. The presentation of the reconstituted RTS,S/AS02A candidate malaria vaccine is an opaque milky liquid.

6.1.2. RTS,S/AS01B candidate malaria and hepatitis B vaccine

RTS,S antigen presentation:

- The lyophilized antigen pellet contains 62.5 µg of RTS,S with 25.2 mg of sucrose as cryoprotectant per 3 mL monodose vial. The pellet is reconstituted with adjuvant in liquid form and 0.5 mL of reconstituted vaccine contains 50 µg RTS,S.

AS01B adjuvant:

- AS01B contains 50 µg of MPL®, 50 µg QS21 (QS21 is a triterpene glycoside purified from the bark of *Quillaja saponaria*) in a suspension of liposomes in phosphate buffered saline per 0.5 mL and is presented in 3 mL monodose vials.

A dose of 0.5 mL will be delivered. The presentation of the reconstituted RTS,S/AS01B candidate malaria vaccine is an opalescent liquid.

6.1.3. Rabipur Rabies Vaccine

Rabipur, is a sterile freeze-dried vaccine obtained by growing the fixed-virus strain Flury LEP in primary cultures of chicken fibroblasts.

The potency of one dose (1.0 mL) Rabipur is at least 2.5 IU of rabies antigen. Rabipur is a white, freeze-dried vaccine for reconstitution with the diluent prior to use; the reconstituted vaccine is a clear to slightly opaque, colorless suspension.

A dose of 1.0 mL will be delivered. The presentation of the reconstituted vaccine is as a clear or slightly opaque suspension.

6.2. Dosage and administration

6.2.1. RTS,S/AS02A (0.5 mL dose)

RTS,S/AS02A will be supplied such that the reconstituted vaccine volume will provide a 0.5 mL adult dose. One 0.5 mL dose will be aspirated from each vial and used.

Disinfect top of vaccine vial (pellet) with alcohol swabs and let dry. Inject complete contents of one PFS of diluent into vial of lyophilized vaccine. Remove and discard the syringe and needle under appropriate safety precautions. The pellet is then dissolved by gently shaking the vial. Wait for 1 minute to ensure complete dissolution of vial contents before withdrawing a sufficient volume to provide a 0.5 mL dose (volume required for RTS,S/AS02A) of the reconstituted vaccine solution using a fresh needle and syringe for injection. The reconstituted vaccine should be administered by slow IM injection, using a 25G needle with length of 1 inch (25 mm), in the deltoid of the arm within 4 hours of reconstitution (storage at 2°C to 8°C).

6.2.2. RTS,S/AS01B (0.5 mL dose)

RTS,S/AS01B will be supplied such that the reconstituted vaccine volume will provide a 0.5 mL adult dose. One 0.5 mL dose will be aspirated from each vial and used.

Disinfect top of vaccine vial (pellet) and adjuvant vial with alcohol swabs and let dry. Aspirate the contents of the adjuvant vial in a syringe and inject adjuvant into the vial of lyophilized antigen. Remove and discard the syringe and needle under appropriate safety precautions. The pellet is then dissolved by gently shaking the vial. Wait for 1 minute to ensure complete dissolution of vial contents before withdrawing a sufficient volume to provide a 0.5 mL dose (volume required for RTS,S/AS01B) of the reconstituted vaccine solution using a fresh needle and syringe for injection. The reconstituted vaccine should be administered by slow IM injection, using a 25G needle with length of 1 inch (25 mm), in the deltoid of the arm within 4 hours of reconstitution (storage at 2°C to 8°C).

6.2.3. Rabipur rabies vaccine (1.0 mL dose)

Disinfect top of vaccine vial with alcohol swabs and let dry. Inject the entire contents of the diluent ampoule into the vaccine vial. Keeping the syringe and needle in place, the freeze-dried vaccine is dissolved by gently shaking the vial. Wait for 1 minute to ensure complete dissolution of vial contents before withdrawing a sufficient volume to provide a 1.0 mL dose still using the original needle and original syringe. The original needle should then be replaced with a fresh needle for IM injection. The reconstituted vaccine should be used immediately.

6.3. Storage

ALL VACCINE VIALS/PRE-FILLED SYRINGES (RTS,S, and Rabies antigen), AND ADJUVANTS (AS02A and AS01B water for injection) MUST BE STORED IN THE REFRIGERATOR (+2°C to +8°C) AND MUST NOT BE FROZEN.

All vaccine/adjuvant/water for injection vials will be stored in a safe and locked place with no access for unauthorized personnel. Storage temperature will be monitored daily, according to SOPs at the investigator's site. An alarm system and a back-up refrigerator will be available in case of power failure/breakdown.

The study monitor must be contacted if the cold chain is broken (e.g. vaccines become frozen or refrigeration fails).

Storage conditions for transport of vaccines from country medical department or dispatch center to study sites or between sites are described in Appendix D.

6.4. Treatment allocation and randomization

6.4.1. Randomization of supplies

A randomization list will be generated at GSK Biologicals, Rixensart, using a standard SAS® (Statistical Analysis System) program and will be used to assign the vaccines to treatment numbers. A 1:1:1 randomization of RTS,S/AS02A : RTS,S/AS01B : Rabipur will be used throughout the study.

6.4.2. Randomization of subjects

Subjects will be allocated sequentially to treatment numbers in the order that they present for vaccination.

6.5. Method of blinding and breaking the study blind

Data pertaining to the investigational products will be collected in a double blinded (observer blinded) manner. 'Double blinded (observer blinded)' means that the recipient of the investigational product as well as those responsible for the evaluation of safety, immunogenicity and efficacy endpoints will all be unaware which treatment (RTS,S/AS02A, RTS,S/AS01B or Rabipur) was administered to a particular subject. The only study staff aware of the vaccine assignment for RTS,S/AS02A, RTS,S/AS01B or Rabipur will be those responsible for the preparation and administration of investigational product; these staff will play no other role in the study.

Code break envelopes, for each study enrolled subject and associating each treatment number with a specific vaccine, will be kept in a safe and locked place with no access for unauthorized personnel by the Local Safety Monitor in Kenya as well as by Central Safety at GlaxoSmithKline Biologicals, Rixensart.

If deemed necessary for reasons such as safety, the Local Safety Monitor in Kenya as well as GSK Biologicals Central Safety will unblind the specific enrolled subject without revealing the study blind to the investigators.

One interim analysis will be conducted during the study: an analysis of Safety and Immunogenicity post Clinic Visit 7. To maintain the blind the interim analysis will be

performed by a statistician independent of the GSK Biologicals and WRAIR clinical teams.

A formal reporting and analysis plan (RAP) will be developed and agreed upon by the PI, the Sponsor (USAMMDA) and GSK Biologicals prior to unblinding. Once the study is completed and the GSK Biologicals reference database locked, GSK Biologicals will be responsible for initiating the execution of the statistical analysis plan in collaboration with WRAIR and breaking the blind.

GSK Biologicals' policy (incorporating ICH E2A guidance, EU Clinical Trial Directive and Federal Regulations) is to unblind any serious adverse event (SAE) report associated with the use of the investigational product, which is unexpected and attributable/suspected, prior to regulatory reporting. The Clinical Safety physician is responsible for unblinding the treatment assignment in accordance with specified time frames for expedited reporting of SAEs (Refer to Section 8.8).

6.6. Replacement of unusable vaccine doses

Additional vaccine doses will be provided to replace those that are unusable (see Appendix G for details of supplies).

In addition to the vaccine doses provided for the planned number of enrolled subjects, 3% additional doses will be supplied. In case a vaccine dose is broken or unusable, the investigator should replace it with a replacement vaccine dose. If a vaccine dose needs replacement, the envelope with the corresponding treatment number will designate the replacement without unblinding the study using a coded letter system. Although the sponsor, USAMMDA, and GSK Biologicals need not be notified immediately in these cases, documentation of the use of the replacement vaccine must be recorded by the investigator on the vaccine administration page of the CRF and on the vaccine accountability form.

6.7. Packaging

See Appendix G.

6.8. Vaccine accountability

See Appendix G.

6.9. Concomitant medication/treatment

At each study visit/contact, the investigator should question the enrolled subject about any medication(s) taken.

All antipyretic, analgesic, and antibiotic and administered at ANY time during the period starting with administration of each dose and ending 30 days after each dose are to be recorded with generic name of the medication (trade names are allowed for combination

drugs, i.e., multi-component drugs), medical indication, total daily dose, route of administration, start and end dates of treatment.

All antimalarial drugs administered from the beginning of the study until the end of the ADI period (i.e. 4 months post Dose 3) are to be recorded with generic name of the medication (trade names are allowed for combination drugs, i.e., multi-component drugs), medical indication, total daily dose, route of administration, start and end dates of treatment.

Any treatments and/or medications which are listed as elimination criteria in Section 4.5, e.g., any immunoglobulins, other blood products and any immune modifying drugs administered within three months preceding the first dose or at any time during the study period are to be recorded with generic name of the medication (trade names are allowed for combination drugs only), medical indication, total daily dose, route of administration, start and end dates of treatment. Refer to Sections 4.4 and 4.5.

Any vaccine not foreseen in the study protocol administered in the period beginning 30 days preceding each dose and ending 30 days after each dose is to be recorded with trade name, route of administration and date(s) of administration. Refer to Sections 4.4 and 4.5.

Any concomitant medication administered prophylactically in anticipation of reaction to the vaccination must be recorded in the CRF with generic name of the medication (trade names are allowed for combination drugs only), total daily dose, route of administration, start and end dates of treatment and coded as 'Prophylactic'.

7. HEALTH ECONOMICS

Not applicable

8. ADVERSE EVENTS AND SERIOUS ADVERSE EVENTS

The investigator is responsible for the detection and documentation of events meeting the criteria and definition of an adverse event (AE) or serious adverse event (SAE) as provided in this protocol. During the study, when there is a safety evaluation, the investigator or site staff will be responsible for detecting AEs and SAEs, as detailed in this section of the protocol. In addition, for this study the investigator is responsible for the timely reporting of unanticipated problems (refer to Section 8.3).

Each subject will be instructed to contact the investigator immediately should they manifest any signs or symptoms they perceive as serious.

8.1. Definition of an adverse event

An AE is any untoward medical occurrence in a clinical investigation subject, temporally associated with the use of a medicinal product, whether or not considered related to the medicinal product.

An AE can therefore be any unfavorable and unintended sign (including an abnormal laboratory finding), symptom, or disease (new or exacerbated) temporally associated with the use of a medicinal product.

Examples of an AE include:

- Exacerbation of a chronic or intermittent pre-existing condition including either an increase in frequency and/or intensity of the condition.
- New conditions detected or diagnosed after investigational product administration even though it may have been present prior to the start of the study.
- Signs, symptoms, or the clinical sequelae of a suspected overdose of either investigational product or a concurrent medication (overdose per se should not be reported as an AE/SAE).
- Signs, symptoms temporally associated with vaccine administration.

AEs may include pre- or post-treatment events that occur as a result of protocol-mandated procedures (i.e., invasive procedures, modification of subject's previous therapeutic regimen).

N.B. AEs to be recorded as endpoints (solicited events) are described in Section 8.4.1. All other AEs will be recorded as **UNSOLICITED AEs**.

Example of events to be recorded in the medical history section of the CRF:

Pre-existing conditions or signs and/or symptoms present in a subject prior to the start of the study (i.e. prior to the first study procedure) should be recorded in the medical history section of the subject's CRF.

8.2. Definition of a serious adverse event

A serious adverse event (SAE) is any untoward medical occurrence that:

a. results in death,

b. is life-threatening,

NOTE: The term 'life-threatening' in the definition of 'serious' refers to an event in which the subject was at risk of death at the time of the event. It does not refer to an event, which hypothetically might have caused death, if it were more severe.

c. requires hospitalization or prolongation of existing hospitalization,

NOTE: In general, hospitalization signifies that the subject has been detained (usually involving at least an overnight stay) at the hospital or emergency ward for observation and/or treatment that would not have been appropriate in the physician's office or out-patient setting. Complications that occur during hospitalization are AEs. If a complication prolongs hospitalization or fulfils any other serious criteria, the event is serious. When in doubt as to whether "hospitalization" occurred or was necessary, the AE should be considered serious.

Hospitalization for elective treatment of a pre-existing condition that did not worsen from baseline is not considered an AE.

d. results in disability/incapacity, or

NOTE: The term disability means a substantial disruption of a person's ability to conduct normal life functions. This definition is not intended to include experiences of relatively minor medical significance such as uncomplicated headache, nausea, vomiting, diarrhea, influenza, and accidental trauma (e.g. sprained ankle) which may interfere or prevent everyday life functions but do not constitute a substantial disruption.

e. is a congenital anomaly/birth defect in the offspring of a study subject.

f. Medical or scientific judgment should be exercised in deciding whether reporting is appropriate in other situations, such as important medical events that may not be immediately life-threatening or result in death or hospitalization but may jeopardize the subject or may require medical or surgical intervention to prevent one of the other outcomes listed in the above definition. These should also be considered serious. Examples of such events are invasive or malignant cancers, intensive treatment in an emergency room or at home for allergic bronchospasm, blood dyscrasias or convulsions that do not result in hospitalization.

8.3. Definition of an Unanticipated Problem

Unanticipated problems are those problems that are not specifically described in the protocol or other study documents. They are problems that involve risks to subjects and others, although the risk may not result in bodily harm. For example, misplacing a subject's study records containing identifiable private information results in the risk of breach of confidentiality. Confidentiality may or may not be breached, but either way this would be a reportable event. Another example would be administering the wrong agent to a subject at one time point in a series of vaccinations. Risks to others must also be reported. For example, an inadvertent exposure of a household contact in a smallpox vaccine trial would be a reportable event. Problems resulting in risks to members of the research team are also reportable. Reporting will be carried out with forms supplied by the HSRRB. Reports by the investigator should be promptly sent to the US Army Medical Research and Materiel Command's Human Subjects Research Review Board and to the medical monitor at GSK Biologicals; details of the addresses to which reports should be sent are given in Section 8.8.3. Appropriate supporting documents, such as laboratory reports, pathology reports, and discharge summaries should be submitted with the unanticipated problem report. The medical monitor should indicate whether she concurs with the details provided in the investigator's report. A medical monitor report that comments on the outcomes of the event and the relationship of the event to participation in the study must be submitted to the HSRRB within ten calendar days. Follow-up reports should be submitted until resolution of the unanticipated problem.

8.4. Clinical laboratory parameters and other abnormal assessments qualifying as adverse events and serious adverse events

Abnormal laboratory findings (e.g., clinical chemistry, hematology, urinalysis) or other abnormal assessments (e.g. blood film) that are judged by the investigator to be clinically significant will be recorded as AEs or SAEs if they meet the definition of an AE, as defined in Section 8.1 or SAE, as defined in Section 8.2. Clinically significant abnormal laboratory findings or other abnormal assessments that are detected during the study or are present at baseline and significantly worsen following the start of the study will be reported as AEs or SAEs. However, clinically significant abnormal laboratory findings or other abnormal assessments that are associated with the disease being studied, unless judged by the investigator as more severe than expected for the subject's condition, or that are present or detected at the start of the study and do not worsen, will not be reported as AEs or SAEs.

The investigator will exercise his or her medical and scientific judgment in deciding whether an abnormal laboratory finding or other abnormal assessment is clinically significant.

8.5. Time period, frequency, and method of detecting adverse events and serious adverse events

All AEs occurring within 30 days following administration of each dose of vaccine must be recorded on the Adverse Event form in the subject's CRF, irrespective of severity or whether or not they are considered vaccination-related.

The standard time period for collecting and recording SAEs will begin at randomization or the first receipt of vaccine and will end 30 days following administration of the last dose of study vaccine for each subject. See Section 8.8 for instructions for reporting and recording SAEs.

Additionally, in order to fulfil international reporting obligations, SAEs that are related to study participation (e.g. procedures, invasive tests, a change from existing therapy) or are related to a concurrent medication will be collected and recorded from the time the subject consents to participate in the study until she/he is discharged.

All AEs either observed by the investigator or one of his clinical collaborators or reported by the subject spontaneously or in response to a direct question will be evaluated by the investigator. AEs not previously documented in the study will be recorded in the Adverse Event form within the subject's CRF. The nature of each event, date and time (where appropriate) of onset, outcome, intensity and relationship to vaccination should be established. Details of any corrective treatment should be recorded on the appropriate page of the CRF. Refer to Section 6.9.

As a consistent method of soliciting AEs, the subject should be asked a non-leading question such as:

"Have you felt different in any way since receiving the vaccine or since the previous visit?"

N.B. The investigator should record only those AEs having occurred within the time frame defined above.

AEs already documented in the CRF, i.e. at a previous assessment, and designated as "not recovered/not resolved" or "recovering/resolving" should be reviewed at subsequent visits, as necessary. If these have resolved, the documentation in the CRF should be completed.

N.B. If an AE changes in frequency or intensity during the specified reporting period, a new record of the event will be entered.

When an AE/SAE occurs, it is the responsibility of the investigator to review all documentation (e.g., hospital progress notes, laboratory, and diagnostics reports) relative to the event. The investigator will then record all relevant information regarding an AE/SAE on the CRF or SAE Report Form as applicable. It is not acceptable for the investigator to send photocopies of the subject's medical records to GSK Biologicals in lieu of the appropriate completed AE/SAE pages. However, there may be instances when copies of medical records for certain cases are requested by GSK Biologicals. In this instance, all subject identifiers will be blinded on the copies of the medical records prior to submission to GSK Biologicals.

The investigator will attempt to establish a diagnosis of the event based on signs, symptoms, and/or other clinical information. In such cases, the diagnosis should be documented as the AE/SAE and not the individual signs/symptoms.

8.5.1. Solicited adverse events

Local (injection site) adverse events

- Pain at injection site
- Swelling at injection site

General adverse events

- Fever (defined as oral temperature ≥ 37.5°C)
- Fatigue
- Gastrointestinal problems
- Headache
- Joint pain
- Muscle ache

The visiting field worker will record these adverse events according to detailed SOPs available on study site during the field worker visits.

N.B. Temperature will be recorded on days 1 to 6 by the field worker or Principal Investigator. Should additional temperature measurements be performed at other times of day, the highest temperature will be recorded.

8.6. Evaluating adverse events and serious adverse events

8.6.1. Assessment of intensity

Intensity of the following AEs will be assessed as described:

Table 31 Intensity scales for solicited symptoms in adults

Adults		
**Adverse Event**	**Intensity grade**	**Parameter**
Pain at injection site	0	Absent
	1	Painful on touch
	2	Painful when limb is moved
	3	Pain that prevents normal activity
Swelling at injection site		Record greatest surface diameter in mm
Fever*		Record temperature in °C
Headache	0	Normal
	1	Headache that is easily tolerated
	2	Headache that interferes with normal activity
	3	Headache that prevents normal activity
Fatigue	0	Normal
	1	Fatigue that is easily tolerated
	2	Fatigue that interferes with normal activity
	3	Fatigue that prevents normal activity
Gastrointestinal symptoms (nausea, vomiting, diarrhoea and/or abdominal pain)	0	Gastrointestinal symptoms normal
	1	Gastrointestinal symptoms that are easily tolerated
	2	Gastrointestinal symptoms that interfere with normal activity
	3	Gastrointestinal symptoms that prevent normal activity
Joint pain	0	Absent
	1	Joint pain that is easily tolerated
	2	Joint pain that interferes with normal activity
	3	Joint pain that prevents normal activity
Muscle ache	0	Absent
	1	Muscle ache that is easily tolerated
	2	Muscle ache that interferes with normal activity
	3	Muscle ache that prevents normal activity

*Fever is defined as oral temperature ≥37.5°C

The maximum intensity of local injection site swelling will be scored at GSK Biologicals as follows:

0 : None
1 : > 0 to 20 mm
2 : > 20 to 50 mm
3 : > 50 mm

The maximum intensity of fever will be scored at GSK Biologicals as follows:

0 : < 37.5°C
1 : 37.5 – 38.0°C
2 : > 38 – 39.0°C
3 : > 39.0°C

Table 32 Intensity scales for monitoring of hepatic and renal function

Creatinine (Males and Females)	
Normal range	0.45 to 1.5 mg/dL
Grade 1	1.1 to 1.5 mg/dL
Grade 2	1.6 to 2.0 mg/dL
Grade 3	2.1 to 2.5 mg/dL
**ALT**	
Males	Normal Range: < 60 IU/mL
Females	Normal Range: < 40 IU/mL
**Abnormal ALT gradings**	
Grade 1	1.1 to 2.5 times the upper limit of normal
Grade 2	2.6 to 5.0 times the upper limit of normal
Grade 3	5.1 to 10.0 times the upper limit of normal

Table 33 Complete blood count: Below normal range values

Hemoglobin	<11 g/dL (males) or < 9.5 g/dL (females)
Platelet count	< 77 000 per mm^3
Absolute lymphocyte count	< 1 $x10^3/\mu L$
Total white cell count	< 3 $x10^3/\mu L$ (males) or < 2.5 x $10^3/\mu L$ (females)

The investigator will make an assessment of intensity for all other AEs, i.e. unsolicited symptoms, including SAEs reported during the study. The assessment will be based on the investigator's clinical judgment. The intensity of each AE and SAE recorded in the CRF or SAE Report Form, as applicable, should be assigned to one of the following categories:

1 (mild) = An AE which is easily tolerated by the subject, causing minimal discomfort and not interfering with everyday activities.

2 (moderate) = An AE which is sufficiently discomforting to interfere with normal everyday activities.

3 (severe) = An AE which prevents normal, everyday activities (in adults, such an AE would, for example, prevent attendance at work and would necessitate the administration of corrective therapy).

An AE that is assessed as Grade 3 (severe) should not be confused with a SAE. Grade 3 is a category utilized for rating the intensity of an event; and both AEs and SAEs can be assessed as Grade 3. An event is defined as 'serious' when it meets one of the pre-defined outcomes as described in Section 8.2.

8.6.2. Assessment of causality

The investigator is obligated to assess the relationship between investigational product and the occurrence of each AE/SAE. The investigator will use clinical judgment to determine the relationship. Alternative causes, such as natural history of the underlying diseases, concomitant therapy, other risk factors and the temporal relationship of the event to the investigational product will be considered and investigated. The investigator will also consult the Investigator Brochure and/or Product Information, for marketed products, in the determination of his/her assessment.

There may be situations when a SAE has occurred and the investigator has minimal information to include in the initial report to the study contacts for reporting of a serious adverse event (refer to Section 8.8.2). However, it is very important that the investigator always makes an assessment of causality for every event prior to transmission of the SAE Report Form to the study contacts for reporting of a serious adverse event. The investigator may change his/her opinion of causality in light of follow-up information, amending the SAE Report Form accordingly. The causality assessment is one of the criteria used when determining regulatory reporting requirements.

In case of concomitant administration of multiple vaccines, it may not be possible to determine the causal relationship of general AEs to the individual vaccines administered. The investigator should, therefore, assess whether the AE could be causally related to vaccination rather than to the individual vaccines.

All solicited local (injection site) reactions will be considered causally related to vaccination. Causality of all other AEs should be assessed by the investigator using the following question:

Is there a reasonable possibility that the AE may have been caused by the investigational product ?

NO	:	The AE is not causally related to administration of the study vaccines. There are other, more likely causes and administration of the study vaccines is not suspected to have contributed to the AE.
YES	:	There is a reasonable possibility that the vaccines contributed to the AE.

Non-serious and serious AEs will be evaluated as two distinct events. If an event meets the criteria to be determined "serious" (see Section 8.2 for definition of serious adverse event), it will be examined by the investigator to the extent to be able to determine ALL contributing factors applicable to each serious adverse event.

Other possible contributors include:

- Medical history
- Other medication
- Protocol required procedure
- Other procedure not required by the protocol
- Lack of efficacy of the vaccine(s), if applicable
- Erroneous administration
- Other cause (specify).

8.7. Follow-up of adverse events and serious adverse events and assessment of outcome

After the initial AE/SAE report, the investigator is required to proactively follow each subject and provide further information to study contacts for reporting of a serious adverse event (refer to Section 8.8.2) on the subject's condition.

All AEs and SAEs documented at a previous visit/contact and designated as not recovered/not resolved or recovering/resolving will be reviewed at subsequent visits/contacts.

Investigators will follow-up subjects:

- with SAEs or subjects withdrawn from the study as a result of an AE, until the event has resolved, subsided, stabilized, disappeared, the event is otherwise explained, or the subject is lost to follow-up;
- or, in the case of other non-serious AEs, until they complete the study or they are lost to follow-up.

Clinically significant laboratory abnormalities will be followed up until they have returned to normal, or a satisfactory explanation has been provided. Additional information (including but not limited to laboratory results) relative to the subsequent course of such an abnormality noted for any subject must be made available to the Study Monitor.

The Sponsor, USAMMDA, Kenya National Ethical Review Committee and GSK Biologicals may request that the investigator perform or arrange for the conduct of supplemental measurements and/or evaluations to elucidate as fully as possible the nature and/or causality of the AE or SAE. The investigator is obliged to assist. If a subject dies during participation in the study or during a recognized follow-up period, study contacts for reporting of a serious adverse event will be provided with a copy of any available post-mortem findings, including histopathology.

New or updated information will be recorded on the originally completed SAE Report Form, with all changes signed and dated by the investigator. The updated SAE report

form should be resent to GSK Biologicals within 24 hours of receipt of the follow-up information as outlined in Section 8.8.1.

Outcome of any non-serious AE occurring within 30 days post-vaccination (i.e. unsolicited AE) or any SAE reported during the entire study will be assessed as:

- Recovered/resolved
- Not recovered/not resolved
- Recovering/resolving
- Recovered with sequelae/resolved with sequelae
- Fatal (SAEs only).

8.8. Prompt reporting of serious adverse events and unanticipated problems to the study contacts for reporting of a serious adverse event

8.8.1. Time frames for submitting serious adverse event reports to GSK Biologicals and USAMMDA

SAEs will be reported promptly to the study contacts for reporting of a serious adverse event (refer to Section 8.8.2) once the investigator determines that the event meets the protocol definition of an SAE. The investigator or designate will fax the SAE reports to the study contacts for reporting of a serious adverse event **WITHIN 24 HOURS OF HIS/HER BECOMING AWARE OF THESE EVENTS**. Additional or follow-up information relating to the initial SAE report is also to be reported to the study contacts for reporting of a serious adverse event within 24 hours of receipt of such information.

8.8.2. Completion and transmission of serious adverse event reports to the study contacts for reporting of a serious adverse event

Once an investigator becomes aware that a SAE has occurred in a study subject, she/he will report the information to study contacts for reporting of a serious adverse event (refer to Section 8.8.2) within 24 hours as outlined in Section 8.8.1. The SAE Report Form will always be completed as thoroughly as possible with all available details of the event, signed by the investigator (or designee), and forwarded to study contacts for reporting of a serious adverse event within the designated time frames. If the investigator does not have all information regarding an SAE, he/she will not wait to receive additional information before notifying study contacts for reporting of a serious adverse event of the event and completing the form. The form will be updated when additional information is received and forwarded to study contacts for reporting of a serious adverse event **WITHIN 24 HOURS** as outlined in Section 8.8.1.

The investigator will always provide an assessment of causality at the time of the initial report as described in Section 8.6.2.

Facsimile (Fax) transmission of the SAE Report Form is the preferred method to transmit this information to the Study Contacts for Reporting SAEs. In rare circumstances and in the absence of facsimile equipment, notification by telephone is acceptable, with a copy of the SAE Report Form sent by overnight mail. Initial notification via the telephone does not replace the need for the investigator to complete and sign the SAE Report Form within 24 hours as outlined in Section 8.8.1.

In the event of a death determined by the investigator to be related to vaccination, sending of the fax must be accompanied by telephone call to the Study Contacts for Reporting SAEs.

**Study contacts for reporting of a Serious Adverse Event**	
**Central Study Coordinator**	**HSRRB**
Isabelle Ramboer,<br>Central Study Coordinator,<br>GlaxoSmithKline Biologicals,<br>Rue de l'Institut 89, 1330 Rixensart,<br>Belgium.<br>Tel: +32.2.656.68.20<br>Fax: +32.2.656.80.44<br>email: isabelle.ramboer@gskbio.com	US Army Medical Research and Materiel Command's Human Subjects Research Review Board,<br>USA.<br>Tel: +001.310.619.21.65<br>Fax: +001.319.619.78.03<br>email: hsrrb@amedd.army.mil
**USAMRMC Deputy for Reporting Serious Adverse Events**	**WRAIR Office of Research Management**
US Army Medical Research and Material Command,<br>ATTN: MCMR-ZB P,<br>504 Scott Street,<br>Fort Detrick, MD 2170 2-5012,<br>USA.<br>Tel: +001.301.619.2165/6<br>Fax: +001.301.619.7803<br>email: hsrrb@amedd.army.mil	Walter Reed Army Institute of Research<br>Office of Research Management<br>503 Robert Grant Avenue<br>Silver Spring, MD 20910-7500<br>USA<br><br>Tel: +001.301.319.9940<br>Fax: +001.301.319.9961<br>email: jody.ference@na.amedd.army.mil
**Kenya National Ethical Review Committee**	
The Chairman,<br>Kenya National Ethical Review Committee,<br>c/o Kenya Medical Research Institute,<br>PO Box 54840, Nairobi, Kenya.<br>Tel: +254.20.72.25.41<br>email: mwasunna@nairobi.mimcom.net	

**Back-up Study Contact at GSK Biologicals for Reporting Serious Adverse Events Clinical Safety Vaccines**
GSK Biologicals Clinical Safety Physician,<br>GlaxoSmithKline Biologicals,<br>Rue de l'Institut 89, 1330 Rixensart,<br>Belgium.<br>Tel: +32.2.656.87.98<br>Fax: +32.2.656.80.09<br>email: rix.ct-safety-vac-@gskbio.com<br>Mobile phone for 7/7 day availability: +32.477.40.47.13

8.8.3. Reporting of unanticipated problems

Unanticipated problems (refer to Section 8.3) involving risk to volunteers or others, serious adverse events related to participation in the study and all volunteer deaths should be promptly reported by phone, by email, or by facsimile to the U.S. Army Medical Research and Materiel Command's Human Subjects Research Review Board and to the GSK Biologicals Medical Monitor (see below for contact details). A complete written report should follow the initial notification. In addition to the methods above, the complete report can be sent to the addresses given below.

**U.S. Army Medical Research and Materiel Command's Human Subjects Research Review Board**	**Medical Monitor**
U.S. Army Medical Research and Materiel Command's Human Subjects Research Review Board,<br>ATTN: MCMR-ZB-P, 504 Scott Street,<br>Fort Detrick, Maryland 21702-5012,<br>USA.<br>Tel: +1.301.619.21.65<br>Fax: +1. 301.619.78.03<br>email: hsrrb@amedd.army.mil	Amanda Leach,<br>Clinical Development Manager,<br>GlaxoSmithKline Biologicals,<br>Rue de l'Institut 89, 1330 Rixensart,<br>Belgium.<br>Tel: +32.2.656.77.88<br>Fax: +32.2.656.61.60<br>email: amanda.leach@gskbio.com

8.9. Regulatory reporting requirements for serious adverse events

The investigator will promptly report all SAEs to study contacts for reporting of a serious adverse event (refer to Section 8.8.2) in accordance with the procedures detailed in Section 8.8. The Sponsor, USAMMDA has a legal responsibility to promptly notify, as appropriate, both the local regulatory authority and other regulatory agencies about the safety of a product under clinical investigation. Prompt notification of SAEs by the investigator to the Study Contacts for Reporting SAEs is essential so that legal obligations and ethical responsibilities towards the safety of other subjects are met.

The investigator, or responsible person according to local requirements, will comply with the applicable local regulatory requirements related to the reporting of SAEs to the IRB/IEC and, if required, to the applicable government authority.

Investigator safety reports are prepared according to FDA requirements by the Sponsor (USAMMDA) and are forwarded to investigators as necessary. An investigator safety report is prepared for a SAE(s) that is both attributable to investigational product and unexpected. The purpose of the report is to fulfill specific regulatory and Good Clinical Practice (GCP) requirements, regarding the product under investigation.

An investigator who receives an investigator safety report describing a SAE(s) or other specific safety information (e.g., summary or listing of SAEs) from GSK Biologicals and/or USAMMDA will file it with the Investigator Brochure or other appropriate study documentation and will notify the IRB or IEC, if appropriate according to local requirements.

8.10. Post study adverse events and serious adverse events

A post-study AE/SAE is defined as any event that occurs outside of the AE/SAE detection period defined in Section 8.4. Investigators are not obligated to actively seek AEs or SAEs in former study participants.

However, if the investigator learns of any SAE, including a death, at any time after a subject has been discharged from the study, and he/she considers the event reasonably related to the investigational product, the investigator will promptly notify the Study Contacts for Reporting SAEs.

8.11. Pregnancy

Subjects who become pregnant during the study (up to 30 days after receiving the last vaccine dose) must not receive additional doses of study vaccine investigative product but may continue other study procedures at the discretion of the investigator.

The investigator, or his designee, will collect pregnancy information on any subject who becomes pregnant while participating in this study. The investigator, or his designee, will record pregnancy information on the Pregnancy Report Form and submit it to study contacts for reporting of a serious adverse event (refer to Section 8.8.2) within 24 hours of learning of a subject's pregnancy. The subject will be followed to determine the outcome of the pregnancy. At the end of the pregnancy, whether that be full-term or prematurely, information on the status of the mother and child will be forwarded to study contacts for reporting of a serious adverse event. Generally, follow-up will be no longer than six to eight weeks following the estimated delivery date.

While pregnancy itself is not considered an AE or SAE, any pregnancy complication or elective termination of a pregnancy for medical reasons will be recorded as an AE or a SAE, as described in Section 8.1 and 8.2, and will be followed as described in Section 8.7.

A spontaneous abortion is always considered to be a SAE and will be reported as described in Section 8.8. Furthermore, any SAE occurring as a result of a post-study pregnancy AND considered reasonably related in time to receipt of the investigational product by the investigator, will be reported to study contacts for reporting of a serious adverse event as described in Section 8.10. While the investigator is not obligated to actively seek this information from former study participants, he may learn of a pregnancy through spontaneous reporting.

Information on pregnancies identified during the screening phase/prior to vaccine administration does not need to be collected; this information need not be communicated to safety.

8.12. Treatment of adverse events

Treatment of any adverse event is at the sole discretion of the investigator and according to current good medical practice. Any medication administered for the treatment of an AE should be recorded in the subject's CRF. Refer to Section 6.9.

9. SUBJECT COMPLETION AND WITHDRAWAL

9.1. Subject completion

A subject who returns for the concluding visit/is available for the concluding contact foreseen in the protocol is considered to have completed the study.

9.2. Subject withdrawal

Subjects who are withdrawn for AEs must be clearly distinguished from subjects who are withdrawn for other reasons. Investigators will follow subjects who are withdrawn as result of a SAE/AE until resolution of the event (see Section 8.7). Withdrawals will not be replaced.

9.2.1. Subject withdrawal from the study

From an analysis perspective, a 'withdrawal' from the study is any subject who did not come back for the concluding visit.

A subject qualifies as a 'withdrawal' from the study when no study procedure has occurred, no follow-up has been performed and no further information has been collected for this subject from the date of withdrawal/last contact.

Investigators will make an attempt to contact those subjects who do not return for scheduled visits or follow-up.

Information relative to the withdrawal will be documented on the Study Conclusion page of the CRF. The investigator will document whether the decision to withdraw from the

study was made by the subject or the investigator and which of the following possible reasons was responsible for withdrawal:

- serious adverse event
- non-serious adverse event
- protocol violation (specify)
- consent withdrawal, not due to an adverse event
- moved from the study area
- lost to follow-up
- other (specify).

9.2.2. Subject withdrawal from investigational product

A ‘withdrawal’ from the investigational product is any subject who does not receive the complete treatment, i.e. when no further planned dose is administered from the date of withdrawal. A subject withdrawn from the investigational product may not necessarily be withdrawn from the study as further study procedures or follow-up may be performed (safety or immunogenicity) if planned in the study protocol.

Information relative to premature discontinuation of the investigational product will be documented on the Vaccine Administration page of the CRF. The investigator will document whether the decision to discontinue further vaccination / treatment was made by the subject or the investigator and which of the following possible reasons was responsible for withdrawal:

- serious adverse event,
- non-serious adverse event,
- other (specify).

10. DATA EVALUATION: CRITERIA FOR EVALUATION OF OBJECTIVES

10.1. Primary Endpoint

10.1.1. Safety & Reactogenicity

- Occurrence of Grade 3 solicited or unsolicited general reactions after each vaccination during a seven day follow-up period (day of vaccination and 6 subsequent days)

10.2. Secondary endpoints

10.2.1. Safety

- Occurrence of SAEs until 4 months post Dose 3.
- Occurrence of unsolicited AEs after Dose 1, 2 and 3 of vaccine over a 30 day follow-up period (day of vaccination and 29 subsequent days)
- Occurrence of solicited general and local reactions over a 7 day follow-up period (day of vaccination and 6 subsequent days) after Dose 1, 2 and 3 of vaccine.
- Occurrence of parameters of hematological monitoring below normal range.
- Occurrence of parameters of biochemical monitoring above normal range

10.2.2. Immunogenicity

Endpoints assessed prior to vaccination, and 1 month post Dose 3:

- Anti HBs antibody titers

Endpoints assessed prior to vaccination,, 1 month post Dose 2, 1 month post Dose 3 and 4 months post Dose 3:

- Anti-CS antibody titers

10.2.3. Efficacy

- The time to first malaria infection (first recording of infection of asexual stage falciparum parasites detected by the active case detection or passive case detection) over a period starting 14 days after Dose 3 and extending for 14 weeks.
- The asexual *P. falciparum* parasitemia (prevalence and density) at 16 weeks post Dose 3

10.3. Tertiary endpoints

10.3.1. Safety

- The occurrence of SAEs from 4 months post Dose 3 until study conclusion

10.3.2. Immunogenicity

Endpoints assessed 10 months post Dose 3:

- Anti HBs antibody titers
- Anti-CS antibody titers

10.4. Exploratory endpoints:

10.4.1. Cell Mediated Immunity

Endpoint assessed prior to vaccination, 1 month post Dose 3 and 4 months post Dose 3.

- For each subject, the frequency of CS-specific CD4+ and CD8+ T-cells, as measured by intracellular cytokine staining.

10.4.2. Efficacy

- The percentage change in hemoglobin value between baseline and 16 weeks post Dose 3.

10.4.3. Parasite Genotyping

- ***Sequencing of the Th2R and Th3R epitope regions in parasite-positive individuals identified by CSP PCR amplification of Day 0 DNA samples.***
- ***DNA samples prepared at first microscopy confirmed infections identified after the third vaccination, used to determine the distribution of amino acid substitutions in the Th2R and Th3R sequence regions of the CSP gene.***
- ***DNA samples prepared at first microscopy confirmed infection identified after the third vaccination, used to determine the number of P. falciparum genotypes (multiplicity of infection) as determined by analysis of msp-1 and msp-2 genes.***

Amended 13 June 2006

10.5. Estimated sample size

10.5.1. Sample size for the Primary Objective

The primary objective will be assessed by comparing the proportion of subjects experiencing a Grade 3 solicited or unsolicited general reaction after each vaccination during a seven day follow-up period (day of vaccination and six subsequent days). In a previous study with RTS,S/AS02A in Africa (Malaria-008) the proportion affected was 10%. The proportion affected was higher in a recent study conducted in non-immune adults at WRAIR (Malaria-027); 39%. Therefore Table 34, below, has been generated for a wide range of hypothetical proportions of Grade 3 general symptoms.

Table 34 Potential of the study to detect a difference in the proportion affected by Grade 3 general symptoms assuming 90% power and a sample size of 255

Frequency of events for RTS,S/AS02A	Frequency of events for RTS,S/AS01B	Power to detect difference (75 per group) ($p < 0.05$)
5%	24%	90%
10%	33%	90%
20%	46%	90%
30%	57%	90%
40%	67%	90%
50%	77%	90%

10.5.2. Sample Size for the Secondary Objective for Safety

The data set will be examined, comparing the rates of SAEs at the Medical Dictionary for Regulatory Activities (MedDRA) preferred term level. SAEs are expected to occur infrequently and the study has therefore the power to detect only large differences in the frequencies of SAEs compared to control. Refer to Table 35.

Table 35 Potential of the study to detect a difference in the frequency of SAEs between groups of subjects vaccinated with candidate malaria vaccines compared to control

Frequency of events for Rabies vaccine	Frequency of events for RTS,S/AS02A or RTS,S/AS01B	Power to detect difference (75 per group) ($p < 0.05$)
1%	13%	80%
3%	17%	80%
5%	21%	80%
10%	29%	80%

10.5.3. Sample Size for the Secondary Objective for Proof-of-Concept

The minimum vaccine efficacy that a study of this size has the power to detect (i.e. vaccine vs. control) is shown in Table 36. This assumes an attack rate of 72%.

Table 36 Power of the study to detect vaccine efficacy (assuming an attack rate of 72%)

VE in malaria vaccine groups	Power ($p < 0.05$)
41%	80%
45%	90%

Table 37 shows the increase in VE of RTS,S/AS01B over RTS,S/AS02A that can be detected with 80% power. Historically, RTS,S/AS02A has demonstrated efficacy against infection in African adults in The Gambia of 34% (Malaria-005) and in children in Mozambique of 45% (Malaria-026).

Table 37 Increase in Vaccine Efficacy of RTS,S/AS01B over RTS,S/AS02A that can be detected with 80% power

VE in RTS,S/AS02A vs control	VE in RTS,S/AS01B vs control	Power (at a 5% level of significance)
30%	65%	80%
40%	71%	80%
50%	78%	80%

10.6. Study cohorts to be evaluated

10.6.1. Total Vaccinated cohort

The Total Vaccinated Cohort will include all vaccinated subjects for whom data are available. Thus, the total analysis of safety will include all subjects with at least one vaccine administration documented and the total analysis of immunogenicity/efficacy will include vaccinated subjects for whom data concerning immunogenicity/efficacy endpoint measures are available. The Total Vaccinated Cohort analysis will be performed per treatment actually administered.

10.6.2. According-To-Protocol (ATP) cohort for analysis of safety

The ATP cohort for analysis of safety will include all evaluable subjects;

- who have received at least one dose of study vaccine according to their random assignment
- have sufficient data to perform an analysis of safety (at least one vaccine dose with safety follow-up)
- for whom administration site of study vaccine is per protocol
- who have not received a vaccine not specified or forbidden in the protocol and for whom elimination criteria were not applied
- for whom the randomization code has not been broken except for when unblinding has been carried out by the SMG for Safety Analysis.
- who meet all eligibility criteria.

10.6.3. According To Protocol (ATP) cohort for analysis of immunogenicity

The ATP cohort for analysis of immunogenicity will include all evaluable subjects (i.e. those meeting all eligibility criteria, complying with the procedures defined in the protocol, with no elimination criteria during the study) for whom data concerning immunogenicity endpoint measures are available.

10.6.4. According To Protocol (ATP) cohort for analysis of proof-of-concept

The ATP cohort for analysis of proof-of-concept will include all evaluable subjects (i.e. those meeting all eligibility criteria, complying with the procedures defined in the protocol, with no elimination criteria during the study) for whom data concerning efficacy endpoint measures are available.

10.7. Derived and transformed data

- A subject seropositive for anti-CS antibody is a subject whose antibody titer is greater than or equal to the cut-off value (anti-CS ≥ 0.5 EU/mL).
- A subject seropositive for anti-HBs antibody is a subject whose antibody titer is greater than or equal to the cut-off value (anti-HBs ≥ 3.3 mIU/mL).
- A subject seroprotected for anti-HBs antibody is a subject whose antibody titer is greater than or equal to the seroprotective level (anti-HBs ≥ 10 mIU/mL).
- The Geometric Mean Titers (GMTs) calculations are performed by taking the anti-log of the mean of the log10 titer transformations. Antibody titers below the cut-off of the assay will be given an arbitrary value of half the cut-off for the purpose of GMT calculation.

For a given subject and a given immunogenicity measurement, missing or non-evaluable measurements will not be replaced. Therefore, an analysis will exclude subjects with missing or non-evaluable measurements.

For the analysis of solicited local/general symptoms, only subjects for which information on the solicited local/general symptom sheet is available for the considered study dose will be included in the analysis.

10.8. Final analyses

The final analysis will take place after 4 months post Dose 3 of vaccine (Month 6½). It will complement the interim analysis which takes place 1 month post Dose 3 (Day 90) to provide complete analysis on all primary and secondary endpoints. Section 10.8 describes the analyses to be carried out for the annex report (data up until 12 months post Dose 1).

10.8.1. Analysis of demographics/baseline characteristics

Demographic characteristics (age, gender, weight, height) of each study cohort will be tabulated.

The distribution of subjects enrolled will be tabulated as a whole and per group.

10.8.2. Analyses of safety

The analysis for safety will be conducted on the Total Cohort for Safety.

10.8.2.1. Primary objective

For the safety primary objective, the occurrence of Grade 3 solicited or unsolicited general reactions will be determined on the Total Vaccinated Cohort. The proportion of subjects with a Grade 3 solicited or unsolicted reaction, reported from study start until study conclusion (end of the double-blind phase) will be tabulated with exact 95% CI. Comparisons between groups will be done using Fisher's Exact Test.

10.8.2.2. Secondary objectives

The occurrence of SAEs will be determined on the Total Vaccinated Cohort. The proportion of subjects with an SAE, classified by the MedDRA preferred term level, reported from study start until study conclusion (end of the double-blind phase) will be tabulated with exact 95% CI. Comparisons between groups will be done using Fisher's Exact Test for each preferred term.

The occurrence of adverse events will be determined on the Total Vaccinated Cohort. The proportion of subjects with an AE, classified by the MedDRA preferred term level, reported from study start until study conclusion (end of the double-blind phase) will be tabulated with exact 95% CI. Comparisons between groups will be done using Fisher's Exact Test.

The percentage of subjects with at least one local adverse event (solicited and unsolicited), with at least one general adverse event (solicited and unsolicited) and with any adverse event during the solicited follow-up period will be tabulated with exact 95% CI after each vaccine dose and overall. The percentage of doses followed by at least one local adverse event (solicited and unsolicited), by at least one general adverse event (solicited and unsolicited) and by any adverse event will be tabulated, overall vaccination course, with exact 95% CI. Comparisons between groups will be done using Fisher's Exact Test. Similar tables will be generated for Grade 3 events, the relationship of the event to vaccination and for fever, temperature (in 0.5°C increments).

The percentage of subjects reporting each individual solicited local and general adverse event during the solicited follow-up period will be tabulated with exact 95% CI. The percentage of doses followed by each individual solicited local and general adverse event will be tabulated, overall vaccination course, with exact 95% CI. Comparisons between groups will be done using Fisher's Exact Test. Similar tables will be generated for Grade 3 events, the relationship of the event to vaccination and for fever, temperature (in 0.5°C increments).

For each sampling timepoint, the biochemical parameters (ALT, bilirubin, creatinine) above normal range and hematological parameters (hemoglobin, total WBC, platelets and absolute lymphocyte count) below normal range will be described. Similar tables will be generated for Grade 3 parameters (as determined from Table 32).

10.8.3. Analyses of immunogenicity

The primary analysis will be based on the ATP cohort for analysis of immunogenicity. If the percent of enrolled subjects excluded from this ATP cohort is more than 5%, a second analysis based on the Total Vaccinated cohort will be performed to complement the ATP analysis.

10.8.3.1. Anti-CS antibodies

The percentage of subjects with sero-positive levels of anti-CS (proportion of subjects with anti-CS antibody titers greater than or equal to 0.5 EU/mL) with 95% CI will be determined prior to vaccination, at 1 month post Dose 2, 1 Month post Dose 3 and 4 months post Dose 3. Antibody titers will be summarized by GMT with 95%CI. Antibody titers after the third dose will also be investigated using reverse cumulative curves.

10.8.3.2. Anti-HBs antibodies

The percentage of subjects with sero-positive levels of anti-HBs (proportion of subjects with anti-HBs antibody titers greater than or equal to 3.3 mIU/mL), the percentage of subjects with seroprotective levels of anti-HBs (≥10 mIU/mL) with 95% CI will be determined prior to vaccination at 1 Month post Dose 3. Antibody titers will be summarized by GMT with 95%CI. Antibody titers after the third dose will also be investigated using reverse cumulative curves.

10.8.4. Analyses of efficacy

10.8.4.1. Case Definitions

Infection is defined as *P. falciparum* asexual parasitemia > 0 detected on a scheduled ADI visit or by passive case detection.

10.8.4.2. Calculation of the time at risk

For the efficacy endpoint (time to first malaria infection), the time at risk will be counted in days, and expressed as person years at risk (days/365.25).

For each subject in the Total Cohort, time at risk begins 14 days after Dose 3 for those that received Dose 3 or 2½ months after Dose 1 (Day 75) otherwise. For the According to Protocol Cohort, time at risk begins 14 days after Dose 3.

The time at risk will end whenever one of the following conditions happen first: fits the case definition for malaria infection, loss of follow up, emigration from the study area, withdrawal, death, end of follow-up period (4 months post Dose 3).

The time at risk will take into account absences from the study area and anti-malaria drug therapy. Absences from the study area of 1 weeks or more will be recorded in multiples of 1 week. The date of departure will be documented. Similarly, if treatment for malaria is administered, the subject will not be considered susceptible to malaria infection for the

longest duration of the combination of drugs the subject could receive for this episode as follows: 28 days if received Coartem®, 7 days days if having received Malarone®, 7 days if having received Fansidar (sulfadoxine-pyrimethamine) and 7 days if having received quinine alone.

If an episode is detected during a period of time not counting for the time at risk it will not be included in the analysis, however a table will be presented showing the number of such episodes not included in the analysis.

Episodes will be included in the analysis only if the subject is identified properly showing his/her ID Card at the contact with the health facility and the study number is written in the OPD form. Only blood slides that complete the reading process according to SOP will be considered in the establishment of a malaria infection.

10.8.4.3. Vaccine efficacy

10.8.4.3.1. Vaccine efficacy compared to Rabies control vaccine

Time to first infection of *P. falciparum* will be examined using Kaplan-Meier curves for all groups. The distribution of both malaria vaccine groups will be compared with the Wilcoxon test (if efficacy varies with time) or the Log-rank test (if it does not). Vaccine Efficacy will be assessed using Cox regression models. Vaccine efficacy is defined as 1 *minus* R where R is the hazard ratio of the RTS,S/AS02A or RTS,S/AS01B group versus the Rabies Vaccine group (with 95% CI). Crude and adjusted estimates for covariates factors (see Section 10.6.3.3) will be presented.

Cox regression assumes proportional hazards throughout the follow-up period. This assumption will be checked by plotting by group the log of the cumulative hazard against the log of time. Under the assumption of proportionality of the hazard, both curves should be parallel. A test based on the Schoenfield residuals will be performed.

If there is strong evidence that the hazard is not constant over the surveillance period, then alternative approaches to analyze the data will be examined according to the following:

- Cox regression models with time varying covariates, using fractional polynomials to identify the function of the time that best fits. The following functions of the time will be explored: -2, -1, -½, log, identity, ½, 2, 3. The Akaike's criterion and the Schwarz Bayesian criterion will be explored.
- Piecewise Cox regression model, partitioning the surveillance period in three periods based on the equal number of events

For the assessment of the parasite density at Month 5½, a 2x2 table presenting the frequency of positive and negative will be presented. The effect of the group will be evaluated using the Fisher exact test. The geometric mean of those positive in each group will be calculated. Difference in geometric mean and 95% CI will be estimated. The effect of the group will be evaluated using the t-test. In case the assumptions of the t-test

are not satisfied (normality of residuals and equal variance) a non-parametric Wilcoxon test will be used instead.

10.8.4.3.2. Vaccine efficacy of RTS,S/AS02A against RTS,S/AS01B

Time to first infection of *P. falciparum* will be examined using Kaplan-Meier curves for both groups. The distribution will be compared with the Wilcoxon test (if efficacy varies with time) or the Log-rank test (if it does not). Vaccine Efficacy will be assessed using Cox regression models. Vaccine efficacy is defined as 1 *minus* R where R is the hazard ratio of the RTS,S/AS02A group versus the RTS,S/AS01B (with 95% CI). Crude and adjusted estimates for covariates factors (see Section 10.8.4.4) will be presented.

10.8.4.4. Adjustment for covariates

The risk for malaria infection and development of clinical disease depends on numerous factors related to the parasite, host and vector biology. Estimates of Vaccine Efficacy will be made for the following covariates:

- age: continuous variable
- village of residence
- distance from Kombewa Clinic
- Sickle-cell trait

An exploratory examination of the marginal effect each of the above covariates have on the treatment estimate may also be undertaken.

10.8.4.5. Hemoglobin values

The percentage change in hemoglobin values will be calculated as 100 x (14 weeks post Dose 3 *minus* baseline) / baseline. Descriptive statistics (mean, standard deviation, median, minimum and maximum) will be tabulated by group (RTS,S/AS02A RTS,S/AS01B and Rabies vaccine recipients). Comparisons between groups will be perfomred by Wilcoxon Rank Sum test (two sided, alpha = 0.05).

10.8.5. Analysis of parasite genotyping

The main endpoint for assessing the strain specificity of the vaccine will be the relative proportion of the vaccine type (3D7 / NF54) in the vaccine vs. control group. For each infection, the predominant allele will be counted in the analysis in two ways. Firstly, the number of polymorphic amino acid positions that differ from the vaccine sequence will be scored for each isolate, and tested. Differences among the three treatment groups will be tested for significance by the Wilcoxon Rank SumTest. Secondly, the prevalence of the vaccine-like amino acid residue at each polymorphic site will be compared between treatment groups using the Fisher's Exact test. If two alleles are equally abundant in an infection, a random number table will be used to determine which allele goes into the analysis.

Between group comparison of the multiplicity of infection of emergent infections will be performed as described in the analyses of previous vaccine trials [Alloueche 2003; Haywood 1999; Enosse, 2006]. Briefly, to determine whether the vaccine reduces the number of P. falciparum genotypes, the number of genotypes in each isolate will be taken as the number of alleles of msp1 or msp2, whichever is the greater. The differences between mean MOI in the vaccinated vs. control groups will be compared using the Wilcoxon test. Regression analysis will be used to assess the effect of the vaccine on multiplicity of infection adjusted for geometric mean parasite density and age.

Amended 13 June 2006

10.9. Planned Interim Analysis

One interim analysis is planned at one month post Dose 3, Clinic Visit 7. The interim analysis will be performed by GSK Biologicals. In order to maintain blinding, the analyses will be performed by a statistician independent of the GSK Biologicals clinical teams. The interim analysis will be done on cleaned data and will contain analyses of reactogenicity, safety and immunogenicity.

This interim analysis will contain the final analyses for the reactogenicity (local and general solicited symptoms within 7 days of vaccination and unsolicited symptoms within 14 or 30 days of vaccination) and immunogenicity on Clinic Visit 2, 3 and 7.

10.9.1. Analysis of demographics/baseline characteristics

Demographic characteristics (age, gender) of each study cohort will be tabulated.

The distribution of subjects enrolled will be tabulated as a whole and per group.

10.9.2. Analyses of safety

The analysis for safety will be conducted on the Total Cohort for Safety.

For the safety primary objective, the occurrence of Grade 3 solicited or unsolicited general reactions will be determined on the Total Vaccinated Cohort. The proportion of subjects with a Grade 3 solicited or unsolicted reaction, reported from study start until study Clinic Visit 7 will be tabulated with exact 95% CI. Comparisons between groups will be done using Fisher’s Exact Test for each preferred term.

The occurrence of SAEs will be evaluated on the Total Vaccinated Cohort. The proportion of subjects with an SAE, classified by the MedDRA preferred term level, reported from study start until study Clinic Visit 7 will be tabulated with exact 95% CI. Comparisons between groups will be done using Fisher’s Exact Test.

The occurrence of adverse events will be evaluated on the Total Vaccinated Cohort. The proportion of subjects with an AE, classified by the MedDRA preferred term level, reported from study start until Clinic Visit 7 will be tabulated with exact 95% CI.

Comparisons between groups will be done using Fisher's Exact Test. Similar tables will be generated for Grade 3 events and the relationship of the event to vaccination.

For each sampling timepoint, the biochemical parameters (ALT, bilirubin, creatinine) above normal range and hematological parameters (hemoglobin, total WBC, platelets and absolute lymphocyte count) below normal range will be described. Similar tables will be generated for Grade 3 parameters (as determined from Table 32).

10.9.3. Analyses of immunogenicity

The primary analysis will be based on the ATP cohort for analysis of immunogenicity. If the percent of enrolled subjects excluded from this ATP cohort is more than 5%, a second analysis based on the Total Vaccinated cohort will be performed to complement the ATP analysis.

10.9.3.1. Anti-CS antibodies

The percentage of subjects with sero-positive levels of anti-CS (proportion of subjects with anti-CS antibody titers greater than or equal to 0.5 EU/mL) with 95% CI will be determined prior to vaccination, at 1 month post Dose 2, 1 Month post Dose 3 and 4 months post Dose 3. Antibody titers will be summarized by GMT with 95%CI. Antibody titers after the third dose will also be investigated using reverse cumulative curves.

10.9.3.2. Anti-HBs antibodies

The percentage of subjects with sero-positive levels of anti-HBs (proportion of subjects with anti-HBs antibody titers greater than or equal to 3.3 mIU/mL), the percentage of subjects with seroprotective levels of anti-HBs (≥10 mIU/mL) with 95% CI will be determined prior to vaccination, at 1 Month post Dose 3. Antibody titers will be summarized by GMT with 95%CI. Antibody titers after the third dose will also be investigated using reverse cumulative curves.

11. ADMINISTRATIVE MATTERS

To comply with Good Clinical Practice important administrative obligations relating to investigator responsibilities, monitoring, archiving data, audits, confidentiality and publications must be fulfilled. See Appendix B for details.

12. REFERENCES

Alloueche A, Milligan P, Conway DJ et al. Protective efficacy of the RTS,S/AS02A Plasmodium falciparum malaria vaccine is not strain specific. Am J Trop Med Hyg 68 (1); 2003: 97–101.

Alonso PL, Sacarlal J, Aponte JJ, Leach A, Macete E et al. Efficacy of the RTS,S/AS02A vaccine against Plasmodium falciparum infection and disease in young African children: randomised controlled trial. Lancet. 2004;364:1411-1420.

Ajjan N, Soulebot JP, Stellmann C, Biron G, Charbonnier C, Triau R et al. Results of preventive rabies vaccination with a concentrated vaccine of the PM/WI38-1503-3M rabies strain cultured on human diploid cells. Preparation of mixed antirabies-antitetanus hyperimmune immunoglobulin by plasmapheresis of blood taken from vaccinated veterinary students. *Dev Biol Stand* 1978; 40: 89-100.

Bojang KA, Milligan PJM, Pinder M, Vigneron L, Alloueche A, Kester KE et al. Efficacy of RTS,S/AS02 Malaria Vaccine against *Plasmodium falciparum* Infection in Semi-Immune Adult Men in the Gambia: A Randomized Trial. *Lancet.* 2001;358: 1927-34.

Breman JG, Egan A, Keusch GT. Introduction and Summary. The Intolerable Burden of Malaria: A New Look at the Numbers. *Am J Trop Med Hyg*. 2001;64 (1, 2)S: iv-vii.

Breman, JG. The Ears of the Hippopotamus: Manifestations, Determinants and Estimates of the Malaria Burden. *Am J Trop Med Hyg*. 2001; 64:1-11.

Costy-Berger F. Preventive rabies vaccination using vaccine prepared from human diploid cells. *Dev Biol Stand* 1978; 40: 101-104.

Cox JH, Schneider LG. Prophylactic immunization of humans against rabies by intradermal inoculation of human diploid cell culture vaccine. *J Clin Microbiol* 1976; 3 (2): 96-101.

Doherty JF, Pinder M, Tornieporth N, Carton C, Vigneron L, Milligan P et al. A Phase I Safety and Immunogenicity Trial with the Candidate Malaria Vaccine RTS,S.SBAS02 in Semi-Immune Adults in The Gambia. *J Am Soc Trop Med Hyg*. 1999;61 (6): 865-68.

Dreesen DW, Fishbein DB, Kemp DT, Brown J. Two-year comparative trial on the immunogenicity and adverse effects of purified chick embryo cell rabies vaccine for pre-exposure immunization. *Vaccine* 1989; 7 (5): 397-400.

Enosse S, Doban˜o C, Quelhas D, et al. RTS,S/AS02A malaria vaccine does not induce selection of parasites encoding divergent CSP T-cell epitopes and reduces the genotypic multiplicity of Plasmodium falciparum infections. PLoS Clin Trials 2006; 1: e5.

Escalante AA, Grebert HM, Isea R, et al. A study of genetic diversity in the gene encoding the circumsporozoite protein (CSP) of Plasmodium falciparum from different transmission areas--XVI. Asembo Bay Cohort Project. Mol Biochem Parasitol. 2002;125(1-2):83-90.

Gallup JL, Sachs JD. The Economic Burden of Malaria. *Am J Trop Med Hyg*. 2001; 64: 85-96.

GSK data on file: Investigator Brochure GlaxoSmithKline Biologicals' Candidate Vaccine Against *Plasmodium falciparum* Malaria (RTS,S/AS02A Malaria Vaccine). March 21 2003. Data on File at GlaxoSmithKline Biologicals.

GSK data on file: Phase IIb controlled randomized double-blind study to evaluate the efficacy, safety and immunogenicity of SmithKline Beecham Biologicals' candidate RTS,S malaria vaccine in semi-immune adult males in The Gambia. Protocol 257049/005 (Malaria-005) 12 December 1997. Data on File at GlaxoSmithKline Biologicals.

GSK data on file: Open Phase I study to evaluate the safety, reactogenicity, and immunogenicity of SmithKline Beecham Biologicals' candidate RTS,S malaria vaccine in semi-immune adults in Western Kenya. Protocol 257049/008 (Malaria-008) 11 September 1998. Data on File at GlaxoSmithKline Biologicals.

GSK data on file: A Phase I Double Blind, Randomized, Controlled, Staggered Study to Evaluate the Safety, Reactogenicity and Immunogenicity of 1/5, 1/2 and Full Doses of GlaxoSmithKline Biologicals' RTS,S/AS02 Candidate Malaria Vaccine, Administered IM According to a 0, 1, 3-month Vaccination Schedule in Semi-Immune Children aged 6 to 11 Years in The Gambia, a Malaria Endemic Region. Protocol 257049/015 (Malaria-015) April 17 2002. Data on File at GlaxoSmithKline Biologicals.

GSK data on file: A Phase I Double Blind, Randomized, Controlled, Staggered Study to Evaluate the Safety, Reactogenicity and Immunogenicity of 1/5, 1/2 and Full Doses of GlaxoSmithKline Biologicals' RTS,S/AS02 Candidate Malaria Vaccine, Administered IM According to a 0, 1, 3-month Vaccination Schedule in Semi-Immune Children aged 1 to 5 Years in The Gambia, a Malaria Endemic Region. Protocol 257049/020 (Malaria-020) April 18 2002. Data on File at GlaxoSmithKline Biologicals.

GSK data on file: A Phase I Double Blind, Randomized, Controlled, Staggered Study to Evaluate the Safety, Reactogenicity and Immunogenicity of GlaxoSmithKline Biologicals' RTS,S/AS02 Candidate Malaria Vaccine, Administered IM According to a 0, 1, 2-month Vaccination Schedule in Children aged 1 through 4 years in Mozambique, a Malaria Endemic Region. Protocol 257049/025 (Malaria-025) December 14, 2001. Data on File at GlaxoSmithKline Biologicals.

GSK data on file: A double-blind randomised controlled phase IIb study to evaluate the safety, immunogenicity and efficacy of GlaxoSmithKline Biologicals' candidate malaria vaccine RTS,S/AS02A, administered IM according to a 0, 1 and 2 month vaccination schedule in toddlers and children aged 1 to 4 years in a malaria-endemic region of Mozambique. Protocol 257049/026 (Malaria-026) 17 January 2003. Data on File at GlaxoSmithKline Biologicals.

GSK data on file: Double-blind, randomized phase I/IIa human challenge study, to evaluate the safety, reactogenicity, immunogenicity, and preliminary efficacy after primary sporozoite challenge and rechallenge, of GSK Biologicals' candidate malaria vaccines containing the antigen RTS,S adjuvanted with either AS02A or AS01B and administered intramuscularly at months 0, 1, 2 in healthy malaria-naïve volunteers aged 18 - 45 years. Protocol 257049/027 (Malaria-027) 24 July 2003. Data on File at GlaxoSmithKline Biologicals.

GSK data on file: A Phase I/II randomized double-blind bridging study to evaluate the safety and immunogenicity of GlaxoSmithKline Biologicals' candidate vaccines RTS,S/AS02D (0.5 mL dose) and RTS,S/AS02A (0.25 mL dose) administered IM

according to a 0, 1, 2 month vaccination schedule in children aged 3 to 5 years living in a malaria-endemic region of Mozambique. Protocol 257049/034 (Malaria-034) 03 October 2003. Data on File at GlaxoSmithKline Biologicals.

Haywood M, Conway DJ, Weiss H, et al. Reduction in the mean number of Plasmodium falciparum genotypes in Gambian children immunized with the malaria vaccine SPf66. Transactions of the Royal Society of Tropical Medicine and Hygiene 1999; 93 (1), 65-68.

Kester KE, McKinney DA, Tornieporth N et al. Efficacy of recombinant circumsporozoite protein vaccine regimens against experimental *Plasmodium falciparum* malaria. *J Infect Dis*. 2001; 15;183(4):640-7

Kuwert EK, Marcus I, Werner J, Iwand A, Thraenhart O. Some experiences with human diploid cell strain-(HDCS) rabies vaccine in pre- and post-exposure vaccinated humans. *Dev Biol Stand* 1978; 40: 79-88.

Nicholson KG, Farrow PR, Bijok U, Barth R. Pre-exposure studies with purified chick embryo cell culture rabies vaccine and human diploid cell vaccine: serological and clinical responses in man. *Vaccine* 1987; 5 (3) :208-210.

Snounou G, Zhu X, Siripoon N, et al. Biased distribution of msp1 and msp2 allelic variants in Plasmodium falciparum populations in Thailand. Transactions of the Royal Society of Tropical Medicine and Hygiene 1999; 93 (4), 369-374

Stoute JA, Slaoui M, Heppner DG, Momin P, Kester KE, Desmons P et al. A Preliminary Evaluation of a Recombinant Circumsporozoite Protein Vaccine against *Plasmodium falciparum* Malaria. *N Eng J Med* 1997; 336: 86-91

Vodopija I, Sureau P, Lafon M, Baklaic Z, Ljubicic M, Svjetlicic M et al. An evaluation of second generation tissue culture rabies vaccines for use in man: a four-vaccine comparative immunogenicity study using a pre-exposure vaccination schedule and an abbreviated 2-1-1 postexposure schedule. *Vaccine* 1986; 4 (4): 245-248.

Vryheid RE, Kane MA, Muller N, Schatz GC, Bezabeh S. Infant and Adolescent Hepatitis B Immunization up to 1999: A Global Overview.*Vaccine* 2001;19: 1026-1037.

Wasi C, Chaiprasithikul P, Chavanich L, Puthavathana P, Thongcharoen P, Trishanananda M. Purified chick embryo cell rabies vaccine. *Lancet* 1986; 1 (8471): 40.

World Health Organization. Hepatitis B Fact Sheet WHO/204. www.who.int/inf-fs/en/fact204.html Accessed 11 April 2003.

Amended 13 June 2006

Appendix A World Medical Association Declaration of Helsinki

**Recommendations guiding physicians
in biomedical research involving human subjects**

**Adopted by the 18th World Medical Assembly
Helsinki, Finland, June 1964**

**and amended by the
29th World Medical Assembly
Tokyo, Japan, October 1975
35th World Medical Assembly
Venice, Italy, October 1983
41st World Medical Assembly
Hong Kong, September 1989
and the
48th General Assembly
Somerset West, Republic of South Africa, October 1996**

INTRODUCTION

It is the mission of the physician to safeguard the health of the people. His or her knowledge and conscience are dedicated to the fulfilment of this mission.

The Declaration of Geneva of the World Medical Association binds the physician with the words, "The health of my patient will be my first consideration," and the International Code of Medical Ethics declares that, "A physician shall act only in the patient's interest when providing medical care which might have the effect of weakening the physical and mental condition of the patient."

The purpose of biomedical research involving human subjects must be to improve diagnostic, therapeutic and prophylactic procedures and the understanding of the etiology and pathogenesis of disease.

In current medical practice most diagnostic, therapeutic or prophylactic procedures involve hazards. This applies especially to biomedical research.

Medical progress is based on research which ultimately must rest in part on experimentation involving human subjects.

In the field of biomedical research a fundamental distinction must be recognized between medical research in which the aim is essentially diagnostic or therapeutic for a patient, and medical research, the essential object of which is purely scientific and without implying direct diagnostic or therapeutic value to the person subjected to the research.

Special caution must be exercised in the conduct of research which may affect the environment, and the welfare of animals used for research must be respected.

Because it is essential that the results of laboratory experiments be applied to human beings to further scientific knowledge and to help suffering humanity, the World Medical Association has prepared the following recommendations as a guide to every physician in

biomedical research involving human subjects. They should be kept under review in the future. It must be stressed that the standards as drafted are only a guide to physicians all over the world. Physicians are not relieved from criminal, civil and ethical responsibilities under the laws of their own countries.

I. BASIC PRINCIPLES

1. Biomedical research involving human subjects must conform to generally accepted scientific principles and should be based on adequately performed laboratory and animal experimentation and on a thorough knowledge of the scientific literature.
2. The design and performance of each experimental procedure involving human subjects should be clearly formulated in an experimental protocol which should be transmitted for consideration, comment and guidance to a specially appointed committee independent of the investigator and the sponsor provided that this independent committee is in conformity with the laws and regulations of the country in which the research experiment is performed.
3. Biomedical research involving human subjects should be conducted only by scientifically qualified persons and under the supervision of a clinically competent medical person. The responsibility for the human subject must always rest with a medically qualified person and never rest on the subject of research, even though the subject has given his or her consent.
4. Biomedical research involving human subjects cannot legitimately be carried out unless the importance of the objective is in proportion to the inherent risk to the subject.
5. Every biomedical research project involving human subjects should be preceded by careful assessment of predictable risks in comparison with foreseeable benefits to the subject or to others. Concern for the interests of the subject must always prevail over the interests of science and society.
6. The right of the research subject to safeguard his or her integrity must always be respected. Every precaution should be taken to respect the privacy of the subject and to minimize the impact of the study on the subject's physical and mental integrity and on the personality of the subject.
7. Physicians should abstain from engaging in research projects involving human subjects unless they are satisfied that the hazards involved are believed to be predictable. Physicians should cease any investigation if the hazards are found to outweigh the potential benefits.
8. In publication of the results of his or her research, the physician is obliged to preserve the accuracy of the results. Reports of experimentation not in accordance with the principles laid down in this Declaration should not be accepted for publication.
9. In any research on human beings, each potential subject must be adequately informed of the aims, methods, anticipated benefits and potential hazards of the study and the discomfort it may entail. He or she should be informed that he or she is at liberty to abstain from participation in the study and that he or she is free to

withdraw his or her consent to participation at any time. The physician should then obtain the subject's freely-given informed consent, preferably in writing.

10. When obtaining informed consent for the research project the physician should be particularly cautious if the subject is in a dependent relationship to him or her or may consent under duress. In that case the informed consent should be obtained by a physician who is not engaged in the investigation and who is completely independent of this official relationship.

11. In case of legal incompetence, informed consent should be obtained from the legal guardian in accordance with national legislation. Where physical or mental incapacity makes it impossible to obtain informed consent, or when the subject is a minor, permission from the responsible relative replaces that of the subject in accordance with national legislation.
    Whenever the minor child is in fact able to give a consent, the minor's consent must be obtained in addition to the consent of the minor's legal guardian.

12. The research protocol should always contain a statement of the ethical considerations involved and should indicate that the principles enunciated in the present Declaration are complied with.

II. MEDICAL RESEARCH COMBINED WITH PROFESSIONAL CARE (Clinical research)

1. In the treatment of the sick person, the physician must be free to use a new diagnostic and therapeutic measure, if in his or her judgement it offers hope of saving life, re-establishing health or alleviating suffering.

2. The potential benefits, hazards and discomfort of a new method should be weighed against the advantages of the best current diagnostic and therapeutic methods.

3. In any medical study, every patient - including those of a control group, if any - should be assured of the best proven diagnostic and therapeutic method. This does not exclude the use of inert placebo in studies where no proven diagnostic or therapeutic method exists.

4. The refusal of the patient to participate in a study must never interfere with the physician–patient relationship.

5. If the physician considers it essential not to obtain informed consent, the specific reasons for this proposal should be stated in the experimental protocol for transmission to the independent committee (I, 2).

6. The Physician can combine medical research with professional care, the objective being the acquisition of new medical knowledge, only to the extent that medical research is justified by its potential diagnostic or therapeutic value for the patient.

III. NON-THERAPEUTIC BIOMEDICAL RESEARCH INVOLVING HUMAN SUBJECTS (Non-clinical biomedical research)

1. In the purely scientific application of medical research carried out on a human being, it is the duty of the physician to remain the protector of the life and health of that person on whom biomedical research is being carried out.

2. The subjects should be volunteers - either healthy persons or patients for whom the experimental design is not related to the patient's illness.
3. The investigator or the investigating team should discontinue the research if in his/her or their judgement it may, if continued, be harmful to the individual.

In research on man, the interest of science and society should never take precedence over considerations related to the well being of the subject.

Appendix B Administrative Matters

I. Responsibilities of the Investigator

- To ensure that he/she has sufficient time to conduct and complete the study and has adequate staff and appropriate facilities and equipment which are available for the duration of the study and to ensure that other studies do not divert essential subjects or facilities away from the study at hand.
- To submit an up-to-date curriculum vitae and other credentials (e.g., medical license number in the United States) to GSK Biologicals and—where required—to relevant authorities.
- To acquire the normal ranges for laboratory tests performed locally and, if required by local regulations, obtain the Laboratory License or Certification.
- To ensure that no clinical samples (including serum samples) are retained on site or elsewhere without the approval of GSK Biologicals and the express written informed consent of the subject and/or the subject's legally authorized representative.
- To perform no other biological assays at the investigator site except those described in the protocol or its amendment(s).
- To prepare and maintain adequate case histories designed to record observations and other data pertinent to the study.
- To conduct the study in compliance with the protocol and appendices.
- To co-operate with a representative of GSK Biologicals in the monitoring process of the study and in resolution of queries about the data.

II. Protocol Amendments and Administrative changes

- No changes to the study protocol will be allowed unless discussed in detail with the GSK Biologicals' Clinical Development Manager/Medical Monitor and filed as an amendment/administrative change to this protocol.
- Any amendment/administrative change to the protocol will be adhered to by the participating centre(s) and will apply to all subjects. Written IRB/IEC approval of protocol amendments is required prior to implementation.

III. Sponsor's Termination of Study

The Sponsor, USAMMDA, reserves the right to temporarily suspend or prematurely discontinue this study either at a single site or at all sites at any time for reasons including, but not limited to, safety or ethical issues or severe non-compliance. Reasons for suspension or early termination will be documented in the study file at USAMMDA and GSK Biologicals.

If USAMMDA determines that suspension or early termination is needed, USAMMDA will discuss this with the Investigator (including the reasons for taking such action) and GSK Biologicals. When feasible, USAMMDAwill provide advance notification to the Investigator and GSK Biologicals of the impending action prior to it taking effect.

USAMMDA will promptly inform, via written communication, all investigators and/or institutions conducting the study, if the study is suspended or terminated for safety reasons, and will also inform the regulatory authorities of the suspension or termination of the study and the reason(s) for the action. If required by applicable regulations, the investigator must inform the IEC/IRB promptly and provide the reason for the suspension or termination.

If the study is prematurely discontinued, all study data must be returned to GSK Biologicals. In addition, arrangements will be made for all unused investigational product(s) in accordance with the applicable GSK procedures for the study. Financial compensation to investigators and/or institutions will be in accordance with the agreement established between the investigator and/or institutions and GSK.

IV. Case Report Form Instructions

Prior to screening the first potential participant, the investigator will provide the Site Monitor with a list (Site Staff Signature Sheet) showing the signature and hand-written initials of all individuals authorized to make or change entries on CRFs (already defined). If the authorized individuals should change during the study, the investigator is to inform GSK Biologicals of the specific change(s).

CRFs (and subject diary cards, if applicable), will be supplied by GSK Biologicals for recording all data. It is the responsibility of the investigator or co-investigator to ensure that CRFs (and subject diary cards) are legible and completely filled in with a black ink fountain or ballpoint pen.

Errors must be corrected by drawing a single line through the incorrect entry and writing in the new value/data positioned as close to the original as possible. The correction must then be initialled, dated and justified, where necessary, by the authorized individual making the change. The original entry must not be obliterated, overwritten or erased when a correction is made.

When a subject completes a visit, it is anticipated that relevant sections of the CRF will be completed by the investigator (or designated staff as documented in the Site Staff Signature Sheet) as soon as possible after the last data becoming available. Similarly, when a subject completes a study, it is anticipated that all relevant CRF pages will be completed promptly after the last data becoming available. This also applies to forms for potential study participants who were screened but not randomized to a study group.

As soon as the subject has completed/withdrawn from the study and the CRF is completed, the principal investigator or designated physician(s) under his/her supervision will sign the study conclusion pages of the CRF to confirm that they have reviewed the data and that the data are complete and accurate. In all cases the investigator remains accountable for the study data collected.

An original (top copy) CRF or log sheets must be submitted for all subjects who have undergone protocol specific procedures, whether or not the subject completed the study.

While completed CRFs are reviewed by a GSK Biologicals professional monitor at the study site, errors detected by subsequent in-house CRF review may necessitate clarification or correction of errors with documentation and approval by the investigator or appropriately qualified staff as documented on the Site Staff Signature Sheet. In all cases, the investigator remains accountable for the study data. Wherever possible the investigator should assist in the clarification or correction of errors detected after study finalization promptly after being brought to the attention of the investigator (preferably within 48 hours).

Any questions or comments related to the CRF should be directed to the assigned Site Monitor.

V. Monitoring by GSK Biologicals

Monitoring visits by a professional representative of GSK Biologicals will be scheduled to take place as close as possible to entry of the first subject, during the study at appropriate intervals and after the last subject has completed the study. It is anticipated that monitoring visits will occur at a frequency defined before study start.

These visits are for the purpose of confirming that GSK Biologicals' studies are being conducted in compliance with the relevant Good Clinical Practice regulations/ guidelines, verifying adherence to the protocol and the completeness and accuracy of data entered on the CRF pages/RDE screens and Vaccine Inventory Forms. The monitor will verify CRF/RDE entries by comparing them with the source data/documents that will be made available by the investigator for this purpose. Data to be recorded directly into the CRF pages/RDE screens will be specified in writing preferably in the source documentation agreement form that is contained in both the monitor's and investigator's study file. For RDE, the monitor will mark completed and approved screens at each visit. The investigator must ensure provision of reasonable time, space and adequate qualified personnel for monitoring visits.

VI. Archiving of Data

Following closure of the study, the investigator must maintain all site study records in a safe and secure location. The records must be maintained to allow easy and timely retrieval, when needed (e.g., audit or inspection), and, whenever feasible, to allow any subsequent review of data in conjunction with assessment of the facility, supporting systems, and staff. Where permitted by applicable laws/regulations or institutional policy, some or all of these records can be maintained in a validated format other than hard copy (e.g., microfiche, scanned, electronic for studies with an eCRF, for example); however, caution needs to be exercised before such action is taken. The investigator must assure that all reproductions are legible and are a true and accurate copy of the original, and meet accessibility and retrieval standards, including re-generating a hard copy, if required. Furthermore, the investigator must ensure there is an acceptable back-up of these reproductions and that an acceptable quality control process exists for making these reproductions.

GSK will inform the investigator/ institution of the time period for retaining these records to comply with all applicable regulatory requirements. However, the investigator/

institution should seek the written approval of the sponsor, USAMMDA, GSK Biologicals and WRAIR before proceeding with the disposal of these records. The minimum retention time will meet the strictest standard applicable to that site for the study, as dictated by ICH GCP E6 Section 4.9, any institutional requirements or applicable laws or regulations, or GSK standards/procedures; otherwise, the minimum retention period will default to 15 years.

The investigator/ institution must notify GSK of any changes in the archival arrangements, including, but not limited to, the following: archival at an off-site facility, transfer of ownership of the records in the event the investigator leaves the site.

VII. Audits

For the purpose of compliance with Good Clinical Practice and Regulatory Agency Guidelines it may be necessary for GSK Biologicals or a Drug Regulatory Agency to conduct a site audit. This may occur at any time from start to after conclusion of the study.

When an investigator signs the protocol, he agrees to permit drug regulatory agencies and GSK Biologicals audits, providing direct access to source data/ documents. Furthermore, if an investigator refuses an inspection, his data will not be accepted in support of a New Drug Registration and/or Application, Biologics Licensing Application.

GSK Biologicals has a substantial investment in clinical studies. Having the highest quality data and studies are essential aspects of vaccine development. GSK Biologicals has a Regulatory Compliance staff who audit investigational sites. Regulatory Compliance assesses the quality of data with regard to accuracy, adequacy and consistency. In addition, Regulatory Compliance assures that GSK Biologicals studies are in accordance with GCP and that relevant regulations/guidelines are being followed.

To accomplish these functions, Regulatory Compliance selects investigational sites to audit. These audits usually take 1 to 2 days. The GSK Biologicals' audits entail review of source documents supporting the adequacy and accuracy of CRFs, review of documentation required to be maintained, and checks on vaccine accountability. The GSK Biologicals' audit therefore helps prepare an investigator for a possible regulatory agency inspection as well as assuring GSK Biologicals of the validity of the database across investigational sites.

The Inspector will be especially interested in the following items:

- Log of visits from the representatives of the sponsor, USAMMDA, GSK Biological and WRAIR
- IRB/IEC approval
- Vaccine accountability
- Approved study protocol and amendments
- Informed consent of the subjects (written consent [or witnessed oral if applicable])

- Medical records and other source documents supportive of CRF data
- Reports to the IRB/IEC the sponsor, USAMMDA , GSK Biologicals and WRAIR
- Record retention.

GSK Biologicals will gladly help investigators prepare for an inspection.

VIII. Ownership, Confidentiality and Publication

Ownership:

This study is vovered by a Cooperative Research and Development Agreement between GSK Biologicals and the Walter Reed Army Institute of Research made effective as of 01 March 2003.

Confidentiality:

All information provided by GSK and all data and information generated by the site as part of the study (other than a subject's medical records) will be kept confidential by the investigator and other site staff. This information and data will not be used by the investigator or other site personnel for any purpose other than conducting the study. These restrictions do not apply to: (1) information which becomes publicly available through no fault of the investigator or site staff; (2) information which it is necessary to disclose in confidence to an IEC or IRB solely for the evaluation of the study; (3) information which it is necessary to disclose in order to provide appropriate medical care to a study subject; or (4) study results which may be published as described in the next paragraph. If a written contract for the conduct of the study which includes confidentiality provisions inconsistent with this statement is executed, that contract's confidentiality provisions shall apply rather than this statement.

Publication:

For multicentre studies, the first publication or disclosure of study results shall be a complete, joint multicentre publication or disclosure coordinated by GSK. Thereafter, any secondary publications will reference the original publication(s).

Prior to submitting for publication, presentation, use for instructional purposes, or otherwise disclosing the study results generated by the site (collectively, a "Publication"), the investigator shall provide GSK with a copy of the proposed Publication and allow GSK a period of at least thirty (30) days [or, for abstracts, at least five (5) working days] to review the proposed Publication. Proposed Publications shall not include either GSK confidential information other than the study results or personal data on any subject, such as name or initials.

At GSK's request, the submission or other disclosure of a proposed Publication will be delayed a sufficient time to allow GSK to seek patent or similar protection of any inventions, know-how or other intellectual or industrial property rights disclosed in the proposed Publication.

If a written contract for the conduct of the study, which includes publication provisions inconsistent with this statement is executed, that contract's publication provisions shall apply rather than this statement.

Appendix C Overview of the Recruitment Plan

Prior to enrollment, a specific information campaign towards the targeted population will be implemented. This will start with the administrative and senior leaders of the villages in the recruitment area around KC and expand to the barazas (town meetings) that everyone in the village can attend.

The sessions will explain the problem of malaria in this community, the current strategies for its control, as well as the limitations of these strategies. The need and the difficulties of developing a vaccine against malaria will be discussed, as well as an outline of the proposed trial, including the rationale, the background data available and the study objectives. Particular attention will be paid to study procedures including screening of subjects for hepatitis B, immunization and blood collection. In that respect a full discussion on the purpose of blood collection and the associated risks will be carried out.

Next, Field Workers will be available in villages in the study area to answer any additional questions on a one-on-one basis, schedule volunteers for a briefing and coordinate transportation to the briefing. On the day of the scheduled briefing Field Workers with transportation will escort them to KC.

At KC, volunteers will view a video in their native language that explains the nature and purpose of the study and reviews the entire informed consent document. There will be a period for asking questions afterwards. Volunteers will then receive both oral and written explanations of the study one-on-one with a study coordinator or clinician and again allowed to ask questions. Afterwards, written informed consent will be obtained from each person who wants to participate in the study. In addition, all volunteers who sign an informed consent form will be required to fill in a form that will be entered into the US Army Medical Research and Materiel Command Volunteer Registry Data Base. This form requires details of the volunteer’s sex, date of birth, permanent home address, permanent home phone number, as well as their local address and phone number if it is different from their permanent details. In the instructions to the volunteer, this form states that the disclosure of each volunteer’s Social Security Number (SSN) is mandatory; however, as SSNs are not issued in Kenya, no SSN will be recorded on this form. The intent of the data base is to readily answer questions concerning an individual’s participation in research sponsored by USAMRMC and to ensure that the USAMRC can exercise its obligation to ensure research volunteers are adequately warned of risks and to provide them with new information as it becomes available. The information will be stored at USAMRMC for a minimum of 75 years. So that language and illiteracy will not be impediments to informed consent, all briefings and explanations will be in Dholuo, the local language.

Screening may be done on a separate visit from recruiting/consenting. Only adults aged 18 to 35 years that have previously given consent will be eligible to be screened for the trial.

Volunteers will provide a medical history through a one-on-one interview with a clinical officer and undergo physical examination and standard laboratory screening tests, which include complete blood count (CBC), creatinine, and ALT. Blood to screen for hemoglobinopathies (sickle cell disease/trait, G6PD deficiency, and alpha-thalassemia)

will also be drawn. Subjects may be screened one additional time if re-screening is determined to be necessary (e.g., after treatment for malaria). Field Workers will notify each volunteer of their eligibility to enroll in the study. Subjects will be excluded from participation if they meet any of the exclusion criteria. Subjects excluded from this study because of significant abnormalities will have follow up evaluation and treatment coordinated for them by the KC personnel (refer to Section 5.7.3 of the Study Protocol).

Appendix D Handling of Biological Samples Collected by the Investigator

Instructions for Handling of Serum Samples

When materials are provided by GSK Biologicals, it is **MANDATORY** that all clinical samples (including serum samples) will be collected and stored using exclusively those materials in the appropriate manner. The use of other materials could result in the exclusion of the subject from the ATP analysis. The investigator must ensure that his/her personnel and the laboratory(ies) under his/her supervision comply with this requirement. However, when GSK Biologicals does not provide material for collecting and storing clinical samples, then appropriate materials from the investigator's site are to be used.

1. Collection

The whole blood (by capillary or venous route) should be collected observing appropriate aseptic conditions. It is recommended that Vacutainer® tubes WITH integrated serum separator (e.g. Becton-Dickinson Vacutainer® SST or Corvac® Sherwood Medical) be used to minimize the risk of haemolysis and to avoid blood cell contamination of the serum when transferring to standard serum tubes.

2. Serum separation

These guidelines aim to ensure high quality serum by minimizing the risk of haemolysis, blood cell contamination of the serum or serum adverse cell toxicity at testing.

- For separation of serum using Vacutainer® tubes, the instructions provided by the manufacturer should be followed. Siliconized tubes should never be used (cell toxicity). Often the manufacturer's instruction states that the relative centrifugal acceleration known also as "G" must be "between 1000 and 1300 G" with tubes spinning for ten minutes. Error in calculation of centrifuge speed can occur when laboratory personnel confuse "G" acceleration with "RPM" (revolutions per minute). The speed of centrifugation must be calculated using the "G" rate provided in the manufacturer's instructions and the radius of the centrifuge head. After measuring the radius of the centrifuge machine, a speed/acceleration nomograph must be employed to determine the centrifuge speed in "RPM".
- Following separation, the serum should be aseptically transferred to the appropriate standard tubes using a sterile disposable pipette. The serum should be transferred as gently as possible to avoid blood cell contamination.
- The tube should not be overfilled (max. 3/4 of the total volume) to allow room for expansion upon freezing.
- The tube should be identified by the appropriate label provided by GSK Biologicals (see point 3).

3. Labelling

- The standard labels provided by GSK Biologicals should be used to label each serum sample.
- If necessary, any hand-written additions to the labels should be made using indelible ink.

- The label should be attached to the tube as follows (see diagram):
  - first attach the paper part of the label to the tube
  - then wrap the label around the tube so that the transparent, plastic part of the label overlaps with the label text and bar code and shields them.

This will ensure optimal label attachment.

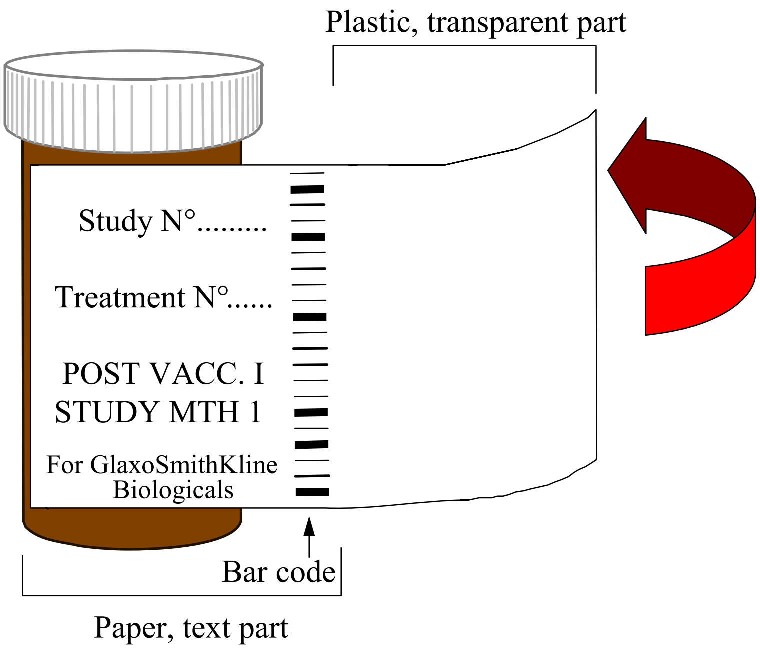

- Labels should not be attached to caps.

4. Sorting and storage

- Tubes should be placed in the GSK Biologicals' cardboard boxes in numerical order from left to right, starting from the lower left hand corner, beginning with the pre-vaccination samples series, then with the post-vaccination sample series.
- The tubes of serum should be stored in a vertical position at approximately -20°C (alternatively at approximately -70°/80°C is also acceptable) until shipment to GSK Biologicals. The storage temperature should be checked regularly and documented. Wherever possible, a backup facility for storage of serum samples should be available.
- A standard Serum Listing Form, specifying the samples being shipped for individual subjects at each timepoint, should be prepared for each shipment. A copy of this list should be retained at the study site, while the original should be sealed in a plastic envelope and shipped with the serum samples.

- Once flight details are known, a standard Specimen Transfer Form must be completed and faxed to GSK Biologicals to the number provided below. A copy of the Specimen Transfer Form must be in the parcel.[1]

GLAXOSMITHKLINE BIOLOGICALS

Attention Biospecimen Reception
Clinical Immunology
R & D Department/Building 44
Rue de l'Institut, 89
B-1330 Rixensart – Belgium

Telephone:	+32-2-656 8949 or +32-2-656 6130<br>or +32-2-656 8549 or +32-2-656 6108
Fax:	+32-2-656 6052
E-mail:	rix.ugbiospecimen-reception@gskbio.com

Instructions for Handling Cells for Cell-Mediated Immunity Assay

When materials are provided by GSK Biologicals, it is mandatory that all clinical samples be collected and stored using exclusively those materials in the appropriate manner. The use of other materials could result in the exclusion of the subject from analysis. The investigator must ensure that his/her personnel and the laboratory(ies) under his/her supervision comply with this requirement.

1. Collection of whole blood

Collect blood by venipuncture in Terumo tubes with heparin (or equivalent) and record time of collection. The tubes should be kept at room temperature and shipped to a designated clinical site for separation of peripheral blood mononuclear cells (PBMCs). The shipment must be timed to ensure that PBMC separation will be performed within 24 hours. Use well closed styrofoam boxes of 5 cm thickness for blood samples transport (see current version of GSK Biologicals SOP RD_HCI_001 for guidance).

2. Separation and freezing of PBMCs

PBMCs will be separated on a density gradient, aliquoted and frozen at –80°C for 24 hours and further cryopreserved in liquid nitrogen until testing (see GSK Biologicals SOP: RD_HCI_007 for guidance).

3. Labelling of cryotubes for PBMC samples

- If labels are provided by GSK, it is mandatory to use them.

[1] The Serum Listing Form and the Specimen Transfer Form are standard documents used in GSK Biologicals' clinical trials. These documents are provided by GSK Biologicals' Clinical Trials' monitor at study initiation.

- If necessary, any hand-written additions to the labels should be made using indelible ink.

4. Sorting and storage of PBMC samples

Samples should be stored in liquid nitrogen until shipment to GSK Biologicals, if needed. Wherever possible, a backup facility for storage of samples should be available.

A standard Cryotube Listing Form (see current version of GSK Biologicals SOP RD_HCI_009 for guidance), specifying the samples being shipped for individual subjects at each timepoint, should be prepared for each shipment. A copy of this list should be retained at the study site, while the original should be sealed in a plastic envelope and shipped with the PBMC samples.

Appendix E Shipment of Biological Samples

Instructions for Shipment of Serum Samples

Serum samples should be sent to GSK Biologicals at regular intervals. The frequency of shipment of samples should be decided upon by the Site Monitor, Central Study Coordinator and the investigator prior to the study start.

Serum samples should always be sent by air, preferably on a Monday, Tuesday or Wednesday, unless otherwise requested by the GSK Biologicals representative.

Serum samples must be placed with dry ice (maximum -20°C) in a container complying with International Air Transport Association (IATA) requirements. The completed standard serum listing form should always accompany the shipment.

The container must be clearly identified with the labels provided by GSK Biologicals specifying the shipment address and the storage temperature (-20°C).

The airway bill should contain the instruction for storage of samples at maximum -20°C.

A “proforma” invoice, stating a value for customs purposes only, should be prepared and attached to the container. This document should contain the instruction for storage of samples at maximum -20°C.

Details of the shipment, including:

* number of samples
* airway bill
* flight number
* flight departure and arrival times

should be sent by fax or by e-mail, two days before shipment, to:

GLAXOSMITHKLINE BIOLOGICALS

Attention Biospecimen Reception
Clinical Immunology
R & D Department/Building 44
Rue de l’Institut, 89
B-1330 Rixensart – Belgium

Telephone: +32-2-656 8949 or +32-2-656 6130
or +32-2-656 8549 or +32-2-656 6108
Fax: +32-2-656 6052
E-mail: rix.ugbiospecimen-reception@gskbio.com

Appendix F Laboratory Assays

Serology testing

Serological responses will be measured principally by evaluating antibody responses to HBs and to CSP repeats (anti R32LR). Other serological testing may be done that are not currently planned. This is included in the informed consent. If a study not specifically detailed in this protocol is proposed it must first be evaluated and approved by the Kenyan Ethical Review Committee. Serum for antibody determination will be collected at the time points defined in the flowchart in Sections 5.4.

Antibody levels against CS will be measured at GSK Biologicals (or a designated laboratory) by standard ELISA methodology using plate adsorbed R32LR antigen with a standard reference antibody as a control according to SOPs from the laboratory. Results will be reported in EU/mL.

Antibody to hepatitis B surface antigen will be measured at GSK Biologicals using a commercially available ELISA immunoassay (AUSAB EIA test kit from Abbott) or equivalent according to the assay instructions. Results will be reported in mIU/mL.

Determination of parasitemia

Estimates of asexual *P. falciparum* parasite density will be made in the microscopy lab at Kombewa Cinic according to laboratory SOPs. Two slides will be air dried, stained with Giemsa and read on a light microscope with a 100x oil immersion lens and 10x eyepieces. Parasite density will be assessed by counting the number of asexual stage parasites per 200 leukocytes. Slides will be declared negative only after 100 high power fields have been read. Parasite numbers will be converted to a parasite density (count/µL) based on the WBC from a recent CBC. All slides will be read twice independently. If there is a discrepancy between the two slide readings it will be read a third time by one of the senior (R3) readers. A discrepancy occurs if the first two readers disagree on presence of malaria or malaria species, or there is a 1.5 fold difference or greater in calculated parasite densities. If there are no discrepancies, as defined above, between the first two readers, the definitive result will be the average of the two densities. If there is a discrepancy, the final result will be the results from the senior (R3) reader. Differences in parasite density are not calculated if both readers have densities less than 10. The final result is the average of the two densities.

Cell-mediated immunity

Cell-mediated immunity Methodology

T-cell mediated immunity (CMI) will be investigated in this study. CMI will be assessed at ~~***three***~~ ***four*** time points during the study, corresponding to prevaccination, 1 month post Dose 3, ~~***and***~~ 4 months post Dose 3 ***and 9 months post Dose 3***. A sample volume of 9 mL will be taken at each of these timepoints. **Amended 13 June 2006**

Three CMI laboratories will be involved in the assessment of the CMI response:

Role and Methodology; CMI Laboratory Kisumu Kenya

Whole blood (9 mL) will be incubated at 37°C for two hours with the relevant antigens. Then, an inhibitor of secretion (brefeldin A) will be added for an additional 4 hours incubation. This step allows the cytokine/activation markers to accumulate within the stimulated cells. After washing and fixing steps, an aliquot of the cells (6 mL) will be frozen at minus 70 to minus 80°C and transferred to Rixensart for analysis. The remaining 3 mL of cells will be analyzed at Kisumu.

Flow-cytometry characterization will be used on cells. For this analysis, the cell samples will be processed with classical intracellular cytokine stainings and analyses designed to identify the frequency of antigen specific CD4/CD8 T-Cells. T-cell immunity will be explored in a short term antigen-stimulation (i.e., PfCS protein peptides) of whole blood by intracellular cytokine staining and determination of cell surface activation markers using flow cytometry (Beckton Dickenson Fast Immune™ kits will be used; these contain all the necessary reagents including the fixation and freezing medium).

Role and Methodology; CMI Laboratory GSK Biologicals, Rixensart

Flow-cytometry characterization will be used on cells transferred from Kisumu, Kenya. For this analysis, the cell samples will be thawed before being processed with classical intracellular cytokine staining and analyses designed to identify the frequency of antigen specific CD4/CD8 T-Cells.

Role and Methodology; CMI Laboratory WRAIR, Washington

The senior staff at this laboratory will support the CMI Laboratory in Kisumu, Kenya, but no samples would be transferred to this laboratory.

Biochemical and hematological analyses

Hematological and biochemical testing will be done at KEMRI in Kenya, following laboratory SOPs.

Parasite Genotyping

All laboratory work will be performed by individuals blinded to vaccine assignment. Vaccine code will be assigned after completion of analyses. DNA extraction, PCR and determination of P. falciparum genotypes (MOI) will be performed at the Walter Reed laboratory, Kisumu, Kenya. The determination of CSP polymorphic sequences will be performed in the Dept of Infectious & Tropical Diseases, LSHTM, UK.

DNA extraction

EDTA blood will be collected during ADI from individuals with microscopically confirmed asexual parasitemia. DNA will be extracted from 200 μL of the EDTA blood using QIAmp DNA blood mini kit as described by the manufacturer (QIAGEN Inc., CA). DNA samples will be stored at -80°C until use.

Polymerase chain reaction

Primers that bind to conserved sequences flanking the Th2R and Th3R region of P. falciparum CSP gene will be used in a nested PCR to amplify a 381 base pair fragment covering nucleotide 936 to 1317 of the CSP gene. For the first round PCR, the following primers will be used:

First round PCR:

Forward primer (CSPf1) - 5'-caattcatgatgagaaaattagctatt-3'

Reverse primer (CSPr1) - 5'-actcaaactaagatgtgttc -3'.

Second round PCR:

Forward primer (CSPf2) - 5'- cctaataaaaacaatcaagg-3'

Reverse primer (CSPr2) -5'- tctaattaaggaacaagaaggat -3'

The composition of 1st PCR reaction mixture is shown below:

Reagent	*Final concentration*	*Unit reaction (μL)*	*X ---100--samples*
10x PCR buffer	*1x*	*2 μL*	*200*
$MgCl_2$ (25 mM)	*2.5 mM*	*2 μL*	*200*
dNTPs (10 mM)	*250 nM*	*0.5 μL*	*50*
Forward primer (CSPf1) 20 μM	*0.3 μM*	*0.3 μL*	*30*
Reverse primer (CSPr1) 20 μM	*0.3 μM*	*0.3 μL*	*30*
AmpliTaq gold polymerase (5 U/ml)	*1 U*	*0.2 μL*	*20*
DNA		*5 μL*	*-*
PCR H2O	*q.s to 20 μL*	*9.7 μL*	*970*
Total		*20 μL*	*2000*

The 2nd PCR reaction mixture will contain 1 μL of primary amplicons in a total volume of 20 μL as shown below:

Reagent	*Final concentration*	*Unit reaction (μL)*	*X ---100 samples*
10x PCR buffer	*1x*	*2 μL*	*200*
$MgCl_2$ (25 mM)	*2.5 mM*	*2 μL*	*200*
dNTPs (10 mM)	*250 nM*	*0.5 μL*	*50*
Forward primer (CSPf1) 20 μM	*0.3 μM*	*0.3 μL*	*30*
Reverse primer (CSPr1) 20 μM	*0.3 μM*	*0.3 μL*	*30*
Taq gold polymerase (5 U/ml)	*1 UI*	*0.2 μL*	*20*
DNA		*1 μL*	*-*
PCR H2O	*q.s to 20 μL*	*13.7 μL*	*1370*
Total		*20 μL*	*2000*

PCR amplifications will be performed in a DNA Tetrad Engine (MJ research) 96-well thermocycler in the following cycling conditions: denaturation during 3 min at 95ºC, followed by 40 cycles of 94ºC (1 min), 60ºC (1 min) and 72ºC (1 min) and final extension at 72ºC for 10 min. 5 μL 2nd round PCR products are monitored on a 2.0% agarose gel in 1xTAE buffer to check the quality, size and yield of the PCR products before proceeding to product purification and sequencing. The following samples will

also be included in every run: Positive control = P. falciparum culture DNA; Negative control 1 = uninfected host genomic DNA; Negative control 2 = no temperate control.

QA/QC:

For QA/QC reasons, if the positive control sample doesn't amplify or the negative control samples amplify, the whole run will be repeated.

Determination of CSP polymorphic sequences

The distribution of polymorphic variants within the Th2R and Th3R regions of P falciparum CSP will be determined using high-throughput direct sequencing of the PCR products. The sequencing strategy will be validated in a pilot study of 50 PCR samples randomly selected from pre-intervention blood samples from study participants. This will also provide important information regarding the pattern of CSP polymorphism in the study area, and an estimate of the expected data loss due to ambiguous sequence reads from mixed alleles. The sequencing using CSPf2 and CSPr2 primers will be performed in an ABI 3730 capillary sequencer (provided by the GMP). The sequence of each PCR product will be manually checked by at least two investigators and a consensus reached or the assay repeated. Data will be collated from all isolates with a single CSP allele, or where a clear majority allele can be unambiguously identified. For those isolates in which a clear majority allele is not determined from the sequence electropherogram, the majority allele will be determined by cloning of PCR products and sequencing.

Genotyping of msp-1, msp-2 and detection of multiplicity of infection (MOI)

Allele specific, nested PCR of 3 polymorphic regions of P. falciparum genes, namely msp1 block 2 and msp2 will be used to detect the genetic structure of the parasite populations, essentially as described by Snounou, 1999. Following electrophoresis, staining with ethidium bromide and observation under ultra-violet illumination, bands corresponding to different parasite allelic forms will be distinguished and counted and the number of genotypes for msp1 and msp2 loci determined. Gels will be double-scored independently by 2 investigators, and either consensus reached or the assay repeated. To gain a preliminary estimate of MOI in the study population, this genotyping will be performed on the 50 samples used in the sequencing pilot.

Amended 13 June 2006

Appendix G Vaccine supplies, packaging and accountability

1. Vaccine and/or other supplies

GSK Biologicals will supply the following amounts of numbered doses of study vaccines, sufficient to administer 3 doses to all subjects as described in the present protocol.

- Sufficient doses for 85 recipients of the candidate vaccine RTS,S/AS02A (doses of RTS,S vaccine in monodose vials and doses of AS02A adjuvant in pre-filled syringes).
- Sufficient doses for 85 recipients of the candidate vaccine RTS,S/AS01B (doses of RTS,S vaccine in monodose vials and doses of AS01B adjuvant in pre-filled syringes).
- Sufficient doses for 85 recipients of the control control rabies vaccine Rabipur (doses of Rabipur vaccine in monodose vials and doses of diluent in containers)

An additional 3% of their respective amounts of RTS,S/AS02A, RTS,S/AS01B and Rabipur will be supplied for replacement in case of breakage, bad storage conditions or any other reason that would make the vaccine unusable (i.e. given by mistake to another subject).

All monodose vials/pre-filled syringes/containers must be accounted for on the form provided.

Vaccine vials will be labeled with the following information

104743/044 (Malaria-044)
Vaccine name
Lot number: Dose number
Expiry date: Treatment Number
non dominant deltoid IM adm
Store at 2-8 deg.C (36-46 deg.F). Do not freeze.
Caution: new drug. Limited by USA law to investigational use
MFD: GSK BIOLOGICALS or CHIRON
Sponsor: USAMMDA
Internal GSK number
Pack Number

Labels for sample identification:

The investigator will receive labels from GSK Biologicals to identify samples taken from each subject at each timepoint. Each label will contain the following information: study number, identification number for the subject (e.g. Treatment Number), sampling timepoint (e.g., post vacc 3), timing (e.g. study Month 7).

Other supplies provided by GSK Biologicals:
In addition to the vaccines, the study documentation and the sample labels, the investigator will receive the following supplies:

- tubes with screw caps for serum samples,
- racks and cardboard boxes for the tubes of serum.

The investigator or pharmacist must sign a statement that he/she has received the clinical supplies for the study.

It is NOT permitted to use any of the supplies provided by GSK Biologicals for purposes other than those specified in the protocol.

2. Vaccine packaging

The vaccines will be packed in labeled boxes. The box label will contain, as a minimum, the following information: study number, treatment number, lot number (or numbers, when double-blind), instructions for vaccine administration and any other relevant regulatory requirements.

3. Vaccine shipment from GSK Biologicals Rixensart to local country medical department, dispatching centre or investigational site

On arrival of vaccine shipment, the freeze watch should be removed from the vaccine boxes and checked after 10 minutes at room temperature. The temperature recording chart (chart from Cox recorder or print-out data of the electronic device) should be obtained from the temperature recording device.

The following documents should be completed and returned to GSK Biologicals on reception of vaccine shipment:

Investigational supply receipt
Notification of vaccine delivery/temperature control
Temperature recording (chart).

These documents should then be returned to:

Attention of Stephanie Nitelet
Clinical Operations Logistics
GSK Biologicals Rixensart
Fax : +32 (0)2 656 75 17
E-mail: stephanie.nitelet@gskbio.com.

In case of any temperature deviation, the official approval for the use of vaccine must be obtained from GSK Biologicals.

4. Vaccine accountability

At all times the figures on supplied, used and remaining vaccine doses should match. At the end of the study, it must be possible to reconcile delivery records with those of used and unused stocks. An explanation must be given of any discrepancies.

After approval from GSK Biologicals and in accordance with GSK SOP NPD-112, used and unused vaccine vials/syringes/containers should be destroyed at the study site using locally approved biosafety procedures and documentation unless otherwise described in

the protocol. If no adequate biosafety procedures are available at the study site, the used and unused vaccine vials/syringes/containers are to be returned to an appropriate GSK Biologicals site for destruction, also in accordance with current GSK SOP NPD-112.

5. Transfers of clinical vaccines or products from country medical department or dispatch centre to study sites or between sites

Storage temperatures must be maintained during transport and deviations must be reported to Clinical Operations Logistics for guidance. All transfers of clinical vaccines or products must be documented using the Clinical Supply Transfer Form.

All packaging and shipment procedures for transfer of clinical vaccines or products must follow procedures approved by GSK Biologicals.

Clinical vaccines or products should always be sent by contract courier designated by GSK Biologicals, unless otherwise requested by GSK Biologicals.

Alternative local validated procedures may be followed after the documentation for these procedures has been sent to Clinical Operations Logistics and approval has then been obtained from the qualified person (or designee) in GSK Biologicals, Rixensart, before any shipment of vaccines.

Appendix H Description of drugs to be used in the trial for clearance of malaria parasites

Malarone

Malarone® is a fixed-dose combination of the antimalarial agents atovaquone and proguanil hydrochloride. Each tablet contains 250 mg of atovaquone and 100 mg proguanil hydrochloride. For treatment of malaria in adults the dosage is four Malarone® tablets in a single daily dose for three consecutive days. Malarone® tablets come in boxes containing one complete treatment dose (12 tablets in a blister pack per box). The box is labeled with the name of the medication as well as the blister pack inside. Malarone® is manufactured by GlaxoSmithKline.

Coartem

Coartem® is a fixed-dose combination of artemether and lumefantrine. Each tablet contains 20 mg artemether and 120 mg lumefantrine. For treatment of malaria in adults in countries where multi-drug resistant P. falciparum is present, the dosage is four Coartem® tablets twice daily for three consecutive days. Coartem® is manufactured by Novartis.

Appendix I Roles and responsibilities of study personnel

Investigator	Title	Responsibility
Mark E Polhemus, MD	Principal Investigator/ Chief of Clinical Trials, USAMRU-K	To conduct the study in compliance with the protocol and appendices. Conduct of the study includes protocol development, logistics, recruiting and screening, supervision of immunization, adverse event reporting, safety report development, final study report development and publication of results.
Mark R Withers, MD, MPH	Associate Investigator/ Kisumu Director, USAMRU-K	To assist the principal investigator in the conduct of the study including protocol review, assisting in recruiting and screening, assisting with pre-vaccination evaluation, assisting with post-vaccination follow ups, review of AEs and safety reports, and review of publications
James F Cummings, MD	Associate Investigator	
D Gray Heppner, MD	Associate Investigator	
Kent Kester, MD	Associate Investigator	
Steve Ntoburi MD	Associate Investigator	
Christian Ockenhouse, MD, PhD	Associate Investigator	
Bernhards Ogutu, MBChB, Mmed (Peds) , PhD	Associate Investigator	
Lucas Otieno, MBChB	Associate Investigator	
Shon A Remich, MD	Associate Investigator	
John Waitumbi DVM, PhD	Associate Investigator	
Amos E O Otedo, MD	Local Safety Monitor	Evaluation of SAEs<br>Evaluation of unanticipated AEs<br>Evaluation of safety reports prior to progression to next vaccination<br>Unblinding a subject
Urszula Krzych	Research Associate	Scientific review of the protocol and coordination and supervision of the research assays required to conduct the study. The research assays include screening for hemoglobinopathies, antibody response to hepatitis B surface antigen, antibody response to circumsporozoite antigen, PCR for circumsporozoite genotype, and evaluation of intracellular cytokine response to immunization

Appendix J Amendments to the Protocol

**GlaxoSmithKline Biologicals**<br>Clinical Research & Development<br>**Protocol Amendment Approval**	
**eTrack study number**	104743
**eTrack abbreviated title**	Malaria-044
**IND number**	BB-IND 11220
**Protocol title:**	A Phase IIb randomized, double-blind, controlled study of the safety, immunogenicity and proof-of-concept of RTS,S/AS02A, and RTS,S/AS01B, two candidate malaria vaccines in malaria-experienced adults living in Western Kenya.
**Amendment number:**	Amendment 1
**Amendment date:**	24 June 2005 (Final)
**Co-ordinating author:**	Conor Cahill, Scientific Writer

Rationale/background for changes:

At the request of the Human Subjects Research Review Board (HSRRB), a number of changes have been made to the text including the following:

The affiliation of the HSRRB has been corrected to USRMC

The email address for reporting an SAE to HSRRB has been corrected

The Kenya National Ethical Review Committee has been added as a study contact for reporting of an SAE

An alternative clearance regimen, Coartem®, has been added for subjects that cannot tolerate Malarone®

A rationale for why no HIV testing is carried out as part of the screening procedures has been added.

Risks of receipt of vaccines, clearance drug regimens and breach of subject confidentiality in the study have been added to the protocol. Benefits to study volunteers have been added.

Contraindications for further vaccination have been amended to include acute allergic reaction following vaccination

Clarification that there will be no HIV testing carried out in this study has been added.

'Significant IgE-mediated events' have been added as exclusion criteria and criteria for the temporary suspension of vaccination

Text has been added to clarify the roles and responsibilities of the LSM.

'Unanticipated problems' will be reported for this trial. A definition of 'unanticipated problems' has been provided as well as instructions for how they are to be reported.

A new appendix has been added to the protocol to provide a description of drugs to be used for the clearance of malaria parasites

A new appendix has been added to the protocol to provide a description of the roles and responsibilities of study personnel.

A number of errors/omissions in the original version of the document have been corrected in this version. These include the following:
Two additional names have been added to the USAMRU-K team and the spelling of one investigator's name corrected.<br><br>The fax number of the GSK Medical Monitor has been corrected<br><br>The text relating to the submission of protocol modifications to the IRBs/IECs has been corrected both in the main protocol text and in the relevant Appendix. Previously it was stated that protocol modifications are submitted to the IRB/IEC for information only. The IRB/IEC must receive any protocol amendments/modifications and express a favorable opinion of it before it is applied.<br><br>It is intended that for all study visits that are not carried out at the clinic, the subjects will be seen by a field worker or some other qualified study personnel. The fact that these visits may not necessarily be carried out solely by field workers has been clarified throughout the protocol.<br><br>An inclusion criterion that all volunteers should be healthy (as established by clinical history and medical examination) in order to be enrolled in the trial was inadvertently left out of the protocol. This has been added.<br><br>Text has been added to clarify that only those pages of the informed consent documentation that are signed by each volunteer will be filed at the investigator's site.<br><br>In order to ensure that the Safety monitoring plan for the trial is practical a number of clarifications have been added:<br><br>• While the members of the SMG are representatives of WRAIR, KEMRI and GSK Biologicals, clarification has been added that the members of the SMG are not members of the investigational teams at those institutions.<br><br>• It is not intended to present data relating to the duration of symptoms in the safety reports to be written on the first 60 subjects to receive Dose 1 and Dose 2.<br><br>• Text detailing that the SMG may request Study Reports to be unblinded if they deem it to be necessary has been added.<br><br>• Previously it was stated that the SMG may stop the trial permanently. It was intended that the decision to stop the trial permanently should rest with the sponsor, USAMMDA (in consultation with GSK Biologicals)<br><br>The drug regimen for the clearance of malaria parasites from all subjects one week prior to Dose 3 of vaccines has been corrected. Previously some sections of the protocol stated that one dose of Malarone® consisted of one tablet. It is intended that each dose of Malarone® should consist of four tablets. This has been corrected throughout the documentation.<br><br>It is intended that subjects who have a positive MBF during the ADI portion of the study should receive treatment for asymptomatic malaria within 72 hours of the MBF being taken, not 36 hours as stated in the protocol.<br><br>Corrections have been made to the 'list of study procedures' table (Table 27)<br><br>• Morbidity surveillance/recording of SAEs for study procedures was inadvertently left out of the original document. This has been added.<br><br>• Clinic Visit 6 (Day 66) was incorrectly indicated as a visit for ADI. It is not intended to start ADI surveillance until the visit following Day 66. The visit procedures have been corrected accordingly.<br><br>• Footnotes have been corrected for Clinic Visit 1, 6, 7 and 8<br><br>• Parasite genotyping has been added as a study procedure at Clinic Visit 2 (Day 0) to allow for a baseline measurement to be analyzed from all subjects<br><br>The table indicating intervals between study visits has been corrected (Table 28).

The text relating to the detailed description of study visits has been corrected to ensure that it is consistent with the outline of study procedures (Table 27). In addition a number of procedures, namely recording of pulse and time of vaccination have been removed from this section. These procedures may be carried out by the investigator, but are not recorded on the CRF.

The text relating to the dosage and administration of Rabipur rabies vaccine has been corrected. This is to reflect the fact that for this trial, 'long' and 'short' needles will not be supplied with this vaccine in order to facilitate blinding of subjects. In addition the Rabipur diluent will be supplied in ampoules for this trial.

There was an error in the text relating to the preparation of Investigator safety reports. The text has been corrected to ensure that Investigator safety reports are prepared by the Sponsor (USAMMDA) in accordance with FDA policy (not GSK policy as previously stated)

In order to ensure that toxicity grading is in line with recent draft guidance from the FDA, the intensity scales for the monitoring of hepatic and renal function have been amended.

Instructions for handling cells for cell-mediated immunity assay have been added to the Appendices

A number of typographical errors have been corrected

Amended text has been included in bold italics **in the following sections:**

Cover pages:

- ***Steve Ntoburi, Clinical Investigator, USAMRU-K***
- ~~Sean~~ ***Shon Remich, Clinical Investigator, USAMRU-K***
- ***John Waitumbi, Laboratory Director, USAMRU-K***

2. Medical Monitor

Amanda Leach,
Clinical Development Manager,
GlaxoSmithKline Biologicals,
Rue de l'Institut 89, 1330 Rixensart,
Belgium.
Tel: +32.2.656.77.88
Fax: +32.2.656.~~61.60~~ ***80.44***

4. Study Contacts for Reporting of a Serious Adverse Event

Human Subjects Research Review Board (HSRRB)

~~US Army Surgeon General's~~ ***US Army Medical Research and Materiel Command's*** Human Subjects Research Review Board,
USA.
Tel : +001.310.619.21.65
Fax : +001.319.619.78.03
email : hsrrb@~~det~~.amedd.army.mil

USAMRMC Deputy for Reporting Serious Adverse Events

US Army Medical Research and Materiel Command,
ATTN: MCMR-ZB-~~QH~~ ***P***,
504 Scott Street,
Fort Detrick, MD 2170~~1~~ ***2***-5012,
USA.
Tel: +001.301.619.2165/6
Fax: +001.301.619.7803
email: hsrrb@amedd.army.mil

WRAIR Office of Research Management

Walter Reed Army Institute of Research
Office of Research Management
503 Robert Grant Avenue
Silver Spring, MD 20910-7500
USA
Tel: +001.301.319.9940
Fax: +001.301.319.9961
email: jody.ference@na.amedd.army.mil

Kenya National Ethical Review Committee

The Chairman,
Kenya National Ethical Review Committee,
c/o Kenya Medical Research Institute,
PO Box 54840, Nairobi, Kenya.
Tel : +254.20.272.25.41
email : mwasunna@nairobi.mimcom.net

Back-up Study Contact at GSK Biologicals for Reporting Serious Adverse Events
Manager Clinical Safety Vaccines

GSK Biologicals Clinical Safety Physician,
GlaxoSmithKline Biologicals,
Rue de l'Institut 89, 1330 Rixensart,
Belgium.
Tel: +32.2.656.87.98
Fax: +32.2.656.80.09
email: rix.ct-safety-vac-@gskbio.com

Synopsis:

- One week prior to Dose 3, subjects will be treated with Malarone® for presumptive clearance of malaria parasitemia ***(in the event that subjects are unable to tolerate Malarone®, Coartem® (artemether/lumefantrine, Novartis AG, Zurich, Switzerland) will be used as the alternative clearance treatment***

List of abbreviations:
HSRRB: ~~US Army Surgeon General's~~ ***US Army Medical Research and Materiel Command's*** Human Subjects Research Review Board

Section 1.6: ***HIV testing at screening***
As part of this trial, no HIV testing is scheduled to be carried out at screening. It is a condition of the trial that all enrolled subjects are in good health (refer to Section 4.3), as confirmed by a range of clinical and blood tests at screening (refer to Section 4.4 for the exclusion criteria for this trial). Those volunteers who exhibit clinical features of immunosuppresion or immunodeficiency will not be enrolled (refer to Section 4.4). There is no reason to believe that any of the vaccines to be administered during this study may have any ill-effect on clinically asymptomatic volunteers infected with HIV.
In addition, HIV testing has been found to be culturally unacceptable at the site. The local community have hesitated to embrace HIV testing and local elders have voiced their concerns about mandatory HIV testing as part of any studies in this community.

Section 1.7: ***Risks and benefits for study volunteers***
Section 1.7.1: ***Risks of receipt of RTS,S/AS02A, RTS,S/AS01B or Rabies Vaccine and risk-management***
Recipients of any of the investigational products in this study (RTS,S/AS02A, RTS,S/AS01B or Rabies Vaccine) may experience pain and/or swelling at the injection site, fever, headache, fatigue, nausea, vomiting and/or abdominal pain, joint pain and/or muscle aches. As with any vaccination, there is the potential for an anaphylactic reaction. Additionally, there may other reactions that at this time are not known.
To facilitate management of these potential problems, medical staff experienced in the management of anaphylactic reaction will observe patients for at least 30 minutes following each vaccination to ensure that should any anaphylactic reaction occur it is managed in a timely manner (refer to Section 5.3). Subjects will be followed up daily by study staff for 6 days following each dose of investigational product in order to evaluate and arrange treatment (where necessary) of any post-vaccination symptoms (refer to Section 5.4).

Section 1.7.2: ***Risk of receipt of Malarone and risk management***
Subjects who receive Malarone® may experience tiredness, abdominal pain, headache, nausea, vomiting hair loss, mouth ulcers, and diarrhea.
Subjects will be seen by field workers on the day following Dose 1 and Dose 2 of Malarone®, and by a study clinician one week post Dose 3 of Malarone®. Each dose of Malarone® will be given under the supervision of a fieldworker. Any subjects who are unwell at these visits will be referred to a study clinician for evaluation and treatment. Subjects who are unable to tolerate Malarone® will be given Coartem® (refer to Section 5.10).

**Section 1.7.3:** ***Risk of receipt of Coartem and risk management***<br>***Subjects who receive Coartem® may experience abdominal pain, anorexia, nausea, vomiting, diarrhea, headache and/or dizziness.***<br>***For those subjects who receive Coartem® as an alternative clearance treatment, they will be seen by field workers on the day following the first two doses and the second two doses of Coartem, and by a study clinician one week post the final two doses of Coartem®. Dose 1, Dose 3 and Dose 5 of Coartem® will be given under the supervision of a fieldworker. Any subjects who are unwell at these visits will be referred to a study clinician for evaluation and treatment.***
**Section 1.7.4:** ***Risk of disclosure of volunteer's confidential/personal information to persons not involved in the study***<br>***In order to ensure that all information collected on study volunteers is kept confidential, the following safeguards will be applied:***<br>• ***Access to study files and personal information will be limited to only WRP and GlaxoSmithKline personnel with a need to access the information.***<br>• ***Study information will be kept in locked cabinets when not in use.***<br>• ***All information or samples that leave WRP will be labeled in a way that it cannot be linked back to a specific study subject.***
**Section 1.7.5:** ***Benefits to study volunteers of taking part in the study***<br>***All volunteers for this study will receive the following benefits for their participation:***<br>• ***All volunteers will undergo a medical examination at screening free of charge. All volunteers, whether accepted for enrollment into the trial or not will benefit from this free health check-up. The results of all tests will be communicated to all volunteers. Where illnesses are newly-diagnosed, a referral to an appropriate specialist will be made for the volunteer.***<br>• ***For the duration of their participation in the trial, all randomized subjects will receive free health care from study medical personnel.***<br>• ***All enrolled subjects will be offered, free of charge, a full three dose course of vaccinations against rabies (except where the principal investigator deems that vaccination is unsuitable for safety concerns). Subjects who are randomized to receive the rabies control vaccine will receive these vaccinations during the course of the trial. Subjects who are randomized to receive RTS,S/AS01B or RTS,S/AS02A will be offered the vaccination course after they have completed all study procedures and returned for the final study visit.***
**Section 3:**<br>• One week prior to Dose 3, subjects will be treated with Malarone® for presumptive clearance of malaria parasitemia ***(in the event that subjects are unable to tolerate Malarone®, Coartem® (artemether/lumefantrine, Novartis AG, Zurich, Switzerland) will be used as the alternative clearance treatment***
**Section 4.3:**<br>• ***Free of obvious health problems as established by medical history and clinical examination before entering into the study.***<br>• If the volunteer is female, she must be of non-childbearing potential, i.e. either surgically sterilized or one year post-menopausal; or, if of childbearing potential, she must be abstinent or have used adequate contraceptive precautions (i.e. ***natural family planning,*** intrauterine contraceptive device; oral contraceptives; diaphragm or condom in combination with contraceptive jelly, cream or foam; Norplant® or DepoProvera®) for 30 days prior to vaccination, have a negative pregnancy test and must agree to continue such precautions for two months after completion of the vaccination series.
**Section 4.4:**<br>• History of allergic reactions ***significant IgE-mediated events*** or anaphylaxis to previous immunizations.<br>***As part of the screening procedure for this trial, no HIV testing will be performed***

Section 4.6.1: The following adverse events (AEs) constitute absolute contraindications to further administration of RTS,S/AS02A, RTS,S/AS01B or ~~Rabies Vaccine~~ ***Rabipur***; if any of these AEs occur during the study, the subject must not receive additional doses of vaccine but may continue other study procedures at the discretion of the investigator (see Section 9). The subject must be followed until resolution of the event, as with any AE (see Section 8.7):

- ~~Anaphylactic reaction~~ ***Acute allergic reaction, significant IgE-mediated event or anaphylactic shock*** following the administration of vaccine investigational product ***(Amended 24 June 2005)***.

Section 5.1.1: No deviations from, or changes to, the protocol should be initiated without prior written consent of the sponsor, USAMMDA, or GSK Biologicals and IRB/IEC favorable opinion of an appropriate amendment ***or administrative change*** except when necessary to eliminate immediate hazards to the subjects ~~or when the change(s) involves only logistical or administrative aspects of the study (e.g., change of monitor[s], telephone number[s]). Modifications are submitted to the IRB/IEC for information only. However, written verification that the modification was submitted should be obtained.~~ Approvals/verifications must be transmitted in writing to GSK Biologicals' Study Monitor by the principal investigator

Section 5.1.2: Each subject's signed informed consent form must be kept on file by the investigator for possible inspection by Regulatory Authorities and/or GSK Biologicals' professional and Regulatory Compliance persons. ***Only the signed Informed Consent Agreement pages of each volunteer will be kept on file at the investigator's site. In addition one entire representative copy of the informed consent documentation will be kept on file by the investigator for reference.*** The subjects should receive a copy of the signed and dated written informed consent form and any other written information provided to the subjects and should receive copies of any signed and dated consent form updates. Any amendments to the written information will be provided to subjects.

Section 5.1.3.1: ***The LSM is required to review all unanticipated problems (refer to Section 8.3) involving risk to subjects or others, serious adverse events and all subject deaths associated with the protocol and provide an unbiased written report of the event. At a minimum, the LSM should comment on the outcomes of the event or problem, and in the case of a serious adverse event or death, comment on the relationship to participation in the study. The LSM should also indicate whether he concurs with the details of the report provided by the study investigator. Reports for events determined by either the investigator or LSM to be possibly or definitely related to participation and reports of events resulting in death should be promptly forwarded to the HSRRB and GSK Biologicals.***

Section 5.1.3.2: The SMG is not a a***n independent*** Data Safety Monitoring Board. It is a group of experienced clinicians, from the organizations involved in the study, that is charged ~~with assisting the PI and LSM with timely~~ and thorough evaluation of the safety reports after Dose 1 and Dose 2. ***The members do not form a part of the investigator groups; i.e. the investigator site, the GSK Biologicals malaria vaccine clinical project team or the investigator group at WRAIR.*** They will be one of the parties responsible for deciding whether to progress to the next dose and whether to resume immunizations if the trial has been suspended. The SMG may, if deemed necessary, convene a meeting with, or request further information from the PI or LSM

Section 5.1.3.3: Safety reports will be written on the first 60 subjects to receive Dose 1 and Dose 2. These reports will be blinded to vaccine allocation and will contain:

- All SAEs and any relationship to vaccines to date;
- For each dose, all Grade 3 solicited and unsolicited symptoms by relationship to vaccine~~ and duration of symptom~~;

Section 5.1.3.4.1: The PI will suspend all vaccination for any of the following SAEs;

- Death or life-threatening SAE which is judged to be related to the study vaccine;
- Anaphylactic shock reaction ***or significant IgE-mediated event*** in an enrolled subject following vaccination.

Section 5.1.3.4.3: The trial may be **temporarily** suspended by the LSM, PI or the SMG. ***The SMG will review all available safety data which may include a full description of reactogenicity. If the SMG judge it to be necessary, the safety data will be unblinded.*** However the final responsibility to recommend whether or not the trial should be stopped **permanently** rests with the ~~SMG~~ ***the sponsor (USAMMDA) in consultation with GSK Biologicals***, after having considered all the safety information available.

Section 5.2.3: Screening will be done on a separate visit from recruiting. Only adults aged 18 to 35 years ***at the scheduled time of first vaccination*** with a written Informed Consent, signed/thumb printed and dated will be eligible to be screened for the trial…

At the screening visit each subject will be given a Subject Number and each will have a clinic record prepared. Subject Numbers will be issued consecutively in the order in which the subjects arrive at the health center. ***This number will be used throughout the study to identify every document and blood sample associated with the volunteer.*** The clinic records will contain the Subject Number, the subject's date of birth, medical history, findings of the physical examination, the date of screening visit, whether the subject was enrolled, and (where applicable) reasons for exclusion from the study. The name of the study will be written on both the ID card and clinic records of enrolled volunteers.

Section 5.7.3: If a volunteer is excluded from the study for a medical condition or laboratory abnormality not previously known to the ~~subject~~ ***volunteer*** they will be referred for care to one of these physicians according to their illness.

Section 5.10: Three weeks prior to the start of surveillance for malaria infection (i.e. 1 week prior to Dose 3), all subjects will be presumptively treated for aymptomatic parasitemia with Malarone® (atovaquone and proguanil hydrochloride, GlaxoSmithKline, Uxbridge, UK). ***(in the event that subjects are unable to tolerate Malarone®, Coartem® (artemether/lumefantrine, Novartis AG, Zurich, Switzerland) will be used as the alternative clearance treatment.*** The subjects will visit field workers for three consecutive days. At each of these visits, the field worker will give the subject ~~one~~ ***four*** tablet***s*** of Malarone® (containing 250 mg atovaquone and 100 mg proguanil hydrochloride), and observe ~~them~~ ***the subject*** taking ***the tablets*** ~~it~~. The first dose of medication will be administered at the field stations on Study Day 53 (Field Worker Visit 32). The second dose will be given under observation the next morning by the field worker team (Study Day 54; Field Worker Visit 33). The third dose (the final one) will be given by the field worker team the following morning (Study Day 55; Field Worker Visit 34). …
For the ADI period, a field worker visit will consist of the subject presenting to a field station or the KC and completing a brief surveillance for infection morbidity questionnaire, which will include the reporting of malaria symptoms and a record of oral temperature. If the subject is well and afebrile, a blood-sample will be taken and examined for malaria parasitemia and evaluated for CBC. Blood slides from the sample will be Giemsa-stained and read at KC to determine the presence of parasites. Treatment for asymptomatic subjects with positive MBF will be sent by a field worker within ~~36~~ ***72*** hours of the when the MBF was taken.

Section 5.12: (for clarity only those procedures that have been added or deleted in this amendment are indicated in this table below. Deletions are marked with ~~strikethrough~~ for this table, additions with underlining)

**Field Worker Visit Code#**			**21-25**			**26-31**	**32-34**		**35-39**		**40, 41**		**42-51**		**52-56**	
**Clinic Visit**	**1**	**2**		**3**	**4**			**5**		**6**[f]		**7**[a]		**8**[a]		**9**
Focused/Symptom directed Physical exam										•						
Check of elimination criteria										•						
Recording of solicited symptoms (Investigator)										•						
Recording of unsolicited AEs up to 1 month post-vaccination by investigator										•						
Recording of unsolicited AEs by Field Workers											•					
History of fever (for ADI)										~~•~~						
Temperature (for ADI)										~~•~~						
Parasite Genotyping	•															

Section 5.12 (footnotes):

~~Shaded areas indicate visits pertaining to ADI~~

[b] Includes analysis of hemoglobin, total white cell count *absolute lymphocyte count* and platelets. Done with safety labs and with MBF to determine parasite count

[j] *SAEs related to study procedures will be collected*

Section 5.13:

Table 28 Intervals between study visits

**Interval**	**Length of interval**
Clinic Visit 5 → Clinic Visit ~~6~~ *7*	21 to 35 days

Clinic Visit 1: Screening

Day –28 to Day 0

- Record oral temperature, blood pressure~~, pulse~~
- Collect 15 mL venous blood sample for measurement of:
  - ~~MBF~~
- ***Record any SAEs that may have occurred as a result of study procedures***

Clinic Visit 2: Vaccination 1
Day 0

Before vaccination:

- Record oral temperature, blood pressure~~, pulse~~
- Collect 15 mL of whole venous blood for the measurement of:
  - ***Malaria Parasite Genotyping (sample to be analyzed from all subjects)***

Vaccination:

- Administer study vaccine Dose 1; record site, date ~~and time~~ of injection.

After vaccination:

- Record blood pressure, ~~pulse,~~ oral temperature at the end of post-vaccination observation period
- ***Record any post-vaccination SAEs***

Field Worker Visit Code Numbers 21 to 25: Daily Post-vaccination follow up visits
Day 1 to Day 5

- Record pulse, oral temperature
- ***Record SAEs experienced by the vaccinee since the last visit***

Clinic Visit 3: Collection of blood sample
Day 6

- Record oral temperature, blood pressure, ~~pulse~~
- ***Record SAEs experienced by the vaccinee since the last visit***

Clinic Visit 4: Vaccination 2
Day 30

Before vaccination:

- Record oral temperature, blood pressure~~, pulse~~

Vaccination:

- Administer study vaccine Dose 2; record site, date ~~and time~~ of injection.

After vaccination:

- Record blood pressure, ~~pulse,~~ oral temperature at the end of post vaccination observation period
- ***Record any post-vaccination SAEs***

Field Worker Visit Code Numbers 26 to 31: Daily Post-vaccination follow up visits
Day 31 to Day 36

- Record ~~pulse,~~ oral temperature
- ***Record SAEs experienced by the vaccinee since the last visit***

Field Worker Visit Code Numbers 32 to 34: Malarone® administration for presumptive clearance of parasites
Day 53 to 55

- Begin three-day Clearance Regimen
  - First Day: Field worker will administer first dose ***(four tablets)*** of Malarone®.
  - Second Day: Field worker will administer ~~third~~ ***second*** dose ***(four tablets)*** of Malarone®.
  - Third Day: Field worker will administer ~~fifth~~ ***third*** dose ***(four tablets)*** of Malarone®.
- ***Record SAEs experienced by the vaccinee since the last visit***

Clinic Visit 5: Vaccination 3
Day 60

Before vaccination:

- Record oral temperature, blood pressure~~, pulse~~
- Collect ~~1~~ ***2½*** mL of whole venous blood for the measurement of:
  - Serology (antibodies to CS)

Vaccination:

- Administer study vaccine Dose 3; record site, date ~~and time~~ of injection.

After vaccination:

- Record blood pressure, ~~pulse,~~ oral temperature at the end of post-vaccination observation period
- ***Record any post-vaccination SAEs***

Field Worker Visit Code Numbers 35 to 39: Daily Post-vaccination follow up visits
Day 61 to Day 65

- Record ~~pulse,~~ oral temperature
- ***Record SAEs experienced by the vaccinee since the last visit***

Clinic Visit 6: Check for clearance of parasitemia
Day 66

- Record oral temperature, blood pressure~~, pulse~~
- ***Record oral temperature, solicit history of fever in the last 24 hours***
- ***Record SAEs experienced by the vaccinee since the last visit***

Field Worker Visit Code Number 40 and 41: ADI visits #1 and 2
Weekly Visits

- ***Record SAEs experienced by the vaccinee since the last visit***

Clinic Visit 7: ADI visit #3 (Clinic Visit)
Day 90

- Record blood pressure~~, pulse~~
- ***Record SAEs experienced by the vaccinee since the last visit***
- Draw 15 mL of blood from asymptomatic patients for:
- ~~antibodies to CS in Plasma (requires 1 mL)~~

Field Worker Visit Code Numbers 42, 43, 44, 45, 46, 47, 48, 49, 50, 51:
ADI visits # 4, 5, 6, 7, 8, 9, 10, 11, 12, 13
Weekly Visits

- ***Record SAEs experienced by the vaccinee since the last visit***

Clinic Visit 8: Final Visit for ADI
Month 6½

- Record blood pressure~~, pulse~~
- ***Record SAEs experienced by the vaccinee since the last visit***
- Draw 15 mL of blood from asymptomatic patients for
- ***Malaria Parasite Genotyping (sample to be analyzed only if MBF result is positive)***

Clinic Visit 9
Month 12

- Record oral temperature, blood pressure~~, pulse~~
- ***Record SAEs experienced by the vaccinee since the last visit***

Section 6.2.3: Disinfect top of ~~diluent~~ ***vaccine*** vial with alcohol swabs and let dry. ~~Using the longer of the 2 needles supplied,~~ inject the entire contents of the diluent ~~vial~~ ***ampoule*** into the vaccine vial. Keeping the syringe and needle in place, the freeze-dried vaccine is dissolved by gently shaking the vial. Wait for 1 minute to ensure complete dissolution of vial contents before withdrawing a sufficient volume to provide a 1.0 mL dose still using the ~~long~~ ***original*** needle and original syringe. The ~~long~~ ***original*** needle should then be replaced with ~~the shorter~~ ***a fresh*** needle for IM injection. The reconstituted vaccine should be used immediately.

Section 6.6: In addition to the vaccine doses provided for the planned number of enrolled subjects, ~~5~~ ***3***% additional doses will be supplied. In case a vaccine dose is broken or unusable, the investigator should replace it with a replacement vaccine dose. If a vaccine dose needs replacement, the envelope with the corresponding treatment number will designate the replacement without unblinding the study using a coded letter system. Although the sponsor, USAMMDA, and GSK Biologicals need not be notified immediately in these cases, documentation of the use of the replacement vaccine must be recorded by the investigator on the vaccine administration page of the CRF and on the vaccine accountability form.

Section 6.9: All antimalarial drugs administered from the beginning of the study until the end of the ADI period (i.e. ~~6½~~ ***4*** months post Dose 3) are to be recorded with generic name of the medication (trade names are allowed for combination drugs, i.e., multi-component drugs), medical indication, total daily dose, route of administration, start and end dates of treatment.

Section 8: The investigator is responsible for the detection and documentation of events meeting the criteria and definition of an adverse event (AE) or serious adverse event (SAE) as provided in this protocol. During the study, when there is a safety evaluation, the investigator or site staff will be responsible for detecting AEs and SAEs, as detailed in this section of the protocol. ***In addition, for this study the investigator is responsible for the timely reporting of unanticipated problems (refer to Section 8.3).***

Section 8.1: Pre-existing conditions or signs and/or symptoms present in a subject prior to the start of the study (i.e. prior to the first study procedure) should be recorded in the medical history section of the subject's CRF.~~Definition of a serious adverse event~~

Section 8.3: ***Unanticipated problems are those problems that are not specifically described in the protocol or other study documents. They are problems that involve risks to subjects and others, although the risk may not result in bodily harm. For example, misplacing a subject's study records containing identifiable private information results in the risk of breach of confidentiality. Confidentiality may or may not be breached, but either way this would be a reportable event. Another example would be administering the wrong agent to a subject at one time point in a series of vaccinations. Risks to others must also be reported. For example, an inadvertent exposure of a household contact in a smallpox vaccine trial would be a reportable event. Problems resulting in risks to members of the research team are also reportable. Reporting will be carried out with forms supplied by the HSRRB. Reports by the investigator should be promptly sent to the US Army Medical Research and Materiel Command's Human Subjects Research Review Board and to the medical monitor at GSK Biologicals; details of the addresses to which reports should be sent are given in Section 8.8.3. Appropriate supporting documents, such as laboratory reports, pathology reports, and discharge summaries should be submitted with the unanticipated problem report. The medical monitor should indicate whether she concurs with the details provided in the investigator's report. A medical monitor report that comments on the outcomes of the event and the relationship of the event to participation in the study must be submitted to the HSRRB within ten calendar days. Follow-up reports should be submitted until resolution of the unanticipated problem.***

Section 8.6.1:

Table 32 Intensity scales for monitoring hepatic and renal function

**Creatinine (Males and Females)**		
Normal range	0.45 to 1.5 mg/dL	
Grade 1	~~> 1.5 and < 1.6~~	*1.1 to 1.5* mg/dL
Grade 2	~~≥ 1.6 and <1.8~~	*1.6 to 2.0* mg/dL
Grade 3	~~≥ 1.8~~	*2.1 to 2.5* mg/dL
**ALT**		
Males	Normal Range: < 60 IU/mL	
Females	Normal Range: < 40 IU/mL	
**Abnormal ALT gradings**		
Grade 1	~~> 1.0 and < 2.5~~	*1.1 to 2.5* times the upper limit of normal
Grade 2	~~≥ 2.5 times and < 4~~	*2.6 to 5.0* times the upper limit of normal
Grade 3	~~≥ 4~~	*5.1 to 10.0* times the upper limit of normal

Section 8.6.2: There may be situations when a SAE has occurred and the investigator has minimal information to include in the initial report to the ~~sponsors and GSK Biologicals~~ ***study contacts for reporting of a serious adverse event (refer to Section 8.8.2)***. However, it is very important that the investigator always makes an assessment of causality for every event prior to transmission of the SAE Report Form to the ~~sponsors and GSK Biologicals~~ ***study contacts for reporting of a serious adverse event***. The investigator may change his/her opinion of causality in light of follow-up information, amending the SAE Report Form accordingly. The causality assessment is one of the criteria used when determining regulatory reporting requirements.

Section 8.7: After the initial AE/SAE report, the investigator is required to proactively follow each subject and provide further information to ~~GSK Biologicals~~ ***study contacts for reporting of a serious adverse event (refer to Section 8.8.2)*** on the subject's condition …

The Sponsor, USAMMDA***, Kenya National Ethical Review Committee*** and GSK Biologicals may request that the investigator perform or arrange for the conduct of supplemental measurements and/or evaluations to elucidate as fully as possible the nature and/or causality of the AE or SAE. The investigator is obliged to assist. If a subject dies during participation in the study or during a recognized follow-up period, ~~GSK Biologicals~~ ***study contacts for reporting of a serious adverse event*** will be provided with a copy of any available post-mortem findings, including histopathology.

Section 8.8: Prompt reporting of serious adverse events and ***unanticipated problems*** to ~~GSK Biologicals, and USAMMDA~~ ***the study contacts for reporting of a serious adverse event***

Section 8.8.1: SAEs will be reported promptly to ~~GSK and USAMMDA~~ ***the study contacts for reporting of a serious adverse event (refer to Section 8.8.2)*** once the investigator determines that the event meets the protocol definition of an SAE. The investigator or designate will fax the SAE reports to ***the study contacts for reporting of a serious adverse event*** ~~GSK Biologicals' and USAMMDA's Study Contacts for Serious Adverse Event Reporting~~ **WITHIN 24 HOURS OF HIS/HER BECOMING AWARE OF THESE EVENTS**. Additional or follow-up information relating to the initial SAE report is also to be reported to the ***study contacts for reporting of a serious adverse event*** ~~GSK Biologicals' and USAMMDA's Study Contacts for Serious Adverse Event Reporting~~ within 24 hours of receipt of such information.

Section 8.8.2: Completion and transmission of serious adverse event reports to G~~SK Biologicals and USAMMDA~~ ***the study contacts for reporting of a serious adverse event***

Once an investigator becomes aware that a SAE has occurred in a study subject, she/he will report the information to ~~GSK and USAMMDA~~ ***study contacts for reporting of a serious adverse event (refer to Section 8.8.2)*** within 24 hours as outlined in Section 8.8.1. The SAE Report Form will always be completed as thoroughly as possible with all available details of the event, signed by the investigator (or designee), and forwarded to ~~GSK and USAMMDA~~ ***study contacts for reporting of a serious adverse event*** within the designated time frames. If the investigator does not have all information regarding an SAE, he/she will not wait to receive additional information before notifying ~~GSK and USAMMDA~~ ***study contacts for reporting of a serious adverse event*** of the event and completing the form. The form will be updated when additional information is received and forwarded to ~~GSK and USAMMDA~~ ***study contacts for reporting of a serious adverse event*** **WITHIN 24 HOURS** as outlined in Section 8.8.1.

Study contacts for reporting of a Serious Adverse Event

HSRRB

~~US Army Surgeon General's~~ ***US Army Medical Research and Materiel Command's*** Human Subjects Research Review Board,
USA.
Tel: +001.310.619.21.65
Fax: +001.319.619.78.03
email: hsrrb@~~det~~.amedd.army.mil

USAMRMC Deputy for Reporting Serious Adverse Events

US Army Medical Research and Materiel Command,
ATTN: MCMR-ZB-~~QH~~ ***P***,
504 Scott Street,
Fort Detrick, MD 2170~~1~~ ***2***-5012,
USA.
Tel: +001.301.619.2165/6
Fax: +001.301.619.7803
email: hsrrb@amedd.army.mil

WRAIR Office of Research Management

Walter Reed Army Institute of Research
Office of Research Management
503 Robert Grant Avenue
Silver Spring, MD 20910-7500
USA
Tel: +001.301.319.9940
Fax: +001.301.319.9961
email: jody.ference@na.amedd.army.mil

Kenya National Ethical Review Committee

The Chairman,
Kenya National Ethical Review Committee,
c/o Kenya Medical Research Institute,
PO Box 54840, Nairobi, Kenya.
Tel: +254.20.272.25.41
email: mwasunna@nairobi.mimcom.net

**Back-up Study Contact at GSK Biologicals for Reporting Serious Adverse Events**<br>**Manager Clinical Safety Vaccines**<br>GSK Biologicals Clinical Safety Physician,<br>GlaxoSmithKline Biologicals,<br>Rue de l'Institut 89, 1330 Rixensart,<br>Belgium.<br>Tel: +32.2.656.87.98<br>Fax: +32.2.656.80.09<br>***email: rix.ct-safety-vac-@gskbio.com***
**Section 8.8.3:** ***Unanticipated problems (refer to Section 8.3) involving risk to volunteers or others, serious adverse events related to participation in the study and all volunteer deaths should be promptly reported by phone, by email, or by facsimile to the U.S. Army Medical Research and Materiel Command's Human Subjects Research Review Board and to the GSK Biologicals Medical Monitor (see below for contact details). A complete written report should follow the initial notification. In addition to the methods above, the complete report can be sent to the addresses given below.***<br>***U.S. Army Medical Research and Materiel Command's Human Subjects Research Review Board***<br>***U.S. Army Medical Research and Materiel Command's Human Subjects Research Review Board,***<br>***ATTN: MCMR-ZB-P, 504 Scott Street,***<br>***Fort Detrick, Maryland 21702-5012,***<br>***USA.***<br>***Tel: +1.301.619.21.65***<br>***Fax: +1. 301.619.78.03***<br>***email: hsrrb@amedd.army.mil***<br>***Medical Monitor***<br>***Amanda Leach,***<br>***Clinical Development Manager,***<br>***GlaxoSmithKline Biologicals,***<br>***Rue de l'Institut 89, 1330 Rixensart,***<br>***Belgium.***<br>***Tel: +32.2.656.77.88***<br>***Fax: +32.2.656.61.60***<br>***email: amanda.leach@gskbio.com***
**Section 8.9:** The investigator will promptly report all SAEs to ~~GSK and USAMMDA~~ ***study contacts for reporting of a serious adverse event (refer to Section 8.8.2)*** in accordance with the procedures detailed in Section 8.8. The Sponsor, USAMMDA has a legal responsibility to promptly notify, as appropriate, both the local regulatory authority and other regulatory agencies about the safety of a product under clinical investigation. Prompt notification of SAEs by the investigator to the Study Contacts for Reporting SAEs is essential so that legal obligations and ethical responsibilities towards the safety of other subjects are met Investigator safety reports are prepared according to ~~GSK policy~~ ***FDA requirements by the Sponsor (USAMMDA)*** and are forwarded to investigators as necessary. An investigator safety report is prepared for a SAE(s) that is both attributable to investigational product and unexpected. The purpose of the report is to fulfil specific regulatory and Good Clinical Practice (GCP) requirements, regarding the product under investigation.

Section 8.11: The investigator, or his designee, will collect pregnancy information on any subject who becomes pregnant while participating in this study. The investigator, or his designee, will record pregnancy information on the Pregnancy Report Form and submit it to ~~GSK and USAMMDA~~ ***study contacts for reporting of a serious adverse event (refer to Section 8.8.2)*** within 24 hours of learning of a subject's pregnancy. The subject will be followed to determine the outcome of the pregnancy. At the end of the pregnancy, whether that be full-term or prematurely, information on the status of the mother and child will be forwarded to ~~GSK and USAMMDA~~ ***study contacts for reporting of a serious adverse event***. Generally, follow-up will be no longer than six to eight weeks following the estimated delivery date …
A spontaneous abortion is always considered to be a SAE and will be reported as described in Section 8.8. Furthermore, any SAE occurring as a result of a post-study pregnancy AND considered reasonably related in time to receipt of the investigational product by the investigator, will be reported to ~~GSK Biologicals and USAMMDA~~ ***study contacts for reporting of a serious adverse event*** as described in Section 8.10. While the investigator is not obligated to actively seek this information from former study participants, he may learn of a pregnancy through spontaneous reporting.

Appendix B:

II. Protocol Amendments and Administrative changes

- Any amendment/administrative change to the protocol will be adhered to by the participating centre(s) and will apply to all subjects. Written IRB/IEC approval of protocol amendments is required prior to implementation.~~; administrative changes are submitted to IRBs/IECs for information only.~~

Appendix C: At KC, volunteers will view a video in their native language that explains the nature and purpose of the study and reviews the entire informed consent document. There will be a period for asking questions afterwards. Volunteers will then receive both oral and written explanations of the study one-on-one with a study coordinator or clinician and again allowed to ask questions. Afterwards, written informed consent will be obtained from each person who wants to participate in the study. ***In addition, all volunteers who sign an informed consent form will be required to fill in a form that will be entered into the US Army Medical Research and Materiel Command Volunteer Registry Data Base. This form requires details of the volunteer's sex, date of birth, permanent home address, permanent home phone number, as well as their local address and phone number if it is different from their permanent details. In the instructions to the volunteer, this form states that the disclosure of each volunteer's Social Security Number (SSN) is mandatory; however, as SSNs are not issued in Kenya, no SSN will be recorded on this form. The intent of the data base is to readily answer questions concerning an individual's participation in research sponsored by USAMRMC and to ensure that the USAMRC can exercise its obligation to ensure research volunteers are adequately warned of risks and to provide them with new information as it becomes available. The information will be stored at USAMRMC for a minimum of 75 years.*** So that language and illiteracy will not be impediments to informed consent, all briefings and explanations will be in Dholuo, the local language.

Appendix D:

Instructions for Handling Cells for Cell-Mediated Immunity Assay

When materials are provided by GSK Biologicals, it is mandatory that all clinical samples be collected and stored using exclusively those materials in the appropriate manner. The use of other materials could result in the exclusion of the subject from analysis. The investigator must ensure that his/her personnel and the laboratory(ies) under his/her supervision comply with this requirement.

1. Collection of whole blood

Collect blood by venipuncture in Terumo tubes with heparin (or equivalent) and record time of collection. The tubes should be kept at room temperature and shipped to a designated clinical site for separation of peripheral blood mononuclear cells (PBMCs). The shipment must be timed to ensure that PBMC separation will be performed within 24 hours. Use well closed Styrofoam boxes of 5 cm thickness for blood samples transport (see current version of GSK Biologicals SOP RD_HCI_001 for guidance).

2. Separation and freezing of PBMCs

PBMCs will be separated on a density gradient, aliquoted and frozen at –80°C for 24 hours and further cryopreserved in liquid nitrogen until testing (see GSK Biologicals SOP: RD_HCI_007 for guidance).

3. Labeling of cryotubes for PBMC samples

- ***If labels are provided by GSK, it is mandatory to use them.***
- ***If necessary, any hand-written additions to the labels should be made using indelible ink.***

4. Sorting and storage of PBMC samples

Samples should be stored in liquid nitrogen until shipment to GSK Biologicals, if needed. Wherever possible, a backup facility for storage of samples should be available.

A standard Cryotube Listing Form (see current version of GSK Biologicals SOP RD_HCI_009 for guidance), specifying the samples being shipped for individual subjects at each time point, should be prepared for each shipment. A copy of this list should be retained at the study site, while the original should be sealed in a plastic envelope and shipped with the PBMC samples.

Appendix F:

Biochemical and hematological analyses

Hematological and biochemical testing will be done at KEMRI in ~~Tanzania~~ ***Kenya***, following laboratory SOPs.

Appendix G:

An additional ~~5~~***3***% of their respective amounts of RTS,S/AS02A, RTS,S/AS01B and Rabipur will be supplied for replacement in case of breakage, bad storage conditions or any other reason that would make the vaccine unusable (i.e. given by mistake to another subject).

Vaccine vials will be labeled with the following information

104743/044 (Malaria-044)
Vaccine name
Lot number: Dose Number
Expiry date: Treatment Number
non dominant deltoid IM adm
Store at 2-8 deg.C (36-46 deg.F). Do not freeze.
Caution: new drug. Limited by USA law to investigational use
MFD: GSK BIOLOGICALS or CHIRON
Sponsor: USAMMDA
Internal GSK number
Pack Number

Appendix H:

Malarone

Malarone® is a fixed-dose combination of the antimalarial agents atovaquone and proguanil hydrochloride. Each tablet contains 250 mg of atovaquone and 100 mg proguanil hydrochloride. For treatment of malaria in adults the dosage is four Malarone® tablets in a single daily dose for three consecutive days. Malarone® tablets come in boxes containing one complete treatment dose (12 tablets in a blister pack per box). The box is labeled with the name of the medication as well as the blister pack inside. Malarone® is manufactured by GlaxoSmithKline.

Coartem

Coartem® is a fixed-dose combination of artemether and lumefantrine. Each tablet contains 20 mg artemether and 120 mg lumefantrine. For treatment of malaria in adults in countries where multi-drug resistant **P. falciparum** ***is present, the dosage is four Coartem® tablets twice daily for three consecutive days. Coartem® is manufactured by Novartis.***

Appendix I:

Investigator	*Title*	*Responsibility*
Mark E Polhemus, MD	*Principal Investigator/ Chief of Clinical Trials, USAMRU-K*	*To conduct the study in compliance with the protocol and appendices. Conduct of the study includes protocol development, logistics, recruiting and screening, supervision of immunization, adverse event reporting, safety report development, final study report development and publication of results.*
Mark R Withers, MD, MPH	*Associate Investigator/ Kisumu Director, USAMRU-K*	*To assist the principal investigator in the conduct of the study including protocol review, assisting in recruiting and screening, assisting with pre-vaccination evaluation, assisting with post-vaccination follow ups, review of AEs and safety reports, and review of publications*
James F Cummings, MD	*Associate Investigator*	
D Gray Heppner, MD	*Associate Investigator*	
Kent Kester, MD	*Associate Investigator*	
Steve Ntoburi MD	*Associate Investigator*	
Christian Ockenhouse, MD, PhD	*Associate Investigator*	
Bernhards Ogutu, MBChB, Mmed (Peds) , PhD	*Associate Investigator*	
Lucas Otieno, MBChB	*Associate Investigator*	
Shon A Remich, MD	*Associate Investigator*	
John Waitumbi DVM, PhD	*Associate Investigator*	
Amos E O Otedo, MD	*Local Safety Monitor*	*Evaluation of SAEs<br>Evaluation of unanticipated AEs<br>Evaluation of safety reports prior to progression to next vaccination<br>Unblinding a subject*
Urszula Krzych	*Research Associate*	*Scientific review of the protocol and coordination and supervision of the research assays required to conduct the study. The research assays include screening for hemoglobinopathies, antibody response to hepatitis B surface antigen, antibody response to circumsporozoite antigen, PCR for circumsporozoite genotype, and evaluation of intracellular cytokine response to immunization*
Ann Stewart	*Research Associate*	

Appendix K Amendments to the Protocol

GlaxoSmithKline Biologicals
Clinical Research & Development
Protocol Amendment Approval

**eTrack study number**	104743
**eTrack abbreviated title**	Malaria-044
**IND number**	BB-IND 11220
**Protocol title:**	A Phase IIb randomized, double-blind, controlled study of the safety, immunogenicity and proof-of-concept of RTS,S/AS02A, and RTS,S/AS01B, two candidate malaria vaccines in malaria-experienced adults living in Western Kenya.
**Amendment number:**	Amendment 2
**Amendment date:**	26 Jul 2005 (Final)
**Co-ordinating author:**	Conor Cahill, Scientific Writer

Rationale/background for changes:

For logistic reasons, the screening interval for the trial is being extended from 4 weeks (28 days) to 6 weeks (42 days)

Amended text has been included in *bold italics* in the following sections:

Section 3: Study Design Overview

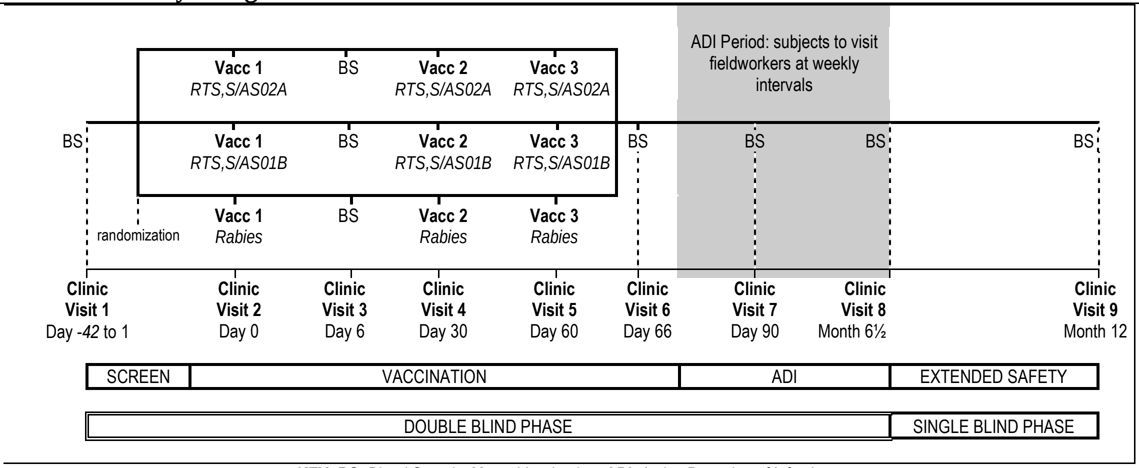

KEY: BS; Blood Sample. **Vacc;** Vaccination. **ADI;** Active Detection of Infection

Amended 26 July 2005

Section 5.2.3: Screening of volunteers

All screening tests will be completed within ~~30~~ ***42*** days prior to entry into the study.

Section 5.12: Outline of study procedures

	Recruit/ Screen	DOUBLE-BLIND PHASE													SINGLE-BLIND PHASE	
		VACCINATION									ADI					
		1			2		X	3								
**Study Day**	**-*42* to 0**	**0**	**1-5**	**6**	**30**	**31-36**	**53-55**	**60**	**61-65**	**66**	**2 weekly visits**	**90**	**10 weekly visits**			
Amended 26 July 2005																

Table 38 Intervals between study visits

**Interval**	**Length of interval**
Clinic Visit 1 → Clinic Visit 2	0 to ~~28~~ *42* days
Clinic Visit 2 → Clinic Visit 4	21 to 35 days
Clinic Visit 4 → Clinic Visit 5	21 to 35 days
Clinic Visit 5 → Clinic Visit 7	21 to 35 days
Clinic Visit 5 → Clinic Visit 8	4 months ± 1 month
Clinic Visit 8 → Clinic Visit 9	6 months ± 1 month
Clinic Visit 5 → Clinic Visit 9	9 months ± 2 months

Amended 26 July 2005

Section 5.13: Detailed description of study visits

Clinic Visit 1: Screening
Day – -~~28~~*42* to Day 0

Appendix L Amendments to the Protocol

<table>
<tr><td colspan="2">GlaxoSmithKline Biologicals
Clinical Research & Development
Protocol Amendment Approval</td></tr>
<tr><td>eTrack study number</td><td>104743</td></tr>
<tr><td>eTrack abbreviated title</td><td>Malaria-044</td></tr>
<tr><td>IND number</td><td>BB-IND 11220</td></tr>
<tr><td>Protocol title:</td><td>A Phase IIb randomized, double-blind, controlled study of the safety, immunogenicity and proof-of-concept of RTS,S/AS02A, and RTS,S/AS01B, two candidate malaria vaccines in malaria-experienced adults living in Western Kenya.</td></tr>
<tr><td>Amendment number:</td><td>Amendment 3</td></tr>
<tr><td>Amendment date:</td><td>13 June 2006 (Final)</td></tr>
<tr><td>Co-ordinating author:</td><td>Sarah Benns, Scientific Writer</td></tr>
<tr><td colspan="2">Rationale/background for changes:</td></tr>
<tr><td colspan="2">In order to assess if CMI response is preserved 9 months after completion of vaccination, an additional blood sample for CMI analysis will be taken at Month 12, Clinic Visit 9.

It is proposed to carry out an ancillary study in collaboration with the London School of Tropical Medicine and Hygiene to use molecular genetic typing methods to determine two important parasitological parameters a) if there is detectable strain-specificity in the effect of the vaccine b) if vaccination alters the multiplicity of subsequent infections. Both these parameters will be examined in both incident and prevalent infections.</td></tr>
<tr><td colspan="2">Amended text has been included in bold italics in the following sections:</td></tr>
<tr><td colspan="2">Cover Pages</td></tr>
<tr><td colspan="2">London School of Hygiene and Tropical Medicine</td></tr>
<tr><td colspan="2">• Brian Greenwood
• Colin Sutherland</td></tr>
<tr><td colspan="2">Kenya Medical Research Institute</td></tr>
<tr><td colspan="2">• Sammy Anyona</td></tr>
<tr><td colspan="2">Synopsis</td></tr>
<tr><td colspan="2">Rationale
The proposed study will also use molecular genetic typing methods to determine two important parasitological parameters: a) if there is detectable strain-specificity in the effect of the vaccine, and b) if vaccination alters the multiplicity of subsequent infections.</td></tr>
</table>

Objectives

Exploratory: Parasite Genotyping

- ***To determine the prevalence of vaccine-like alleles of CSP in the study population.***
- ***To investigate whether vaccination with RTS,S/AS0A2 and RTS,S/AS01B modifies the distribution of amino acid substitutions in the Th2R and Th3R sequence regions of the CSP gene in subsequent P. falciparum infections.***
- ***To investigate whether vaccination with RTS,S/AS0A2 and RTS,S/AS01B modifies the number of P. falciparum genotypes (multiplicity of infection) as determined by analysis of msp-1 and msp-2 genes.***

Study design

DNA will be extracted from EDTA blood of all subjects with patent asexual parasitemia.

CS-specific CMI will be assessed at ~~***three***~~ ***four*** time points during the study; prevaccination, 1 month post Dose 3, ~~***and***~~ 4 months post Dose 3 ***and 9 months post Dose 3***.

Exploratory endpoints: Cell Mediated Immunity

Endpoint assessed prior to vaccination, 1 month post Dose 3, ~~***and***~~ 4 months post Dose 3 ***and 9 months post Dose 3***.

Exploratory endpoints: Parasite Genotyping

- ***Sequencing of the Th2R and Th3R epitope regions in parasite-positive individuals identified by CSP PCR amplification of Day 0 DNA samples.***
- ***DNA samples prepared at first microscopy confirmed infections identified after the third vaccination, used to determine the distribution of amino acid substitutions in the Th2R and Th3R sequence regions of the CSP gene.***
- ***DNA samples prepared at first microscopy confirmed infection identified after the third vaccination, used to determine the number of P. falciparum genotypes (multiplicity of infection) as determined by analysis of msp-1 and msp-2 genes.***

LIST OF ABBREVIATIONS

LSHTM	***London School of Hygiene and Tropical Medicine***
MOI	***Multiplicity of Infection***

Section 1.6. Rationale for Parasite Genotyping evaluation

It is proposed to carry out an ancillary study in collaboration with the London School of Hygiene and Tropical Medicine (LSHTM) to use molecular genetic typing methods to determine two important parasitological parameters a) if there is detectable strain-specificity in the effect of the vaccine b) if vaccination alters the multiplicity of subsequent infections. Both these parameters will be examined in both incident and prevalent infections.

The CSP is the predominant protein found in the surface of the sporozoite. Studies of the genetic diversity of the gene encoding the CSP of P. falciparum have demonstrated the existence of high levels of genetic polymorphisms within the region known to contain T-cell epitopes in isolates from different areas in Africa [Escalante, 2002], which is therefore potentially important for generation of immunity. This has raised the concern whether a vaccine containing a sequence of a selected strain of P. falciparum would confer protection against other variant parasites. In the Gambia, the RTS,S/AS02A vaccine was demonstrated to induce protection against first P. falciparum infections that was not strain-specific. The frequency of the vaccine allele type, which was derived from the sequence of the CSP allele in laboratory clone 3D7, was similar to that of all other CSP alleles studied in the vaccine vs control group [Allloueche, 2003]. The key polymorphic sites in the CSP gene which are encompassed by the RTS,S antigen are the T-cell epitopes at the carboxy-terminus of the protein, designated Th2R and Th3R. Polymorphisms in these epitopes were the focus of the evaluation of strain-specificity of vaccine efficacy in the Gambian trial [Alloueche, 2003]. Evidence was also found in that study that RTS,S modified the average number of clones (multiplicity) carried during post-vaccination parasitemia. Modification of multiplicity of P. falciparum infections was also found after vaccination with the chimaeric peptide multimer vaccine SPf66 [Haywood, 1999]. These findings were confirmed by recent analysis of CSP sequences and clone multiplicity in 521 parasite isolates from Mozambiquan children who participated in a large Phase II study of the efficacy of RTS,S/AS02A [Alonso, 2004; Enosse, 2006].

To evaluate strain-specific vaccine effects in this trial of the RTS,S/AS02A and RTS,S/AS01B vaccines in Kenyan adults, DNA samples will be used for sequence analysis of the Th2R and Th3R sequences in the CSP gene of parasitemic individuals identified during follow-up by active detection of infections (ADI). Vaccine effects on multiplicity of infection (MOI) will also be investigated using polymorphic antigens loci, namely msp1 and msp2.

Section 2.4.1. Exploratory Objectives; Parasite Genotyping

- ***To determine the prevalence of vaccine-like alleles of CSP in the study population.***
- ***To investigate whether vaccination with RTS,S/AS0A2 and RTS,S/AS01B modifies the distribution of amino acid substitutions in the Th2R and Th3R sequence regions of the CSP gene in subsequent P. falciparum infections.***
- ***To investigate whether vaccination with RTS,S/AS0A2 and RTS,S/AS01B modifies the number of P. falciparum genotypes (multiplicity of infection) as determined by analysis of msp-1 and msp-2 genes.***

Refer to Section 10.4 for definition of the exploratory endpoints.

Section 3. STUDY DESIGN OVERVIEW

DNA will be extracted from EDTA blood of all subjects with patent asexual parasitemia.

CS-specific CMI will be assessed at ~~three~~ ***four*** time points during the study; prevaccination, 1 month post Dose 3, ~~and~~ 4 months post Dose 3 ***and 9 months post dose 3***.

Section 5.12. Outline of study procedures

Table 27 List of study procedures

	Recruit/ Screen	DOUBLE-BLIND PHASE													SINGLE-BLIND PHASE	
		VACCINATION									ADI					
		1			2		X	3								
**Study Day**	-42 to 0	0	1-5	6	30	31-36	53-55	60	61-65	66	2 weekly visits	90	10 weekly visits			
**Study Month**		0			1			2				3		6½	7 to 11	12
**Clinic Visit**	1	2		3	4			5		6[f]		7[a]		8[a]		9
**STUDY PROCEDURES**																
Cell-mediated immunity		•										•		•		•

Section 5.13. Detailed description of study visits

FINAL STUDY VISIT, Clinic Visit 9, Month 12

- Draw ***4 13*** mL of blood from asymptomatic patients for
  - CBC
  - Serology (antibodies to CS and HBs) (requires 3 mL)
  - ***Cell-mediated immunity (requires 9 mL)***

Section 5.14.2 Laboratory assays

DNA extraction, PCR and determination of P. falciparum genotypes (MOI) will be performed at the Walter Reed laboratory, Kisumu, Kenya. The determination of CSP polymorphic sequences will be done at HPA Malaria Reference Laboratory, LSHTM, UK.

Table 30 Summary of blood sampling timepoints/immunological assays

Blood sampling timepoint			Test	No. subjects	Laboratory	Priority Rank
Timing	Timepoint	Clinic Visit No.				
Dose 1	Day 0	2	Anti-CS antibodies	255	GSK Bio*	1
			Anti-HBs antibodies	255	GSK Bio*	3
			CMI	255	KEMRI/ GSK Bio/ WRAIR	2
Dose 3	Day 60	5	Anti-CS antibodies	255	GSK Bio*	1
Post Dose 3	Day 90	7	Anti-CS antibodies	255	GSK Bio*	1
			Anti-HBs antibodies	255	GSK Bio*	3
			CMI	255	KEMRI/ GSK Bio/ WRAIR	2
Post Dose 3	Month 6½	8	Anti-CS antibodies	255	GSK Bio*	1
			CMI	255	GSK Bio*	2
Post Dose 3	Month 12	9	Anti-HBs antibodies	255	GSK Bio*	2
			Anti-CS antibodies	255	GSK Bio*	1
			CMI	*255*	*KEMRI/ GSK Bio/ WRAIR*	*2*

*or designated laboratory
CMI: cell-mediated immunity

DNA will be extracted from 200 µL of the EDTA blood using QIAmp DNA blood mini kit as described by the manufacturer (QIAGEN Inc., CA). DNA samples will be stored at -80 °C until use. Details for PCR analysis, determination of CSP polymorphic sequences, genotyping of msp-1 and msp-2 and detection of multiplicity of infection are provided in Appendix F.

Section 10.4.3. Parasite Genotyping

- ***Sequencing of the Th2R and Th3R epitope regions in parasite-positive individuals identified by CSP PCR amplification of Day 0 DNA samples.***
- ***DNA samples prepared at first microscopy confirmed infections identified after the third vaccination, used to determine the distribution of amino acid substitutions in the Th2R and Th3R sequence regions of the CSP gene.***
- ***DNA samples prepared at first microscopy confirmed infection identified after the third vaccination, used to determine the number of P. falciparum genotypes (multiplicity of infection) as determined by analysis of msp-1 and msp-2 genes.***

Section 10.8.5. Analysis of parasite genotyping

The main endpoint for assessing the strain specificity of the vaccine will be the relative proportion of the vaccine type (3D7 / NF54) in the vaccine vs. control group. For each infection, the predominant allele will be counted in the analysis in two ways. Firstly, the number of polymorphic amino acid positions that differ from the vaccine sequence will be scored for each isolate, and tested. Differences among the three treatment groups will be tested for significance by the Wilcoxon Rank SumTest. Secondly, the prevalence of the vaccine-like amino acid residue at each polymorphic site will be compared between treatment groups using the Fisher's Exact test. If two alleles are equally abundant in an infection, a random number table will be used to determine which allele goes into the analysis.

Between group comparison of the multiplicity of infection of emergent infections will be performed as described in the analyses of previous vaccine trials [Alloueche 2003; Haywood 1999; Enosse, 2006]. Briefly, to determine whether the vaccine reduces the number of P. falciparum genotypes, the number of genotypes in each isolate will be taken as the number of alleles of msp1 or msp2, whichever is the greater. The differences between mean MOI in the vaccinated vs. control groups will be compared using the Wilcoxon test. Regression analysis will be used to assess the effect of the vaccine on multiplicity of infection adjusted for geometric mean parasite density and age.

Section 12. REFERENCES

Alloueche A, Milligan P, Conway DJ et al. Protective efficacy of the RTS,S/AS02A Plasmodium falciparum malaria vaccine is not strain specific. Am J Trop Med Hyg 68 (1); 2003: 97–101.

Alonso PL, Sacarlal J, Aponte JJ, Leach A, Macete E et al. Efficacy of the RTS,S/AS02A vaccine against Plasmodium falciparum infection and disease in young African children: randomised controlled trial. Lancet. 2004;364:1411-1420.

Escalante AA, Grebert HM, Isea R, et al. A study of genetic diversity in the gene encoding the circumsporozoite protein (CSP) of Plasmodium falciparum from different transmission areas--XVI. Asembo Bay Cohort Project. Mol Biochem Parasitol. 2002;125(1-2):83-90.

Enosse S, Doban˜o C, Quelhas D, et al. RTS,S/AS02A malaria vaccine does not induce selection of parasites encoding divergent CSP T-cell epitopes and reduces the genotypic multiplicity of Plasmodium falciparum infections. PLoS Clin Trials 2006; 1: e5.

Haywood M, Conway DJ, Weiss H, et al. Reduction in the mean number of Plasmodium falciparum genotypes in Gambian children immunized with the malaria vaccine SPf66. Transactions of the Royal Society of Tropical Medicine and Hygiene 1999; 93 (1), 65-68.

Snounou G, Zhu X, Siripoon N, et al. Biased distribution of msp1 and msp2 allelic variants in Plasmodium falciparum populations in Thailand. Transactions of the Royal Society of Tropical Medicine and Hygiene 1999; 93 (4), 369-374.

Appendix F Laboratory Assays

Cell-mediated immunity Methodology

T-cell mediated immunity (CMI) will be investigated in this study. CMI will be assessed at ~~three~~ ***four*** time points during the study, corresponding to prevaccination, 1 month post Dose 3, ~~*and*~~ 4 months post Dose 3 ***and 9 months post Dose 3***. A sample volume of 9 mL will be taken at each of these timepoints.

Parasite Genotyping

All laboratory work will be performed by individuals blinded to vaccine assignment. Vaccine code will be assigned after completion of analyses. DNA extraction, PCR and determination of P. falciparum genotypes (MOI) will be performed at the Walter Reed laboratory, Kisumu, Kenya. The determination of CSP polymorphic sequences will be performed in the Dept of Infectious & Tropical Diseases, LSHTM, UK.

DNA extraction

EDTA blood will be collected during ADI from individuals with microscopically confirmed asexual parasitemia. DNA will be extracted from 200 μL of the EDTA blood using QIAmp DNA blood mini kit as described by the manufacturer (QIAGEN Inc., CA). DNA samples will be stored at -80°C until use.

Polymerase chain reaction

Primers that bind to conserved sequences flanking the Th2R and Th3R region of P. falciparum CSP gene will be used in a nested PCR to amplify a 381 base pair fragment covering nucleotide 936 to 1317 of the CSP gene. For the first round PCR, the following primers will be used:

First round PCR:

Forward primer (CSPf1) - 5'-caattcatgatgagaaaattagctatt-3'

Reverse primer (CSPr1) - 5'-actcaaactaagatgtgttc -3'.

Second round PCR:

Forward primer (CSPf2) - 5'- cctaataaaaacaatcaagg-3'

Reverse primer (CSPr2) -5'- tctaattaaggaacaagaaggat -3'

The composition of 1st PCR reaction mixture is shown below:

Reagent	*Final concentration*	*Unit reaction (μL)*	*X ---100-- samples*
10x PCR buffer	*1x*	*2 μL*	*200*
MgCl2 (25 mM)	*2.5 mM*	*2 μL*	*200*
dNTPs (10 mM)	*250 nM*	*0.5μL*	*50*
Forward primer (CSPf1) 20 μM	*0.3 μM*	*0.3 μL*	*30*
Reverse primer (CSPr1) 20 μM	*0.3 μM*	*0.3 μL*	*30*
AmpliTaq gold polymerase (5 U/ml)	*1 U*	*0.2 μL*	*20*
DNA		*5 μL*	*-*
PCR H2O	*q.s to 20 μL*	*9.7 μL*	*970*
Total		*20 μL*	*2000*

The 2nd PCR reaction mixture will contain 1 μL of primary amplicons in a total volume of 20 μL as shown below:

Reagent	*Final concentration*	*Unit reaction(μL)*	*X ---100 samples*
10x PCR buffer	*1x*	*2 μL*	*200*
$MgCl_2$ (25 mM)	*2.5 mM*	*2 μL*	*200*
dNTPs (10 mM)	*250 nM*	*0.5 μL*	*50*
Forward primer (CSPf1) 20 μM	*0.3 μM*	*0.3 μL*	*30*
Reverse primer (CSPr1) 20μM	*0.3 μM*	*0.3 μL*	*30*
Taq gold polymerase (5 U/ml)	*1 UI*	*0.2 μL*	*20*
DNA		*1 μL*	*-*
PCR H2O	*q.s to 20 μL*	*13.7 μL*	*1370*
Total		*20 μL*	*2000*

PCR amplifications will be performed in a DNA Tetrad Engine (MJ research) 96-well thermocycler in the following cycling conditions: denaturation during 3 min at 95ºC, followed by 40 cycles of 94ºC (1 min), 60ºC (1 min) and 72ºC (1 min) and final extension at 72ºC for 10 min. 5 μL 2nd round PCR products are monitored on a 2.0% agarose gel in 1xTAE buffer to check the quality, size and yield of the PCR products before proceeding to product purification and sequencing. The following samples will also be included in every run: Positive control = P. falciparum culture DNA; Negative control 1 = uninfected host genomic DNA; Negative control 2 = no temperate control.

QA/QC:

For QA/QC reasons, if the positive control sample doesn't amplify or the negative control samples amplify, the whole run will be repeated.

Determination of CSP polymorphic sequences

The distribution of polymorphic variants within the Th2R and Th3R regions of P falciparum CSP will be determined using high-throughput direct sequencing of the PCR products. The sequencing strategy will be validated in a pilot study of 50 PCR samples randomly selected from pre-intervention blood samples from study participants. This will also provide important information regarding the pattern of CSP polymorphism in the study area, and an estimate of the expected data loss due to ambiguous sequence reads from mixed alleles. The sequencing using CSPf2 and CSPr2 primers will be performed in an ABI 3730 capillary sequencer (provided by the GMP). The sequence of each PCR product will be manually checked by at least two investigators and a consensus reached or the assay repeated. Data will be collated from all isolates with a single CSP allele, or where a clear majority allele can be unambiguously identified. For those isolates in which a clear majority allele is not determined from the sequence electropherogram, the majority allele will be determined by cloning of PCR products and sequencing.

Genotyping of msp-1, msp-2 and detection of multiplicity of infection (MOI)

Allele specific, nested PCR of 3 polymorphic regions of P. falciparum genes, namely msp1 block 2 and msp2 will be used to detect the genetic structure of the parasite populations, essentially as described by Snounou, 1999. Following electrophoresis, staining with ethidium bromide and observation under ultra-violet illumination, bands corresponding to different parasite allelic forms will be distinguished and counted and the number of genotypes for msp1 and msp2 loci determined. Gels will be double-scored independently by 2 investigators, and either consensus reached or the assay repeated. To gain a preliminary estimate of MOI in the study population, this genotyping will be performed on the 50 samples used in the sequencing pilot.

GlaxoSmithKline Biologicals
Clinical Research & Development
Protocol Amendment Approval

**eTrack study number**	104743
**eTrack abbreviated title**	Malaria-044
**IND number**	BB-IND 11220
**Protocol title:**	A Phase IIb randomized, double-blind, controlled study of the safety, immunogenicity and proof-of-concept of RTS,S/AS02A, and RTS,S/AS01B, two candidate malaria vaccines in malaria-experienced adults living in Western Kenya.
**Amendment number:**	Amendment 3
**Amendment date:**	13 June 2006 (Final)

Approved by:
Sponsor Signatory title

Amanda Leach — dd-mm-yyyy
Clinical Development Manager

**GlaxoSmithKline Biologicals**<br>Clinical Research & Development<br>**Protocol Amendment Approval**	
**eTrack study number**	104743
**eTrack abbreviated title**	Malaria-044
**IND number**	BB-IND 11220
**Protocol title:**	A Phase IIb randomized, double-blind, controlled study of the safety, immunogenicity and proof-of-concept of RTS,S/AS02A, and RTS,S/AS01B, two candidate malaria vaccines in malaria-experienced adults living in Western Kenya.
**Amendment number:**	Amendment 3
**Amendment date:**	13 June 2006 (Final)
**Agreed by:**<br>Investigator	______________________ Mark E Polhemus MD __________ dd-mm-yyyy
